# Outstanding Multi‐Photon Absorption at π‐Delocalizable Metallodendrimers

**DOI:** 10.1002/anie.202116181

**Published:** 2022-01-20

**Authors:** Ling Zhang, Mahbod Morshedi, Mark G. Humphrey

**Affiliations:** ^1^ Research School of Chemistry Australian National University Canberra ACT 2601 Australia

**Keywords:** Dendrimers, Metal Complexes, Multi-Photon Absorption, Nonlinear Optics, Organometallics

## Abstract

Multi‐photon absorption (MPA) has attracted interest for applications exploiting the tight spatial control of interaction volume and long wavelength excitation. However, a deficiency of molecules exhibiting higher‐order nPA (*n*‐photon absorption, *n*>2) and a lack of structure–property studies to define the key structural characteristics needed to optimize higher‐order MPA performance have hindered practical development. We herein report the syntheses of second‐ and third‐generation metallodendrimers and assessment of their nonlinear absorption, together with those of zero‐ and first‐generation analogues. We report the first 5PA and 6PA data for an organometallic. The largest dendrimer exhibits exceptional three‐, four‐, five‐ and six‐photon absorption to femtosecond‐pulsed light. The systematically varied compounds highlight the crucial role of metal‐to‐oligo(phenyleneethynylene) charge transfer in promoting outstanding MPA activity.

## Graphical abstract

Metallodendrimers comprising bis(diphosphine)ruthenium units in a phenyleneethynylene‐derived organic π‐framework are shown to display outstanding instantaneous multi‐photon absorption. The key structural requirements for the rare five‐ and six‐photon absorption seen with these compounds have been identified.

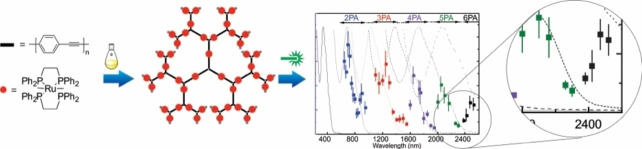

Instantaneous two‐photon absorption (2PA) can occur at the focal point of a laser beam, where the light is sufficiently intense. The resultant localization of “action” enables exquisite 3D control in applications that can exploit 2PA, such as micromachining, data storage, biological imaging, or photodynamic therapy; the development of efficient two‐photon absorbers has consequently been of great interest.^[1]^ Higher‐order multi‐photon absorption (MPA, *n*>2) may be more advantageous than 2PA, because the higher‐order light intensity dependence permits even greater spatial control, and because the excitation is shifted to longer wavelengths that may have technological utility (e.g. “near‐infrared II”, NIR‐II, 1000–1350 nm, and NIR‐III, 1550–1870 nm, at which biological transparency is maximized, and the “telecom” wavelengths, 1260–1675 nm, at which silica transparency is maximized). Studies of the MPA efficiency of diverse materials have therefore been of exceptional interest (Table S3, Supporting Information).[^2^, ^3^, ^4^, ^5^, ^6^, ^7^, ^8^] Thus far, the molecular structure‐property studies needed to elucidate efficient higher‐order MPA design criteria are largely restricted to 3PA,[^9^, ^10^, ^11^, ^12^, ^13^, ^14^] for which extent of π‐system is a broad guideline for effective materials; the dearth of structure‐property studies for higher‐order effects has stymied the development of efficient MPA materials. We herein report i) the design, synthesis, and characterization of new electron‐rich π‐delocalizable metallodendrimers, ii) studies examining their MPA performance towards femtosecond light pulses over a spectral range spanning the NIR and the aforementioned key technological “windows”, iii) the first 5PA and 6PA data for an organometallic, iv) an analysis of the experimental MPA data as a function of molecular composition, and development of structure‐property correlations for this class of molecule, v) the discovery of exceptional higher‐order MPA coefficients, and vi) the identification of metal‐to‐oligo(phenyleneethynylene) charge transfer as a key indicator of outstanding MPA merit.

Figure 1 depicts the systematically varied zero‐ to third‐generation metallodendrimers, which contain octahedrally‐ligated bis{bis(diphenylphosphino)ethane}ruthenium modules with *trans*‐disposed alkynyl ligands, a motif that affords an electron‐rich highly‐polarizable environment conducive to the formation of molecules with the potential to possess significant optical nonlinearities.[^15^, ^16^] The incorporation of bis(diphosphine)ruthenium units into an organic π‐framework not only significantly enhances electron‐richness, but also affords more soluble molecules than purely organic analogues.^[17]^ Synthetic details and ^1^H, ^13^C, and ^31^P NMR spectra confirming the identity of the new dendrimers and their precursors are collected in the Supporting Information (Figures S1–S57). Diffusion‐ordered NMR spectroscopy (Figures S58–S62), molecular modelling and transmission electron microscopy (Figure S64) and size‐exclusion chromatography (Figure S65) confirmed the size and mono‐disperse nature of the dendrimers, while electrospray ionization time‐of‐flight mass spectrometry proved useful for confirming the identity of the chlorido‐terminated dendrimer synthesis intermediates, ionization with loss of chloride and gain of solvent acetonitrile being a facile process (Figure S63). UV/Vis‐NIR spectra of the dendrimers are similar in appearance (Figure S66); the low‐energy MLCT bands centered at 410–430 nm correspond to charge transfer from the electron‐rich metal centers to the π‐extended arylalkynyl framework (Ru→(C_2_‐1,4‐C_6_H_4_)_*n*_C_2_Ar, *n*=1‐3), and the higher‐energy and more intense bands centered at 340–350 nm correspond to admixtures of charge transfer from ruthenium to peripheral phenylalkynyl ligands (Ru→C_2_Ph) and to proximal aryl branching points (Ru→C_2_C_6_H_3_), and intra‐ and inter‐phosphine/alkynyl ligand transitions, assigned from TD‐DFT calculations undertaken on the monometallic complex analogue *trans*‐[Ru(C≡C‐1,4‐C_6_H_4_C≡CPh)(C≡CPh)(κ^2^‐dppe)_2_].^[18]^

**Figure 1:**
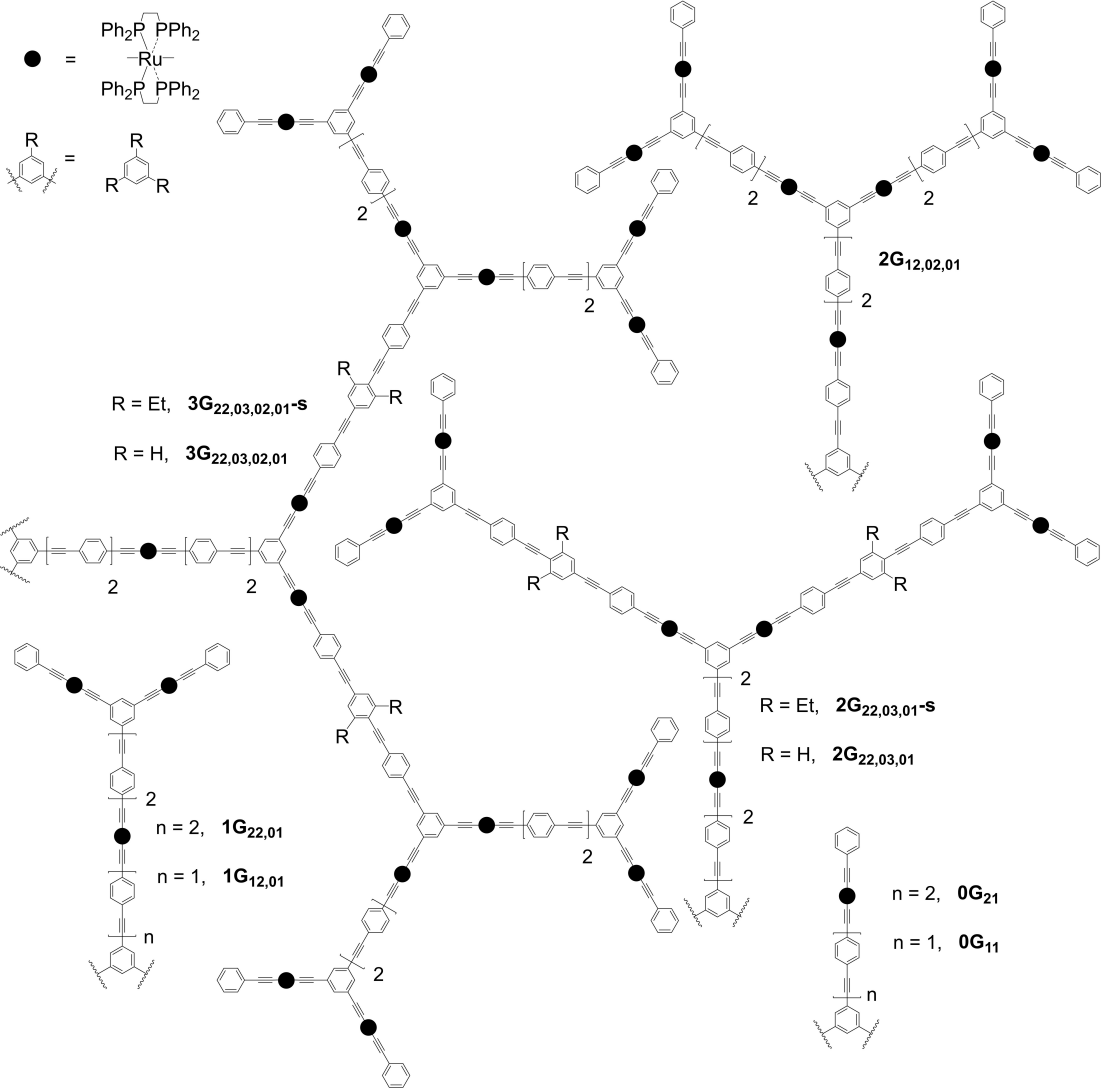
Dendrimers in this study. Black circles: *trans*‐[Ru(κ^2^‐dppe)_2_] (dppe=1,2‐bis(diphenylphosphino)ethane). Wavy lines: equivalent dendrons at dendrimer cores. Names follow the format: i) **nG** (*n*‐th generation dendrimer), ii) _**xy**_ (x and y phenyleneethynylene units between the branching points and the ligated Ru at each generation level, commencing at the core), iii) a comma separating the “**xy**” numbers of OPE units at each generation level, and in some cases iv) **‐s** (installation of solubilizing Et groups at the central phenylene of the OPE unit between the first‐ and second‐generation branching points).

The nonlinear absorption properties of the dendrimers were measured using the open‐aperture Z‐scan technique (600–2520 nm, ca. 130 femtosecond pulses); the nonlinear refractive behavior was measured simultaneously using closed‐aperture Z‐scan, revealing negative or zero γ_real_ values across the spectral range (Figures S67–S73). The absorptive nonlinearities replotted as the multi‐photon absorption cross‐sections are presented in Figure 2 and Figures S67–S73, accompanied by the linear optical absorption spectra plotted at twice to six‐times the wavelength to provide an eye‐guide. All new dendrimers show nonlinear absorption maxima at ca. 550–750 nm, 810–900 nm, 1200–1250 nm, and 1650 nm, confirmed to be 2PA, 2PA, 3PA, and 4PA in nature, respectively, from the intensity dependencies of the corresponding open‐aperture fs Z‐scan traces (see Figures S74–S77 for those of **3G_22,03,02,01_‐s**). The higher‐generation dendrimers also show MPA behavior with maximal values centered at 2050–2160 nm, confirmed to be the first reported 5PA data for organometallics from the intensity dependencies (Figure S78: **3G_22,03,02,01_‐s**). In addition, the lower‐generation dendrimers show weak maxima around 2000 nm for which the Z‐scan intensity dependencies are ambiguous (4PA or 5PA), while the higher‐generation dendrimers show weak maxima around 2400–2500 nm that their intensity dependencies (Figures S79–S80: **3G_22,03,02,01_‐s**) suggest are 5PA or, more likely, 6PA in nature. It is particularly noteworthy that the wavelengths of the maximal values of the intensity dependence‐assigned MPA correspond closely to the appropriate multiples of the wavelength of the low‐energy MLCT band, rather than multiples of the higher‐energy, more intense, MLCT/ILCT/LLCT admixtures; in combination with the intensity dependence, this strongly suggests that the longest wavelength higher‐generation dendrimer MPA data are indeed 6PA in nature (i.e. **3G_22,03,02,01_‐s**: σ_6_=53×10^−170^ cm^12^ s^5^ photon^−5^ @2470 nm), the first 6PA data for an organometallic, although we emphasize the need for caution. Correlation with the lower‐intensity, lower‐energy MLCT, rather than higher‐intensity, higher‐energy MLCT/ILCT/LLCT admixtures, emphasizes the importance of ligated metal modules and certain charge transfer in the MPA performance.

**Figure 2:**
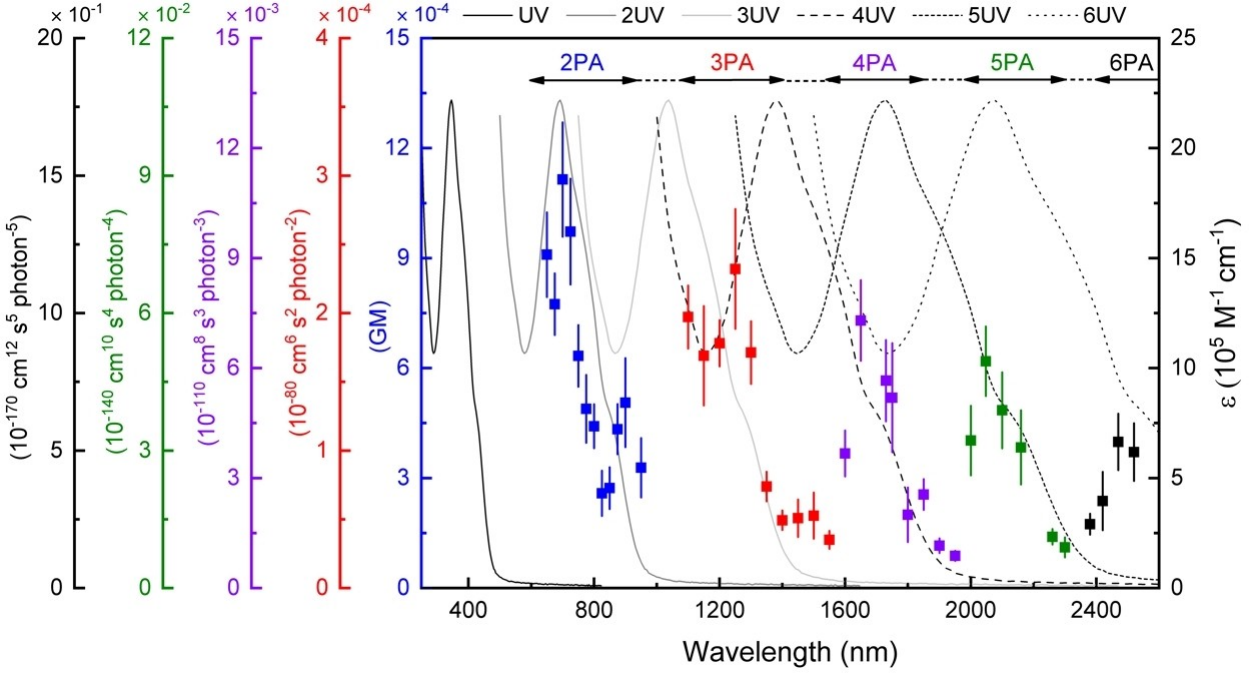
Wavelength dependence of the nonlinear absorption of **3G_22,03,02,01_‐s**. Plots of σ_2_ (blue), σ_3_ (red), σ_4_ (purple), σ_5_ (green), and σ_6_ (black) overlaid on the UV/Vis spectrum (black), and including plots of the UV/Vis spectrum as a function of twice (dark grey), three times (light grey), four times (long dashes), five times (medium‐sized dashes), and six times (short dashes) the wavelength.

Linear and nonlinear absorption data are collected in Table 1, together with reported data for the zero‐generation analogues.[^11^, ^19^] For clarity, the data for dendrimers with mono(phenyleneethynylene) (1PE) units linking the zero‐generation‐level Ru atoms to the core (**0G_11_**, **1G_12,01_**, **2G_12,02,01_**) have been separated from those for the analogous dendrimers with a 2PE linkage by a dotted line. Across the suite of compounds, increasing the length of the π‐linkage attached to the core (proceeding from “1PE‐core” to “2PE‐core” analogues) generally leads to an increase in linear and nonlinear absorption parameters. Introduction of solubilizing alkyl substituents in the “2PE‐core” group (proceeding from **2G_22,03,01_** to **2G_22,03,01_‐s** or from **3G_22,03,02,01_** to **3G_22,03,02,01_‐s**) has a more subtle impact on MPA performance (σ_3_‐σ_5_ values improve slightly). Significant increases in molar extinction coefficients and 2PA to 5PA cross‐sections are seen with both the “1PE‐core” and “2PE‐core” groups on increase in dendrimer generation. The increases in maximal MPA values that are seen on increasing dendrimer generation warranted a closer inspection, so additional data comparisons were undertaken scaling maximal values by both molecular weight, the most common literature approach to compare molecules, and also by the appropriate power of the “number of effective electrons”, a rigorous comparison that considers the most polarizable electrons[^20^, ^21^, ^22^] (Scheme S1, Tables 1 and S2). Interestingly, the *σ*_2_/M and *σ_2_*/N_eff_^2^ values corresponding to the maxima at wavelengths roughly twice those of the higher‐energy MLCT/ILCT/LLCT admixtures increase substantially proceeding from zero‐ to third‐generation dendrimers, i.e. these data reveal a 2PA dendritic effect, while the *σ_n_*/M and *σ_n_*/N_eff_^2^ values at wavelengths corresponding to multiples of the lower‐energy MLCT band do not display a superlinear increase with increasing generation.

**Table 1:** Linear optical and NLO absorption cross‐section maxima, and MWt‐ and number of “effective” electrons‐scaled short‐wavelength *σ*_2_ data.^[a]^

Complex	*λ*_1,max_^[b]^ [ϵ]^[c]^	*λ*_2,max_^[b]^ [ϵ]^[c]^	*σ*_2_^[d]^ (*λ*_max_)^[b]^	*σ*_2_/M^[e]^, *σ_2_*/N_eff_^2[d,f]^ (*λ*_max_)^[b]^	*σ*_3_^[g]^ (*λ*_max_)^[b]^	*σ*_4_^[h]^ (*λ*_max_)^[b]^	*σ*_5_^[i]^ (*λ*_max_)^[b]^
**3G_22,03,02,01_‐s**	346 [222]	424 [81]	111 500 (700), 50 600 (900)	2.15, 7.97 (700)	23 200 (1250)	7300 (1650)	500 (2050)
**3G_22,03,02,01_**	340 [206]	418 [106]	113 200 (725), 45 900 (900)	2.20, 8.09 (725)	22 200 (1200)	5850 (1650)	350 (2100)
**2G_22,03,01_‐s**	343 [119]	421 [52]	42 450 (750), 16 000 (875)	1.71, 5.21 (750)	15 000 (1250)	3700 (1650)	170 (2100)
**2G_22,03,01_**	343 [118]	415 [49]	43 600 (700), 20 000 (900)	1.78, 5.35 (700)	13 750 (1250)	2950 (1650)	100 (2160)
**1G_22,01_**	340 [43]	427 [19]	13 700 (725), 8800 (900)	1.31, 4.77 (725)	4800 (1200)	1200 (1650)	^[j]^
**0G_21_** ^[k]^	^[l]^	422 [12.6]	3200 (900)		2300 (1200)	^[j]^	^[j]^
**2G_12,02,01_**	340 [96]	409 [46]	34 700 (750), 18 000 (900)	1.47, 6.83 (750)	7950 (1250)	2700 (1650)	^[j]^
**1G_12,01_**	343 [38]	412 [18]	14 000 (725), 6300 (875)	1.38, 6.64 (725)	2500 (1250)	900 (1650)	^[j]^
**0G_11_** ^[k,m]^	^[l]^	412 [11.6]	1500 (650), 370 (810)	0.43, 2.40 (650)	100 (1240)	^[j]^	^[j]^

[a] Solvent CH_2_Cl_2_. [b] nm. [c] 10^4^ L mol^−1^ cm^−1^. [d] GM=10^−50^ cm^4^ s photon^−1^. [e] GM mol g^−1^. [f] N_eff_=118.3 (**3G_22,03,02,01_‐s**, **3G_22,03,02,01_**), 90.3 (**2G_22,03,01_‐s**, **2G_22,03,01_**), 53.6 (**1G_22,01_**), 37.3 (**0G_21_**), 71.3 (**2G_12,02,01_**), 46.0 (**1G_12,01_**), 25.0 (**0G_11_**). [g] 10^−80^ cm^6^ s^2^ photon^−2^. [h] 10^−110^ cm^8^ s^3^ photon^−3^. [i] 10^−140^ cm^10^ s^4^ photon^−4^. [j] No measurable activity. [k] Ref. [11]. [l] Not reported. [m] Ref. [19].

Molecular MPA cross‐sections for the third‐generation dendrimers are exceptionally large: i) 3PA data are only exceeded by certain ladder‐type oligo(*p*‐phenylene)s (LOPPs)[^10^, ^23^] for which π‐system planarity is enforced, ii) 4PA data are the largest thus far for organic or organometallic molecules, iii) 5PA data are only the third and fourth molecular cross‐sections reported,[^10^, ^24^] and iv) the 6PA datum is only the second reported for a molecule, following that of a spiro‐fused LOPP.^[10]^ We note that, compared to the present dendrimers, the LOPPs exhibit 4PA, 5PA, and 6PA at much shorter wavelengths, viz. 1440, 1540, and 1820 nm, respectively; as well as identifying a new class of compound exhibiting higher‐order MPA, the present study therefore expands the wavelength region in which higher‐order molecular MPA has been observed.

It has previously been shown that related “all‐organic” π‐delocalizable dendrimers exhibit 2PA but show no measurable MPA.^[17]^ The present suite of complexes, with ligated metals embedded in an organic framework, display analogous intra‐(phenyleneethynylene) transitions to those of the organic dendrimers at high‐energy (as admixtures with LLCT and Ru→C_2_Ph/C_2_C_6_H_3_ MLCT: see above), but in addition show low‐energy Ru→(C_2_‐1,4‐C_6_H_4_)_*n*_C_2_Ar (*n*=1‐3). The high‐energy bands are associated with 2PA at double the wavelength, but no corresponding higher‐order MPA is seen, a similar observation to the organic dendrimers. In contrast, the low‐energy bands have associated MPA behavior (up to 6PA). The 1,3,5‐trisubstituted aryl groups disrupt the formal conjugation, and so electron density is delocalized from the metal centers to oligo(phenyleneethynylene) (OPE) units of 1PE to 4PE in length (Figure 1). It is the 2PE to 4PE segments attached to the metal that are the key distinguishing features promoting the outstanding MPA behavior.

In conclusion, the present study has revealed that appropriately designed metal‐containing organic dendrimers can display exceptional higher‐order MPA behavior. The studies have also disclosed a 2PA dendritic effect, and have afforded the first 5PA and 6PA data for an organometallic, and only the second such 6PA data for any molecule. The crucial molecular‐composition requirement for promoting outstanding MPA activity is seen to be the presence of 2PE or longer OPE units directly attached to the metal. Previous observations with LOPPs have highlighted the importance of extended π‐framework co‐planarity in achieving exceptional MPA‐activity; the present study complements those observations, highlighting the key role that ligated metals and certain charge transfer can play in affording efficient higher‐order MPA materials.

## Conflict of interest

The authors declare no conflict of interest.

## Supporting information

As a service to our authors and readers, this journal provides supporting information supplied by the authors. Such materials are peer reviewed and may be re‐organized for online delivery, but are not copy‐edited or typeset. Technical support issues arising from supporting information (other than missing files) should be addressed to the authors.

Supporting Information

## Data Availability

The data that support the findings of this study are available in the supplementary material of this article.
