# Supplementary material for: Outstanding Multi‐Photon Absorption at π‐Delocalizable Metallodendrimers
Source: Angew Chem Int Ed Engl. 2022 Jan 20;61(10):e202116181. doi: 10.1002/anie.202116181 (PMC9306932; doi:10.1002/anie.202116181)
Supplement: Supplementary file 1 — Supporting Information [file ANIE-61-0-s001.pdf]

Angewandte Chemie
Eine Zeitschrift der Gesellschaft Deutscher Chemiker

Supporting Information

Outstanding Multi-Photon Absorption at π-Delocalizable Metallodendrimers

*L. Zhang, M. Morshedi, M. G. Humphrey**

Author Contributions

L.Z. Data curation:Lead; Formal analysis:Lead; Investigation:Supporting; Validation:Supporting; Writing – original draft:Supporting
M.M. Data curation:Supporting; Formal analysis:Supporting; Investigation:Supporting; Supervision:Supporting; Validation:Supporting; Writing – original draft:Supporting
M.H. Conceptualization:Lead; Data curation:Supporting; Formal analysis:Supporting; Investigation:Supporting; Project administration:Lead; Resources:Lead; Supervision:Lead; Validation:Lead; Writing – original draft:Lead; Writing – review & editing:Lead

Contents

General Conditions and Reagents……………………………………………………………S3

Synthesis of Organic Compounds…………………………………………..……………..S4

Synthesis of Metal Complexes…………………………...………………………………...S7

Instrumentation………………………………………………………………………......S25

Calculation of the “Number of Effective π-Electrons”………………………………………S27

NMR Spectra of New Compounds………………………………………………………S31

DOSY 2D NMR Spectra of Ruthenium Dendrimers………………………………………S83

MS, TEM and Size-exclusion Studies of Ruthenium Dendrimers………………………....S86

UV-Vis and Z-scan Studies of Ruthenium Dendrimers……………………………………S88

Three-photon, Four-photon, Five-photon, and Six-photon Absorption Data for Coordination Complexes, Organic Molecules, Polymers, MOFs, Perovskites and Carbon-based Materials (for data pre-2016, see ref [13])…………………………………………………………...S102

References…………………………………………………………………………………S122

General Conditions and Reagents

Reactions were performed under a nitrogen atmosphere using standard Schlenk techniques, with no precautions to exclude air during workup. The following solvents, starting materials and catalysts were commercially available, purchased from common chemical suppliers and used as received (unless otherwise specifically mentioned): 1,3,5-tribromobenzene, trimethylsilylacetylene (TMSA), (triisopropylsilyl)acetylene (TIPSA), tetra-*n*-butylammonium fluoride (TBAF, as a 1.0 M solution in THF), $PdCl_2(PPh_3)_2$, CuI, I_2, ethynylbenzene, *n*-BuLi (2.5 M in *n*-hexane), $NaPF_6$, K_2CO_3, NEt_3, NH_4Cl, $MgSO_4$, *n*-pentane, petrol, methanol (MeOH), diethyl ether (Et_2O), Celite. The starting material *trans*-$[RuCl_2(dppe)_2]$ was synthesised following standard procedures.[1] The term "petrol" refers to a fraction of petroleum with a boiling range of 60-80 °C. Reagent grade solvent dichloromethane (CH_2Cl_2) (Merck) was dried by distilling over calcium hydride and stored under nitrogen, and tetrahydrofuran (THF) (Merck) over sodium/benzophenone. Column chromatography was performed using either alumina or silica (230-400 mesh).

Commercially available $Pd(PPh_3)_4$ was recrystallized from deoxygenated ethanol and stored under a nitrogen atmosphere before use. Commercial $[Cu(NCMe)_4]PF_6$ was recrystallized from hot acetonitrile prior to use and kept under an inert atmosphere avoiding light. The purification followed the standard procedure.[2]

The following materials were synthesised based on reported procedures or slight modifications thereof:

1-Bromo-4-ethynylbenzene [3]

{(4-Iodophenyl)ethynyl}triisopropylsilane [4]

1-Ethynyl-4-{(4-iodophenyl)ethynyl}benzene [4]

((3,5-Diethyl-4-iodophenyl)ethynyl)trimethylsilane [5]

1,4-(*i*-Pr)$_3$SiC≡CC$_6$H$_4$C≡C-1,4-C$_6$H$_4$I [6]

HC≡C-1,4-C$_6$H$_4$C≡C-1,4-C$_6$H$_4$I [4]

1,3-{*trans*-[(dppe)$_2$ClRu(C≡C)]}$_2$-5-HC≡CC$_6$H$_3$ [7]

1,3-{*trans*-[(dppe)$_2$(PhC≡C)Ru(C≡C)]}$_2$-5-HC≡CC$_6$H$_3$ (**10**) [7]

1,3-{*trans*-[(dppe)$_2$(PhC≡C)Ru(C≡C)]}$_2$-5-(*i*-Pr$_3$SiC≡C-1,4-C$_6$H$_4$C≡C-1,4-C$_6$H$_4$C≡C)C$_6$H$_3$ (**12**) [8]

1,3-{*trans*-[(dppe)$_2$(PhC≡C)Ru(C≡C)]}$_2$-5-(HC≡C-1,4-C$_6$H$_4$C≡C-1,4-C$_6$H$_4$C≡C)C$_6$H$_3$ (**13**) [8]

1,3,5-{*trans*-[(1,4-IC$_6$H$_4$C≡C-1,4-C$_6$H$_4$C≡C)Ru(dppe)$_2$(C≡C-1,4-C$_6$H$_4$C≡C-1,4-C$_6$H$_4$C≡C)]}$_3$C$_6$H$_3$ (**31**) [8]

1,3,5-{*trans*-[{3,5-{*trans*-[(PhC≡C)Ru(dppe)$_2$(C≡C)]}$_2$C$_6$H$_3$-1-(C≡C-1,4-C$_6$H$_4$C≡C-1,4-C$_6$H$_4$C≡C)}Ru(dppe)$_2${C≡C-1,4-C$_6$H$_4$C≡C-1,4-C$_6$H$_4$C≡C}]}$_3$C$_6$H$_3$ (**1G$_{22,01}$**) [8]

1,3,5-{*trans*-[{3,5-{*trans*-[(PhC≡C)Ru(dppe)$_2$(C≡C)]}$_2$C$_6$H$_3$-1-(C≡C-1,4-C$_6$H$_4$C≡C-1,4-C$_6$H$_4$C≡C)}Ru(dppe)$_2${C≡C-1,4-C$_6$H$_4$C≡C}]}$_3$C$_6$H$_3$ (**1G$_{12,01}$**) [4]

1,3,5-{*trans*-[{3,5-{*trans*-[(1,3-{*trans*-[(dppe)$_2$(PhC≡C)RuC≡C]}$_2$-5-C≡CC$_6$H$_3$-1,4-C≡CC$_6$H$_4$-1,4-C$_6$H$_4$C≡C)Ru(dppe)$_2$(C≡C)]}$_2$C$_6$H$_3$-1-(C≡C-1,4-C$_6$H$_4$C≡C-1,4-C$_6$H$_4$C≡C)}Ru(dppe)$_2$(C≡C-1,4-C$_6$H$_4$C≡C)]}$_3$C$_6$H$_3$ (**2G$_{12,02,01}$**) [4]

Synthesis of Organic Compounds

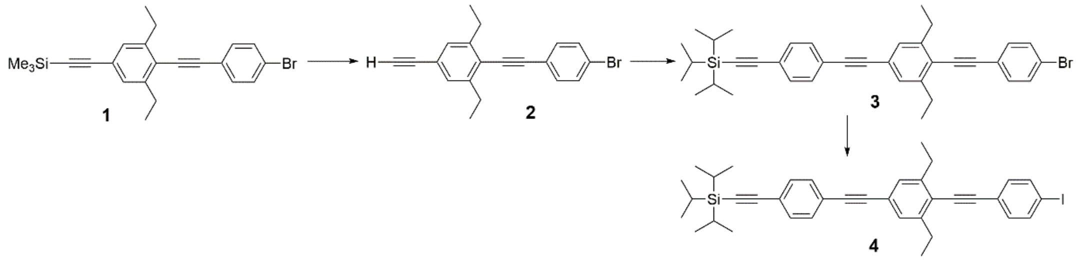

Synthesis of 1-(Me$_3$SiC≡C)-3,5-Et$_2$-C$_6$H$_2$-4-C≡C-1,4-C$_6$H$_4$Br (1). ((3,5-Diethyl-4-iodophenyl)ethynyl)trimethylsilane (3.00 g, 8.42 mmol) was dissolved in 100 mL deoxygenated NEt$_3$, and 1-bromo-4-ethynylbenzene (2.21 g, 12.21 mmol, 1.4 eq.) was added to the solution. The reaction was cooled to 0 °C and Pd(PPh$_3$)$_4$ (0.29 g, 0.25 mmol, 0.03 eq.) and CuI (0.048 g, 0.25 mmol, 0.03 eq.) were added in catalytic amounts. The reaction was stirred at 0 °C for 3 h and then slowly warmed to room temperature to react overnight. Saturated NH$_4$Cl solution was added to quench the reaction. The crude product was extracted by CH$_2$Cl$_2$ (3 × 50 mL), and the solution was dried over MgSO$_4$. Further purification was conducted by column chromatography on silica, eluting with petrol to afford **1** as a white powder (2.87 g, 7.00 mmol, 83%).

^{1}H NMR (400 MHz, CDCl$_3$): δ 7.49 (d, J = 8.4 Hz, 2H, H$_2$), 7.37 (d, J = 8.4 Hz, 2H, H$_3$), 7.21 (s, 2H, H$_9$), 2.84 (q, J = 7.5 Hz, 4H, H$_{s1}$), 1.28 (t, J = 7.5 Hz, 6H, H$_{s2}$), 0.26 (s, 9H, H$_{p1}$) ppm. ^{13}C NMR (176 MHz, CDCl$_3$): δ 146.6 (C$_8$), 132.9 (C$_3$), 131.8 (C$_2$), 129.0 (C$_9$), 123.0 (C$_{10}$), 122.7 (C$_1$), 122.6 (C$_4$), 121.8 (C$_7$), 105.5 (C$_{11}$), 97.4 (C$_5$), 95.4 (C$_{12}$), 87.7 (C$_6$), 28.0 (C$_{s1}$), 14.7 (C$_{s2}$), 0.1 (C$_{p1}$) ppm. IR: ν(C≡C) 2150 cm^{-1}. UV-Vis (CH$_2$Cl$_2$, ν_{max} in cm^{-1}, [ε] in 10^3 M^{-1} cm^{-1}): 29 850 [42.3], 31 850 [47.5]. EI MS *m/z* (%): 409 ([M-H]$^+$, 100). HR EI MS: Calcd. for C$_{23}$H$_{25}$BrSi: 410.0885. Found: 410.0888. Anal. Calcd. for C$_{23}$H$_{25}$BrSi: C, 67.47; H, 6.15%. Found: C, 67.46; H, 6.18%.

Synthesis of 1-(HC≡C)-3,5-Et$_2$-C$_6$H$_2$-4-C≡C-1,4-C$_6$H$_4$Br (2). Compound **1** (2.68 g, 6.55 mmol) was dissolved in a solution of MeOH/CH_2Cl_2 (1:1, 60 mL) and K_2CO_3 (1.00 g, 7.23 mmol, 1.1 eq.) was added. The reaction was stirred at room temperature for 1 h. The crude product was obtained by extraction with water/CH_2Cl_2 (1:1, 3 × 50 mL), and then passed through a Celite pad, removing the solvent *in vacuo*. Further purification was conducted via column chromatography on silica (gradient elution with petrol/CH_2Cl_2 = 9:1) to afford a white powder identified as **2** (1.97 g, 5.84 mmol, 89%).

^{1}H NMR (400 MHz, $CDCl_3$): δ 7.50 (d, J = 8.4 Hz, 2H, H_2), 7.37 (d, J = 8.4 Hz, 2H, H_3), 7.23 (s, 2H, H_9), 3.13 (s, 1H, H_{12}), 2.85 (q, J = 7.5 Hz, 4H, H_{s1}), 1.28 (t, J = 7.5 Hz, 6H, H_{s2}) ppm. ^{13}C NMR (151 MHz, $CDCl_3$) δ 146.7 (C_8), 132.9 (C_3), 131.8 (C_2), 129.2 (C_9), 122.7 (C_4), 122.6 (C_1), 122.2 (C_7), 122.0 (C_{10}), 97.5 (C_5), 87.5 (C_6), 84.0 (C_{11}), 78.2 (C_{12}), 28.0 (C_{s1}), 14.7 (C_{s2}) ppm. EI MS *m/z* (%): 338 ([M]$^+$, 100). HR EI MS: Calcd. for $C_{20}H_{17}Br$: 336.0514. Found: 336.0514. IR: ν(C≡C) 2106 cm^{-1}. Anal. Calcd. for $C_{20}H_{17}Br$: C, 71.23; H, 5.08%; Found: C, 71.12; H, 5.07%.

Synthesis of 1,4-{(i-Pr)$_3$SiC≡C}C$_6$H$_4$C≡C-3,5-Et$_2$-1,4-C$_6$H$_2$C≡C-1,4-C$_6$H$_4$Br (3). Compound **2** (1.85 g, 5.50 mmol) was dissolved in 100 mL deoxygenated NEt_3, and {(4-iodophenyl)ethynyl}triisopropylsilane (2.87 g, 7.20 mmol, 1.3 eq.) was added to the solution. Pd(PPh_3)$_4$ (0.184 g, 0.16 mmol, 0.03 eq.) and CuI (0.011 g, 0.06 mmol, 0.01 eq.) were added to the solution. The reaction mixture was stirred at 0 °C for 3 h and then slowly warmed to room temperature overnight. Saturated NH_4Cl solution (40 mL) was added to quench the reaction. The crude product was extracted by CH_2Cl_2 (3 × 40 mL), and the solution was dried over $MgSO_4$. Further purification was conducted by column chromatography on silica, eluting with petrol to afford a white powder identified as **3** (2.37 g, 3.99 mmol, 73%).

^{1}H NMR (400 MHz, $CDCl_3$): δ 7.50 (d, J = 8.3 Hz, 2H, H_2), 7.46 (s, 4H, H_{14}, H_{15}), 7.39 (d, J = 8.3 Hz, 2H, H_3), 7.27 (s, 2H, H_9), 2.88 (q, J = 7.5 Hz, 4H, H_{s1}), 1.31 (t, J = 7.5 Hz, 6H, H_{s2}), 1.14 (s, 21H, H_{p1}, H_{p2}) ppm. ^{13}C NMR (176 MHz, $CDCl_3$) δ 146.7 (C_8), 137.8 (C_{14} or C_{15}), 133.0 (C_3), 132.1 (C_{14} or C_{15}), 131.5 (C_2), 128.7 (C_9), 123.5 (C_{13} or C_{16}), 123.2-123.2 (C_1 or C_4)), 122.9 (C_{10}), 121.8 (C_7), 106.8 (C_{12} or C_{17}), 97.7 (C_5), 94.2 (C_{18}), 93.0 (C_{13} or C_{16}), 91.7 (C_{11}), 90.4 (C_{12} or C_{17}), 88.0 (C_6), 28.1 (C_{s1}), 18.8 (C_{p2}), 14.7 (C_{s2}), 11.5 (C_{p1}) ppm. IR: ν(C≡C) 2156 cm^{-1}. UV-Vis (CH_2Cl_2, ν_{max} in cm^{-1}, [ε] in 10^3 M^{-1} cm^{-1}): 27 600 [33.3, sh], 29 500 [48.0]. HR ESI TOF MS: Calcd. for $C_{37}H_{41}BrSi$: 593.2235. Found: 593.2239. Anal. Calcd. for $C_{37}H_{41}BrSi$: C, 74.85; H, 6.96%. Found: C, 74.75; H, 6.89%.

Synthesis of 1,4-{(i-Pr)$_3$SiC≡C}C$_6$H$_4$C≡C-3,5-Et$_2$-1,4-C$_6$H$_2$C≡C-1,4-C$_6$H$_4$I (4). Compound **3** (1.37 g, 2.31 mmol) was dissolved in distilled THF (40 mL) and the solution was cooled to -78 °C and stirred for 30 min. *n*-BuLi (2.5 M in *n*-hexane, 1.0 mL, 1.1 eq.) was slowly added to the reaction via syringe. The mixture was stirred for 2 h at -78 °C and then ground iodine powder (0.79 g, 3.12 mmol, 1.5 eq.) was slowly added. After warming the reaction to room temperature, saturated $Na_2S_2O_3$ solution (10 mL) was added to the reaction mixture. The

product was extracted by CH_2Cl_2 (3 × 50 mL) and then the solution was dried over $MgSO_4$. Further purification was conducted by recrystallisation from CH_2Cl_2/MeOH (1:10) to afford **4** as a white solid (1.20 g, 1.87 mmol, 81%).

^{1}H NMR (400 MHz, $CDCl_3$): δ 7.71 (d, J = 8.1 Hz, 2H, H_2), 7.46 (s, 4H, H_{14}, H_{15}), 7.27 (s, 2H, H_9), 7.25 (d, J = 8.1 Hz, 2H, H_3), 2.87 (q, J = 7.5 Hz, 4H, H_{s1}), 1.30 (t, J= 7.5 Hz, 6H, H_{s2}), 1.14 (s, 21H, H_{p1}, H_{p2}) ppm. ^{13}C NMR (176 MHz, $CDCl_3$) δ 146.7 (C_8), 137.8 (C_2), 133.0 (C_3), 132.1 (C_{14} or C_{15}), 131.5 (C_{14} or C_{15}), 128.7 (C_9), 123.5 (C_{13} or C_{16}), 123.2 (C_{10}), 123.2 (C_4), 122.9 (C_{13} or C_{16}), 121.8 (C_7), 106.8 (C_{12} or C_{17}), 97.7 (C_1), 94.2 (C_5), 93.0 (C_{18}), 91.7 (C_{11}), 90.4 (C_{12} or C_{17}), 88.0 (C_6), 28.1 (C_{s1}), 18.8 (C_{p2}), 14.7 (C_{s2}), 11.5 (C_{p1}) ppm. IR: ν(C≡C) 2154 cm^{-1}. UV-Vis (CH_2Cl_2, ν_{max} in cm^{-1}, [ε] in 10^3 M^{-1} cm^{-1}): 27 450 [50.7, sh], 29 300 [72.4]. EI MS *m/z* (%): 640 ([M]$^+$, 85). HR EI MS: Calcd. for $C_{37}H_{41}ISi$: 640.2022. Found: 640.2025. Anal. Calcd. for $C_{37}H_{41}ISi$: C, 69.36; H, 6.45%. Found: C, 69.42; H, 6.51%.

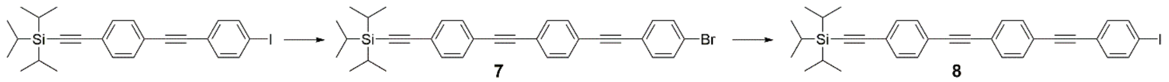

Synthesis of 1,4-{(i-Pr)$_3$SiC≡C}C_6H_4C≡C-1,4-C_6H_4C≡C-1,4-C_6H_4Br (7). 1,4-{(*i*-Pr)$_3$SiC≡C}C_6H_4C≡C-1,4-C_6H_4I (1.21 g, 2.50 mmol) was dissolved in 50 mL deoxygenated NEt_3, and 1-bromo-4-ethynylbenzene (0.50 g, 2.75 mmol, 1.1 eq.) was added to the reaction mixture. After cooling the mixture to 0 °C, Pd(PPh_3)$_4$ (0.09 g, 0.08 mmol, 0.03 eq.) and CuI (0.02 g, 0.08 mmol, 0.03 eq.) were added to the reaction mixture. The reaction was stirred at 0 °C for 3 h and then slowly warmed to room temperature overnight. Saturated NH_4Cl solution (30 mL) was added to quench the reaction. The crude product was extracted by CH_2Cl_2 (3 × 50 mL), and the solution was dried over $MgSO_4$. Further purification was conducted by column chromatography on silica (eluting with petrol), to afford **7** as a white powder. (0.982 g, 1.82 mmol, 73%).

^{1}H NMR (400 MHz, $CDCl_3$): δ 7.50-7.48 (m, 3H, H_8, H_9, H_2), 7.46 (s, 4H, H_{14}, H_{15}), 7.39 (d, J = 8.5 Hz, 2H, H_3), 1.13 (s, 21H, H_{p1}, H_{p2}) ppm. ^{13}C NMR (176 MHz, $CDCl_3$): δ 133.2 (C_3), 132.2 (C_{14} or C_{15}), 131.8 (C_2), 131.73-131.70 (C_8 or C_9), 131.5 (C_{14} or C_{15}), 123.8 (C_{13}), 123.3-123.1 (C_7 or C_{10}), 123.0 (C_{16}), 122.9 (C_4), 122.1 (C_1), 106.7 (C_{12}), 93.2 (C_{11}), 91.3 (C_{17}), 91.0 (C_6), 90.4 (C_5), 90.3 (C_{18}), 18.8 (C_{p2}), 11.5 (C_{p1}) ppm. IR: ν(C≡C) 2151 cm^{-1}. UV-Vis (CH_2Cl_2, ν_{max} in cm^{-1}, [ε] in 10^3 M^{-1} cm^{-1}): 27 850 [20.7, sh], 29 850 [30.3]. EI MS *m/z* (%): 538 ([M]$^+$, 75). HR EI MS: Calcd. for $C_{33}H_{33}BrSi$: 538.1509. Found: 538.1514. Anal. Calcd. for $C_{33}H_{33}BrSi$: C, 73.73; H, 6.19%. Found: C, 73.80; H, 6.34%.

Synthesis of 1,4-{(i-Pr)$_3$SiC≡C}C_6H_4C≡C-1,4-C_6H_4C≡C-1,4-C_6H_4I (8). Compound **7** (0.114 g, 0.21 mmol) was dissolved in distilled THF (20 mL) and the solution was cooled to -78 °C. *n*-BuLi (2.5 M in *n*-hexane, 0.1 mL, 1.2 eq.) was slowly added to the solution via syringe. The mixture was stirred at -78 °C for 2 h and then ground iodine powder (0.10 g, 0.39 mmol, 1.9

eq.) was slowly added. After warming the reaction to room temperature, saturated sodium thiosulfate solution (5 mL) was added to the reaction mixture. The product was extracted via CH_2Cl_2 (3 × 30 mL), and the solution was dried over $MgSO_4$. Further purification was conducted by crystallization from MeOH/CH_2Cl_2 solution to afford **8** as a white solid (0.086 g, 0.15 mmol, 71%).

^{1}H NMR (700 MHz, $CDCl_3$) δ 7.60 (d, J = 8.4 Hz, 2H, H_2), 7.41 (s, 4H, H_8, H_9), 7.37 (s, 4H, H_{14}, H_{15}), 7.16 (d, J = 8.4 Hz, 2H, H_3), 1.05 (s, 21H, H_{p1}, H_{p2}) ppm. ^{13}C NMR (176 MHz, $CDCl_3$) δ137.7 (C_2), 133.2 (C_3), 132.1 (C_{14}), 131.7 (C_9), 131.7 (C_8), 131.5 (C_{15}), 123.7 (C_{13}), 123.3 (C_{16}), 123.0 (C_{10}), 123.0 (C_7), 122.7 (C_4), 106.7 (C_1), 94.6 (C_5), 93.2 (C_{12}), 91.3 (C_{17}), 91.0 (C_{11}), 90.62 (C_6), 90.58 (C_{18}), 18.8 (C_{p2}), 11.5 (C_{p1}) ppm. IR: ν(C≡C) 2150 cm^{-1}. UV-Vis (CH_2Cl_2, ν_{max} in cm^{-1}, [ε] in 10^3 M^{-1} cm^{-1}): 27 750 [48.1, sh], 29 650 [72.6]. EI MS m/z (%): 584 ($[M]^+$, 70). HR EI MS: Calcd. for $C_{33}H_{33}ISi$: 584.1396. Found: 584.1396. Anal. Calcd. for $C_{33}H_{33}ISi$: C, 67.80; H, 5.69%. Found: C, 67.72; H, 5.75%.

Synthesis of Metal Complexes.

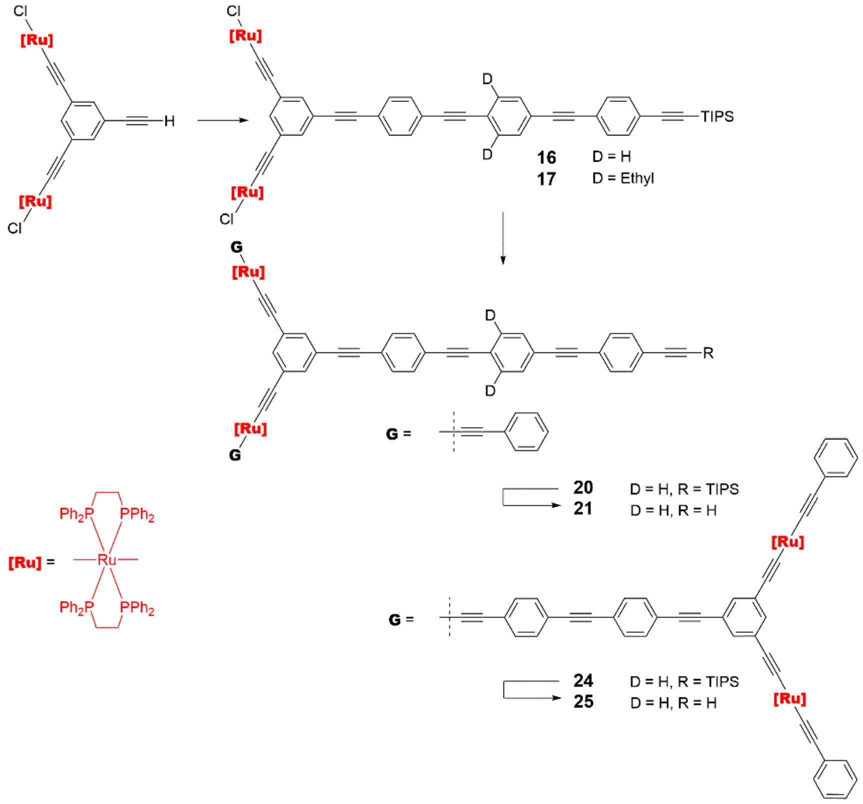

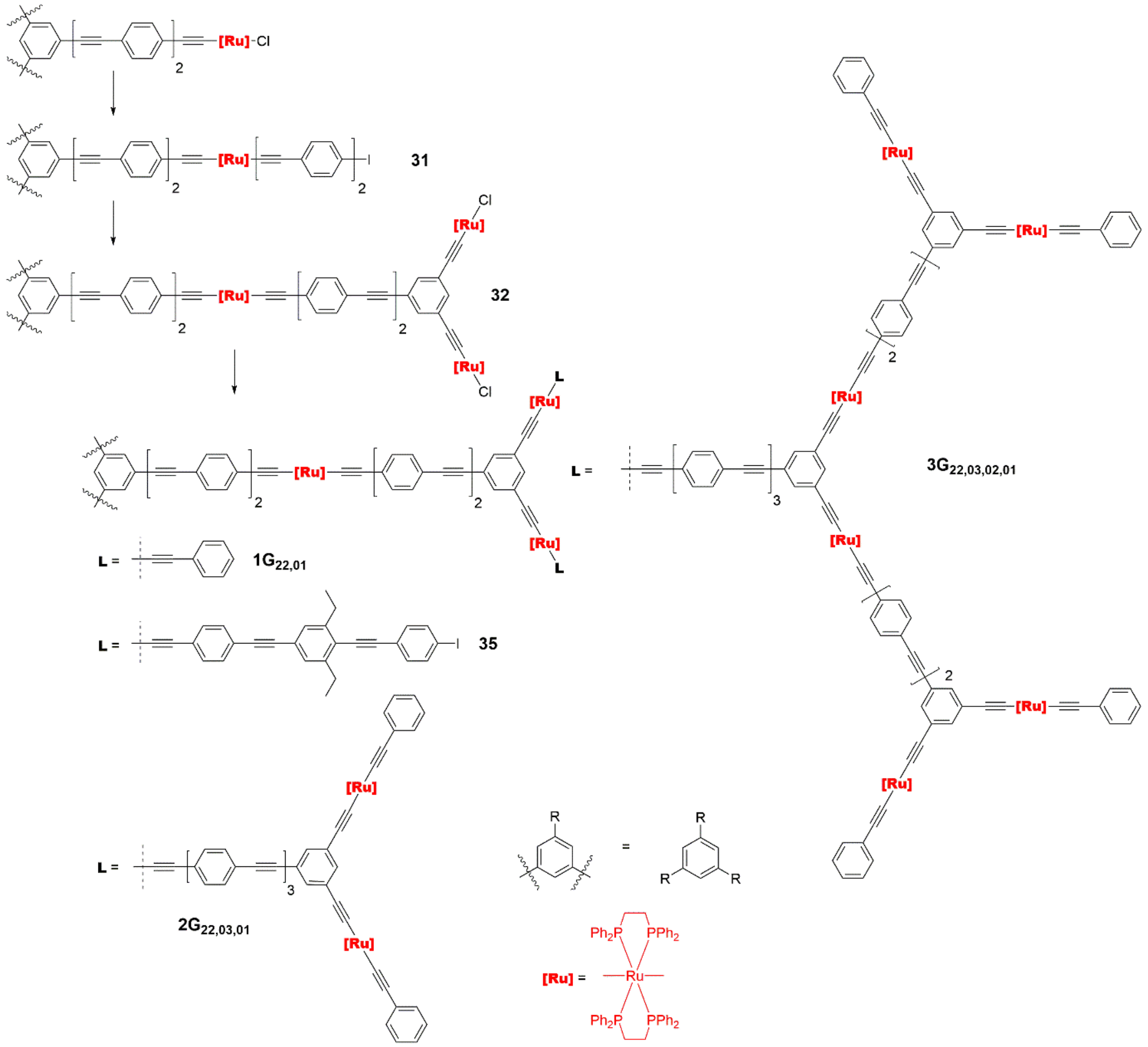
[Ru]
Cl
2
31
32
L
L =
1G22,01
35
3G22,03,02,01
2G22,03,01
3
R
=
Ph2P
PPh2
Ru
[Ru] =

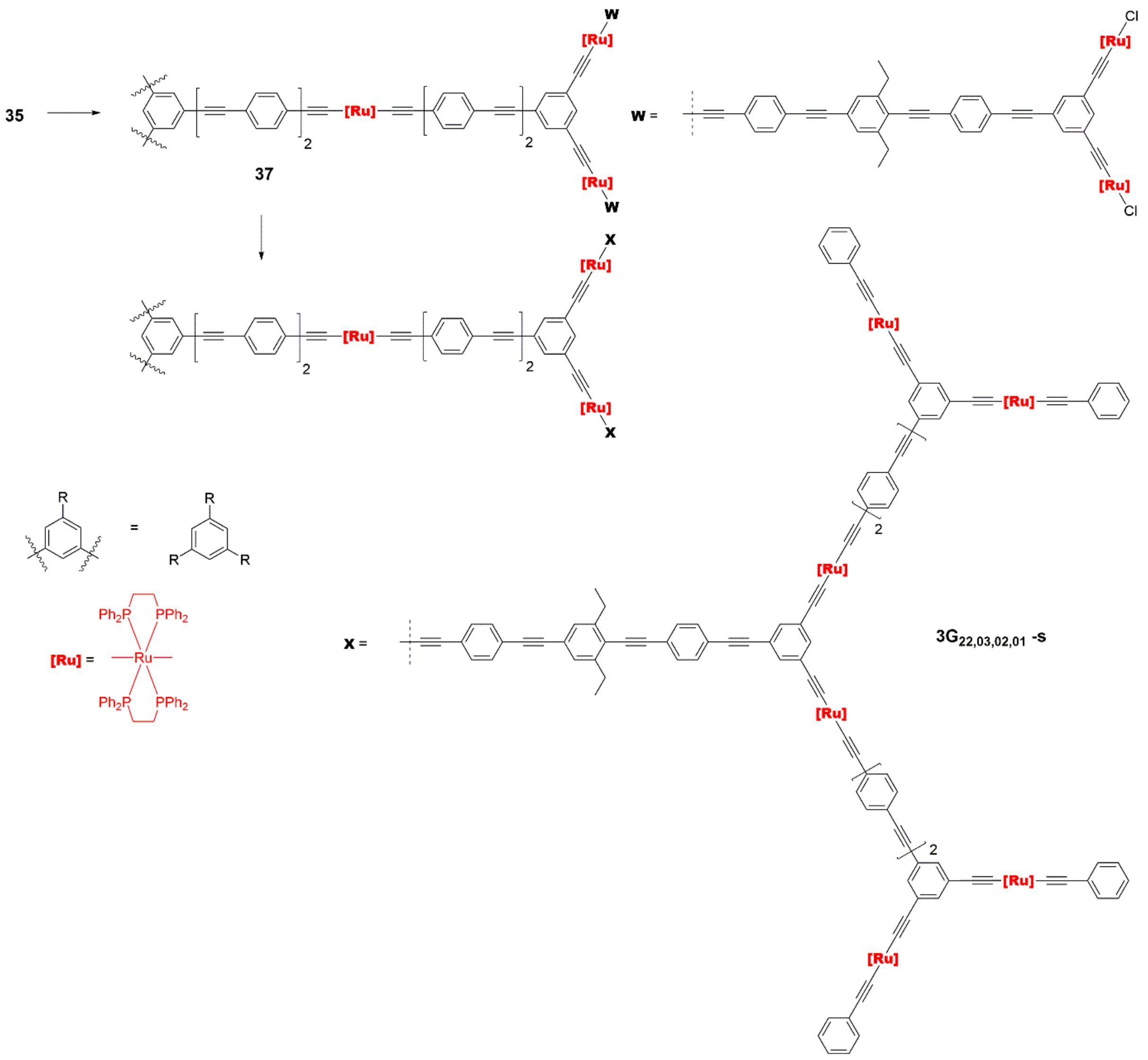
35
37
2
[Ru]
W
W =
Cl
X
X =
R
=
R
R
R
Ph2P
PPh2
[Ru] =
Ru
3G22,03,02,01 -S

Synthesis of 1,3-{trans-[(dppe)$_2$ClRu(C≡C)]}$_2$-5-{(i-Pr)$_3$SiC≡C-1,4-C$_6$H$_4$C≡C-1,4-C$_6$H$_4$C≡C-1,4-C$_6$H$_4$C≡C}C$_6$H$_3$ (16). Compound **8** (0.182 g, 0.36 mmol, 1.4 eq.) and 1,3-{*trans*-[(dppe)$_2$ClRu(C≡C)]}$_2$-5-(HC≡C)C$_6$H$_3$ (0.504 g, 0.25 mmol) were added to a 250 mL flask

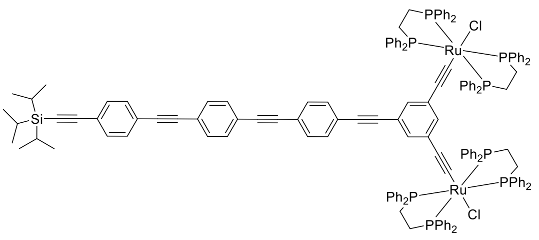

with solvent mixture CH_2Cl_2/NEt_3 (1:1, 120 mL). After deoxygenation three times under nitrogen, Pd(PPh$_3$)$_4$ (0.009 g, 0.01 mmol, 0.03 eq.) and CuI (0.002 g, 0.01 mmol, 0.03 eq.) were added to the solution. The reaction mixture was stirred at room temperature for three days. The crude product was obtained by passing the mixture through a Celite pad and removing the solvent *in vacuo*. The product was purified by precipitation of a CH_2Cl_2 extract from stirring MeOH. Further purification was conducted via alumina column chromatography (eluting with petrol/CH_2Cl_2 = 1:1), to afford **16** as a bright yellow powder (0.452 g, 0.18 mmol, 72%).

^{1}H NMR (700 MHz, CDCl$_3$): δ7.62-7.61 (m, 16H, $H_{[Ru]γ\text{-}3}$ or $H_{[Ru]γ\text{-}7}$), 7.58 (s, 4H, H_{119}, H_{120}), 7.54-7.53 (m, 4H, H_{113}, H_{114}), 7.47 (s, 4H, H_{107}, H_{108}), 7.19-7.10 (m, 16H, $H_{[Ru]γ\text{-}5}$, $H_{[Ru]γ\text{-}9}$), 7.19-7.17 (m, 16H, $H_{[Ru]γ\text{-}3}$ or $H_{[Ru]γ\text{-}7}$), 6.99-6.94 (m, 32H, $H_{[Ru]γ\text{-}4}$, $H_{[Ru]γ\text{-}8}$), 6.60 (s, 1H, H_{126}), 6.44 (s, 2H, H_{125}), 2.76-2.68 (m, 16H, $H_{[Ru]γ\text{-}1}$), 1.14 (s, 21H, H_{p1}, H_{p2}) ppm. ^{13}C NMR (176 MHz, CDCl$_3$) δ 137.0-135.5 (m, $C_{[Ru]γ\text{-}2}$, $C_{[Ru]γ\text{-}6}$), 134.5, 134.4 ($C_{[Ru]γ\text{-}3}$, $C_{[Ru]γ\text{-}7}$), 132.2 (C_{108}), 131.8 (C_{107}), 131.7 (C_{113}, C_{114}), 131.5(C_{119}, C_{120}), 129.9 (C_{125}), 129.7 (C_{126}), 129.6 (C_{201}), 129.1, 128.8 ($C_{[Ru]γ\text{-}5}$, $C_{[Ru]γ\text{-}9}$), 127.4, 127.0 ($C_{[Ru]γ\text{-}4}$, $C_{[Ru]γ\text{-}8}$), 124.6 (C_{117} or C_{121}), 124.4, 124.2 (C_{202}, C_{203}), 123.7 (C_{106}), 123.3 (C_{112}), 123.2 (C_{115}), 123.0 (C_{118}), 122.4 (C_{117} or C_{121}), 121.2 (C_{124}), 114.0 (C_{123}), 106.8 (C_{110}), 93.5 (C_{105}), 93.2 (C_{111}), 91.4 (C_{116}), 91.3 (C_{117}), 91.0 (C_{104}), 87.5 (C_{122}), 31.0 ($C_{[Ru]γ\text{-}1}$), 18.8 (C_{p2}), 11.5 (C_{p1}) ppm. ^{31}P NMR (162 MHz, CDCl$_3$): δ 49.9 ppm. IR: $ν$(C≡C) 2059 cm^{-1}. UV-Vis (CH_2Cl_2, $ν_{max}$ in cm^{-1}, [ε] in 10^3 M^{-1} cm^{-1}): 28 900 [123.7]. HR ESI TOF MS: Calcd. for $C_{151}H_{135}NClP_8Ru_2Si$: 2476.6016. Found: 2476.6040 ([M - Cl + MeCN]$^+$). Anal. Calcd. for $C_{149}H_{132}Cl_2P_8Ru_2Si$: C, 72.41; H, 5.38%; Found: C, 72.48; H, 5.38%.

Synthesis of 1,3-{trans-[(dppe)$_2$ClRu(C≡C)}$_2$-5-{(i-Pr)$_3$SiC≡C-1,4-C$_6$H$_4$C≡C-3,5-Et$_2$-1,4-C$_6$H$_2$C≡C-1,4-C$_6$H$_4$C≡C}C$_6$H$_3$ (17). Compound **4** (0.179 g, 0.28 mmol, 1.4 eq.) and 1,3-{*trans*-[(dppe)$_2$ClRu(C≡C)]}$_2$-5-(HC≡C)C$_6$H$_3$ (0.403 g, 0.20 mmol) were added to a 250 mL flask with solvent mixture

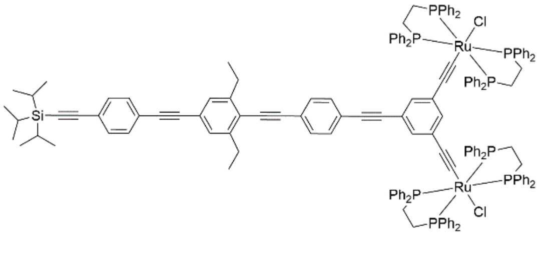

CH_2Cl_2/NEt_3 (2:1, 120 mL). After degassing and backfilling three times with nitrogen, catalytic amounts of Pd(PPh$_3$)$_4$ (0.003 g, 0.002 mmol, 0.01 eq.) and CuI (0.001 g, 0.002 mmol, 0.01 eq.) were added to the solution. The reaction was stirred at room temperature

for three days. The crude product was obtained by passing the mixture through a Celite pad and removing the solvent *in vacuo*. The product was purified by precipitation of a CH_2Cl_2 extract from stirring MeOH. After filtration, the product was washed three times with *n*-pentane. Compound **17** was obtained as a bright yellow powder (0.380 g, 0.15 mmol, 75%).

^{1}H NMR (700 MHz, $CDCl_3$): δ 7.62-7.61 (m, 16H, $H_{[Ru]\gamma-3}$ or $H_{[Ru]\gamma-7}$), 7.59 (s, 4H, H_{119}, H_{120}), 7.47-7.46 (m, 4H, H_{107}, H_{108}), 7.30 (s, 4H, H_{113}, H_{114}), 7.19-7.17 (m, 16H, $H_{[Ru]\gamma-5}$, $H_{[Ru]\gamma-9}$), 7.19-7.17 (m, 16H, $H_{[Ru]\gamma-3}$ or $H_{[Ru]\gamma-7}$), 6.99-6.94 (m, 16H, $H_{[Ru]\gamma-4}$, $H_{[Ru]\gamma-8}$), 6.61 (s, 1H, H_{126}), 6.44 (s, 2H, H_{125}), 2.94 (q, J = 7.6 Hz, 4H, H_{s1}), 2.76-2.69 (m, 16H, $H_{[Ru]\gamma-1}$), 1.35 (t, J = 7.6 Hz, 6H, H_{s2}), 1.14 (s, 21H, H_{p1}, H_{p2}) ppm. ^{13}C NMR (176 MHz, $CDCl_3$) δ 146.7 (C_{114}), 137.0-135.5 (m, $C_{[Ru]\gamma-2}$, $C_{[Ru]\gamma-6}$), 134.5, 134.4 ($C_{[Ru]\gamma-3}$, $C_{[Ru]\gamma-7}$), 132.2 (C_{108}), 131.6 (C_{107}), 131.5 (C_{119}, C_{120}), 129.9 (C_{126}), 129.8 (C_{125}), 129.6 (C_{201}), 129.1, 128.9 ($C_{[Ru]\gamma-5}$, $C_{[Ru]\gamma-9}$), 128.7 (C_{113}), 127.4, 127.0 ($C_{[Ru]\gamma-4}$, $C_{[Ru]\gamma-8}$), 124.4 (C_{118} or C_{121}), 123.6 (C_{109}), 123.3 (C_{106}), 123.1, 122.9 (C_{112}, C_{115}), 122.1 (C_{118} or C_{121}), 121.2 (C_{124}), 114.0 (C_{123}), 98.6 (C_{110}), 93.4 (C_{105}), 91.7 (C_{104}, C_{117}), 88.4 (C_{111}, C_{116}), 87.6 (C_{122}), 31.0 ($C_{[Ru]\gamma-1}$), 28.1 (C_{s1}), 18.8 (C_{p2}), 14.8 (C_{s2}), 11.5 (C_{p1}) ppm. ^{31}P NMR (162 MHz, $CDCl_3$): δ 49.9 ppm. IR: ν(C≡C) 2055 cm^{-1}. UV-Vis (CH_2Cl_2, ν_{max} in cm^{-1}, [ε] in 10^3 M^{-1} cm^{-1}): 28 650 [117.8]. HR ESI TOF MS: Calcd. for $C_{155}H_{143}NClP_8Ru_2Si$: 2532.6638. Found: 2532.6666 ([M - Cl + MeCN]$^+$). Anal. Calcd. for $C_{153}H_{140}Cl_2P_8Ru_2Si$: C, 72.70; H, 5.58%; Found: C, 72.91; H, 5.41%.

Synthesis of 1,3-{trans-[(dppe)$_2$(PhC≡C)Ru(C≡C)]}$_2$-5-{(i-Pr)$_3$SiC≡C-1,4-C$_6$H$_4$C≡C-1,4-C$_6$H$_4$C≡C-1,4-C$_6$H$_4$C≡C}C$_6$H$_3$ (20). Compound **16** (0.240 g, 0.10 mmol) and ethynylbenzene (0.1 mL, 0.93 mmol, 9.3 eq.) were added to freshly distilled CH_2Cl_2 (50 mL) and 1 mL NEt_3 was added to the solution. $NaPF_6$ (0.104 g, 0.62 mmol, 6.2 eq.) was added to the flask and the reaction was stirred at room temperature overnight. The crude product was obtained by passing the mixture through a Celite pad and removing the solvent *in vacuo*. Further purification was conducted by precipitation of a CH_2Cl_2 extract from stirring MeOH (twice) and *n*-pentane (twice), to afford **20** as a yellow powder (0.228 g, 0.09 mmol, 90%).

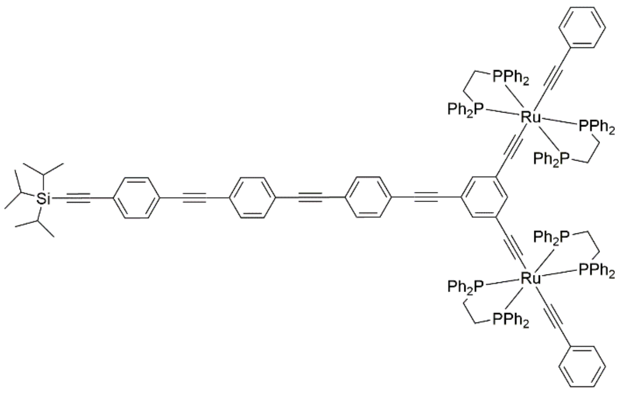

^{1}H NMR (400 MHz, $CDCl_3$): δ 7.60-7.58 (m, 20H, $H_{[Ru]\gamma-3}$ or $H_{[Ru]\gamma-7}$, H_{119}, H_{120}), 7.54 (m, 4H, H_{113}, H_{114}), 7.47 (s, 4H, H_{107}, H_{108}), 7.45-7.44 (m, 16H, $H_{[Ru]\gamma-3}$ or $H_{[Ru]\gamma-7}$), 7.18-7.07 (m, 16H, $H_{[Ru]\gamma-5}$, $H_{[Ru]\gamma-9}$), 7.02-7.00 (m, 2H, H_{209}), 6.96-6.93 (m, 32H, $H_{[Ru]\gamma-4}$, $H_{[Ru]\gamma-8}$), 6.76-6.74 (m, 5H, H_{126}, H_{207})), 6.51 (s, 2H, H_{125}), 2.69 (m, 16H, $H_{[Ru]\gamma-1}$), 1.14 (s, 21H, H_{p1}, H_{p2}) ppm. ^{13}C NMR (176 MHz, $CDCl_3$) δ 137.4-137.1 (m, $C_{[Ru]\gamma-2}$, $C_{[Ru]\gamma-6}$), 134.5, 134.3

($C_{[Ru]\gamma\text{-}3}$, $C_{[Ru]\gamma\text{-}7}$), 132.2 (C_{108}), 131.8 (C_{107}), 131.7 (C_{113}, C_{114}), 131.58, 131.55 (C_{119}, C_{120}), 130.8 (C_{206}), 130.2 (C_{125}, C_{126}, C_{207}), 129.8 (C_{201}), 128.8, 128.7 ($C_{[Ru]\gamma\text{-}5}$, $C_{[Ru]\gamma\text{-}9}$), 127.5 (C_{207}), 127.3, 127.2 ($C_{[Ru]\gamma\text{-}4}$, $C_{[Ru]\gamma\text{-}8}$), 124.7 (C_{118} or C_{121}), 123.7 (C_{109}), 123.3 (C_{106}), 123.2, 123.0 (C_{112}, C_{115}), 122.9 (C_{209}), 122.4 (C_{118} or C_{121}), 121.1 (C_{124}), 117.0 (C_{202} or C_{205}), 116.3 (C_{202} or C_{205}), 106.8 (C_{123}), 93.7 (C_{110}), 93.2 (C_{105}), 91.5, 91.3, 91.1, 90.9 (C_{116}, C_{111}, C_{117}, C_{104}), 87.5 (C_{122}), 31.8-31.7 ($C_{[Ru]\gamma\text{-}1}$), 18.8 (C_{p1}), 11.5 (C_{p2}) ppm. ^{31}P NMR (162 MHz, $CDCl_3$): δ 53.8 ppm. IR: ν(C≡C) 2054 cm^{-1}. UV-Vis (CH_2Cl_2, ν_{max} in cm^{-1}, [ε] in 10^3 M^{-1} cm^{-1}): 28 900 [150.2]. HR ESI MS: Calcd. for $C_{165}H_{143}P_8Ru_2Si$: 2604.7007. Found: 2604.6866 ([M + H]$^+$). Anal. Calcd. for $C_{165}H_{142}P_8Ru_2Si$: C, 76.14; H, 5.50%. Found: C, 76.22; H, 5.38%.

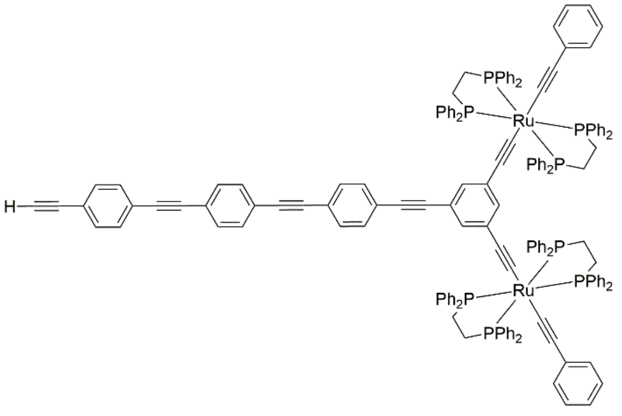

Synthesis of 1,3-{trans-[(dppe)$_2$(PhC≡C)Ru(C≡C)]}$_2$-5-(HC≡C-1,4-C$_6$H$_4$C≡C-1,4-C$_6$H$_4$C≡C-1,4-C$_6$H$_4$C≡C)C$_6$H$_3$ (21). Compound **20** (0.180 g, 0.07 mmol) was dissolved in distilled CH_2Cl_2 (60 mL) and TBAF (1.0 M in THF, 0.1 mL, 1.4 eq.) was added by syringe. The reaction was stirred at room temperature overnight. The crude product was obtained by removing the solvent *in vacuo*. Further purification was conducted by precipitation of a CH_2Cl_2 extract from MeOH and *n*-pentane sequentially, to afford **21** as a bright yellow powder (0.157 g, 0.06 mmol, 93%).

^{1}H NMR (400 MHz, $CDCl_3$): δ 7.60-7.58 (m, 20H, $H_{[Ru]\gamma\text{-}3}$ or $H_{[Ru]\gamma\text{-}7}$, H_{119}, H_{120}), 7.54 (m, 4H, H_{113}, H_{114}), 7.49 (s, 4H, H_{107}, H_{108}), 7.46-7.45 (m, 16H, $H_{[Ru]\gamma\text{-}3}$ or $H_{[Ru]\gamma\text{-}7}$), 7.17-7.08 (m, 16H, $H_{[Ru]\gamma\text{-}5}$, $H_{[Ru]\gamma\text{-}9}$), 7.01-6.99 (m, 2H, H_{209}), 6.96-6.93 (m, 32H, $H_{[Ru]\gamma\text{-}4}$, $H_{[Ru]\gamma\text{-}8}$), 6.75-6.74 (m, 5H, H_{126}, H_{207})), 6.52 (s, 2H, H_{125}), 3.18 (s, 1H, H_{104}), 2.70 (m, 16H, $H_{[Ru]\gamma\text{-}1}$) ppm. ^{13}C NMR (151 MHz, $CDCl_3$) δ 137.4-137.1 (m, $C_{[Ru]\gamma\text{-}2}$, $C_{[Ru]\gamma\text{-}6}$), 134.5, 134.3 ($C_{[Ru]\gamma\text{-}3}$, $C_{[Ru]\gamma\text{-}7}$), 132.3 (C_{108}), 131.82 (C_{107}), 131.75, 131.7 (C_{113}, C_{114}), 131.6 (C_{119}, C_{120}), 130.8 (C_{206}), 130.2 (C_{125}, C_{126}, C_{207}), 129.8 (C_{201}), 128.8, 128.7 ($C_{[Ru]\gamma\text{-}5}$, $C_{[Ru]\gamma\text{-}9}$), 127.5 (C_{208}), 127.3, 127.2 ($C_{[Ru]\gamma\text{-}4}$, $C_{[Ru]\gamma\text{-}8}$), 124.7 (C_{118} or C_{121}), 123.7 (C_{109}), 123.4 (C_{106}), 123.1 (C_{112}, C_{115}), 122.9 (C_{209}), 122.3 (C_{118} or C_{121}), 121.1 (C_{124}), 117.0 (C_{202} or C_{205}), 116.4 (C_{205} or C_{202}). 93.7 (C_{123}), 91.5 (C_{110}), 91.2 (C_{111}), 90.9 (C_{116}, C_{117}), 87.4 (C_{122}), 83.4 (C_{105}), 79.2 (C_{104}), 31.8 ($C_{[Ru]\gamma\text{-}1}$) ppm. ^{31}P NMR (162 MHz, $CDCl_3$): δ 53.8 ppm. HR ESI MS: Calcd. for $C_{156}H_{123}P_8Ru_2$: 2447.5669. Found: 2447.5581 ([M + H]$^+$). IR: ν(C≡C) 2054 cm^{-1}. Anal. Calcd. for $C_{156}H_{122}P_8Ru_2$: C, 76.58; H, 5.03%; Found: C, 76.47; H, 5.16%.

Synthesis of 1,3-{trans-[(dppe)$_2$(1,4-IC$_6$H$_4$C≡C-1,4-C$_6$H$_4$C≡C)Ru(C≡C)]}$_2$-5-{(i-Pr)$_3$SiC≡C-1,4-C$_6$H$_4$C≡C-3,5-Et$_2$-1,4-C$_6$H$_2$C≡C-1,4-C$_6$H$_4$C≡C}C$_6$H$_3$ (18). Compound **17** (0.300 g, 0.12 mmol) and 1-ethynyl-4-{(4-iodophenyl)ethynyl}benzene (0.097 g, 0.30 mmol, 2.5 eq.) were added to distilled, deoxygenated CH_2Cl_2 (100 mL) and 1 mL NEt_3 was added to the solution. $NaPF_6$ (0.104 g, 0.62 mmol, 6.0 eq.) was added to the flask and the reaction was stirred at room temperature overnight. The crude product was obtained by passing the mixture through a Celite pad and removing the solvent *in vacuo*. Further purification was conducted by precipitation of a CH_2Cl_2 extract from MeOH and *n*-pentane several times, to afford **18** as a yellow powder (0.319 g, 0.10 mmol, 87%).

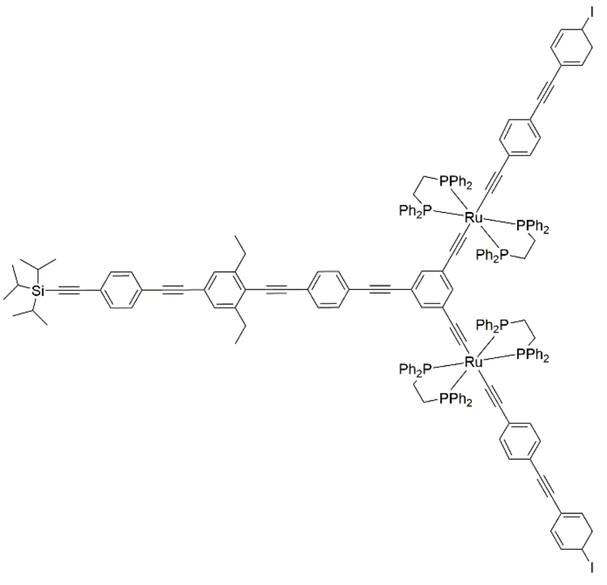

^{1}H NMR (400 MHz, $CDCl_3$): δ 7.70 (m, 4H, H_{214}), 7.67-7.66 (m, 16H, $H_{[Ru]γ\text{-}3}$ or $H_{[Ru]γ\text{-}7}$), 7.64-7.60 (m, 2H, H_{119}), 7.52-7.51 (m, 4H, H_{208}), 7.47 (s, 4H, H_{107}, H_{108}), 7.36-7.34 (m, 16H, $H_{[Ru]γ\text{-}3}$ or $H_{[Ru]γ\text{-}7}$), 7.30-7.29 (m, 2H, H_{113}), 7.27 (m, 4H, H_{213}), 7.24 (s, 4H, H_{207}), 7.17-7.09 (m, 16H, $H_{[Ru]γ\text{-}5}$, $H_{[Ru]γ\text{-}9}$), 6.98-6.90 (m, 32H, $H_{[Ru]γ\text{-}4}$, $H_{[Ru]γ\text{-}8}$), 6.79 (s, 1H, H_{126}), 6.63 (s, 4H, H_{213}), 6.60 (m, 2H, H_{125}), 2.93 (q, J = 7.5 Hz, 4H, H_{s1}), 2.70 (m, 16H, $H_{[Ru]γ\text{-}1}$), 1.35 (t, J = 7.5 Hz, 6H, H_{s2}), 1.14 (s, 21H, H_{p1}, H_{p2}) ppm. ^{13}C NMR (201 MHz, $CDCl_3$) δ 146.7 (C_{114}), 137.6 (C_{214}), 137.3-136.8 (m, $C_{[Ru]γ\text{-}2}$, $C_{[Ru]γ\text{-}6}$), 134.5, 134.1 ($C_{[Ru]γ\text{-}3}$, $C_{[Ru]γ\text{-}7}$), 133.0 (C_{207}), 132.1, 131.7 (C_{107}, C_{108}, C_{208}), 131.5 (C_{119}), 131.0 (C_{213}), 130.1 (C_{125}), 129.8 (C_{126}), 128.9, 128.7 ($C_{[Ru]γ\text{-}5}$, $C_{[Ru]γ\text{-}9}$), 128.67 (C_{113}), 127.3, 127.2 ($C_{[Ru]γ\text{-}4}$, $C_{[Ru]γ\text{-}8}$), 124.4 (C_{118} or C_{121}), 123.6 (C_{209}), 122.8 (C_{118} or C_{121}), 122.0 (C_{112}), 121.3 (C_{115}), 117.5 (C_{124}), 117.0 (C_{212}), 116.3 (C_{202}, C_{205}), 106.8 (C_{123}), 98.6 (C_{117}), 94.6, 93.5 (C_{210}), 93.4 (C_{122}), 93.0 (C_{211}), 92.4 (C_{111}), 91.7, 90.4, 88.7, 88.4, 87.6 , 31.7 ($C_{[Ru]γ\text{-}1}$), 28.1 (C_{s1}), 18.8 (C_{p2}), 14.8 (C_{s2}), 11.5 (C_{p1}) ppm. ^{31}P NMR (162 MHz, $CDCl_3$): δ 53.4 ppm. IR: ν(C≡C) 2049 cm^{-1}. UV-Vis (CH_2Cl_2, ν_{max} in cm^{-1}, [ε] in 10^3 M^{-1} cm^{-1}): 27 950 [185.4]. HR ESI MS: Calcd. for $C_{185}H_{157}I_2P_8Ru_2Si$: 3112.6203. Found: 3112.6117 ([M + H]$^+$). Anal. Calcd. for $C_{185}H_{156}I_2P_8Ru_2Si$: C, 71.42; H, 5.05%. Found: C, 71.37; H, 4.96%.

Synthesis of 1,3-{trans-[(dppe)$_2$(1,4-IC$_6$H$_4$C≡C-1,4-C$_6$H$_4$C≡C)Ru(C≡C)]}$_2$-5-(HC≡C-1,4-C$_6$H$_4$C≡C-3,5-Et$_2$-1,4-C$_6$H$_2$C≡C-1,4-C$_6$H$_4$C≡C)C$_6$H$_3$ (19). Compound **18** (0.202 g, 0.07 mmol) was added to distilled, deoxygenated CH_2Cl_2 (60 mL) with several drops of NEt_3, and TBAF (1.0 M in THF, 0.1 mL, 1.6 eq.) was slowly added by syringe. The reaction was stirred at room temperature for 2 h. The crude product was obtained by passing the mixture

through a Celite pad and removing the solvent *in vacuo*. Further purification was conducted by precipitation of a CH_2Cl_2 extract from MeOH once and *n*-pentane twice, to afford **19** as a yellow powder (0.178 g, 0.06 mmol, 93%).

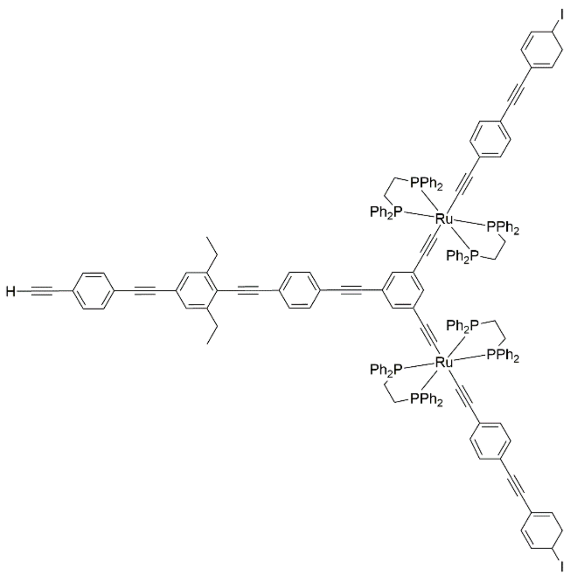

^{1}H NMR (700 MHz, $CDCl_3$) δ 7.71-7.69 (m, 4H, H_{214}), 7.66-7.65 (m, 16H, $H_{[Ru]\gamma-3}$ or $H_{[Ru]\gamma-7}$), 7.61-7.60 (m, 2H, H_{119}), 7.51 (m, 4H, H_{208}), 7.49 (m, 4H, H_{107}, H_{108}), 7.36 (m, 16H, $H_{[Ru]\gamma-3}$ or $H_{[Ru]\gamma-7}$), 7.30 (m, 2H, H_{113}), 7.24 (m, 4H, H_{207}), 7.16-7.11 (m, 16H, $H_{[Ru]\gamma-5}$, $H_{[Ru]\gamma-9}$), 6.97-6.93 (m, 32H, $H_{[Ru]\gamma-4}$, $H_{[Ru]\gamma-8}$), 6.80 (s, 1H, H_{126}), 6.63-6.60 (m, 6H, H_{213}, H_{125}), 3.18 (s, 1H, H_{104}), 2.94 (q, J = 7.6 Hz, 6H, H_{s1}), 2.70 (m, 16H, $H_{[Ru]\gamma-1}$), 1.36 (t, J = 7.6, 6H, H_{s2}) ppm. ^{13}C NMR (176 MHz, $CDCl_3$) δ 146.7 (C_{114}), 137.8, 137.6 (C_{214}), 137.3-136.8 (m, $C_{[Ru]\gamma-2}$, $C_{[Ru]\gamma-6}$), 134.5, 134.1 ($C_{[Ru]\gamma-3}$, $C_{[Ru]\gamma-7}$), 133.3, 133.1 (C_{207}), 132.2, 131.7 (C_{107}, C_{108}, C_{208}), 131.6 (C_{119}), 131.2, 131.0 (C_{113}), 130.1 (C_{125}, C_{213}),129.8 (C_{206}), 128.9, 128.7 ($C_{[Ru]\gamma-5}$, $C_{[Ru]\gamma-9}$), 127.3, 127.2 ($C_{[Ru]\gamma-4}$, $C_{[Ru]\gamma-8}$), 124.4 (C_{118} or C_{121}), 123.6 (C_{209}), 122.7, 122.1, 121.3, 117.5 (C_{124}), 117.0 (C_{212}), 116.3 (C_{202}, C_{205}), 31.7 ($C_{[Ru]\gamma-1}$), 28.1 (C_{s1}), 14.8 (C_{s2}) ppm. ^{31}P NMR (162 MHz, $CDCl_3$): δ 53.6 ppm. IR: ν(C≡C) 2047 cm^{-1}. HR ESI MS: Calcd. for $C_{176}H_{137}I_2P_8Ru_2$: 2955.4744. Found: 2955.4798 ([M + H]$^+$). Anal. Calcd. for $C_{176}H_{136}I_2P_8Ru_2$: C, 71.54; H, 4.64%; Found: C, 71.68; H, 4.64%.

Synthesis of 1,3-{trans-[(1,3-trans-[(dppe)$_2$(PhC≡C)Ru(C≡C)]$_2$C$_6$H$_3$-5-C≡C-1,4-C$_6$H$_4$C≡C-1,4-C$_6$H$_4$C≡C)Ru(dppe)$_2$(C≡C)]}$_2$-5-{(i-Pr)$_3$SiC≡C-1,4-C$_6$H$_4$C≡C-1,4-C$_6$H$_4$C≡C-1,4-C$_6$H$_4$C≡C}C$_6$H$_3$ (24). Compound **16** (0.161 g, 0.065 mmol) and 1,3-{*trans*-[(dppe)$_2$(PhC≡C)Ru(C≡C)]}$_2$-5-(HC≡C-1,4-C_6H_4C≡C-1,4-C_6H_4C≡C)C_6H_3 (0.349 g, 0.15 mmol, 2.3 eq.) were added to distilled, deoxygenated CH_2Cl_2 (100 mL) and NEt_3 (2 mL) was added to the solution. $NaPF_6$ (0.218 g, 1.30 mmol, 20.0 eq.) was added to the flask. The reaction was stirred at room temperature overnight. The crude product was obtained by passing the mixture through a Celite pad and removing the solvent *in vacuo*. Further purification was conducted by sequential precipitation of a CH_2Cl_2 extract from firstly MeOH and then *n*-pentane, to afford **24** as a bright yellow powder (0.453 g, 0.065 mmol, 99%).

^{1}H NMR (400 MHz, $CDCl_3$): δ 7.66 (m, 16H, $H_{[Ru]\gamma-3}$ or $H_{[Ru]\gamma-7}$), 7.59 (m, 32H, $H_{[Ru]\delta-3}$ or $H_{[Ru]\delta-7}$), 7.54 (m, 4H, H_{113}, H_{114}), 7.46-7.45 (m, 48H, H_{213}, H_{214}, H_{107}, H_{108}, $H_{[Ru]\delta-3}$ or $H_{[Ru]\delta-7}$), 7.37-7.36 (m, 16H, $H_{[Ru]\gamma-3}$ or $H_{[Ru]\gamma-7}$), 7.34-7.33 (m, 4H, H_{208}), 7.18-7.10 (m, 48H, $H_{[Ru]\gamma-5}$, $H_{[Ru]\gamma-9}$, $H_{[Ru]\delta-5}$, $H_{[Ru]\delta-9}$), 7.00 (m, 4H, H_{309}), 6.94 (m, 96H, $H_{[Ru]\gamma-4}$, $H_{[Ru]\gamma-8}$, $H_{[Ru]\delta-4}$,

$H_{[Ru]δ-8}$), 6.76-6.53 (m, 29H, H_{125}, H_{126}, H_{207}, H_{219}, H_{220}, H_{307}, H_{308}), 2.70 (m, 48H, $H_{[Ru]γ-1}$, $H_{[Ru]δ-1}$), 1.14 (s, 21H, H_{p1}, H_{p2}) ppm. ^{13}C NMR (176 MHz, $CDCl_3$) δ 137.3-137.2 (m, $C_{[Ru]γ-2}$, $C_{[Ru]γ-6}$, $C_{[Ru]δ-2}$, $C_{[Ru]δ-6}$), 134.5, 134.2 ($C_{[Ru]γ-3}$, $C_{[Ru]γ-7}$, $C_{[Ru]δ-3}$, $C_{[Ru]δ-7}$), 132.2 (C_{107} or C_{108}), 131.8, 131.5 (C_{119}, C_{120}), 131.0 (C_{208}), 130.8, 130.2, 130.1 (C_{125}, C_{126}, C_{207}, C_{219}, C_{220}), 129.8, 129.0, 128.8, 128.73, 128.65 ($C_{[Ru]γ-5}$, $C_{[Ru]γ-9}$, $C_{[Ru]δ-5}$, $C_{[Ru]δ-9}$), 127.5 (C_{308}), 127.3, 127.2 ($C_{[Ru]γ-4}$, $C_{[Ru]γ-8}$, $C_{[Ru]δ-4}$, $C_{[Ru]δ-8}$), 123.4, 122.9 (C_{309}), 121.2, 93.2, 92.7, 91.3, 90.9, 89.6, 31.8 ($C_{[Ru]γ-1}$, $C_{[Ru]δ-1}$), 18.8 (C_{p2}), 11.5 (C_{p1}) ppm. ^{31}P NMR (162 MHz, $CDCl_3$): δ 53.8 (s, 16P, $P_{[Ru]δ}$), 53.6 (s, 8P, $P_{[Ru]γ}$) ppm. IR: $ν$(C≡C) 2051 cm^{-1}. UV-Vis (CH_2Cl_2, $ν_{max}$ in cm^{-1}, [ε] in 10^3 M^{-1} cm^{-1}): 29 150 [331.4]. ESI MS *m/z* (%): 999 ([Ru(C≡CPh)(dppe)$_2$]$^+$, 30), 1027 ([Ru(C≡CPh)(CO)(dppe)$_2$]$^+$, 100). Anal. Calcd. for $C_{445}H_{366}P_{24}Ru_6Si$: C, 75.37; H, 5.20%; Found: C, 74.96; H, 5.17%.

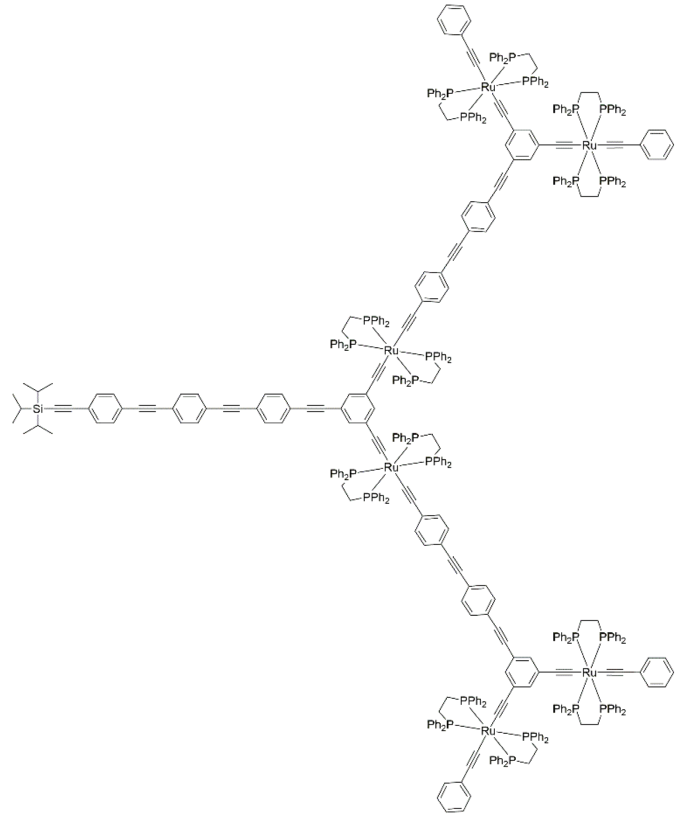

Synthesis of 1,3-{trans-[(1,3-{trans-[(dppe)$_2$(PhC≡C)Ru(C≡C)]}$_2$C$_6$H$_3$-5-C≡C-1,4-C$_6$H$_4$C≡C-1,4-C$_6$H$_4$C≡C)(dppe)$_2$Ru(C≡C)]}$_2$-5-(HC≡C-1,4-C$_6$H$_4$C≡C-1,4-C$_6$H$_4$C≡C-1,4-C$_6$H$_4$C≡C)C$_6$H$_3$ (25). Compound **24** (0.487 g, 0.07 mmol) was added to distilled deoxygenated CH_2Cl_2 (60 mL) and TBAF (0.1 mL,1.0 M in THF, 15.0 eq.) was added by syringe. The reaction was stirred at room temperature to react overnight. The crude product was obtained by removing the solvent *in vacuo*. Further purification was conducted following precipitation of a CH_2Cl_2 extract from stirring MeOH and *n*-pentane several times, to afford a bright-yellow powder identified as **25** (0.471 g, 0.07 mmol, 99%).

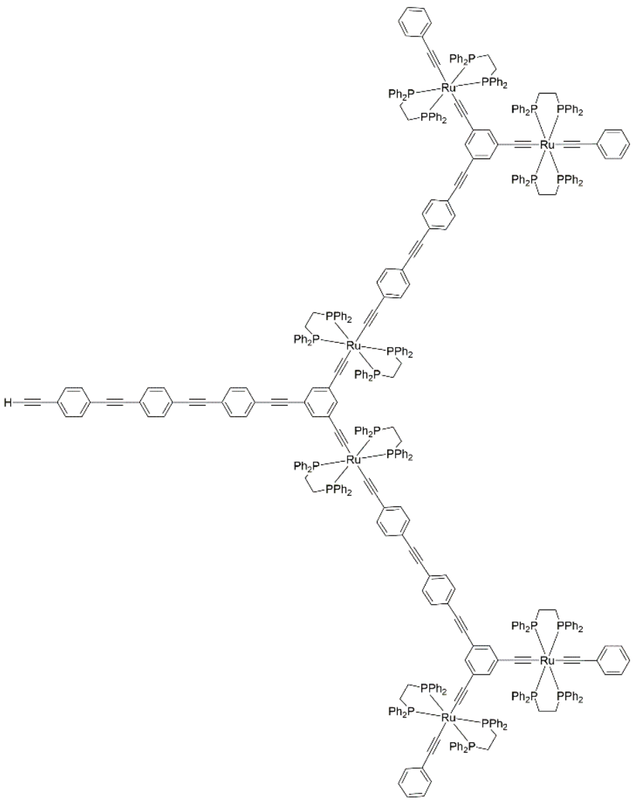

^{1}H NMR (400 MHz, $CDCl_3$): δ 7.67-7.65 (m, 16H, $H_{[Ru]\gamma\text{-}3}$ or $H_{[Ru]\gamma\text{-}7}$), 7.60-7.59 (m, 36H, $H_{[Ru]\delta\text{-}3}$ or $H_{[Ru]\delta\text{-}7}$, H_{119}, H_{120}), 7.54 (m, 4H, H_{113}, H_{114}), 7.49 (s, 4H, H_{107}, H_{108}), 7.46-7.45 (m, 40H, H_{213}, H_{214}, $H_{[Ru]\delta\text{-}3}$ or $H_{[Ru]\delta\text{-}7}$), 7.37-7.35 (m, 16H, $H_{[Ru]\gamma\text{-}3}$ or $H_{[Ru]\gamma\text{-}7}$), 7.32 (m, 4H, H_{208}), 7.19-7.09 (m, 48H, $H_{[Ru]\gamma\text{-}5}$,$H_{[Ru]\gamma\text{-}9}$, $H_{[Ru]\delta\text{-}5}$, $H_{[Ru]\delta\text{-}9}$), 7.02-6.99 (m, 4H, H_{309}), 6.96-6.93 (m, 96H, $H_{[Ru]\gamma\text{-}4}$,$H_{[Ru]\gamma\text{-}8}$, $H_{[Ru]\delta\text{-}4}$, $H_{[Ru]\delta\text{-}8}$), 6.76-6.53 (m, 29H, H_{125}, H_{126}, H_{207}, H_{219}, H_{220}, H_{307}, H_{308}), 3.19 (s, 1H, H_{104}), 2.70 (m, 48H, $H_{[Ru]\gamma\text{-}1}$, $H_{[Ru]\delta\text{-}1}$) ppm. ^{13}C NMR (176 MHz, $CDCl_3$) δ 137.2-137.0 (m, $C_{[Ru]\gamma\text{-}2}$,$C_{[Ru]\gamma\text{-}6}$, $C_{[Ru]\delta\text{-}2}$, $C_{[Ru]\delta\text{-}6}$), 136.7, 134.3, 134.2 ($C_{[Ru]\gamma\text{-}3}$,$C_{[Ru]\gamma\text{-}7}$, $C_{[Ru]\delta\text{-}3}$, $C_{[Ru]\delta\text{-}7}$), 132.1 (C_{107}, C_{108}), 131.7, 131.6, 131.5-131.4 (C_{119}, C_{120}), 130.9 (C_{208}), 130.7, 130.0 (C_{125}, C_{126}, C_{207}, C_{219}, C_{220}), 129.7, 128.8, 128.7, 128.6, 128.5 ($C_{[Ru]\gamma\text{-}5}$,$C_{[Ru]\gamma\text{-}9}$, $C_{[Ru]\delta\text{-}5}$, $C_{[Ru]\delta\text{-}9}$), 127.4 (C_{308}), 127.1, 127.0 ($C_{[Ru]\gamma\text{-}4}$,$C_{[Ru]\gamma\text{-}8}$, $C_{[Ru]\delta\text{-}4}$, $C_{[Ru]\delta\text{-}8}$), 124.6, 124.5, 123.7, 123.5, 123.3, 122.9, 122.8 (C_{309}), 122.3, 122.2, 122.1, 121.14, 121.10, 117.4, 116.9, 116.5, 116.2, 93.5, 93.1, 92.6, 91.1, 90.8, 89.5, 87.4, 83.3, 79.1, 31.8 ($C_{[Ru]\gamma\text{-}1}$, $C_{[Ru]\delta\text{-}1}$) ppm. ^{31}P NMR (162 MHz, $CDCl_3$): δ 53.9 (s, 16P, $P_{[Ru]\delta}$), 53.6 (s, 8P, $P_{[Ru]\gamma}$) ppm. IR: *ν*(C≡C) 2053 cm^{-1}. ESI MS *m/z* (%): 999 ([Ru(C≡CPh)(dppe)$_2$]$^+$, 34), 1027 ([Ru(C≡CPh)(CO)(dppe)$_2$]$^+$, 100). Anal. Calcd. for $C_{436}H_{346}P_{24}Ru_6$: C, 75.51; H, 5.03%; Found: C, 75.43; H, 4.96%.

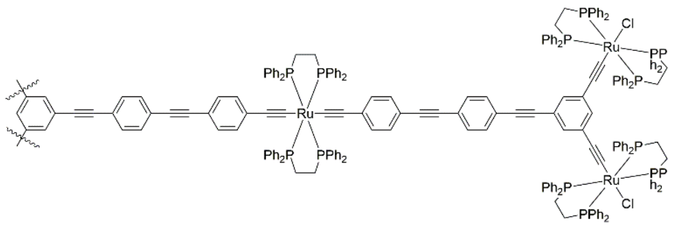

Synthesis of 1,3,5-{trans-[{3,5-{trans-[(dppe)$_2$ClRu(C≡C)]}$_2$C$_6$H$_3$-1-(C≡C-1,4-C$_6$H$_4$C≡C-1,4-C$_6$H$_4$C≡C)}Ru(dppe)$_2$(C≡C-1,4-C$_6$H$_4$C≡C-1,4-C$_6$H$_4$C≡C)]}$_3$C$_6$H$_3$ (32). 1,3,5-{*trans*-[(1,4-IC$_6$H$_4$C≡C-1,4-C$_6$H$_4$C≡C)Ru(dppe)$_2$(C≡C-1,4-C$_6$H$_4$C≡C-1,4-C$_6$H$_4$C≡C)]}$_3$C$_6$H$_3$ (0.088 g, 0.020 mmol) and 1,3-{*trans*-[(dppe)$_2$ClRu(C≡C)]}$_2$-5-(HC≡C)C$_6$H$_3$ (0.121 g, 0.060 mmol, 3.0 eq.) were added to distilled deoxygenated CH_2Cl_2 (20 mL) and several drops of NEt_3. The solution was degassed and backfilled with nitrogen three times. Catalytic amounts of $Pd(PPh_3)_4$ and CuI were added to the solution. The reaction was conducted overnight at room temperature. The crude product was obtained by removing the solvent *in vacuo*. Further purification was conducted by precipitation of a CH_2Cl_2 extract from stirring MeOH and *n*-pentane, to afford **32** as a bright yellow powder (0.143 g, 0.014 mmol, 71%).

^{1}H NMR (700 MHz, CDCl$_3$): δ 7.69 (s, 3H, H_0), 7.62-7.61 (m, 72H, $H_{[Ru]α-3}$ or $H_{[Ru]α-7}$, $H_{[Ru]β-3}$ or $H_{[Ru]β-7}$), 7.58-7.54 (m, 24H, H_5, H_6, H_{25}, H_{26}), 7.51 (m, 72H, $H_{[Ru]α-3}$ or $H_{[Ru]α-7}$, $H_{[Ru]β-3}$ or $H_{[Ru]β-7}$), 7.35-7.33 (m, 12H, H_{11}, H_{20}), 7.20-7.11 (m, 48H, $H_{[Ru]α-5}$, $H_{[Ru]α-9}$, $H_{[Ru]β-5}$, $H_{[Ru]β-9}$), 6.99-6.95 (m, 144H, $H_{[Ru]α-4}$, $H_{[Ru]α-8}$, $H_{[Ru]β-4}$, $H_{[Ru]β-8}$), 6.72-6.70 (m, 12H, H_{12}, H_{19}), 6.60 (s, 6H, H_{32}), 6.45 (s, 3H, H_{31}), 2.77-2.65 (m, 72H, $H_{[Ru]α-1}$, $H_{[Ru]β-1}$) ppm. ^{13}C NMR (151 MHz, CDCl$_3$) δ 137.0-135.5 (m, $C_{[Ru]α-2}$, $C_{[Ru]α-6}$, $C_{[Ru]β-2}$, $C_{[Ru]β-6}$), 134.5, 134.4, 134.3 ($C_{[Ru]α-3}$, $C_{[Ru]α-7}$, $C_{[Ru]β-3}$, $C_{[Ru]β-7}$), 131.8, 131.6, 131.5 (C_{11}, C_{20}), 131.1, 131.1, 130.1 (C_{12}, C_{19}), 129.9, 129.8 (C_{31}), 129.1, 128.8 ($C_{[Ru]α-4}$, $C_{[Ru]α-8}$, $C_{[Ru]β-4}$, $C_{[Ru]β-8}$), 128.1, 127.4, 127.0 ($C_{[Ru]α-5}$, $C_{[Ru]α-9}$, $C_{[Ru]β-5}$, $C_{[Ru]β-9}$), 124.4, 124.2, 124.1, 124.0, 123.8, 123.4 (C_5, C_6, C_{25}, C_{26}), 122.1, 121.3, 118.0 (C_{14}, C_{17}), 116.8, 116.7 (C_{13}, C_{18}), 114.0 (C_{102}), 93.14, 93.11, 92.7, 90.8, 90.5, 89.6, 89.4, 87.8, 31.3 ($C_{[Ru]α-1}$, $C_{[Ru]β-1}$) ppm. ^{31}P NMR (162 MHz, CDCl$_3$): δ 53.4 (s, 12P, $P_{[Ru]α}$), 49.9 (s, 24P, $P_{[Ru]β}$) ppm. IR: *ν*(C≡C) 2050 cm^{-1}. UV-Vis (CH_2Cl_2, $ν_{max}$ in cm^{-1}, [ε] in 10^3 M^{-1} cm^{-1}): 23 750 [197.5, sh], 29 400 [349.3]. Anal. Calcd. for $C_{612}H_{492}Cl_6P_{36}Ru_9$: C, 72.89; H, 4.93%. Found: C, 72.80; H, 5.07%.

Synthesis of 1,3,5-{trans-[{3,5-{trans-[(dppe)$_2$(1,4-IC$_6$H$_4$C≡C-2,6-Et$_2$-1,4-C$_6$H$_2$C≡C-1,4-C$_6$H$_4$C≡C)Ru(C≡C)]}$_2$C$_6$H$_3$-1-(C≡C-1,4-C$_6$H$_4$C≡C-1,4-C$_6$H$_4$C≡C)}Ru(dppe)$_2${C≡C-1,4-C$_6$H$_4$C≡C-1,4-C$_6$H$_4$C≡C}]}$_3$C$_6$H$_3$ (35). Compound **4** (0.042 g, 0.066 mmol, 6.6 eq.) was added to distilled deoxygenated CH_2Cl_2 (60 mL) and the solvent was degassed and backfilled with nitrogen three times. TBAF (0.1 mL, 1.0 M in THF, 10.0 eq.) was added by syringe and the mixture stirred for about 1 h until TLC indicated reaction completion. **32** (0.101 g, 0.010 mmol) and $NaPF_6$ (0.104 g, 0.62 mmol, 60.0 eq.) were added and the reaction mixture stirred overnight at 35 °C. The crude product was obtained by passing the mixture through a Celite pad and removing the solvent *in vacuo*. Further purification was conducted by precipitation of a CH_2Cl_2 extract from stirring MeOH and *n*-pentane several times, to afford **35** as a yellow powder (0.088 g, 0.0071 mmol, 71%).

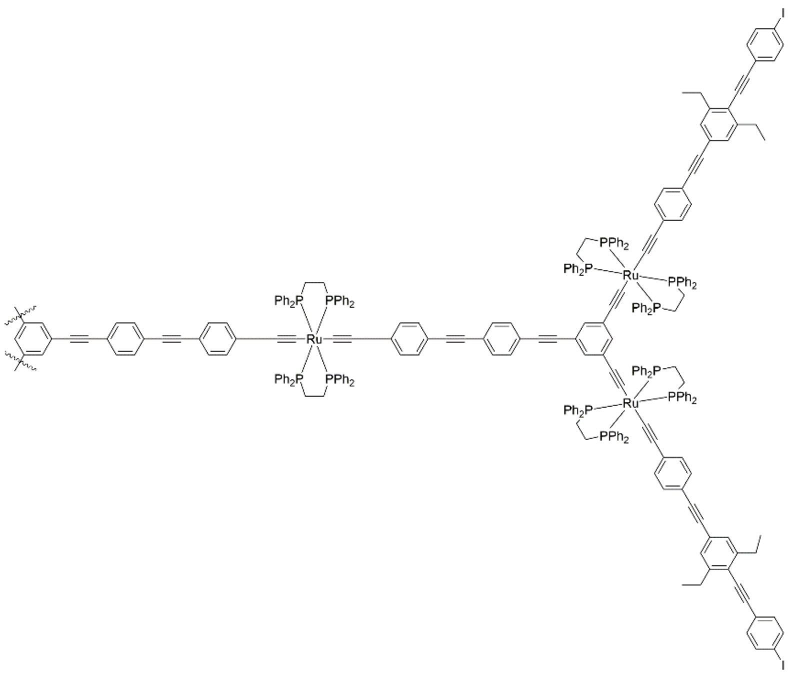

^{1}H NMR (400 MHz, CDCl$_3$): δ 7.72-7.71 (m, 15H, H$_0$, H$_{120}$), 7.66-7.36 (m, 144H, H$_{[Ru]\alpha-3}$, H$_{[Ru]\alpha-7}$, H$_{[Ru]\beta-3}$, H$_{[Ru]\beta-7}$), 7.31-7.29 (m, 36H, H$_{11}$, H$_{20}$, H$_{113}$, H$_{119}$), 7.19-7.12 (m, 72H, H$_{[Ru]\alpha-5}$, H$_{[Ru]\alpha-5}$, H$_{[Ru]\beta-9}$, H$_{[Ru]\beta-9}$), 6.98-6.94 (m, 144H, H$_{[Ru]\alpha-4}$, H$_{[Ru]\alpha-8}$, H$_{[Ru]\beta-4}$, H$_{[Ru]\beta-8}$), 6.79-6.62 (m, 33H, H$_{12}$, H$_{19}$, H$_{31}$, H$_{32}$, H$_{107}$), 2.89 (q, J = 7.5 Hz, 24H, H$_{s1}$), 2.71-2.65 (m, 72H, H$_{[Ru]\alpha-1}$, H$_{[Ru]\beta-1}$), 1.34 (t, J = 7.5 Hz, 36H, H$_{s2}$) ppm. ^{13}C NMR (151 MHz, CDCl$_3$) δ 146.6 (C$_{114}$), 137.8 (C$_{120}$), 137.3-136.9 (m, C$_{[Ru]\alpha-2}$, C$_{[Ru]\alpha-6}$, C$_{[Ru]\beta-2}$, C$_{[Ru]\beta-6}$), 134.5, 134.3, 134.2 (C$_{[Ru]\alpha-3}$, C$_{[Ru]\alpha-7}$, C$_{[Ru]\beta-3}$, C$_{[Ru]\beta-7}$), 133.0 (C$_{119}$), 131.8, 131.6 (C$_{10}$, C$_{21}$), 131.0, 130.1 (C$_{12}$, C$_{19}$, C$_{32}$, C$_{31}$, C$_{107}$), 129.0, 128.7 (C$_{[Ru]\alpha-5}$, C$_{[Ru]\alpha-9}$, C$_{[Ru]\beta-5}$, C$_{[Ru]\beta-9}$), 128.5 (C$_{11}$, C$_{20}$, C$_{113}$), 127.3 (C$_{[Ru]\alpha-4}$, C$_{[Ru]\alpha-8}$, C$_{[Ru]\beta-4}$, C$_{[Ru]\beta-8}$), 124.0, 123.4 (C$_{121}$), 121.0 (C$_{115}$, C$_{118}$), 116.7 (C$_{13}$, C$_{18}$, C$_{101}$, C$_{106}$), 97.4, 94.1 (C$_{117}$), 92.2, 90.0 (C$_{116}$), 88.3 (C$_9$, C$_{22}$, C$_{111}$), 31.7 (C$_{[Ru]\alpha-1}$, C$_{[Ru]\beta-1}$), 28.1 (C$_{s1}$), 14.8 (C$_{s2}$) ppm. ^{31}P NMR (162 MHz, CDCl$_3$): δ 53.6 (s, 24P, P$_{[Ru]\alpha}$), 53.4 (s, 12P, P$_{[Ru]\beta}$) ppm. IR: ν(C≡C) 2049 cm^{-1}. UV-Vis (CH$_2$Cl$_2$, ν_{max} in cm^{-1}, [ε] in 10^3 M^{-1} cm^{-1}): 28 650 [662.2]. Anal. Calcd. for $C_{780}H_{612}I_6P_{36}Ru_9$: C, 73.35; H, 4.83%; Found: C, 73.50; H, 4.86%.

Synthesis of 1,3,5-{trans-[1,3-{trans-[1,3-{trans-[(dppe)$_2$ClRu(C≡C)]}$_2$C$_6$H$_3$-5-(C≡C-1,4-C$_6$H$_4$C≡C-2,6-Et$_2$-1,4-C$_6$H$_2$C≡C-1,4-C$_6$H$_4$C≡C)Ru(dppe)$_2$(C≡C)]}$_2$C$_6$H$_3$-5-(C≡C-1,4-C$_6$H$_4$C≡C-1,4-C$_6$H$_4$C≡C)Ru(dppe)$_2$(C≡C-1,4-C$_6$H$_4$C≡C-1,4-C$_6$H$_4$C≡C)]}$_3$C$_6$H$_3$ (37). Compound **35** (0.080 g, 0.006 mmol) and 1,3-{*trans*-[(dppe)$_2$ClRu(C≡C)]}$_2$-5-(HC≡C)C$_6$H$_3$ (0.083 g, 0.041 mmol, 6.6 eq.) were added to distilled deoxygenated CH_2Cl_2 (30 mL) mixed with triethylamine (30 mL). The solution was deoxygenated three times under nitrogen. Catalytic amounts of Pd(PPh$_3$)$_4$ and [Cu(MeCN)$_4$]PF$_6$ were added to the solution. The reaction was conducted for three days at room temperature. The crude product was obtained by pouring into stirring MeOH and collected by filtration with a sintered funnel. Further purification was conducted by precipitation from MeOH (50 mL), Et_2O (25 mL) and washing with *n*-pentane (20 mL), to afford **37** as a bright yellow powder (0.094 g, 0.004 mmol, 64%).

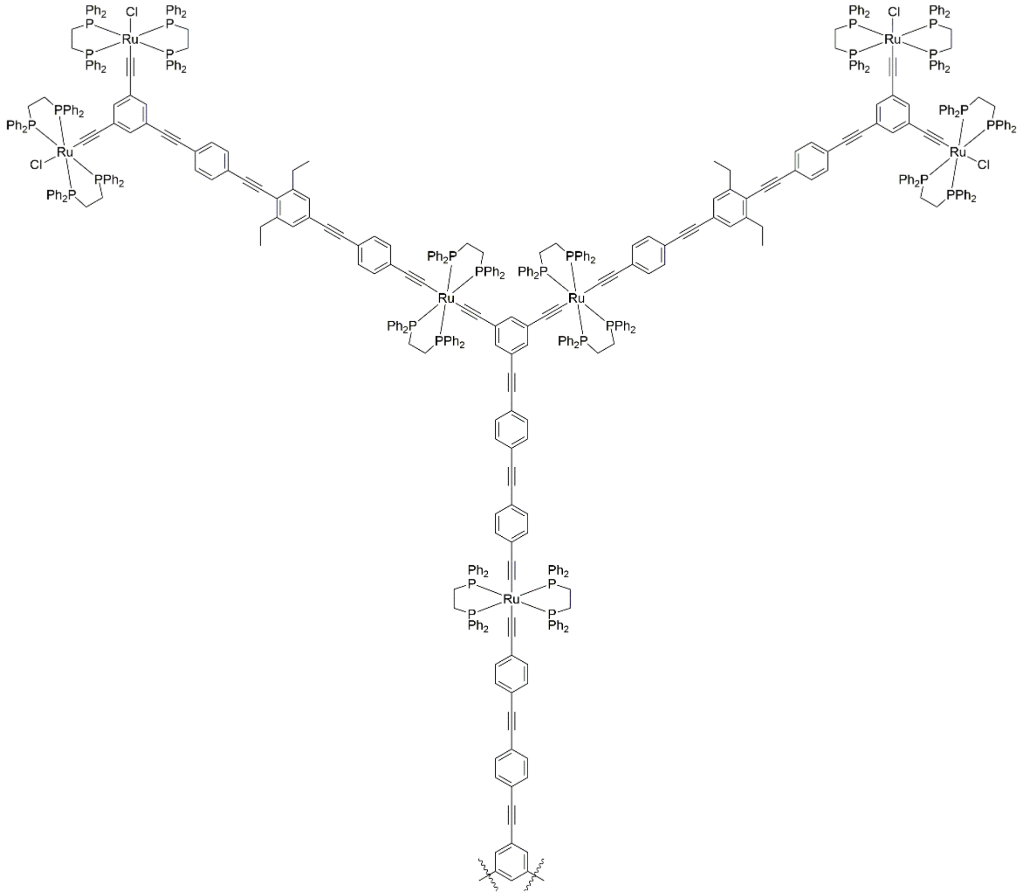

^{1}H NMR (700 MHz, CDCl$_3$): δ 7.69 (m, 3H, H_0), 7.65-7.36 (m, 336H, $H_{[Ru]\alpha-3}$, $H_{[Ru]\alpha-7}$, $H_{[Ru]\beta-3}$, $H_{[Ru]\beta-7}$, $H_{[Ru]\gamma-3}$, $H_{[Ru]\gamma-7}$), 7.30 (m, 24H, H_{11}, H_{20}, H_{108}), 7.18-7.12 36 (m, 168H, $H_{[Ru]\alpha-5}$, $H_{[Ru]\alpha-9}$, $H_{[Ru]\beta-5}$, $H_{[Ru]\beta-9}$, $H_{[Ru]\gamma-5}$, $H_{[Ru]\gamma-9}$), 6.98-6.95 (m, 336H, $H_{[Ru]\alpha-4}$, $H_{[Ru]\alpha-8}$, $H_{[Ru]\beta-4}$, $H_{[Ru]\beta-8}$, $H_{[Ru]\gamma-4}$, $H_{[Ru]\gamma-8}$), 6.71-6.61 (m, 27H, H_{12}, H_{19}, H_{31}, H_{32}, H_{126}), 6.44 (m, 12H, H_{125}), 2.94 (m, 48H, H_{s1}), 2.77-2.69 (m, 168H, $H_{[Ru]\alpha-1}$, $H_{[Ru]\beta-1}$, $H_{[Ru]\gamma-1}$), 1.37 (m, 72H, H_{s2}) ppm. ^{13}C NMR (176 MHz, CDCl$_3$) δ 137.3, 137.0-135.5 (m, $C_{[Ru]\alpha-2}$, $C_{[Ru]\alpha-6}$, $C_{[Ru]\beta-2}$, $C_{[Ru]\beta-6}$, $C_{[Ru]\gamma-2}$, $C_{[Ru]\gamma-6}$), 134.4 ($C_{[Ru]\alpha-3}$, $C_{[Ru]\alpha-7}$, $C_{[Ru]\beta-3}$, $C_{[Ru]\beta-7}$, $C_{[Ru]\gamma-3}$, $C_{[Ru]\gamma-7}$), 131.7, 131.5, 131.0 (C_{11}, C_{12}, C_{19}, C_{20}, C_{31}, C_{32}), 130.1, 129.9, 129.1, 128.8 ($C_{[Ru]\alpha-5}$, $C_{[Ru]\alpha-9}$, $C_{[Ru]\beta-}$

5, $C_{[Ru]\beta\text{-}9}$, $C_{[Ru]\gamma\text{-}5}$, $C_{[Ru]\gamma\text{-}9}$), 128.7, 128.4, 127.4, 127.2, 127.0 ($C_{[Ru]\alpha\text{-}4}$, $C_{[Ru]\alpha\text{-}8}$, $C_{[Ru]\beta\text{-}4}$, $C_{[Ru]\beta\text{-}}$8, $C_{[Ru]\gamma\text{-}4}$, $C_{[Ru]\gamma\text{-}8}$), 114.0 (C_{202}), 31.7, 31.0 ($C_{[Ru]\alpha\text{-}1}$, $C_{[Ru]\beta\text{-}1}$, $C_{[Ru]\gamma\text{-}1}$), 28.1 (C_{s1}), 14.8 (C_{s2}) ppm. ^{31}P NMR (162 MHz, $CDCl_3$): δ 53.6 (s, 24P, $P_{[Ru]\beta}$), 53.4 (s, 12P, $P_{[Ru]\alpha}$), 49.9 (s, 48P, $P_{[Ru]\gamma}$) ppm. IR: *ν*(C≡C) 2051 cm^{-1}. UV-Vis (CH_2Cl_2, ν_{max} in cm^{-1}, [ε] in 10^3 M^{-1} cm^{-1}): 23 600 [439.4, sh], 27 450 [907.2]. Anal. Calcd. for $C_{1476}H_{1206}Cl_{12}P_{84}Ru_{21}$: C, 73.58; H, 5.05%. Found: C, 73.69; H, 5.12%.

Synthesis of 1,3,5-{trans-[{3,5-{trans-[(1,3-{trans-[(dppe)$_2$(PhC≡C)Ru(C≡C)]}$_2$-5-C$_6$H$_3$(C≡C-1,4-C$_6$H$_4$C≡C-1,4-C$_6$H$_4$C≡C-1,4-C$_6$H$_4$C≡C)Ru(dppe)$_2$(C≡C)]}$_2$C$_6$H$_3$-1-(C≡C-1,4-C$_6$H$_4$C≡C-1,4-C$_6$H$_4$C≡C)}Ru(dppe)$_2$(C≡C-1,4-C$_6$H$_4$C≡C-1,4-C$_6$H$_4$C≡C)]}$_3$C$_6$H$_3$ (2G$_{22,03,01}$). Compound **21** (0.088 g, 0.004 mmol, 7.2 eq.) and **32** (0.054 g, 0.005 mmol) were added to distilled CH_2Cl_2 (60 mL) and NEt_3 (1 mL) was added to the solution. $NaPF_6$ (0.104 g, 0.62 mmol, 20.0 eq.) was added to the flask and the reaction was stirred at room temperature for three days. The crude product was obtained by passing the solution through a Celite pad and removing the solvent *in vacuo*. Further purification was conducted by the sequential precipitation of a CH_2Cl_2 extract from firstly stirring MeOH and then *n*-pentane, to afford a yellow powder identified as **2G$_{22,03,01}$** (0.118 g, 0.004 mmol, 96%).

^{1}H NMR (600 MHz, $CDCl_3$): δ 7.69 (m, 3H, H_0), 7.66-7.36 (m, 336H, $H_{[Ru]\alpha\text{-}3}$, $H_{[Ru]\alpha\text{-}7}$, $H_{[Ru]\beta\text{-}3}$, $H_{[Ru]\beta\text{-}7}$, $H_{[Ru]\gamma\text{-}3}$, $H_{[Ru]\gamma\text{-}7}$), 7.32-7.30 (m, 24H, H_{11}, H_{20}, H_{208}), 7.16-7.10 (m, 168H, $H_{[Ru]\alpha\text{-}5}$, $H_{[Ru]\alpha\text{-}9}$, $H_{[Ru]\beta\text{-}5}$, $H_{[Ru]\beta\text{-}9}$, $H_{[Ru]\gamma\text{-}5}$, $H_{[Ru]\gamma\text{-}9}$), 6.95 (m, 12H, H_{209}), 6.95-6.94 (m, 336H, $H_{[Ru]\alpha\text{-}4}$, $H_{[Ru]\alpha\text{-}8}$, $H_{[Ru]\beta\text{-}4}$, $H_{[Ru]\beta\text{-}8}$, $H_{[Ru]\gamma\text{-}4}$, $H_{[Ru]\gamma\text{-}8}$), 6.74 (m, 30H, H_{126}, H_{207}), 6.64-6.62 (m, 24H, H_{12}, H_{19}, H_{107}), 6.53 (m, 12H, H_{125}), 2.70-2.65 (m, 144H, $H_{[Ru]\alpha\text{-}1}$, $H_{[Ru]\beta\text{-}1}$, $H_{[Ru]\gamma\text{-}1}$) ppm. ^{13}C NMR (151 MHz, $CDCl_3$) δ 137.4-137.1 (m, $C_{[Ru]\alpha\text{-}2}$, $C_{[Ru]\alpha\text{-}6}$, $C_{[Ru]\beta\text{-}2}$, $C_{[Ru]\beta\text{-}6}$, $C_{[Ru]\gamma\text{-}2}$, $C_{[Ru]\gamma\text{-}6}$), 134.5, 134.2 ($C_{[Ru]\alpha\text{-}3}$, $C_{[Ru]\alpha\text{-}7}$, $C_{[Ru]\beta\text{-}3}$, $C_{[Ru]\beta\text{-}7}$, $C_{[Ru]\gamma\text{-}3}$, $C_{[Ru]\gamma\text{-}7}$), 134.2, 132.3, 132.2 (C_{11}, C_{20}), 131.8, 131.7, 131.6, 131.5, 131.1, 131.0, 130.8, 130.2 (C_{125}, C_{126}, C_{207}), 130.1, 129.8, 128.8, 128.7 ($C_{[Ru]\alpha\text{-}5}$, $C_{[Ru]\alpha\text{-}9}$, $C_{[Ru]\beta\text{-}5}$, $C_{[Ru]\beta\text{-}9}$, $C_{[Ru]\gamma\text{-}5}$, $C_{[Ru]\gamma\text{-}9}$), 127.5 (C_{208}), 127.3, 127.1 ($C_{[Ru]\alpha\text{-}4}$, $C_{[Ru]\alpha\text{-}8}$,$C_{[Ru]\beta\text{-}4}$, $C_{[Ru]\beta\text{-}8}$, $C_{[Ru]\gamma\text{-}4}$, $C_{[Ru]\gamma\text{-}8}$), 124.2, 122.9 (C_{209}), 122.5, 121.1, 117.0, 116.4 (C_{14},C_{17}, C_{102}, C_{105}, C_{202}, C_{205}), 31.8 ($C_{[Ru]\alpha\text{-}1}$, $C_{[Ru]\beta\text{-}1}$, $C_{[Ru]\gamma\text{-}1}$) ppm. ^{31}P NMR (162 MHz, $CDCl_3$): δ 53.8 (s, 48P, $P_{[Ru]\gamma}$), 53.6 (s, 24P, $P_{[Ru]\beta}$), 53.4 (s, 12P, $P_{[Ru]\alpha}$) ppm. IR: *ν*(C≡C) 2052 cm^{-1}. UV-Vis (CH_2Cl_2, ν_{max} in cm^{-1}, [ε] in 10^3 M^{-1} cm^{-1}): 24 100 [489.0, sh], 29 150 [1179]. Anal. Calcd. for $C_{1548}H_{1218}P_{84}Ru_{21}$: C, 75.75; H, 5.00%; Found: C, 75.83; H, 4.84%.

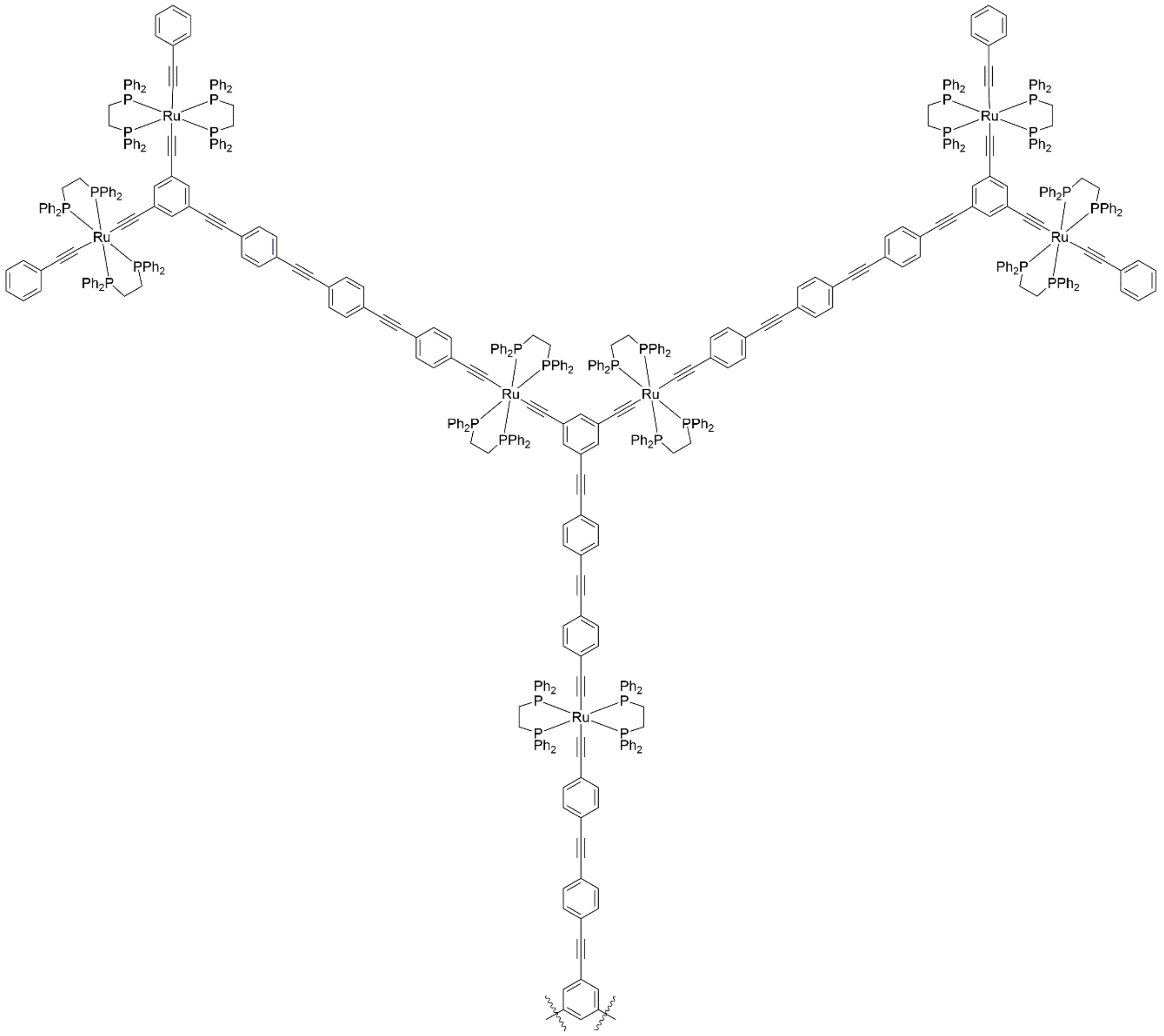

Synthesis of 1,3,5-{trans-[1,3-{trans-[1,3-{trans-[1,3-{trans-[(dppe)$_2$(PhC≡C)Ru(C≡C)]}$_2$C$_6$H$_3$-5-(C≡C-1,4-C$_6$H$_4$C≡C-1,4-C$_6$H$_4$C≡C)Ru(dppe)$_2$(C≡C)]}$_2$C$_6$H$_3$-5-(C≡C-1,4-C$_6$H$_4$C≡C-1,4-C$_6$H$_4$C≡C-1,4-C$_6$H$_4$C≡C)Ru(dppe)$_2$(C≡C)]}$_2$C$_6$H$_3$-5-(C≡C-1,4-C$_6$H$_4$C≡C-1,4-C$_6$H$_4$C≡C)Ru(dppe)$_2$(C≡C-1,4-C$_6$H$_4$C≡C-1,4-C$_6$H$_4$C≡C)]}$_3$C$_6$H$_3$ (3G$_{22,03,02,01}$). Compound **32** (0.030 g, 0.003 mmol) and **25** (0.136 g, 0.020 mmol, 6.6 eq.) were added to distilled, deoxygenated CH_2Cl_2 (60 mL), and NEt_3 (1 mL) was added to the solution. $NaPF_6$ (0.104 g, 0.62 mmol, 20 eq.) was added to the flask and the reaction mixture stirred at room temperature overnight. The crude product was obtained by passing the solution through a Celite pad and removing the solvent *in vacuo*. Further purification was conducted by sequential precipitation of a CH_2Cl_2 extract from stirring MeOH and *n*-pentane several times, to afford **3G$_{22,03,02,01}$** as a dark yellow powder (0.098 g, 0.002 mmol, 64%).

^{1}H NMR (400 MHz, $CDCl_3$): δ 7.65-7.45 (m, 720H, $H_{[Ru]\alpha\text{-}3}$, $H_{[Ru]\alpha\text{-}7}$, $H_{[Ru]\beta\text{-}3}$, $H_{[Ru]\beta\text{-}7}$, $H_{[Ru]\gamma\text{-}3}$, $H_{[Ru]\gamma\text{-}7}$, $H_{[Ru]\delta\text{-}3}$, $H_{[Ru]\delta\text{-}7}$), 7.36, 7.33-7.32 (m, 96H, H_{11}, H_{20}, H_{108}, H_{208}, H_{308}), 7.15-7.10 (m, 360H, $H_{[Ru]\alpha\text{-}5}$, $H_{[Ru]\alpha\text{-}9}$, $H_{[Ru]\beta\text{-}5}$, $H_{[Ru]\beta\text{-}9}$, $H_{[Ru]\gamma\text{-}5}$, $H_{[Ru]\gamma\text{-}9}$, $H_{[Ru]\delta\text{-}5}$, $H_{[Ru]\delta\text{-}9}$), 7.00 (m, 24H,

H_{309}), 6.95-6.94 (m, 720H, $H_{[Ru]\alpha-4}$, $H_{[Ru]\alpha-8}$, $H_{[Ru]\beta-4}$, $H_{[Ru]\beta-8}$, $H_{[Ru]\gamma-4}$, $H_{[Ru]\gamma-8}$, $H_{[Ru]\delta-4}$, $H_{[Ru]\delta-8}$), 6.79-6.53 (m, 147H, H_{12}, H_{19}, H_{31}, H_{32}, H_{125}, H_{126}, H_{207}, H_{219}, H_{220}, H_{307}), 2.70 (m, 360H, $H_{[Ru]\alpha-1}$, $H_{[Ru]\beta-1}$, $H_{[Ru]\gamma-1}$, $H_{[Ru]\delta-1}$) ppm. ^{13}C NMR (151 MHz, $CDCl_3$) δ 137.4-137.2 (m, $C_{[Ru]\alpha-2}$, $C_{[Ru]\alpha-6}$, $C_{[Ru]\beta-2}$, $C_{[Ru]\beta-6}$, $C_{[Ru]\gamma-2}$, $C_{[Ru]\gamma-6}$, $C_{[Ru]\delta-2}$, $C_{[Ru]\delta-6}$), 134.5, 134.3, 134.2 ($C_{[Ru]\alpha-3}$, $C_{[Ru]\alpha-7}$, $C_{[Ru]\beta-3}$, $C_{[Ru]\beta-7}$, $C_{[Ru]\gamma-3}$, $C_{[Ru]\gamma-7}$, $C_{[Ru]\delta-3}$, $C_{[Ru]\delta-7}$), 131.8, 131.5, 130.8, 130.2 (C_{125}, C_{126}, C_{207}), 129.8, 128.8, 128.6 ($C_{[Ru]\alpha-5}$, $C_{[Ru]\alpha-9}$, $C_{[Ru]\beta-5}$, $C_{[Ru]\beta-9}$, $C_{[Ru]\gamma-5}$, $C_{[Ru]\gamma-9}$, $C_{[Ru]\delta-5}$, $C_{[Ru]\delta-9}$), 127.5 (C_{308}), 127.3, 127.1 ($C_{[Ru]\alpha-4}$, $C_{[Ru]\alpha-8}$, $C_{[Ru]\beta-4}$, $C_{[Ru]\beta-8}$, $C_{[Ru]\gamma-4}$, $C_{[Ru]\gamma-8}$, $C_{[Ru]\delta-4}$, $C_{[Ru]\delta-8}$), 123.9, 123.4, 122.9 (C_{309}), 121.3, 117.0-116.4 (C_{14}, C_{17}, C_{102}, C_{105}, C_{202}, C_{205}), 31.8 ($C_{[Ru]\alpha-1}$, $C_{[Ru]\beta-1}$, $C_{[Ru]\gamma-1}$, $C_{[Ru]\delta-1}$) ppm. ^{31}P NMR (162 MHz, $CDCl_3$): δ 53.9 (s, 96P, $P_{[Ru]\delta}$), 53.6 (s, 48P, $P_{[Ru]\gamma}$), 53.4 (m, 36P, $P_{[Ru]\alpha}$, $P_{[Ru]\beta}$) ppm. IR: ν(C≡C) 2052 cm^{-1}. UV-Vis (CH_2Cl_2, ν_{max} in cm^{-1}, [ε] in 10^3 M^{-1} cm^{-1}): 23 900 [1057], 29 400 [2065]. Anal. Calcd. for $C_{3228}H_{2562}P_{180}Ru_{45}$: C, 75.32; H, 5.02%; Found: C, 75.32; H, 4.91%.

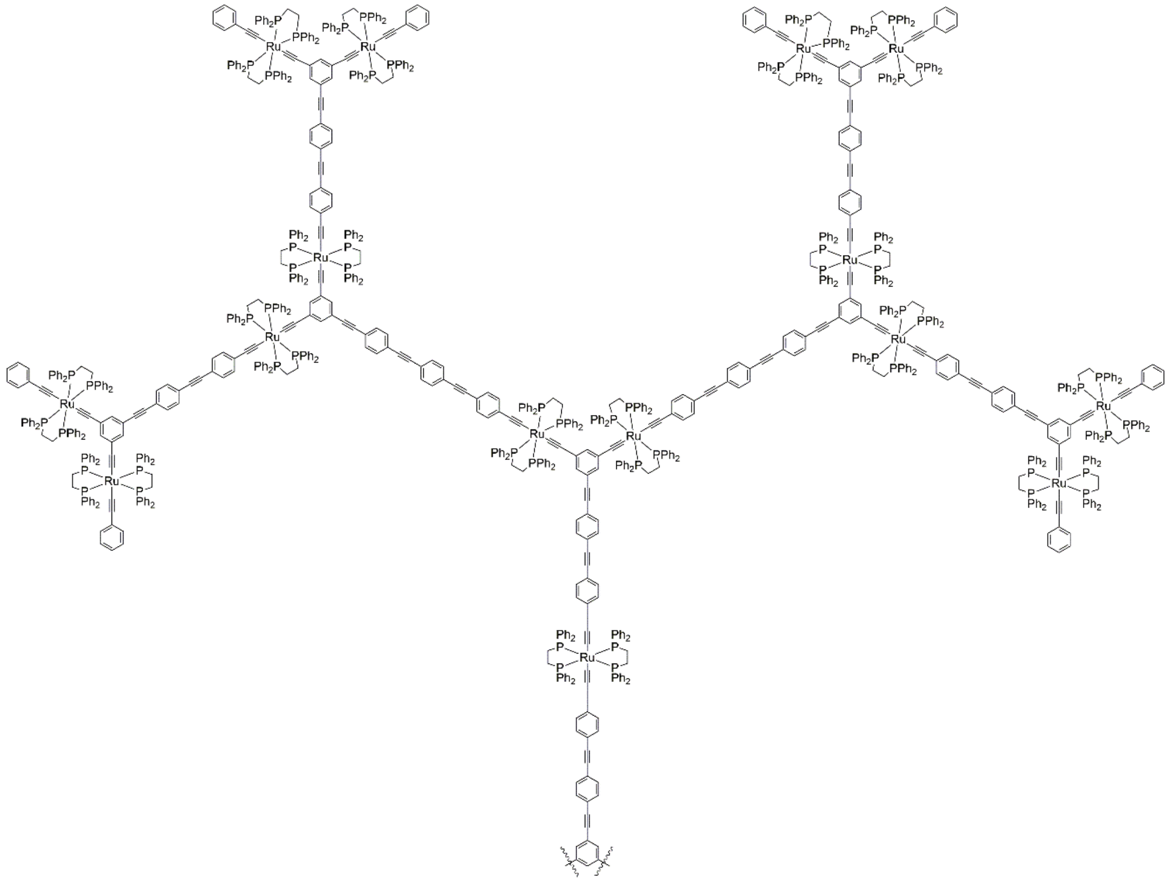

Synthesis of 1,3,5-{trans-[1,3-{trans-[1,3-{trans-[(dppe)$_2$(PhC≡C)Ru(C≡C)]}$_2$C$_6$H$_3$-5-(C≡C-1,4-C$_6$H$_4$C≡C-2,6-Et$_2$-1,4-C$_6$H$_2$C≡C-1,4-C$_6$H$_4$C≡C)Ru(dppe)$_2$(C≡C)]}$_2$C$_6$H$_3$-5-(C≡C-1,4-C$_6$H$_4$C≡C-1,4-C$_6$H$_4$C≡C)Ru(dppe)$_2$(C≡C-1,4-C$_6$H$_4$C≡C-1,4-C$_6$H$_4$C≡C)]}$_3$C$_6$H$_3$ (2G$_{22,03,01}$-s). Compound **35** (0.100 g, 0.008 mmol) and 1,3-{*trans*-[(dppe)$_2$(PhC≡C)Ru(C≡C)]}-5-(HC≡C)C_6H_3 (0.103 g, 0.052 mmol, 6.1 eq.) were added to distilled, deoxygenated CH_2Cl_2 (50 mL) and NEt_3 (10 mL). The solution was degassed and

backfilled with nitrogen three times. Catalytic amounts of $Pd(PPh_3)_4$ and $[Cu(NCMe)_4]PF_6$ were added to the solution. [To avoid homo-coupling the byproduct from the Sonogashira reaction, which is exceptionally difficult to separate from the product due to the similar structure and solubility, $[Cu(NCMe)_4]PF_6$ was selected as the copper(I) source in the catalytic cycle owing to its ease of access and purification and its long shelf-life time in the dark, compared to other common copper(I) sources.] The reaction mixture was stirred at room temperature for three days. The crude product was obtained by pouring a CH_2Cl_2 extract into stirring MeOH and collected by filtration with a sintered funnel. Further purification was conducted by precipitation of a CH_2Cl_2 extract from sequentially MeOH (50 mL) and then Et_2O (25 mL), and washing several times with *n*-pentane, to afford **$2G_{22,03,01}$-s** as a yellow powder (0.116 g, 0.005 mmol, 62%).

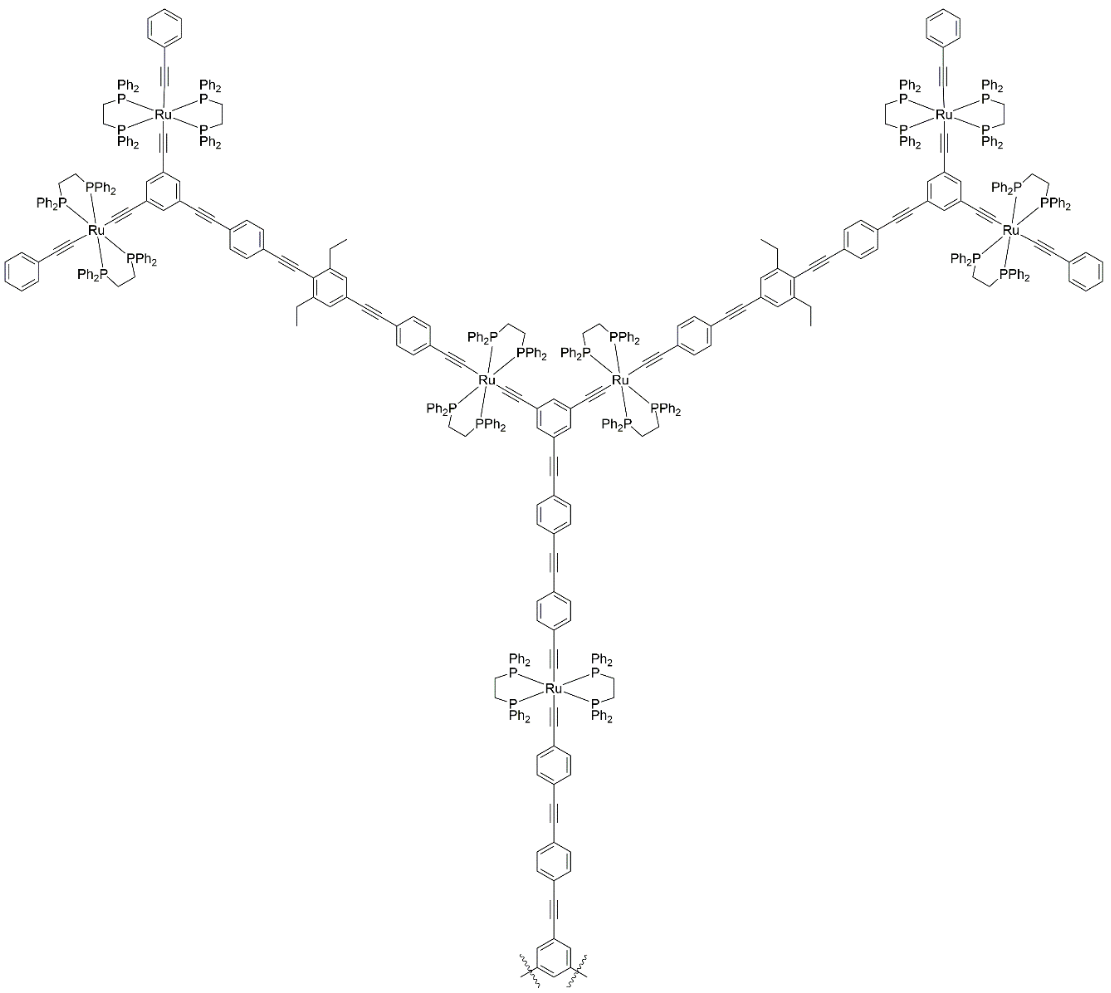

^{1}H NMR (600 MHz, $CDCl_3$) δ 7.72 (m, 3H, H_0), 7.66-7.37 (m, 336H, $H_{[Ru]\alpha\text{-}3}$, $H_{[Ru]\alpha\text{-}7}$, $H_{[Ru]\beta\text{-}3}$, $H_{[Ru]\beta\text{-}7}$, $H_{[Ru]\gamma\text{-}3}$, $H_{[Ru]\gamma\text{-}7}$), 7.30 (m, 36H, H_{11}, H_{20}, H_{208}), 7.16-7.10 (m, 168H, $H_{[Ru]\alpha\text{-}5}$, $H_{[Ru]\alpha\text{-}9}$, $H_{[Ru]\beta\text{-}5}$, $H_{[Ru]\beta\text{-}9}$, $H_{[Ru]\beta\text{-}9}$, $H_{[Ru]\gamma\text{-}5}$, $H_{[Ru]\gamma\text{-}9}$), 7.01 (m, 12H, H_{209}), 6.96-6.95 (m, 336H, $H_{[Ru]\alpha\text{-}4}$, $H_{[Ru]\alpha\text{-}8}$, $H_{[Ru]\beta\text{-}4}$, $H_{[Ru]\beta\text{-}8}$, $H_{[Ru]\gamma\text{-}4}$, $H_{[Ru]\gamma\text{-}8}$), 6.79-6.63 (m, 54H, H_{12}, H_{19}, H_{107}, H_{126}, H_{207}), 6.53 (m, 12H, H_{125}), 2.95 (m, 24H, H_{s1}), 2.70 (m, 168H, $H_{[Ru]\alpha\text{-}1}$, $H_{[Ru]\beta\text{-}1}$, $H_{[Ru]\gamma\text{-}1}$), 1.36 (m, 36H, H_{s2}) ppm. ^{13}C NMR (151 MHz, $CDCl_3$) δ 146.6 (C_{114}), 137.4-137.1 (m, $C_{[Ru]\alpha\text{-}2}$, $C_{[Ru]\alpha\text{-}6}$, $C_{[Ru]\beta\text{-}2}$, $C_{[Ru]\beta\text{-}6}$, $C_{[Ru]\gamma\text{-}2}$, $C_{[Ru]\gamma\text{-}6}$) 134.5, 134.3, 134.2 ($C_{[Ru]\alpha\text{-}3}$, $C_{[Ru]\alpha\text{-}7}$, $C_{[Ru]\beta\text{-}3}$,

$C_{[Ru]β-7}$, $C_{[Ru]γ-3}$, $C_{[Ru]γ-7}$), 133.0, 131.8, 130.8 (C_{11}, C_{20}), 130.2, 129.8 (C_{125}, C_{126}, C_{207}), 128.9, 128.8 ($C_{[Ru]α-5}$, $C_{[Ru]α-9}$, $C_{[Ru]β-5}$, $C_{[Ru]β-9}$, $C_{[Ru]γ-5}$, $C_{[Ru]γ-9}$), 128.7, 128.5, 127.5 (C_{208}), 127.3, 27.2 ($C_{[Ru]α-4}$, $C_{[Ru]α-8}$, $C_{[Ru]β-4}$, $C_{[Ru]β-8}$, $C_{[Ru]γ-4}$, $C_{[Ru]γ-8}$), 122.9 (C_{209}), 117.0, 116.4 (C_{14}, C_{17}, C_{102}, C_{105}, C_{202}, C_{205}), 31.8 ($C_{[Ru]α-1}$, $C_{[Ru]β-1}$, $C_{[Ru]γ-1}$) 28.2 (C_{s1}), 14.8 (C_{s2}) ppm. ^{31}P NMR (162 MHz, $CDCl_3$): δ 53.9 (s, 48P, $P_{[Ru]γ}$), 53.6 (s, 24P, $P_{[Ru]β}$), 53.4 (s, 12P, $P_{[Ru]α}$) ppm. IR: *ν*(C≡C) 2055 cm^{-1}. UV-Vis (CH_2Cl_2, $ν_{max}$ in cm^{-1}, [ε] in 10^3 M^{-1} cm^{-1}): 23 550 [543.2, sh], 29 150 [1218]. Anal. Calcd. for $C_{1548}H_{1218}P_{84}Ru_{21}$: C, 75.75; H, 5.00%. Found: C, 75.46; H, 5.13%.

Synthesis of 1,3,5-{trans-[1,3-{trans-[1,3-{trans-[1,3-{trans-[(dppe)$_2$(PhC≡C)Ru(C≡C)]}$_2$C$_6$H$_3$-5-(C≡C-1,4-C$_6$H$_4$C≡C-1,4-C$_6$H$_4$C≡C)Ru(dppe)$_2$(C≡C)]}$_2$C$_6$H$_3$-5-(C≡C-1,4-C$_6$H$_4$C≡C-2,6-Et$_2$-1,4-C$_6$H$_2$C≡C-1,4-C$_6$H$_4$C≡C)Ru(dppe)$_2$(C≡C)]}$_2$C$_6$H$_3$-5-(C≡C-1,4-C$_6$H$_4$C≡C-1,4-C$_6$H$_4$C≡C)Ru(dppe)$_2$(C≡C-1,4-C$_6$H$_4$C≡C-1,4-C$_6$H$_4$C≡C)]}$_3$C$_6$H$_3$ (3G$_{22,03,02,01}$-s). Compound **37** (0.090 g, 0.004 mmol) and **13** (0.114 g, 0.049 mmol, 13.0 eq.) were added to distilled CH_2Cl_2 (70 mL) and NEt_3 (1 mL). The solution was degassed and backfilled with nitrogen three times. $NaPF_6$ (0.038 g, 0.224 mmol, 60.0 eq.) was added to the flask and the reaction mixture was stirred at room temperature overnight. The crude product was obtained by passing the solution through a Celite pad and removing the solvent *in vacuo*. Further purification was conducted following precipitation of a CH_2Cl_2 extract from stirring MeOH and *n*-pentane several times, to afford a yellow powder identified as compound **3G$_{22,03,02,01}$-s** (0.171 g, 0.003 mmol, 88%).

^1H NMR (600 MHz, $CDCl_3$) δ 7.66-7.36 (m, 720H, $H_{[Ru]α-3}$, $H_{[Ru]α-7}$, $H_{[Ru]β-3}$, $H_{[Ru]β-7}$, $H_{[Ru]γ-3}$, $H_{[Ru]γ-7}$, $H_{[Ru]δ-3}$, $H_{[Ru]δ-7}$), 7.34-7.29 (m, 96H, H_{11}, H_{20}, H_{108}, H_{208}, H_{308}), 7.16-7.11 (m, 360H, $H_{[Ru]α-5}$, $H_{[Ru]α-9}$, $H_{[Ru]β-5}$, $H_{[Ru]β-9}$, $H_{[Ru]β-9}$, $H_{[Ru]γ-5}$, $H_{[Ru]γ-9}$, $H_{[Ru]δ-5}$, $H_{[Ru]δ-9}$), 7.01 (m, 24H, H_{309}), 7.00, 6.96-6.95 (m, 720H, $H_{[Ru]α-4}$, $H_{[Ru]α-8}$, $H_{[Ru]β-4}$, $H_{[Ru]β-8}$, $H_{[Ru]γ-4}$, $H_{[Ru]γ-8}$, $H_{[Ru]δ-4}$, $H_{[Ru]δ-8}$), 6.80-6.53 (m, 147H, H_{12}, H_{19}, H_{31}, H_{32}, H_{125}, H_{126}, H_{207}, H_{219}, H_{220}, H_{307}), 2.92 (m, 24H, H_{s1}), 2.70 (m, 168H, $H_{[Ru]α-1}$, $H_{[Ru]β-1}$, $H_{[Ru]γ-1}$, $H_{[Ru]δ-1}$), 1.35 (m, 36H, H_{s2}) ppm. ^{13}C NMR (176 MHz, $CDCl_3$) δ 146.8 (C_{114}), 137.4-137.2 (m, $C_{[Ru]α-2}$, $C_{[Ru]α-6}$, $C_{[Ru]β-2}$, $C_{[Ru]β-6}$, $C_{[Ru]γ-2}$, $C_{[Ru]γ-6}$, $C_{[Ru]δ-2}$, $C_{[Ru]δ-6}$), 134.5, 134.3, 134.2 ($C_{[Ru]α-3}$, $C_{[Ru]α-7}$, $C_{[Ru]β-3}$, $C_{[Ru]β-7}$, $C_{[Ru]γ-3}$, $C_{[Ru]γ-7}$, $C_{[Ru]δ-3}$, $C_{[Ru]δ-7}$), 131.8, 131.5, 131.0, 130.8, 130.2, 129.8, 128.8, 128.7 ($C_{[Ru]α-5}$, $C_{[Ru]α-9}$, $C_{[Ru]β-5}$, $C_{[Ru]β-9}$, $C_{[Ru]γ-5}$, $C_{[Ru]γ-9}$, $C_{[Ru]δ-5}$, $C_{[Ru]δ-9}$), 127.49 (C_{308}), 127.3, 127.2 ($C_{[Ru]α-4}$, $C_{[Ru]α-8}$, $C_{[Ru]β-4}$, $C_{[Ru]β-8}$, $C_{[Ru]γ-4}$, $C_{[Ru]γ-8}$, $C_{[Ru]δ-4}$, $C_{[Ru]δ-8}$), 122.9 (C_{309}), 117.0, 116.4, 31.8 (m, $C_{[Ru]α-1}$, $C_{[Ru]β-1}$, $C_{[Ru]γ-1}$, $C_{[Ru]δ-1}$), 28.1 (C_{s1}), 14.8 (C_{s2}) ppm. ^{31}P NMR (162 MHz, $CDCl_3$): δ 53.9 (s, 96P, $P_{[Ru]δ}$), 53.6, 53.4 (m, 84P, $P_{[Ru]α}$, $P_{[Ru]β}$, $P_{[Ru]γ}$) ppm. IR: *ν*(C≡C) 2052 cm $^{-1}$. UV-Vis (CH_2Cl_2, $ν_{max}$ in cm^{-1}, [ε] in 10^3 M^{-1} cm^{-1}): 28 900 [2219]. Anal. Calcd. for $C_{3252}H_{2610}P_{180}Ru_{45}$: C, 75.38; H, 5.08%. Found: C, 75.33; H, 5.16%.

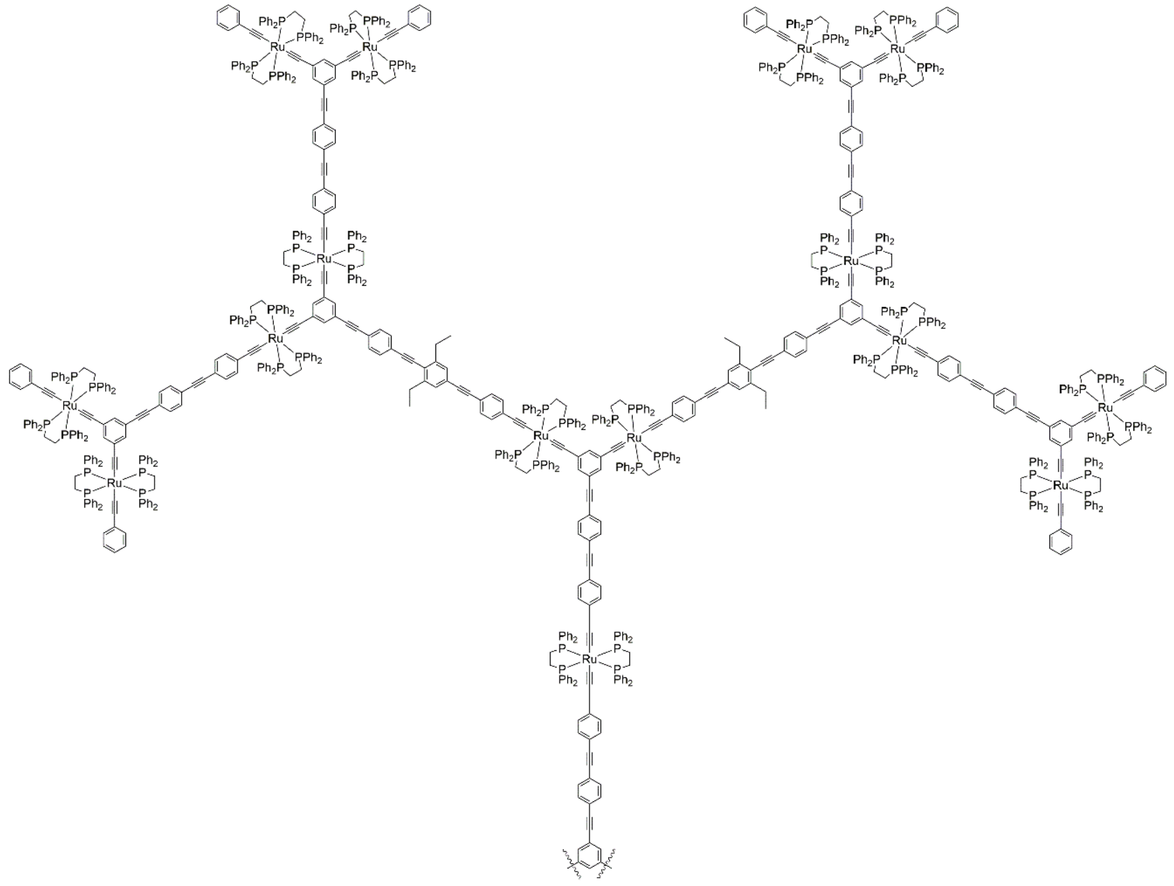

Instrumentation

NMR spectra were recorded using Bruker Avance 400 MHz, 600 MHz, 700 MHz and 800 MHz Bruker NMR spectrometers and are referenced to residual $CHCl_3$ (^{1}H, 7.26 ppm) or $CDCl_3$ (^{13}C, 77.16 ppm) or external 85% H_3PO_4 (^{31}P, 0.0 ppm). Infrared spectra were recorded by using a PerkinElmer Spectrum One FT-IR spectrometer using ATR analysis. The TEM micrographs were recorded using a TEM JEOL 2100F in STEM mode at the Centre for Advanced Microscopy at the Australian National University. The TEM samples were placed on lacey carbon films on 200 mesh copper grids. UV-Vis spectra were recorded in CH_2Cl_2 in 1 cm quartz cells using a PerkinElmer Lambda 950 spectrophotometer and are reported as λ_{max} nm (ε 10^3 M^{-1} cm^{-1}). TOF MS ESI were recorded using a Waters LCT Premier mass spectrometer. Solutions in methanol or acetonitrile were ionized with sodium ions; peaks are reported as *m/z* (assignment, relative intensity). Microanalyses were carried out at the London Metropolitan University.

Size-exclusion chromatography (SEC). The molecular weight distributions of the samples were determined using a high-performance gel liquid chromatography Viscotek GPC Max VE2001 fitted with a Viscotek TDA 305 triple detector array consisting of a differential viscometer, right-angle laser-light scattering, low-angle laser-light scattering and refractive index detectors. The column set consisted of a Viscotek TGuard Organic Guard Column (10 ×

4.6 mm) and two Viscotek LT5000L Mixed Medium Organic Columns (300 × 7.8 mm, 300 × 8.0 mm). The system was fitted with an online solvent deoxygenater system and eluent (THF) flow rate was set to 1 mL min^{-1} and columns were held at 30 °C. Calibration was carried out using PolyCAL™ poly(methyl methacrylate) standards with M_p = 800, 2380, 5050, 9680, 18700, 41400, 88500, 202000, 340000, 608000, and 988000 using OmniSEC software version 4.6.1.354. Unless otherwise stated, injection volumes of 100 μL were employed.

Diffusion-ordered spectroscopy. All DOSY experiments were performed on a Bruker Avance 600 MHz NMR instrument with Cryoprobe. The standard Bruker pulse sequence **dstebpgp3s**, employing a double stimulated echo sequence, bipolar gradient pulsed for diffusion and three spoil gradients, was chosen to minimize the convection effect. The gradient amplitudes were set from 2 to 95 % in 32 linear steps. The gradient duration and the diffusion time were optimized for each sample and were in the ranges 0. 5-2.5 ms and 100-200 ms, respectively. 4 Dummy scans were used for temperature equilibration in the sample. The recycle delay was adjusted for each sample by measuring T_1 values, using the standard inversion recovery method, for all the resonances of interest and setting the recycle delay to 5 times the T_1 value of the most slowly relaxing resonance of interest. Signal intensities *I* were measured by integration. Plots of $ln(I/I_0)$ *vs.* mG^2 were fitted using a standard linear regression algorithm implemented in the Bruker TopSpin 3.5 pl 7 with T_1/T_2 module. The diffusion coefficients of three different molecules (methanol, phenol, and anthracene) were measured in $CDCl_3$ solution for comparison to literature values.[9] At 298.0 ± 0.1 K, the diffusion coefficients of the three compounds were found to be: methanol, $D = 3.310 \times 10^{-9}$ m^2 s^{-1}; phenol, $D = 2.016 \times 10^{-9}$ m^2 s^{-1}; anthracene, $D = 1.742 \times 10^{-9}$ m^2 s^{-1}, which match well with the reported values.[9]

Due to the temperature dependence of the diffusion coefficients, as well as the solvent viscosity, the actual sample temperature inside the probe must be calibrated before experiments, following the standard procedure in the Bruker manual. Neat methanol samples were used to calibrate the actual temperatures. Results showed that the temperature was maintained at a constant 298.0 ± 0.1 K after 20 min in the NMR bore.

The gradient strength was calibrated using literature values for water diffusion in HDO/D_2O obtained from Aldrich (D_2O % = 99.96%).[10]

For NMR diffusion measurements, the samples were placed into 5 mm NMR tubes to a height of 40 mm. The magnetic susceptibility of the sample tube is closely matched to solution samples, thereby obtaining good magnetic homogeneity over the whole sample volume while keeping good signal-to-noise ratio in the ^1H NMR spectrum. The concentration of samples was ~10^{-4} M in $CDCl_3$ solution at room temperature, to fit the assumption that the solution viscosity can be approximated to that of pure $CDCl_3$.

Cubic NLO and MPA studies. The real and imaginary parts of the second hyperpolarizability γ (γ_{Re} and γ_{Im}) were determined using the Z-scan technique. An amplified femtosecond laser system consisting of an Integra-C regenerative amplifier (Quantronix) operating as an 800 nm pump and a Palitra-FS BIBO crystal-based optical parametric amplifier (Quantronix) was used. The system was tunable over a wavelength range from 650-2520 nm, delivering 130 fs pulses

at a 1 kHz repetition rate. The output wavelength was confirmed using an Ocean Optics USB2000+ spectrometer (650-1000 nm) or an Ocean Optics NIR-Quest spectrometer (1000-1700 nm). Coloured glass filters and a Thorlabs polarizing filter were used to remove unwanted wavelengths, and the power was adjusted by use of neutral density filters to obtain nonlinear phase shifts between 0.2 and 1.3 rad. The focal length of the lens used in the experiment was 75 mm, which gave 25-50 μm beam waists resulting in Rayleigh lengths longer than that of the sample thickness. Measurements were made in 1 mm optical cells, such that the total thickness was $\leqslant$ 3 mm including the glass walls, so results could safely be treated using the thin-sample approximation. Samples travelled down the Z-axis on a Thorlabs motorized stage between -20 and +20 mm (where 0 was the laser focus). Data were collected by three Thorlabs photodiodes, 650-1100 nm with Si-based detectors, 1100-1700 nm with InGaAs detectors, and 1700-2520 nm with amplified InGaAs detectors. Data from the detectors were collected by a Tektronix oscilloscope with a custom LabVIEW program and curve-fitted with theoretical traces computed using equations derived by Sheik-Bahae et al.[11] All measurements were calibrated against closed-aperture Z-scans of the solvent, as well as those of a 3 mm thick silica plate. The real and imaginary components of the second hyperpolarizability (γ) of the materials were calculated assuming additivity of the contributions of the solvent and the solute. The negative γ_{real} maximal values approximately coincide with the positive maximal values of γ_{imag}, and therefore consistent with the expected dependence of γ_{real} on all nonlinear absorption processes through a nonlinear Kramers-Kronig relationship. Samples were prepared with the concentration around 0.3 w/w% in deoxygenated and distilled CH_2Cl_2. Overall, the fitting of data was consistent with 2PA from 650-1000 nm, 3PA from 1050 to 1550 nm, 4PA from 1600 to 1950 nm, and 5PA from 2000 to 2520 nm, although fits at the extrema of these ranges did not necessarily fit the corresponding ideal multiphoton curve, which may suggest that multiple absorptive processes occur at these wavelengths.

Multi-photon absorption cross-sections were calculated using [12, 13]:

$$\sigma_{nPA} = \frac{\alpha_n(\hbar\omega)^{n-1}}{N_0}$$

where n = 2, 3, 4, 5, and 6, and α_n is the appropriate nonlinear absorption coefficient, while N_0 is Avogadro's number.

Calculation of the "Number of Effective *π*-Electrons"

Following the procedure developed by Kuzyk[14, 15] for organic molecules and extended by Schwich[16] to organometallic compounds, we associate two electrons with each triple bond along the conjugation path. Because some of the *π*-electrons of the central phenyl ring are delocalised into each bridge, two of the six electrons are assigned to each bridge. Ruthenium centres are considered as "disrupting" units that separate conjugated moieties from each other. The N_{eff} can be calculated[14, 17] using:

$$N_{eff} = \sqrt{\sum_{i}^{n} N_i^2}$$

affording the following results:

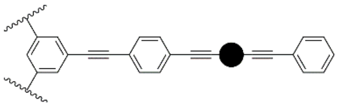

$0G_{11}$ $N_{eff} = 25.0$

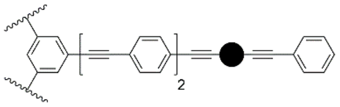

$0G_{21}$ $N_{eff} = 37.3$

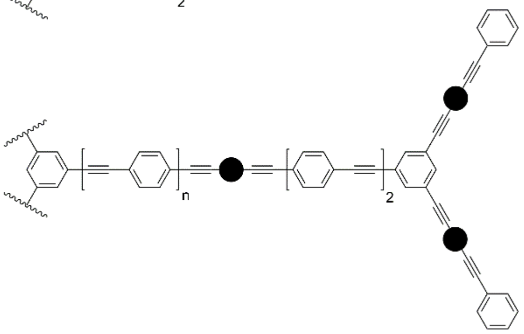

n = 1, $1G_{12,01}$ $N_{eff} = 46.0$

n = 2, $1G_{22,01}$ $N_{eff} = 53.6$

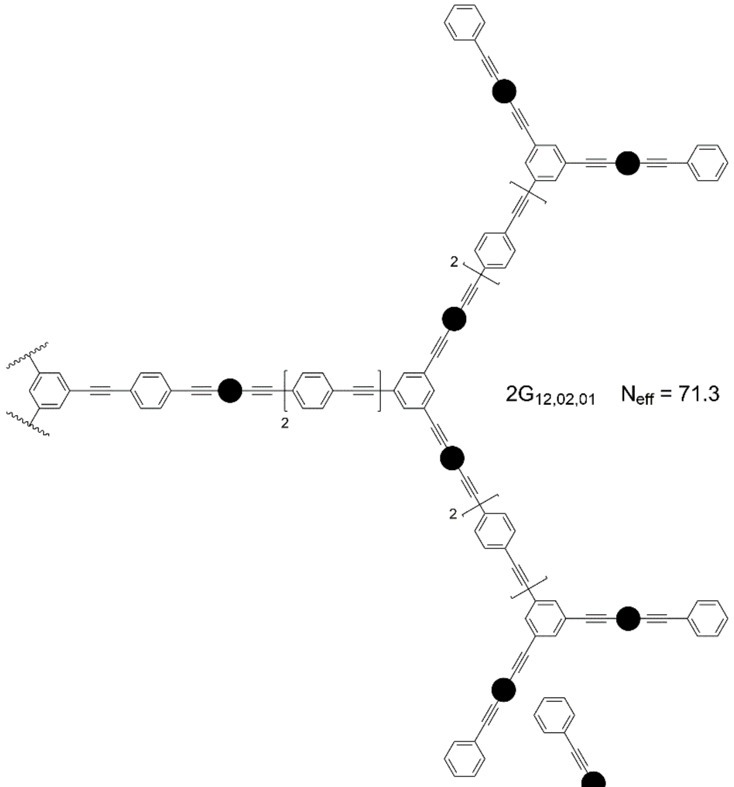

$2G_{12,02,01}$ N_{eff} = 71.3

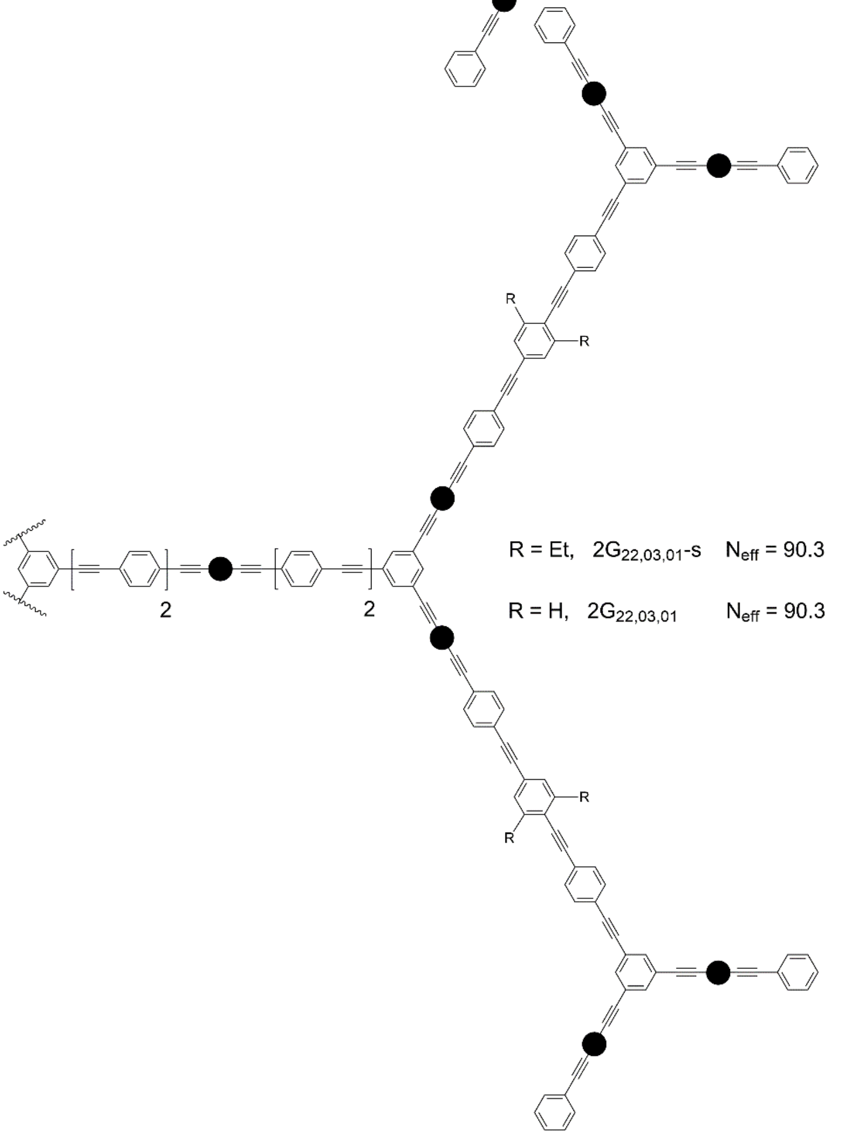

R = Et, $2G_{22,03,01}$-s N_{eff} = 90.3

R = H, $2G_{22,03,01}$ N_{eff} = 90.3

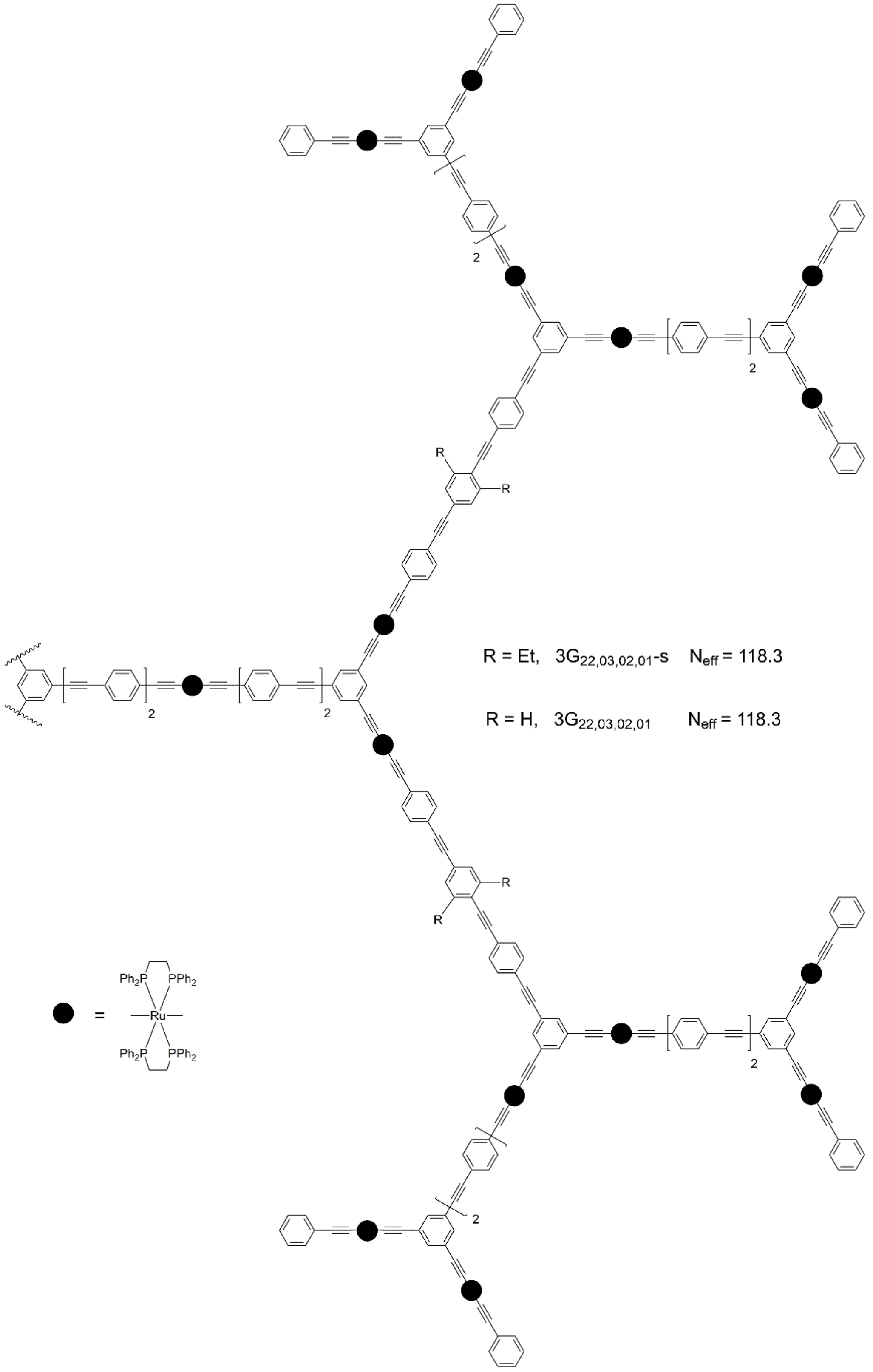

Scheme S1. Effective π-electrons in the organometallic compounds in this study.

NMR Spectra of New Compounds

Proton and carbon labelling of the spectral data for the new compounds started from the arene core, as shown in Scheme S2, and within each generation (e.g., from the core to the first-generation branching point as generation zero, from the first-generation branching point to the next branching point as generation one) atoms were labelled followed the dendrimer's extension direction and the label contains generation number followed by the number of the atom. The atoms in the ethyl groups were labelled as "s1" and "s2" to distinguish them from the dendrimer skeleton atoms. The atoms in the silyl protecting groups were labelled as "p1" and "p2". The Ru(κ^2-dppe)$_2$ units were abbreviated as "[Ru]" with subscript of generation number (α, β, γ, δ). For each ruthenium centre, the labelling started from carbons on the ethylene group through all the carbons on the aromatic rings. All the protons attached to the same carbon atom that are chemically equivalent share the same number. Peaks in the ^{13}C spectra were assigned with the assistance of 2D spectra H-H COSY, H-C HSQC and H-C HMBC, and were labelled as "C_{xx}" (xx represents the atom's number). Proton peaks were labelled as "H_{xx}" in the same manner.

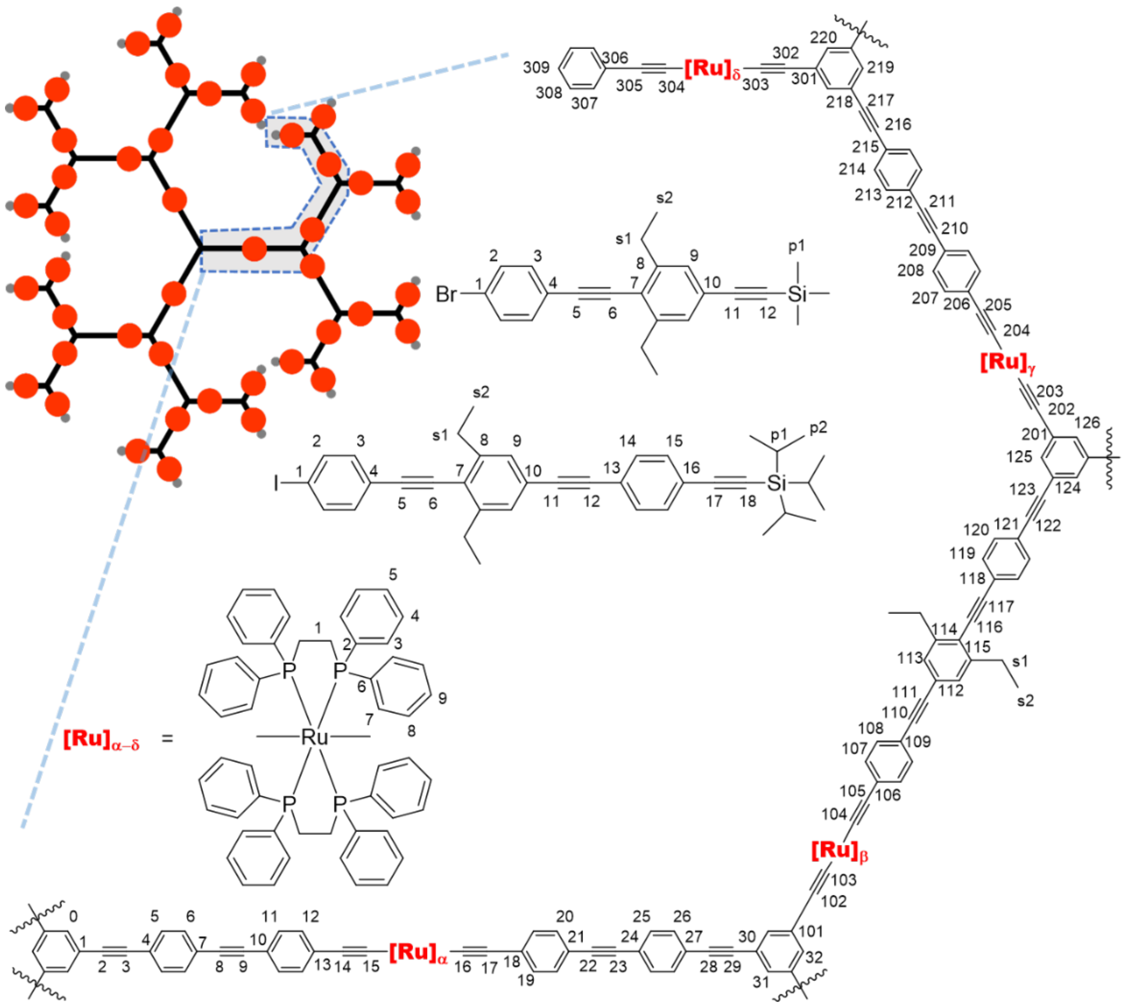

Scheme S2. ^{1}H and ^{13}C NMR labelling for new compounds.

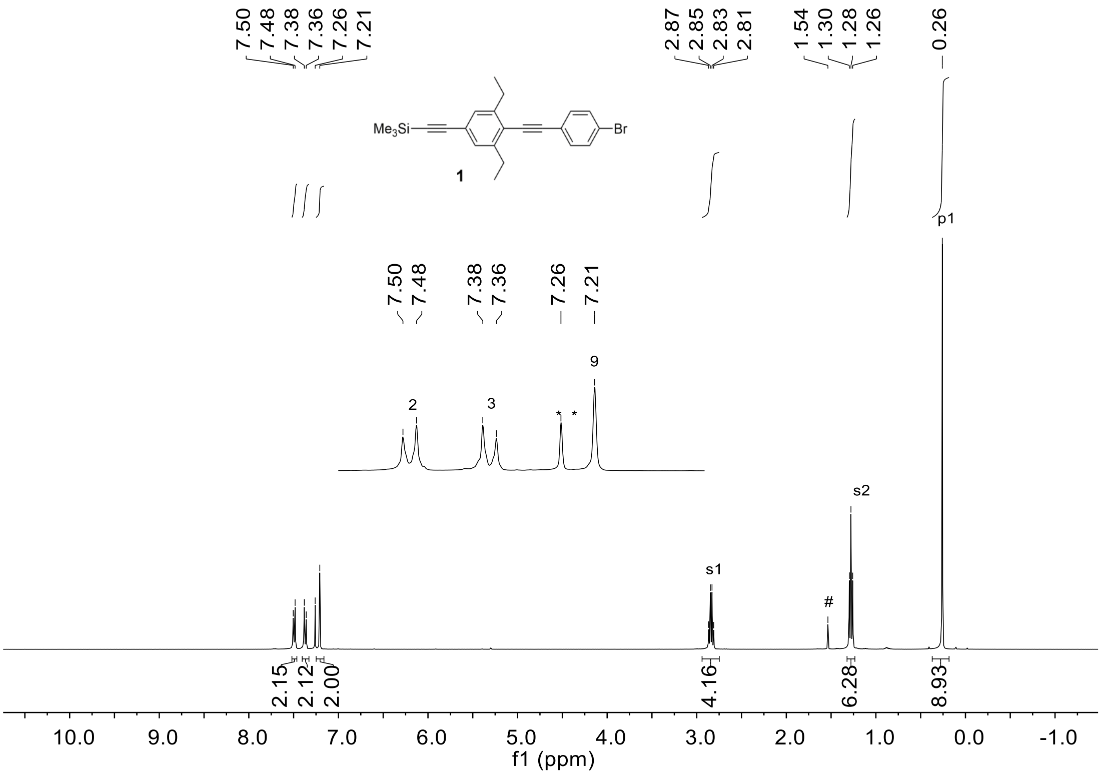

Figure S1. ¹H NMR spectrum of **1**. The peak marked as * * corresponds to the residual $CHCl_3$ signal. The peak marked as # corresponds to the residual water signal.

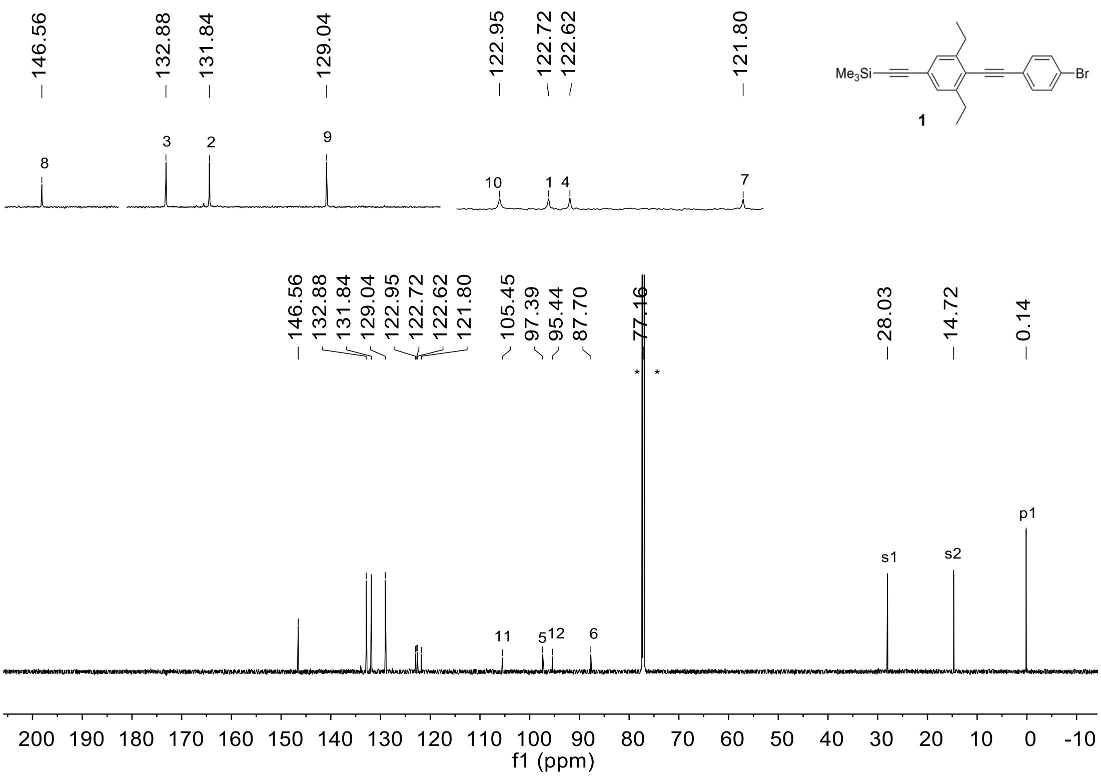

Figure S2. ¹³C NMR spectrum of **1**. The peak marked as * * corresponds to $CDCl_3$.

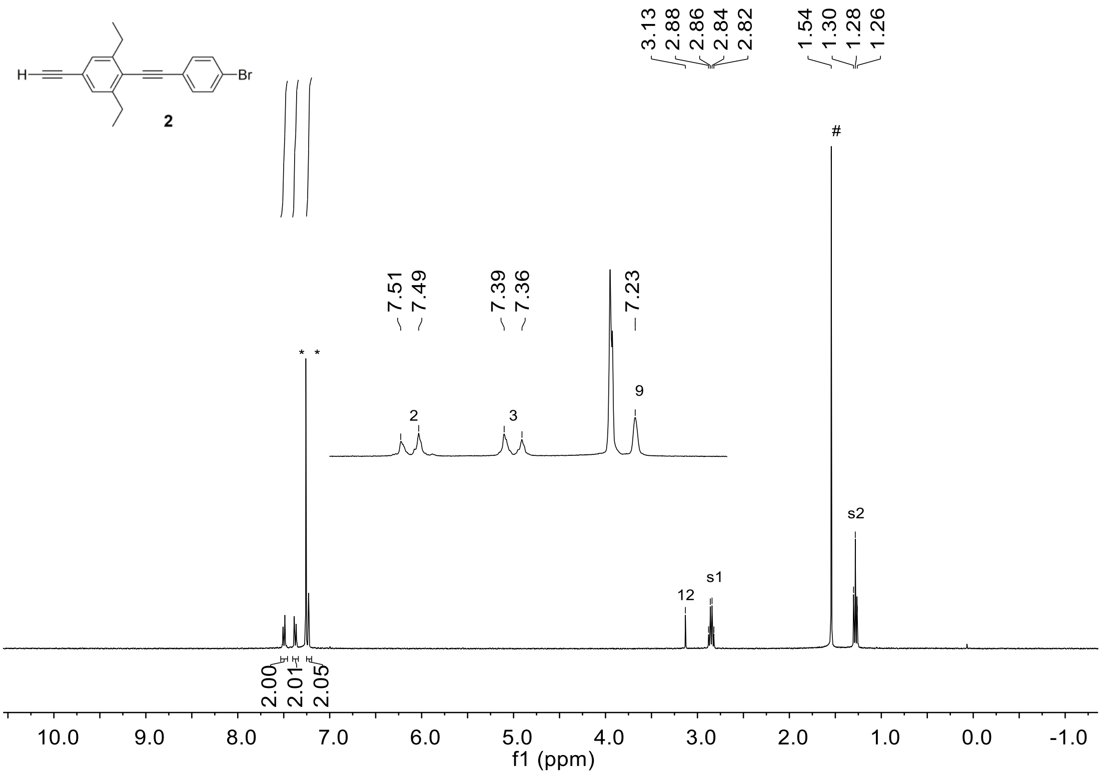

Figure S3. ^{1}H NMR spectrum of **2**. The peak marked as * * corresponds to the residual $CHCl_3$ signal. The peak marked as # corresponds to the residual water signal.

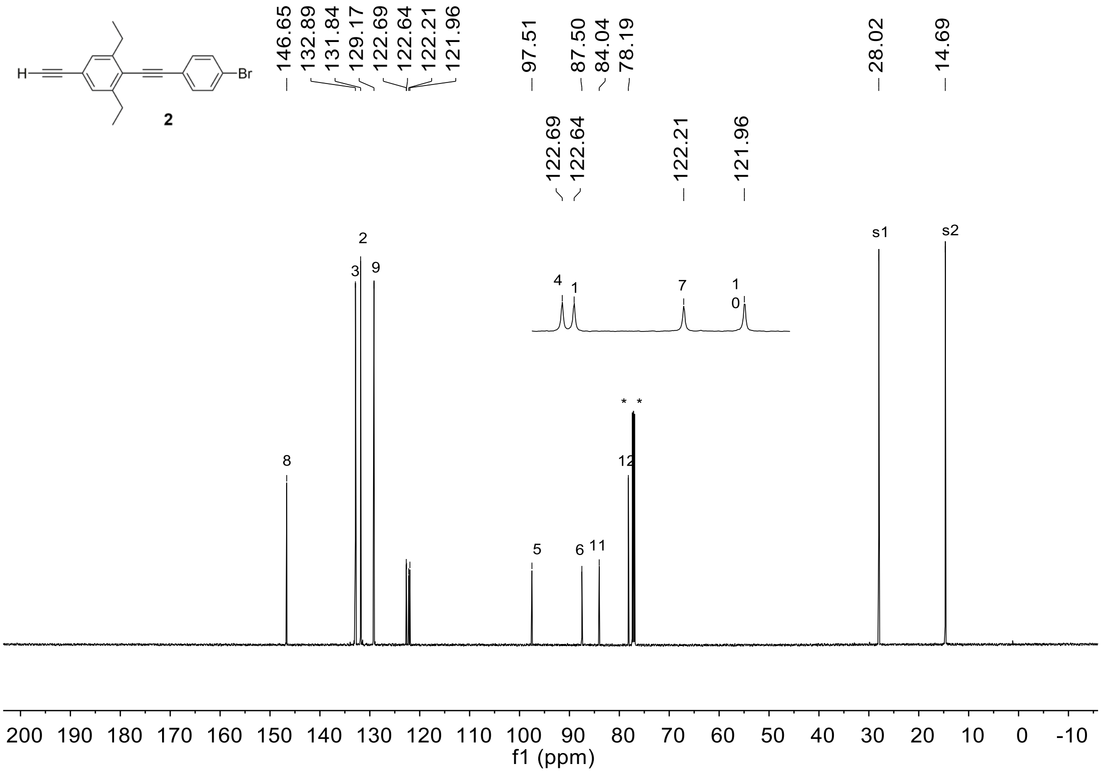

Figure S4. ^{13}C NMR spectrum of **2**. The peak marked as * * corresponds to $CDCl_3$.

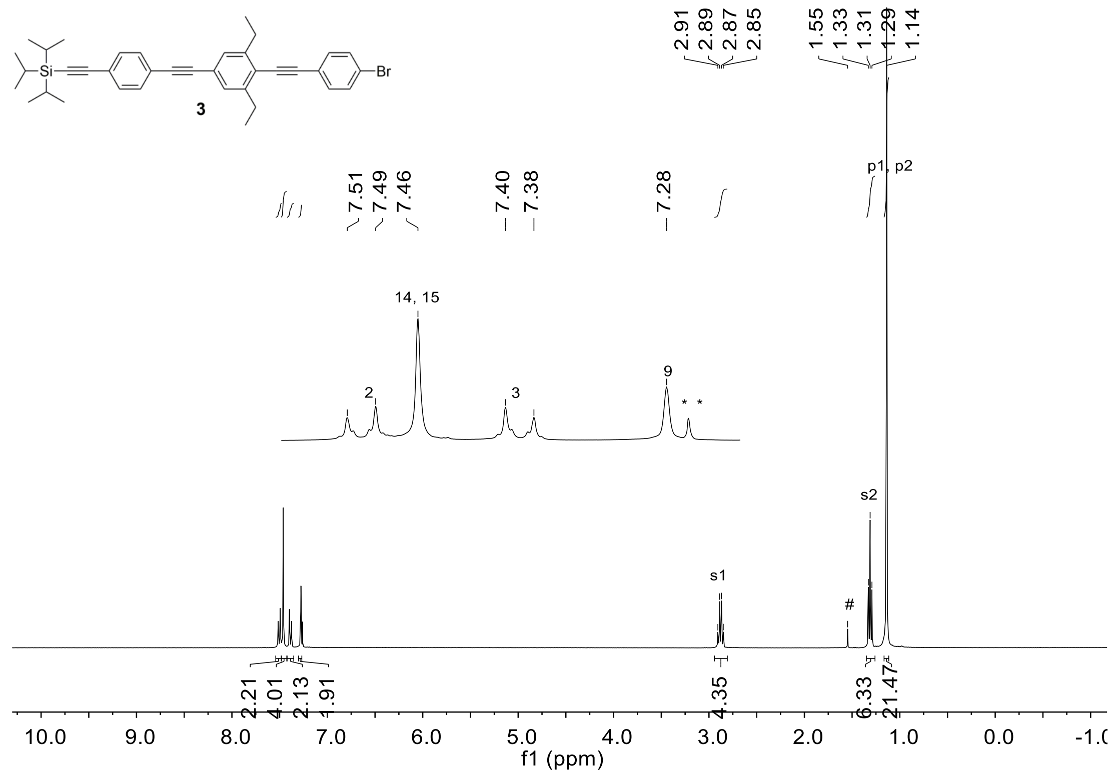

Figure S5. ^{1}H NMR spectrum of **3**. The peak marked as * * corresponds to the residual $CHCl_3$ signal. The peak marked as # corresponds to the residual water signal.

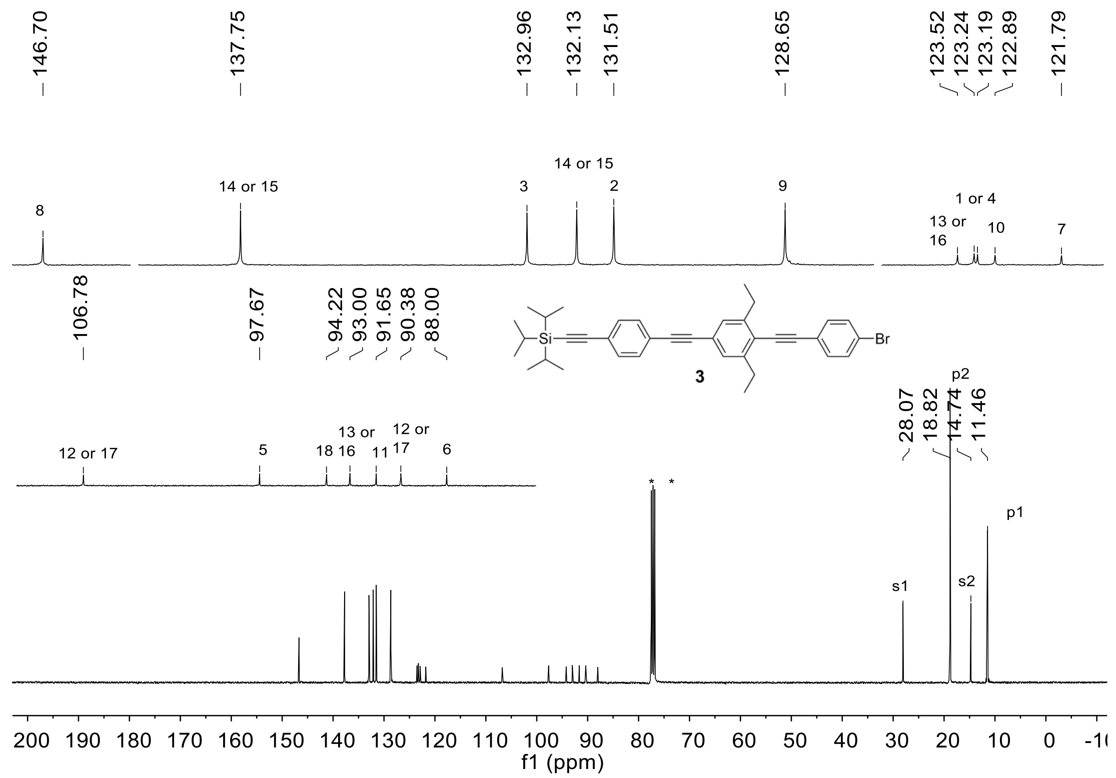

Figure S6. ^{13}C NMR spectrum of **3**. The peak marked as * * corresponds to $CDCl_3$.

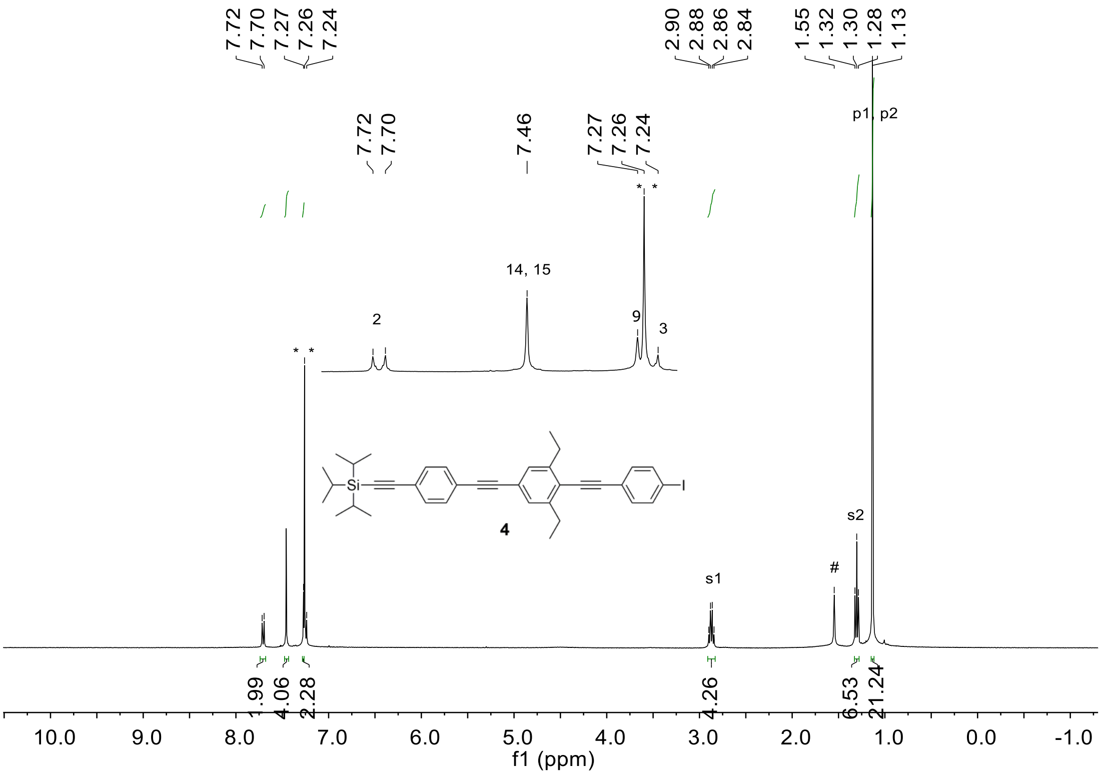

Figure S7. ^{1}H NMR spectrum of **4**. The peak marked as * * corresponds to the residual $CHCl_3$ signal. The peak marked as # corresponds to the residual water signal.

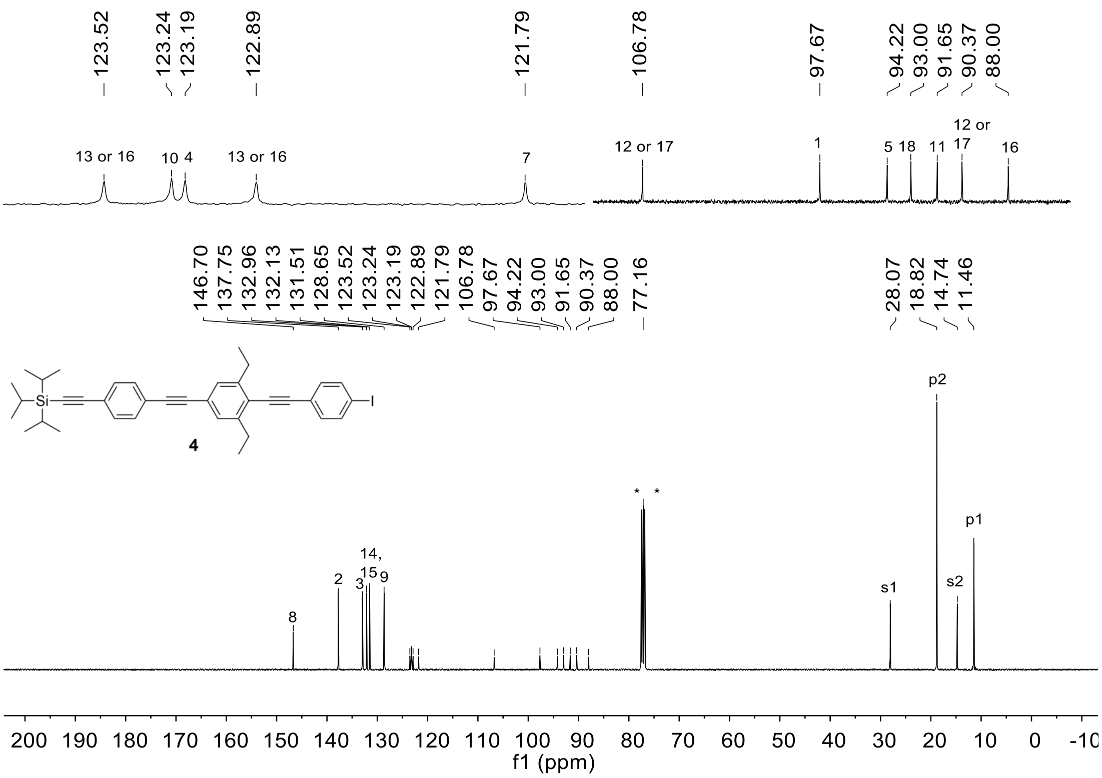

Figure S8. ^{13}C NMR spectrum of **4**. The peak marked as * * corresponds to $CDCl_3$.

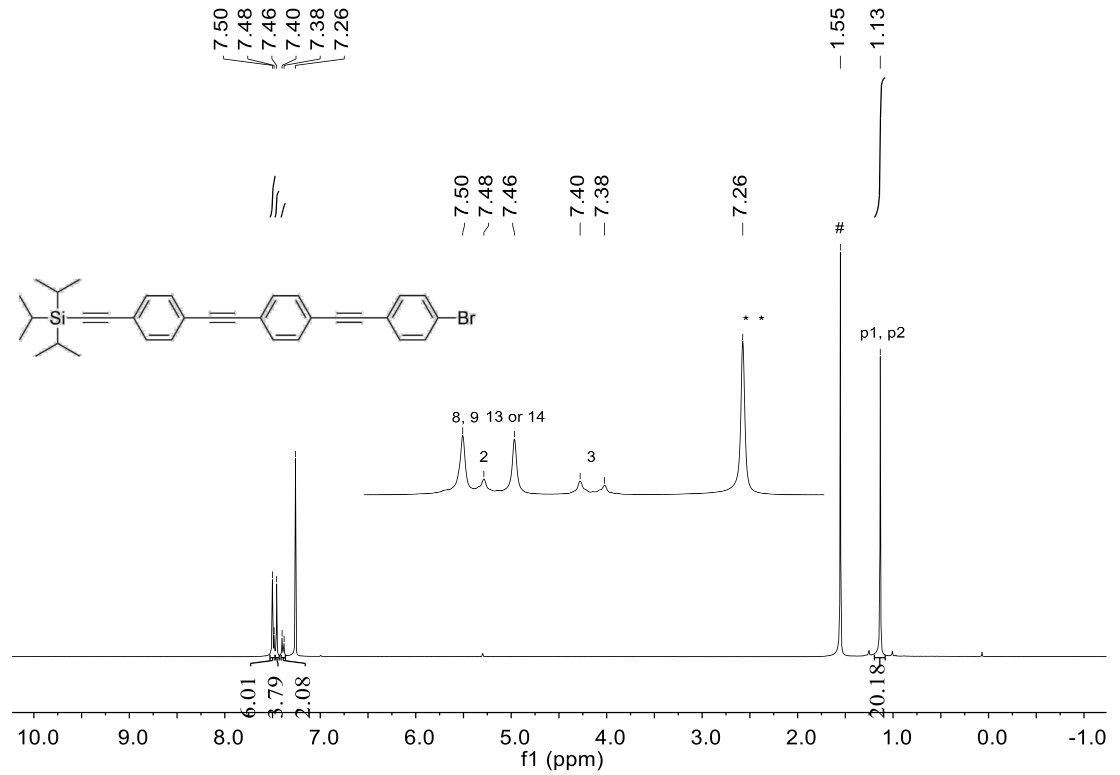

Figure S9. ^{1}H NMR spectrum of **7**. The peak marked as * * corresponds to the residual $CHCl_3$ signal. The peak marked as # corresponds to the residual water signal.

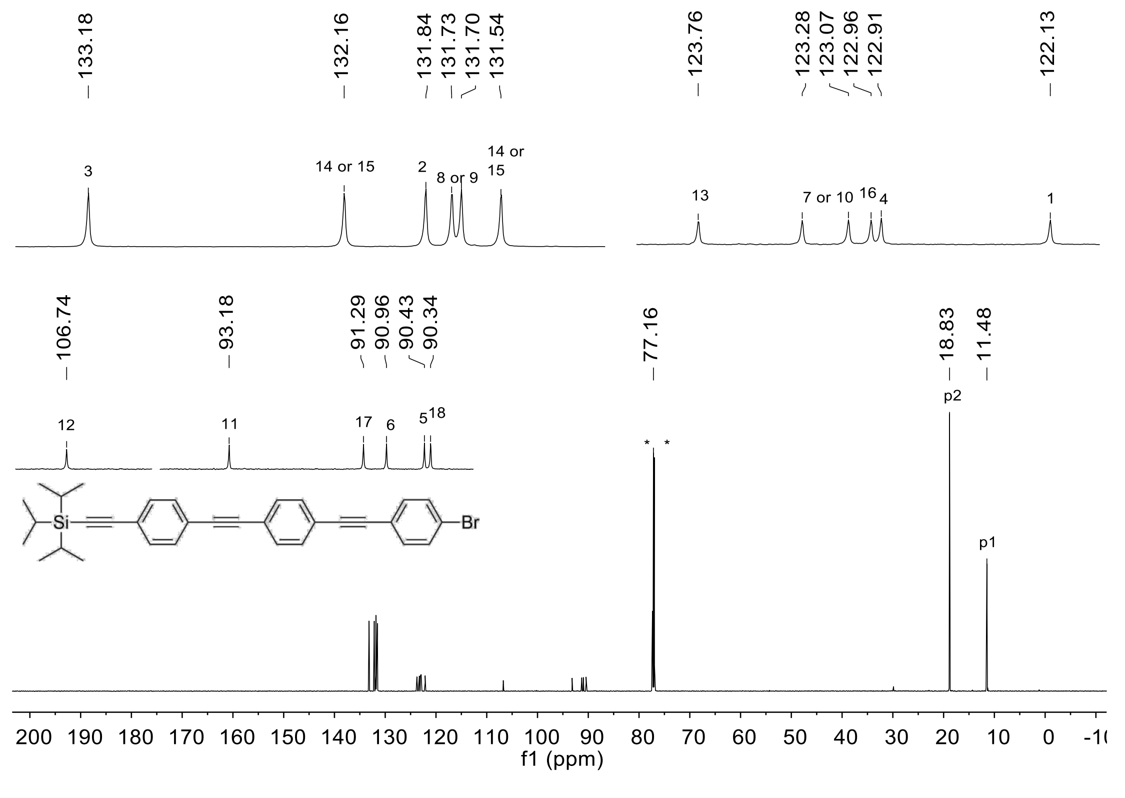

Figure S10. ^{13}C NMR spectrum of **7**. The peak marked as * * corresponds to $CDCl_3$.

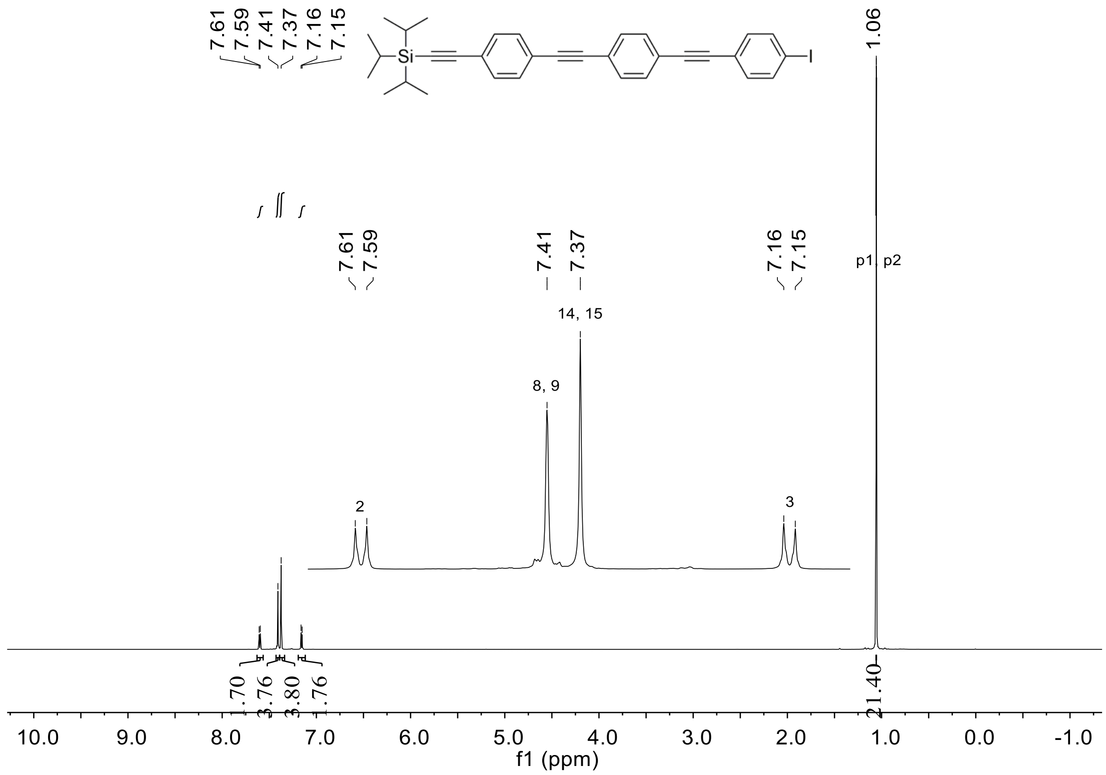

Figure S11. ^{1}H NMR spectrum of **8** in $CDCl_3$.

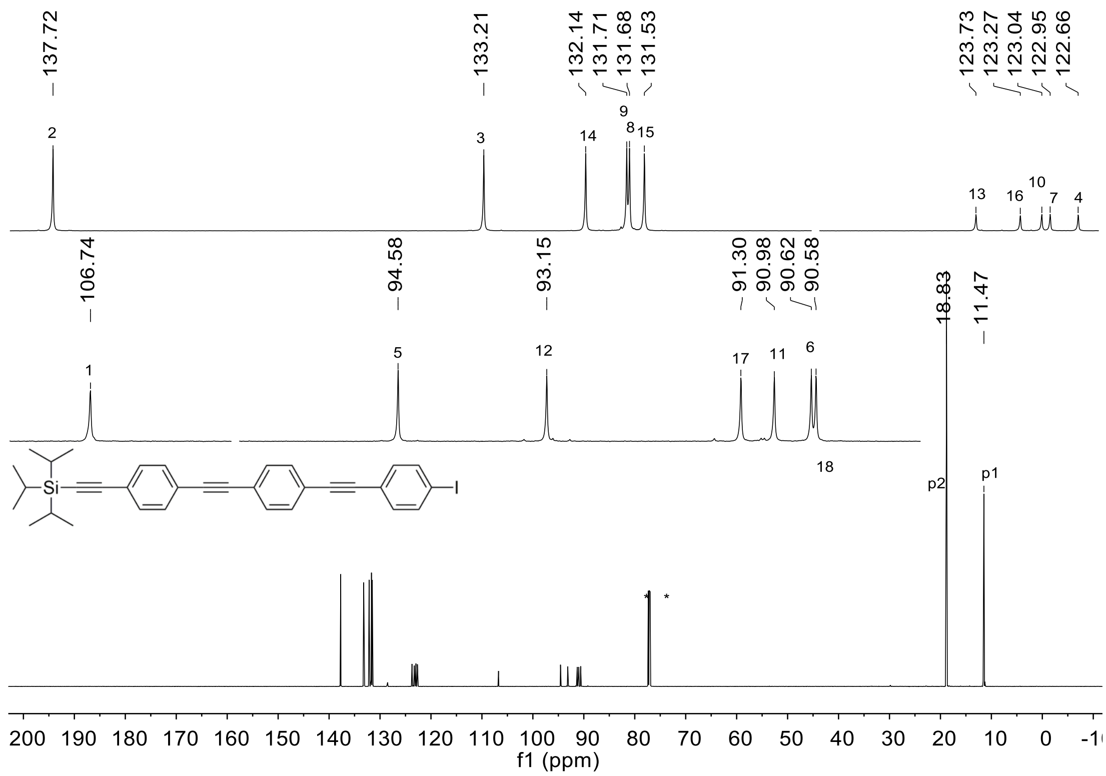

Figure S12. ^{13}C NMR spectrum of **8**. The peak marked as * * corresponds to $CDCl_3$.

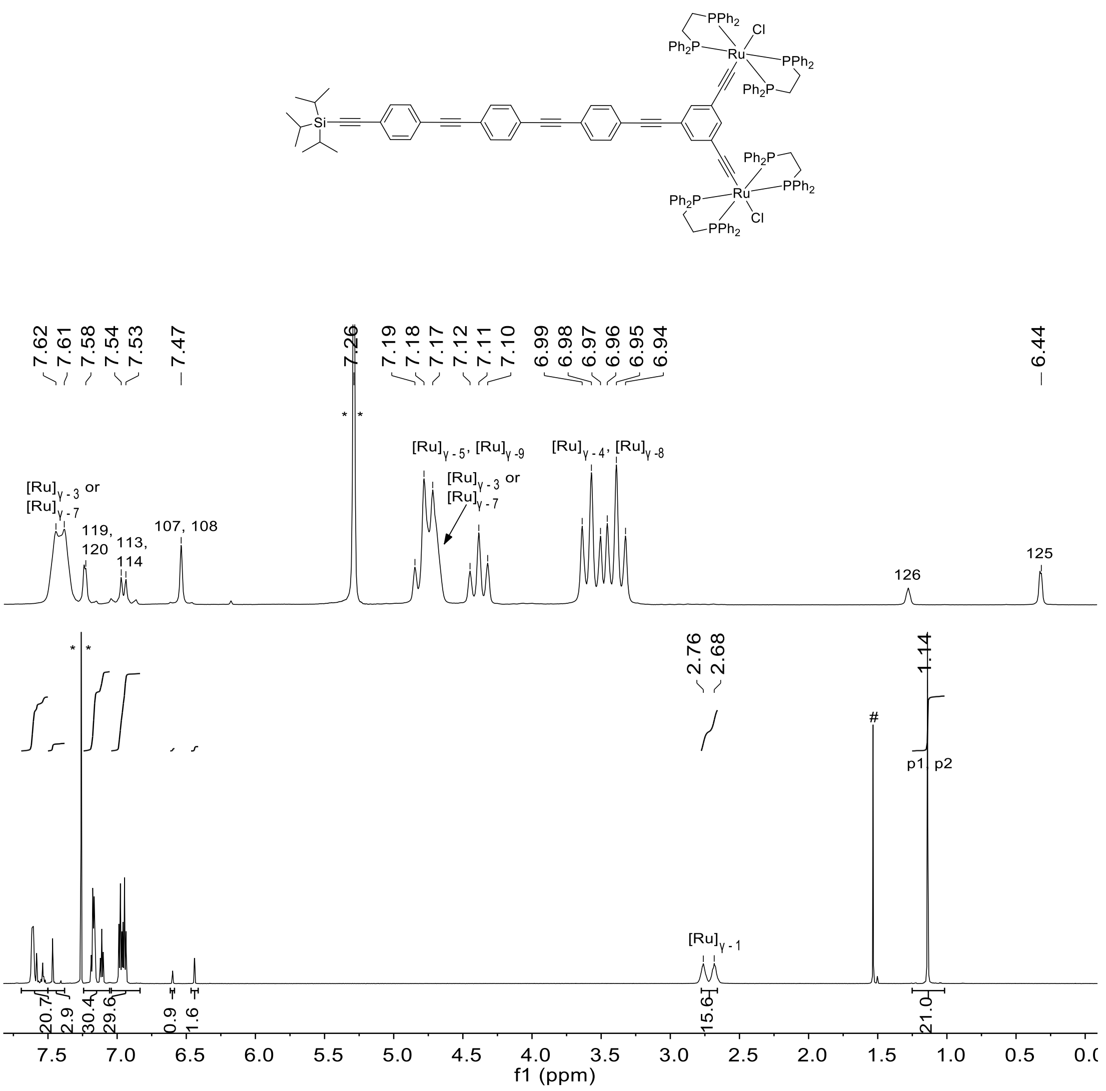

Figure S13. ^{1}H NMR spectrum of **16**. The peak marked as * * corresponds to the residual $CHCl_3$ signal. The peak marked as # corresponds to the residual water signal.

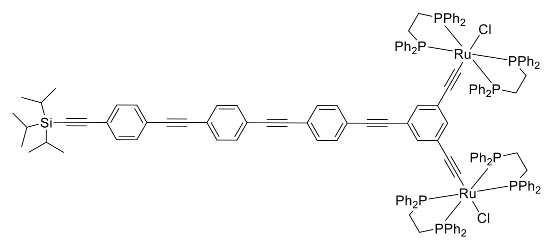

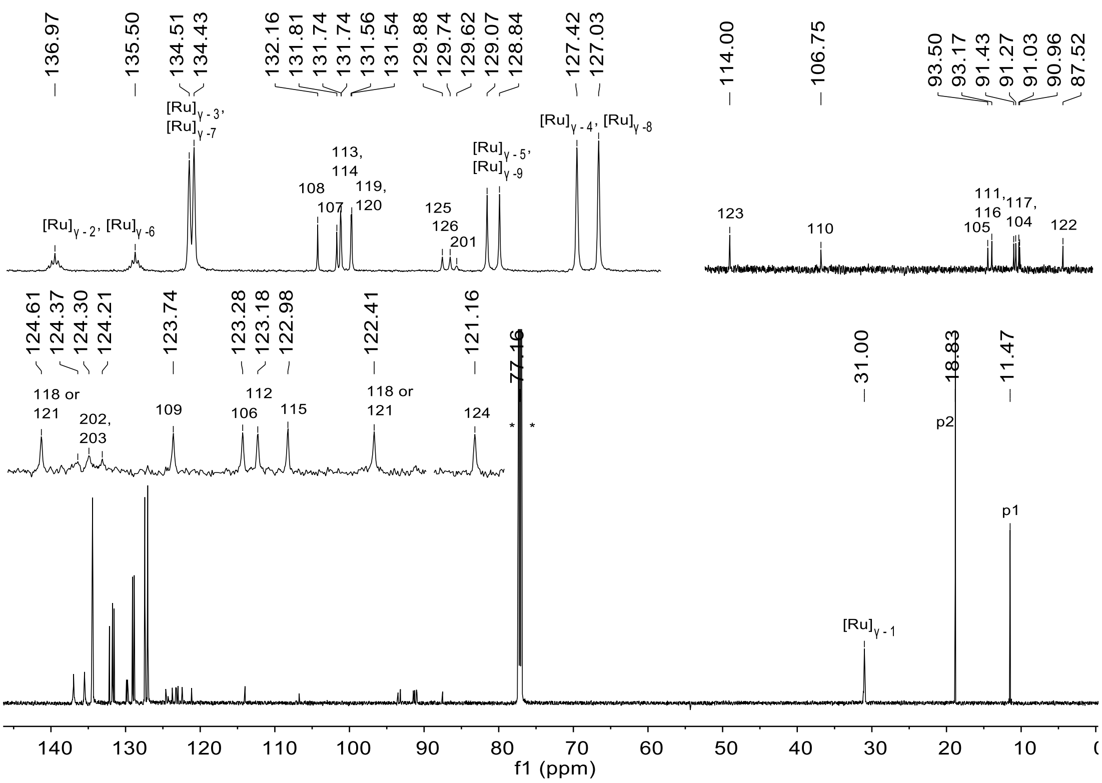

Figure S14. ^{13}C NMR spectrum of **16**. The peak marked as * * corresponds to $CDCl_3$.

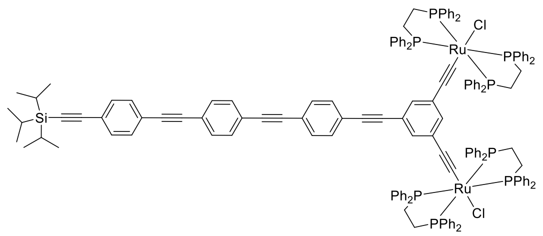

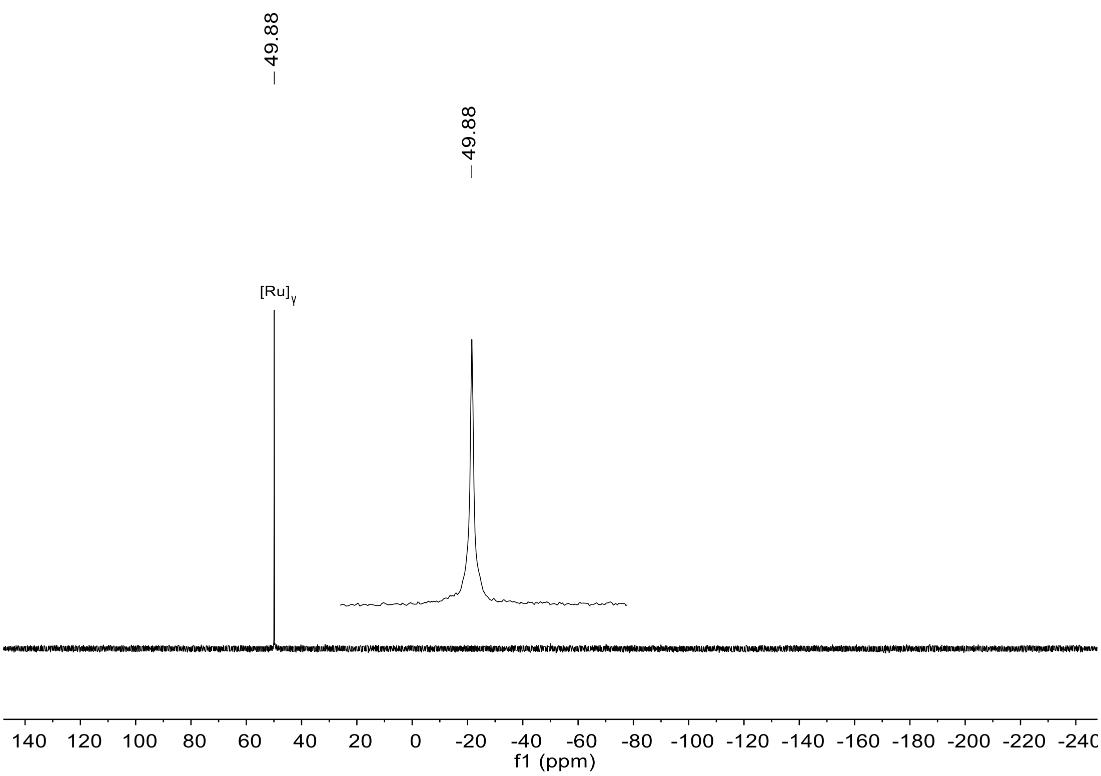

Figure S15. ^{31}P NMR spectrum of **16**.

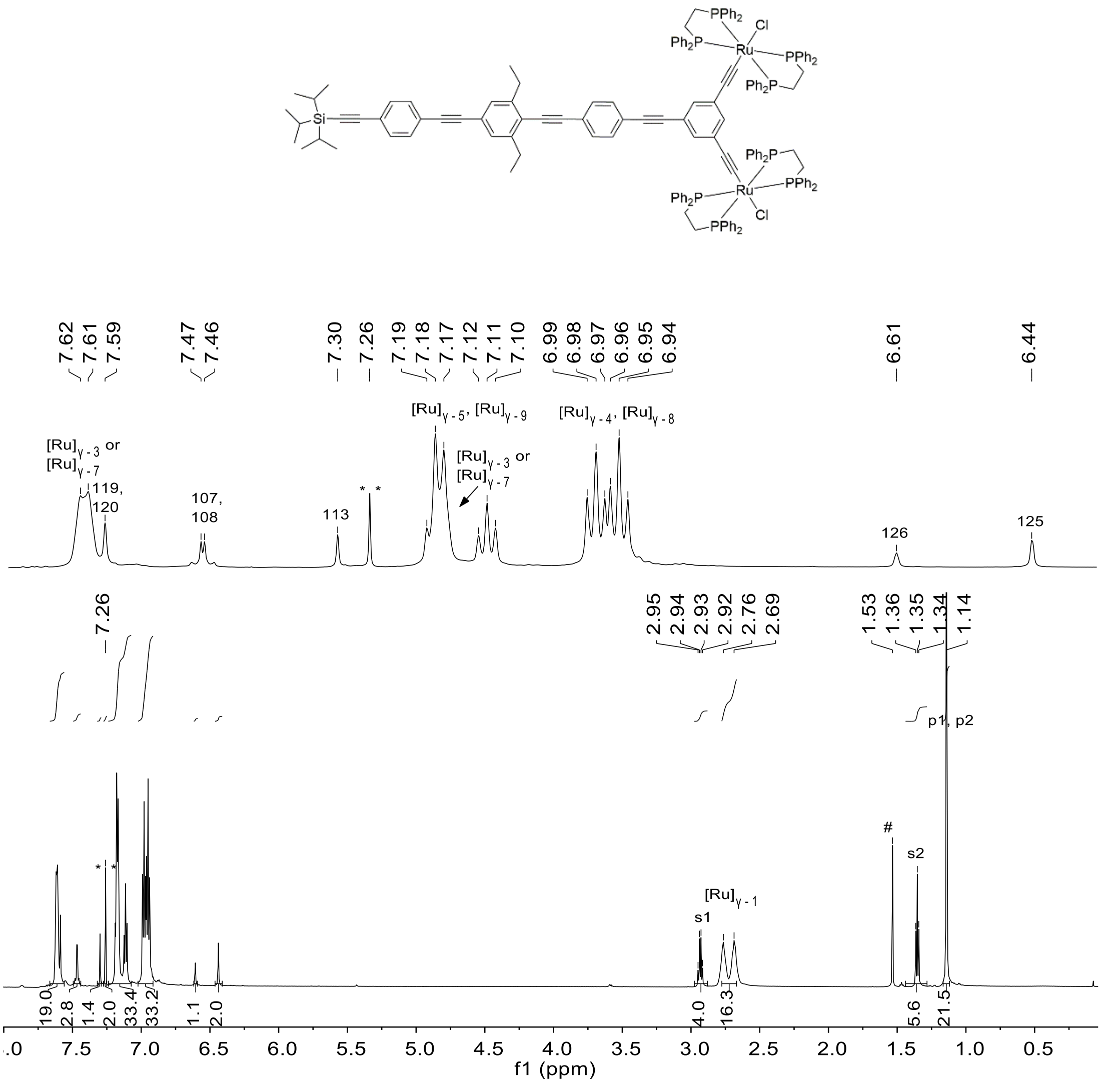

Figure S16. ^{1}H NMR spectrum of **17**. The peak marked as * * corresponds to the residual $CHCl_3$ signal. The peak marked as # corresponds to the residual water signal.

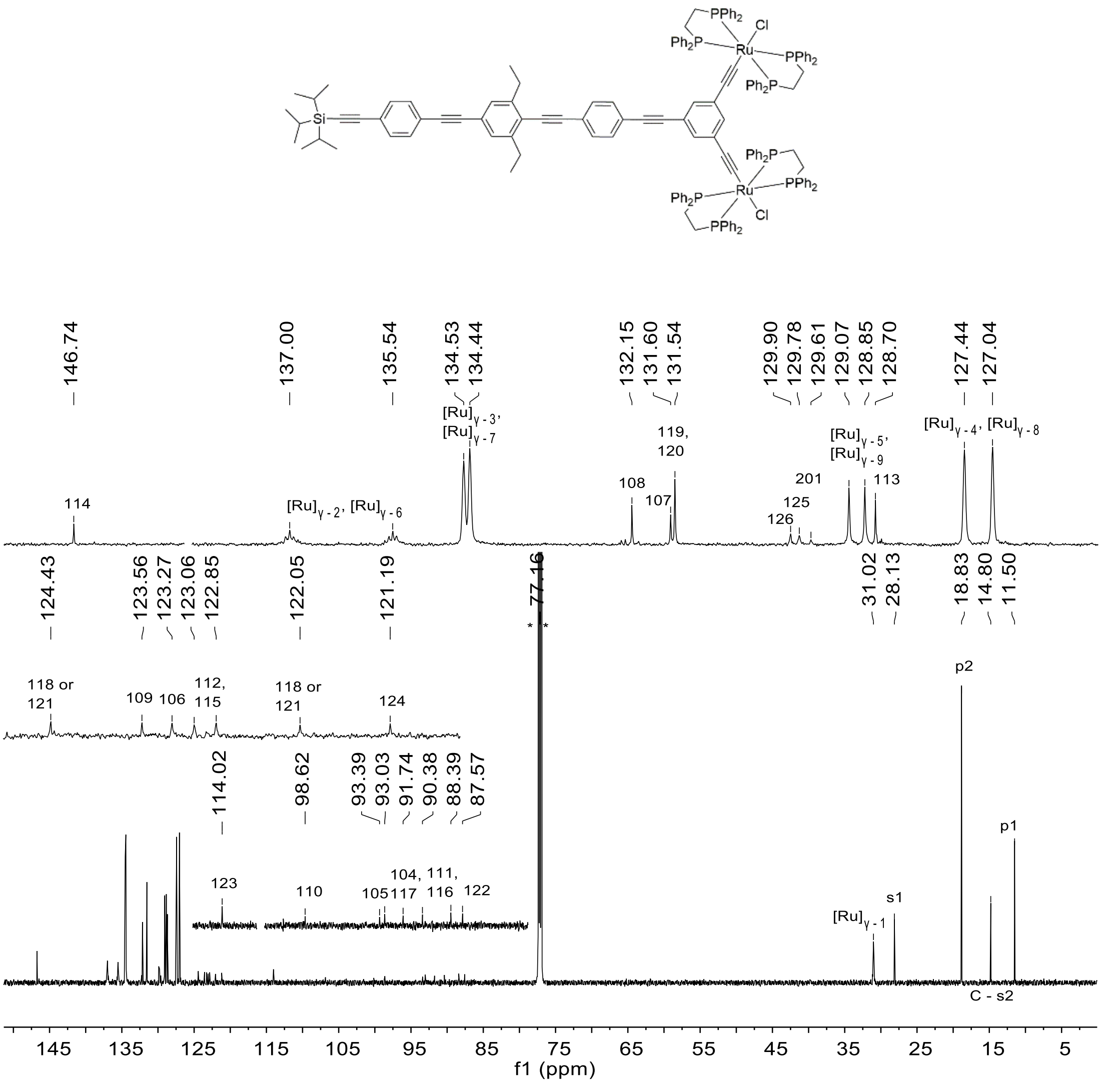

Figure S17. ^{13}C NMR spectrum of **17**. The peak marked as * * corresponds to $CDCl_3$.

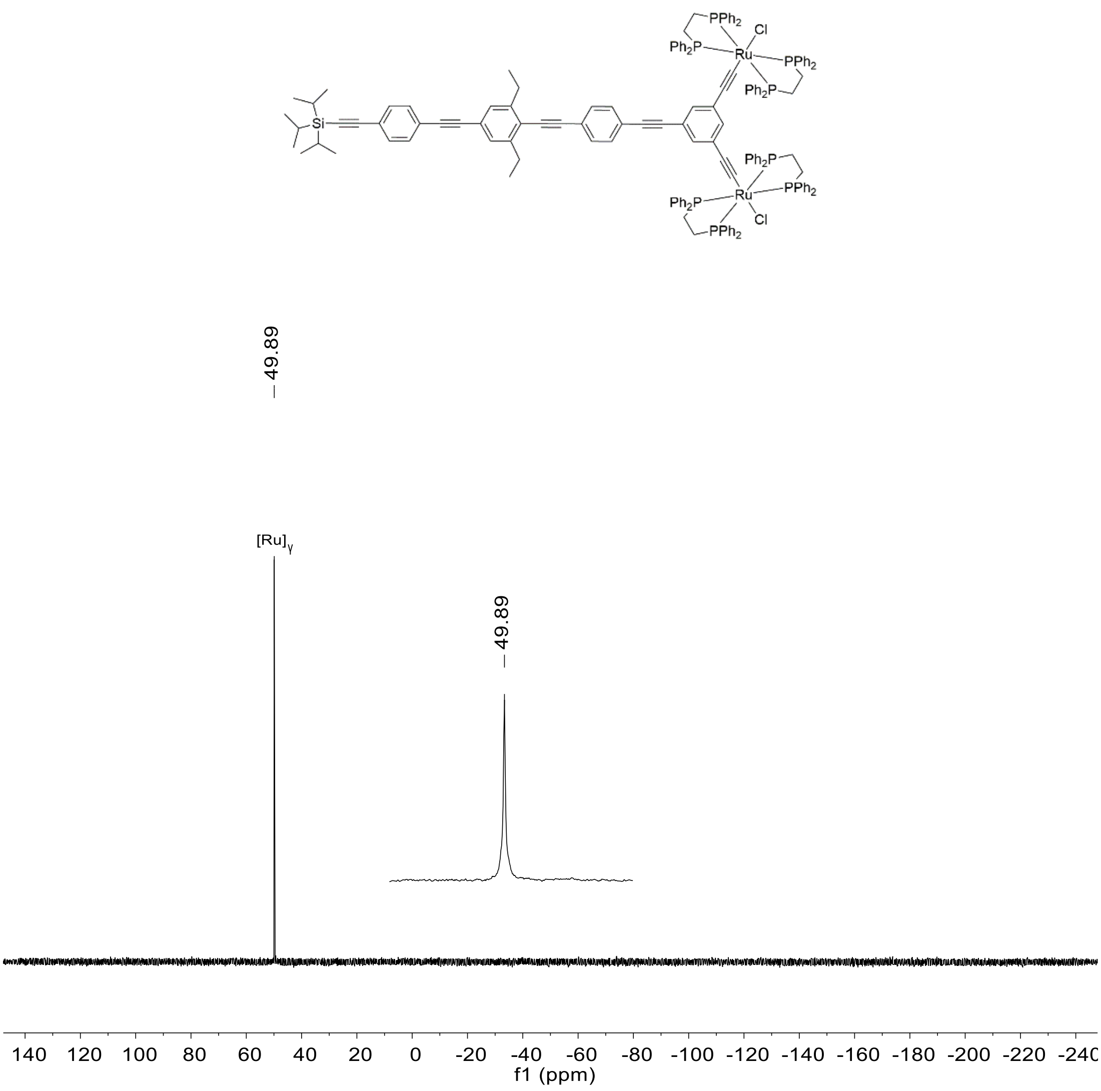

Figure S18. ^{31}P NMR spectrum of **17**.

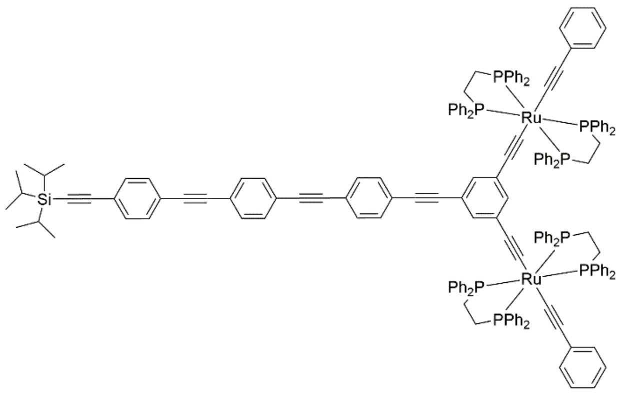

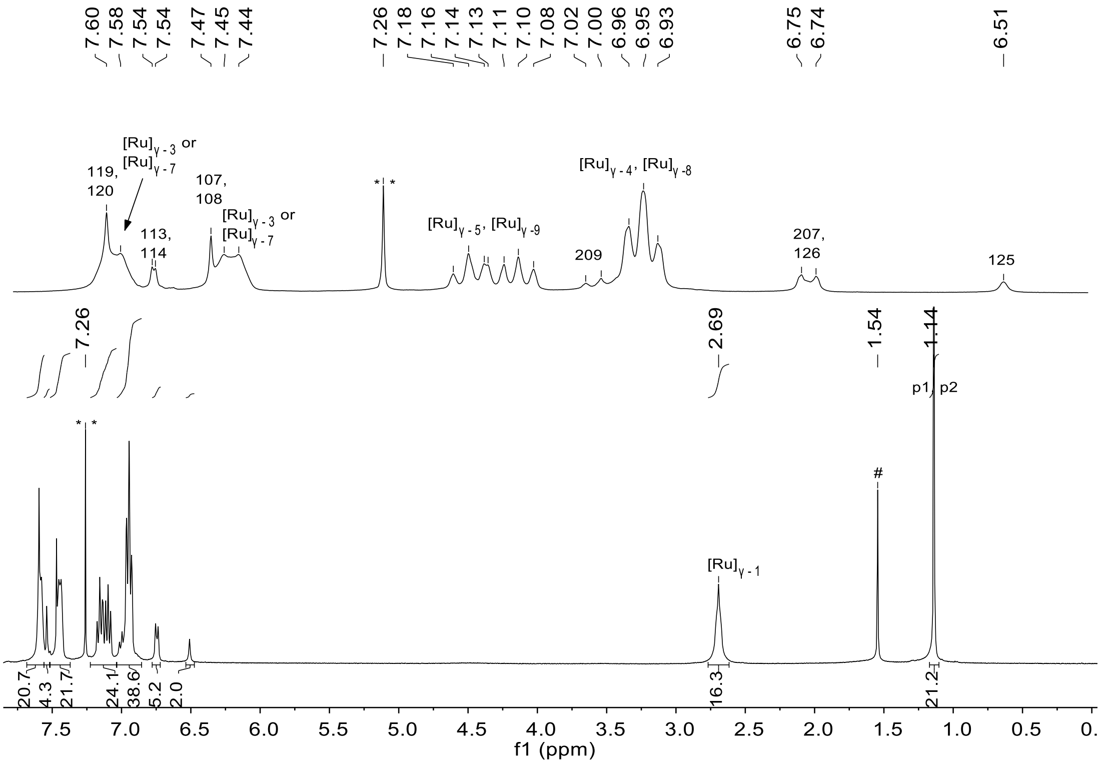

Figure S19. ^{1}H NMR spectrum of **20**. The peak marked as * * corresponds to the residual $CHCl_3$ signal. The peak marked as # corresponds to the residual water signal.

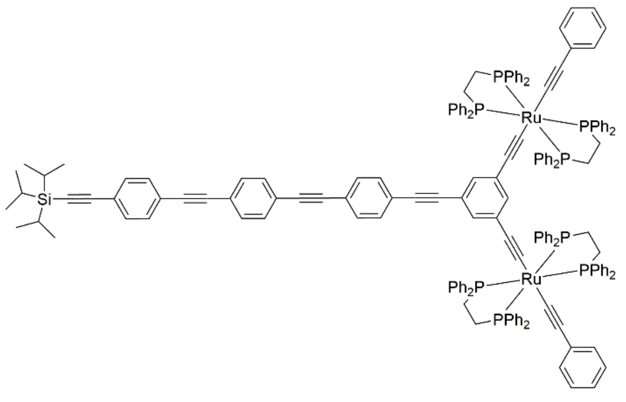

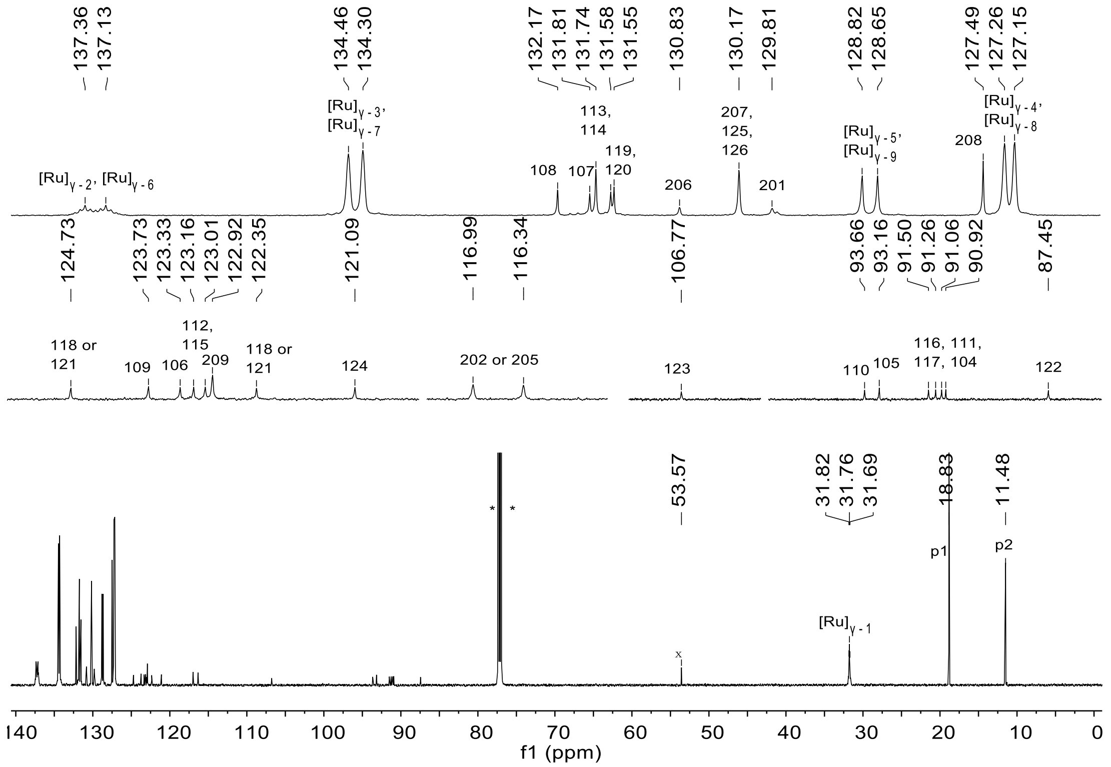

Figure S20. ^{13}C NMR spectrum of **20**. The peak marked as * * corresponds to $CDCl_3$. The peak marked as x corresponds to the residual CH_2Cl_2 signal.

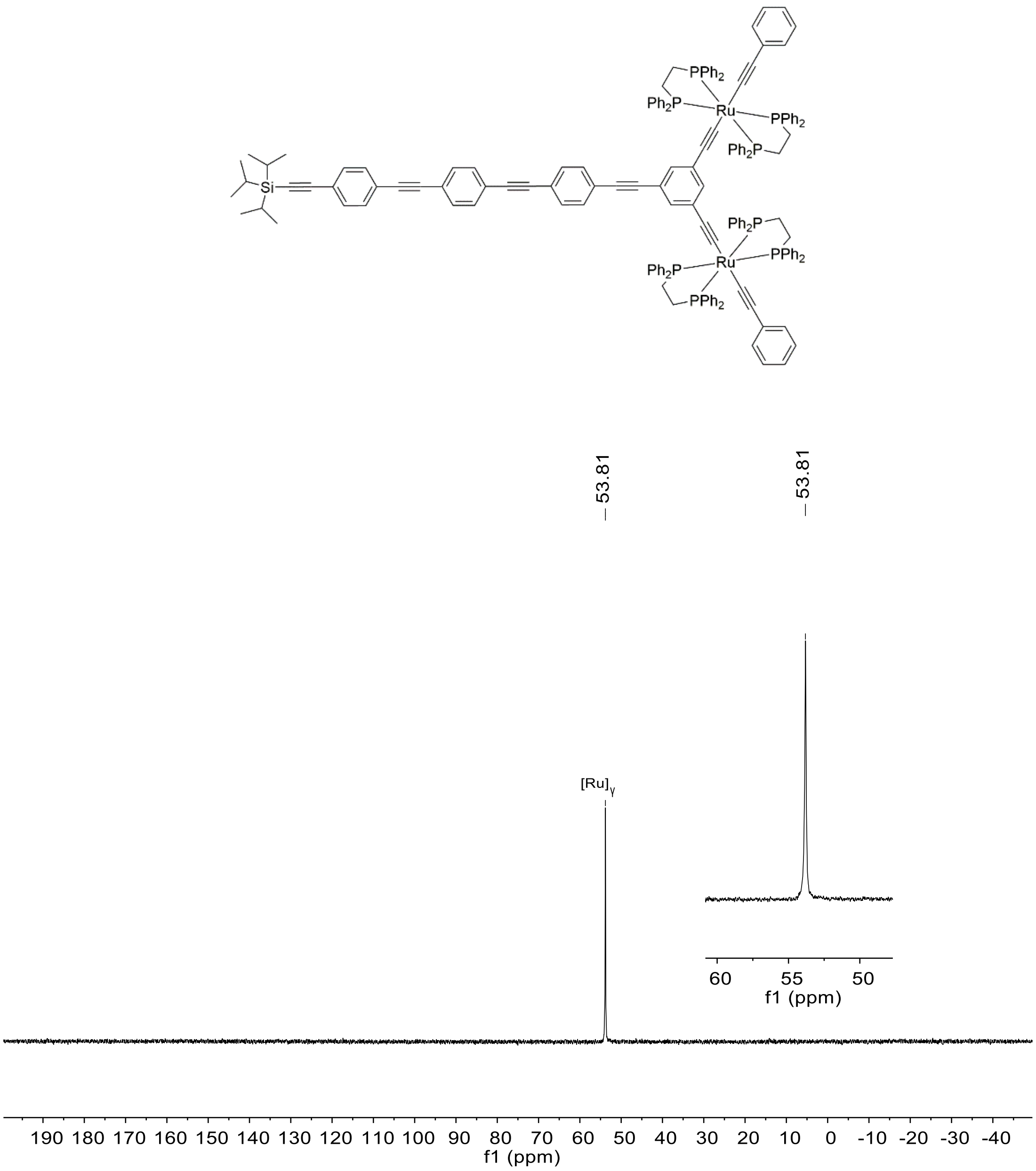

Figure S21. ^{31}P NMR spectrum of **20**.

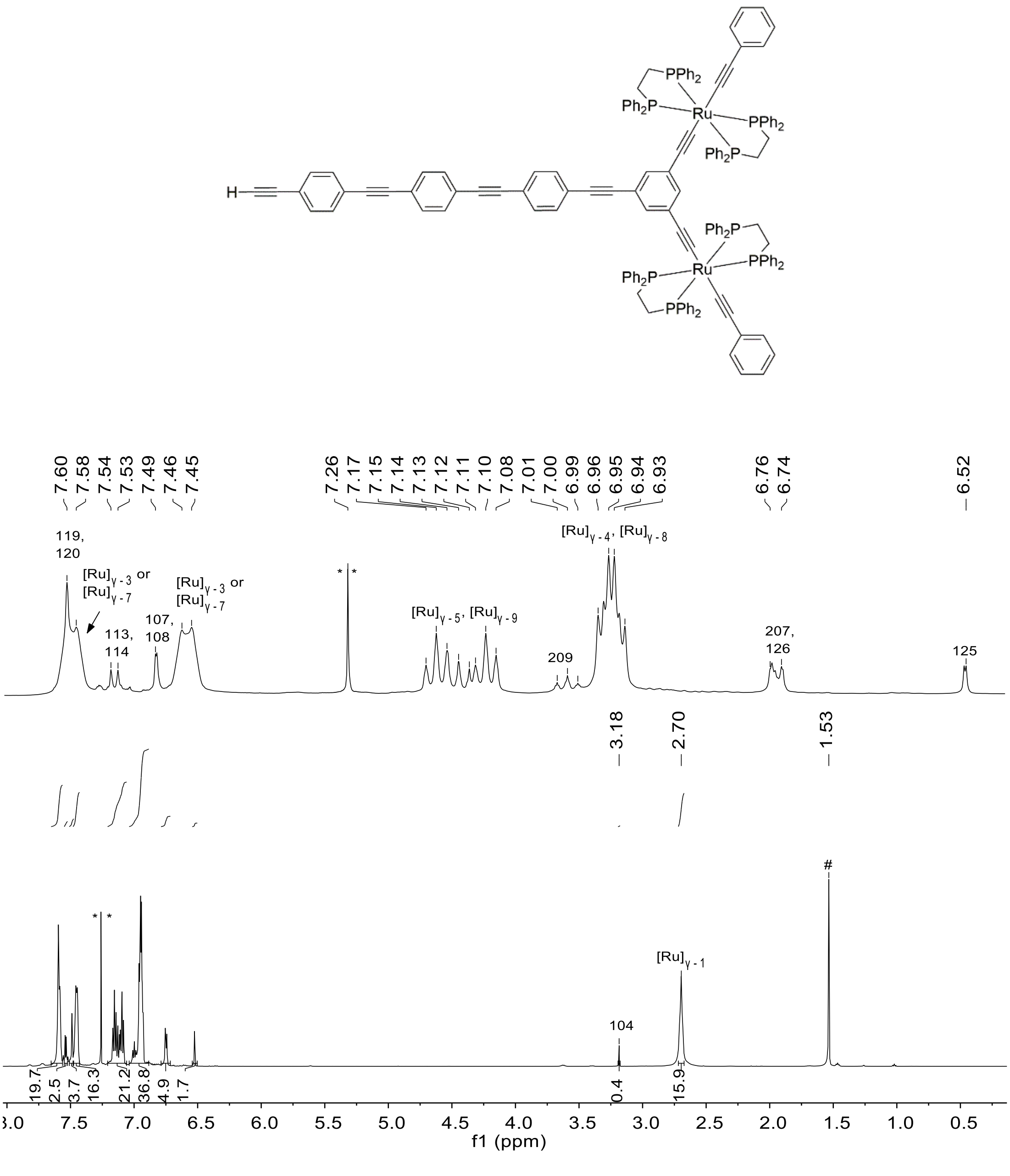

Figure S22. ^{1}H NMR spectrum of **21**. The peak marked as * * corresponds to the residual $CHCl_3$ signal. The peak marked as # corresponds to the residual water signal.

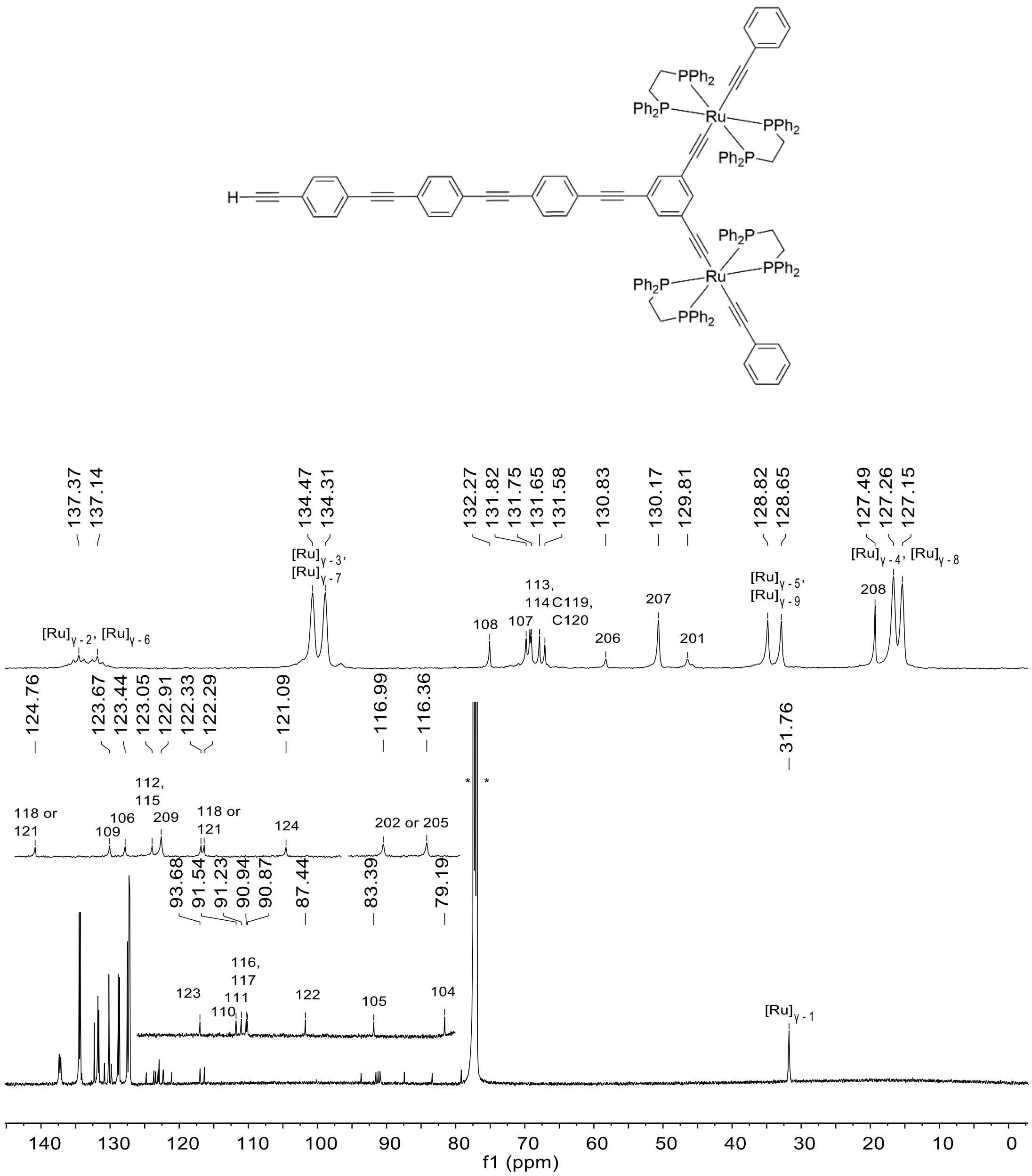

Figure S23. ^{13}C NMR spectrum of **21**. The peak marked as * * corresponds to $CDCl_3$.

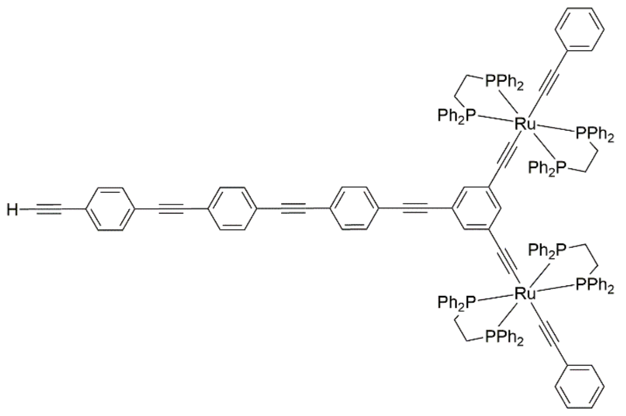

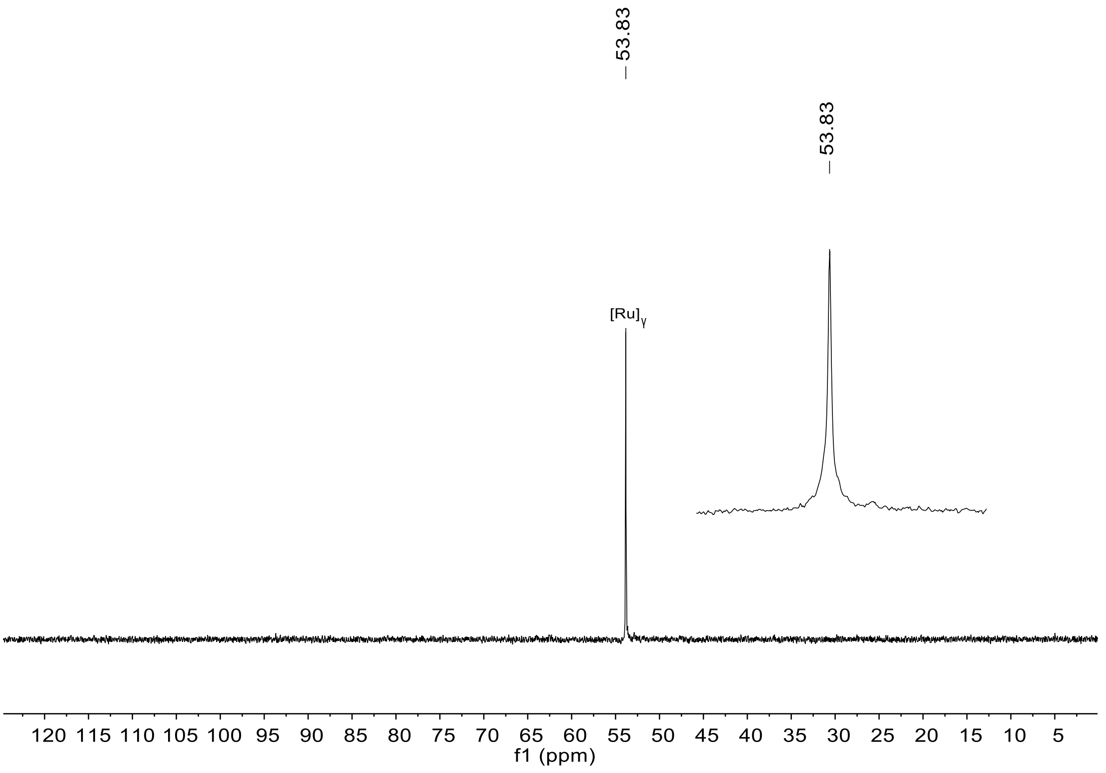

Figure S24. ^{31}P NMR spectrum of **21**.

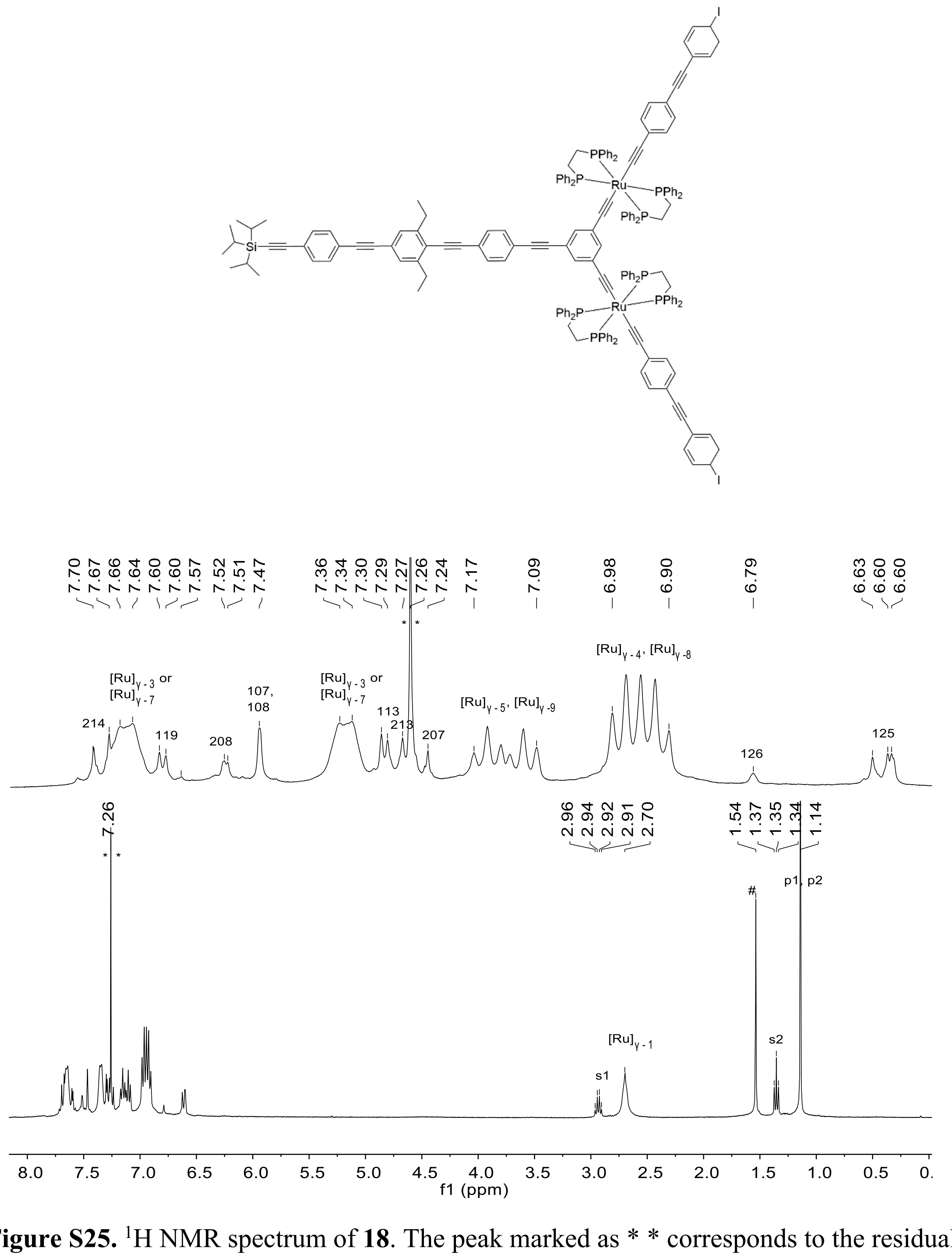

Figure S25. ^{1}H NMR spectrum of **18**. The peak marked as * * corresponds to the residual $CHCl_3$ signal. The peak marked as # corresponds to the residual water signal.

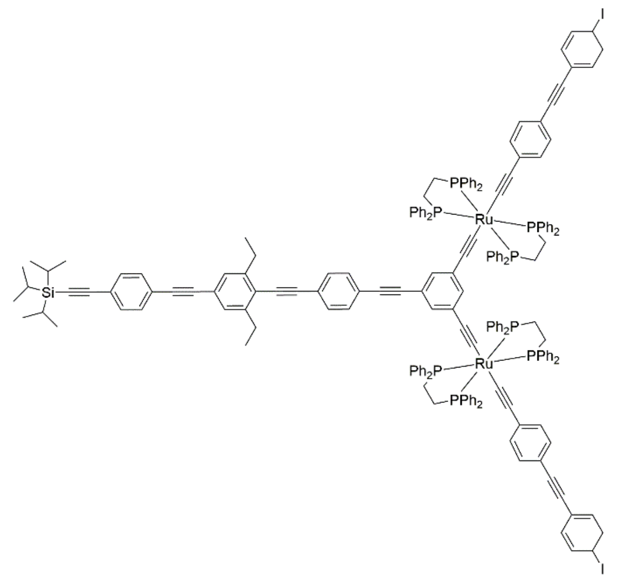

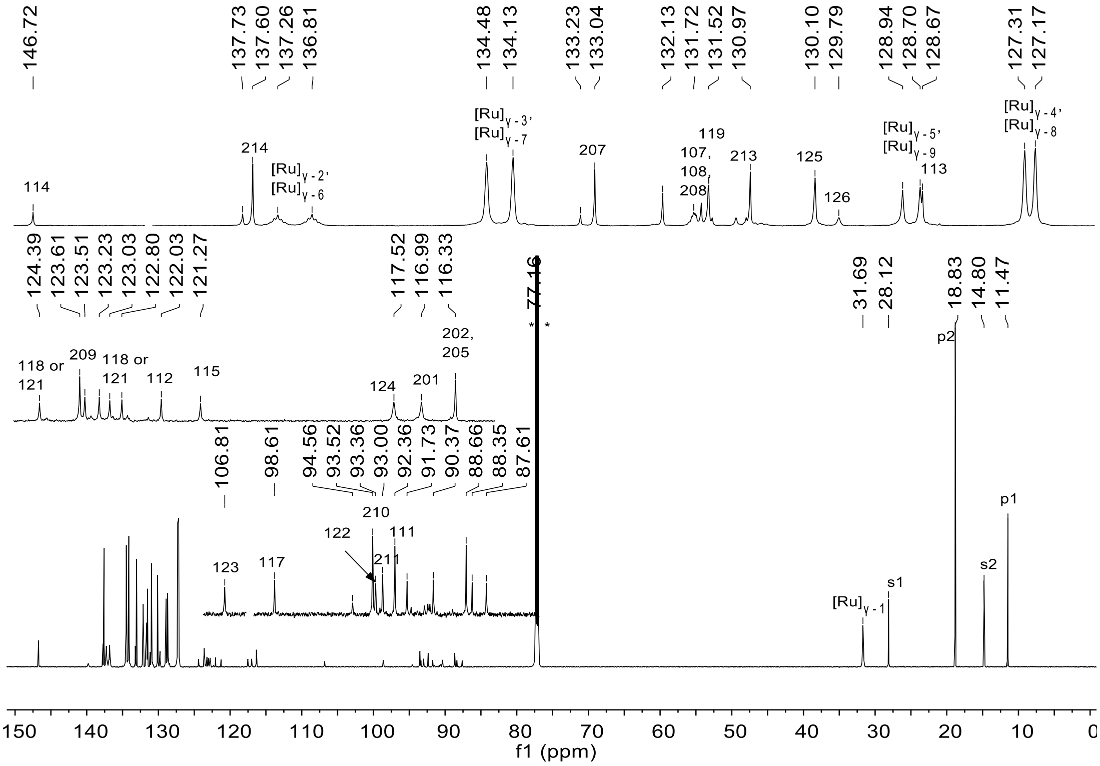

Figure S26. ^{13}C NMR spectrum of **18**. The peak marked as * * corresponds to $CDCl_3$.

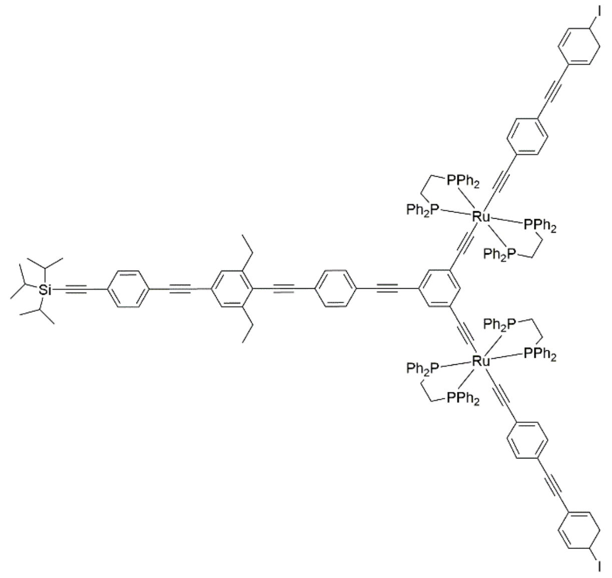

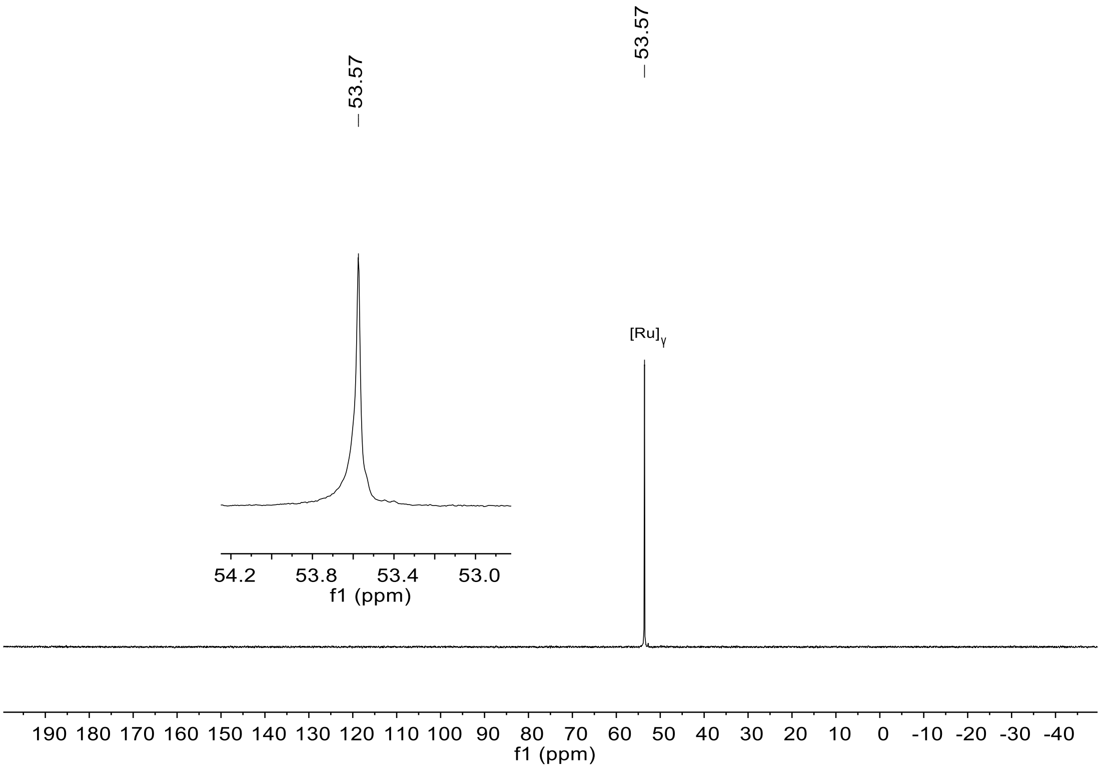

Figure S27. ^{31}P NMR spectrum of **18**.

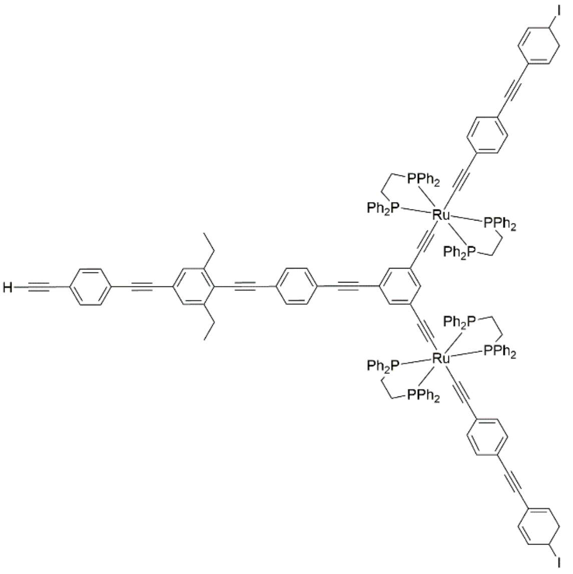

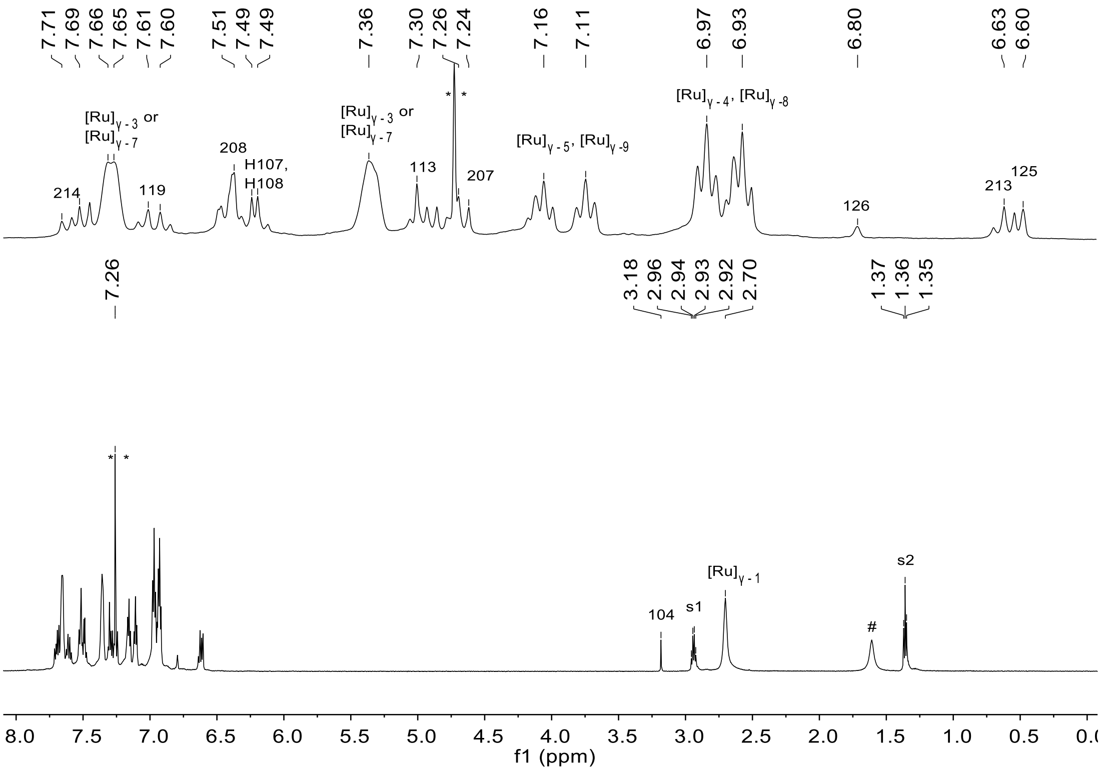

Figure S28. ^{1}H NMR spectrum of **19**. The peak marked as * * corresponds to the residual $CHCl_3$ signal. The peak marked as # corresponds to the residual water signal.

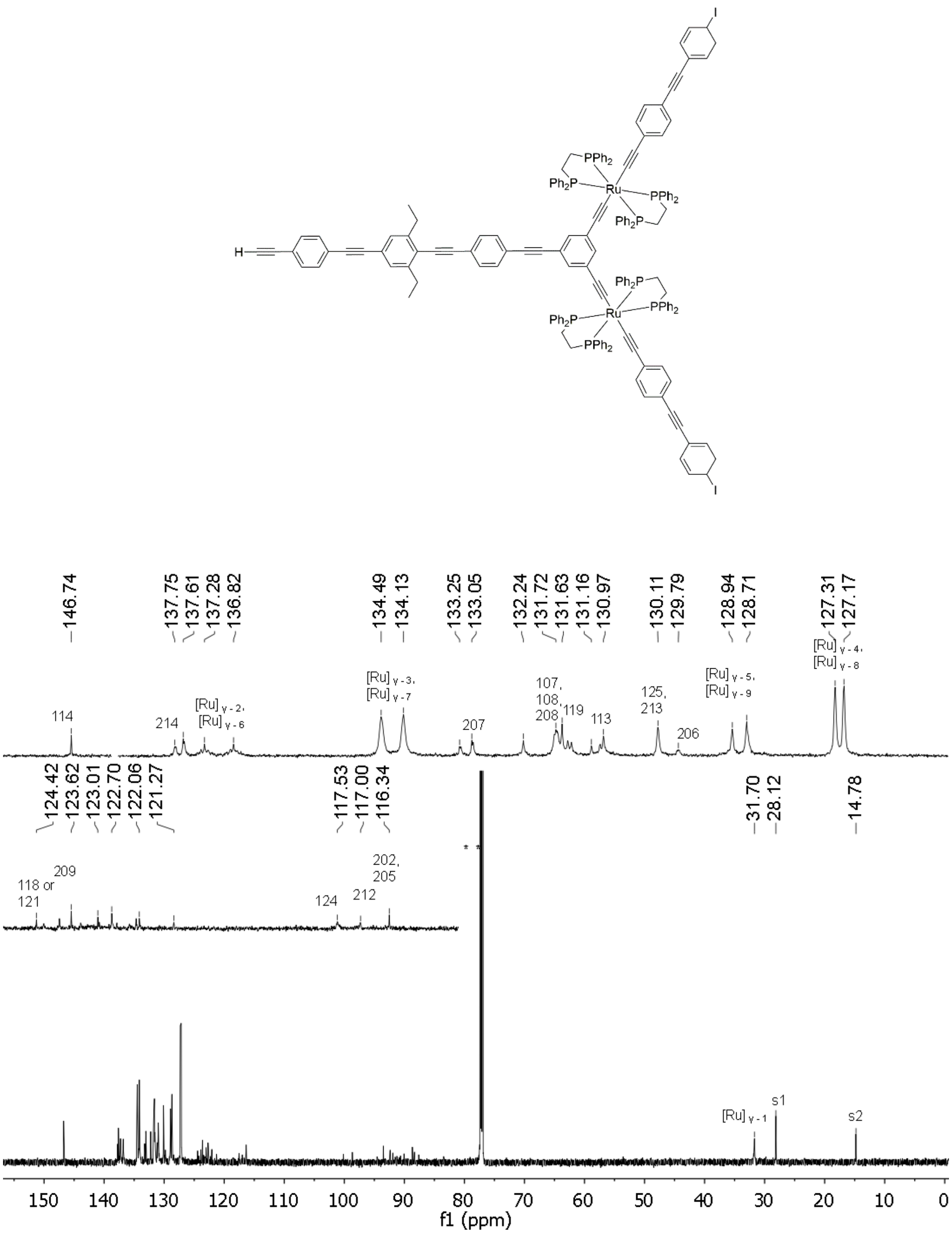

Figure S29. ^{13}C NMR spectrum of **19**. The peak marked as * * corresponds to $CDCl_3$.

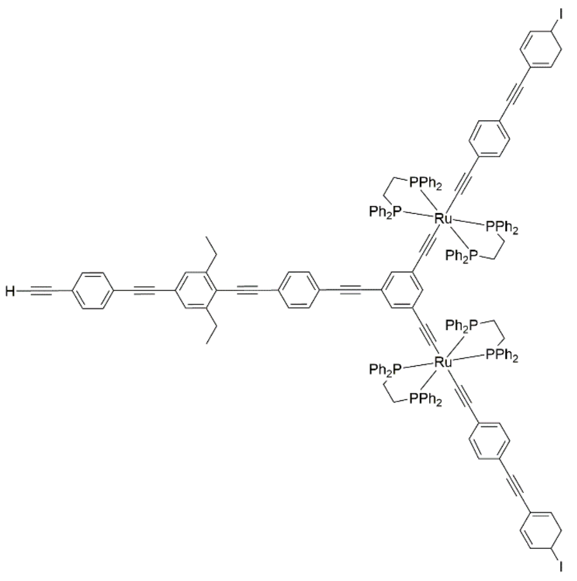

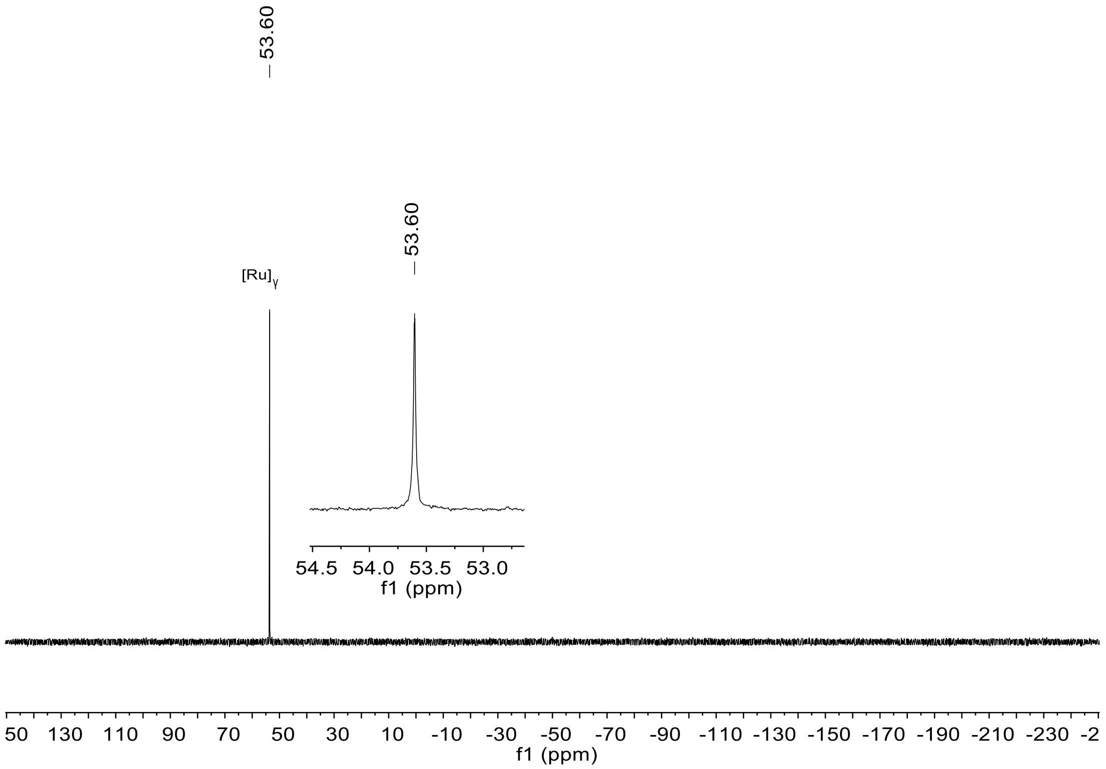

Figure S30. ^{31}P NMR spectrum of **19**.

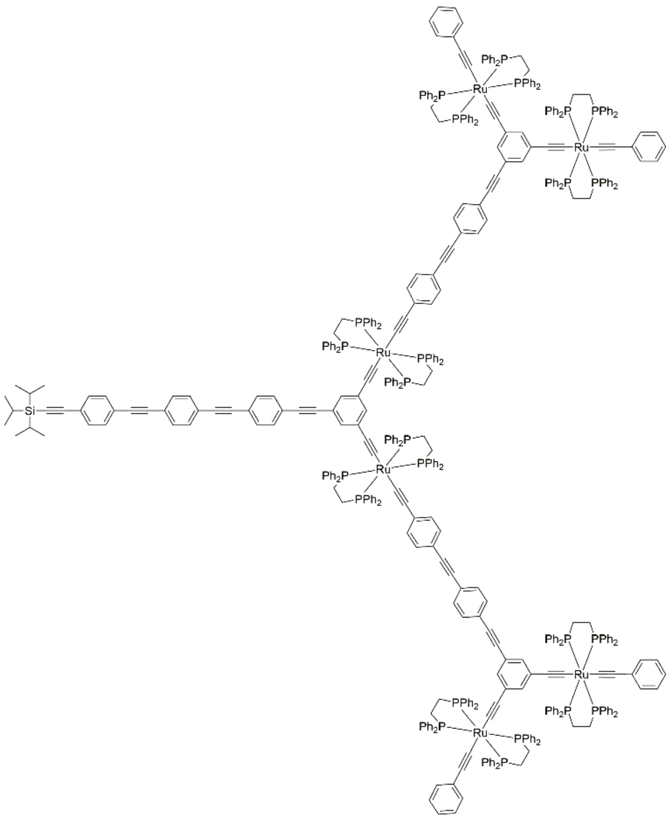

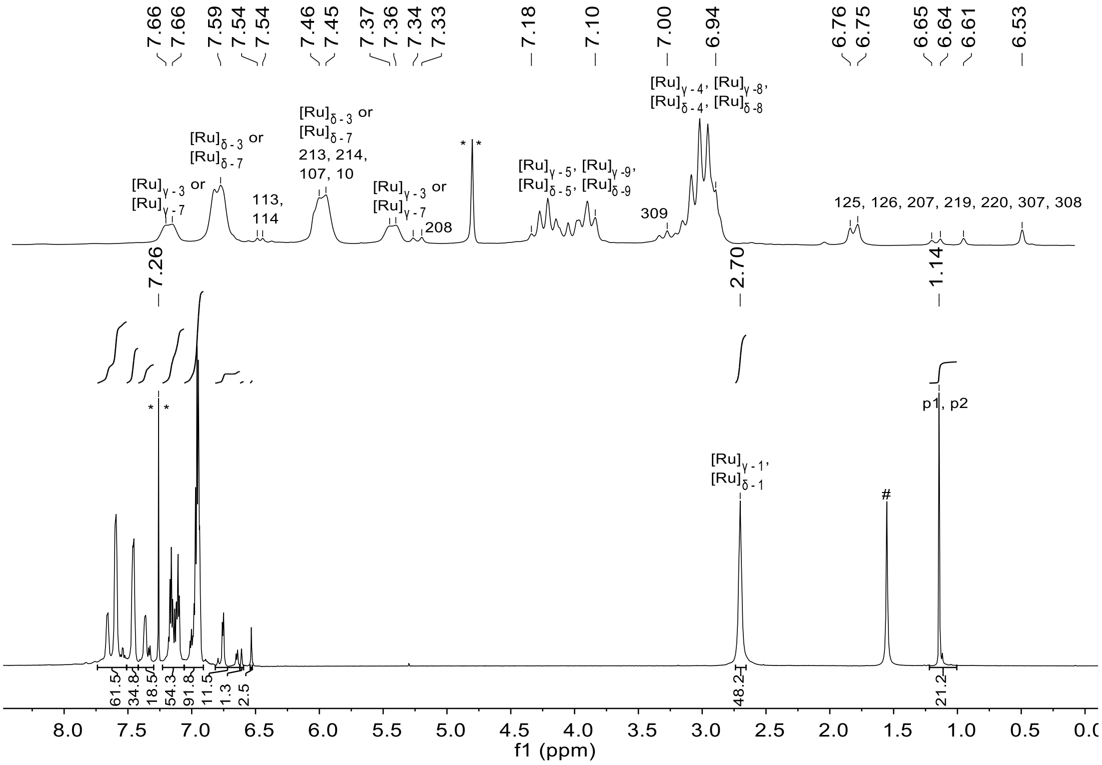

Figure S31. ^{1}H NMR spectrum of **24**. The peak marked as * * corresponds to the residual $CHCl_3$ signal. The peak marked as # corresponds to the residual water signal.

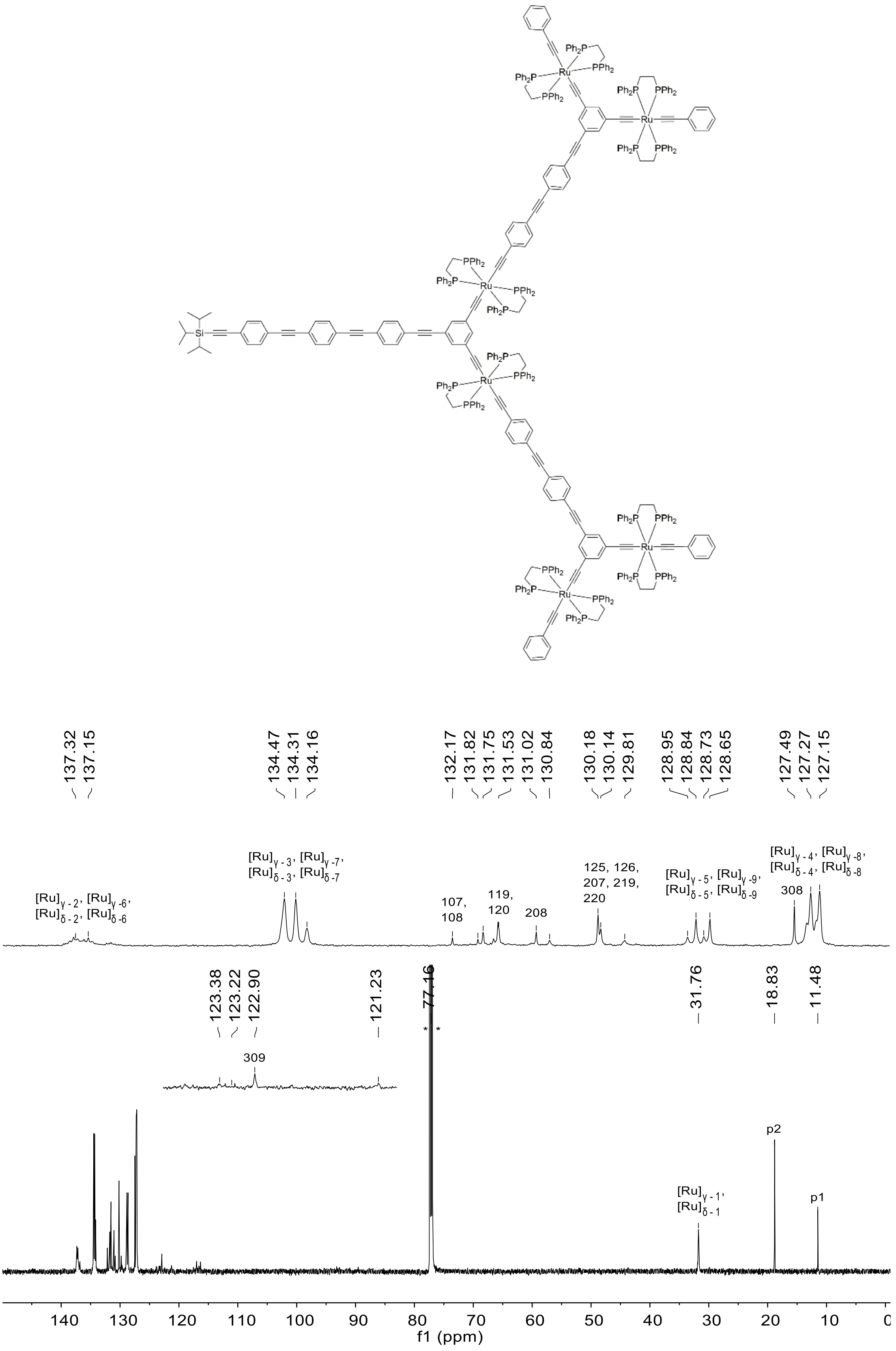

Figure S32. ^{13}C NMR spectrum of **24**. The peak marked as * * corresponds to $CDCl_3$.

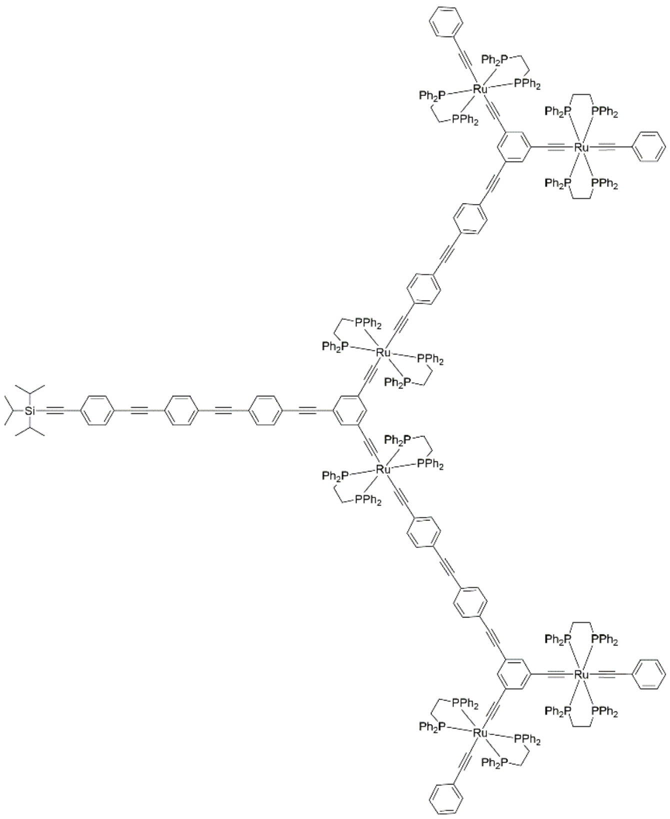

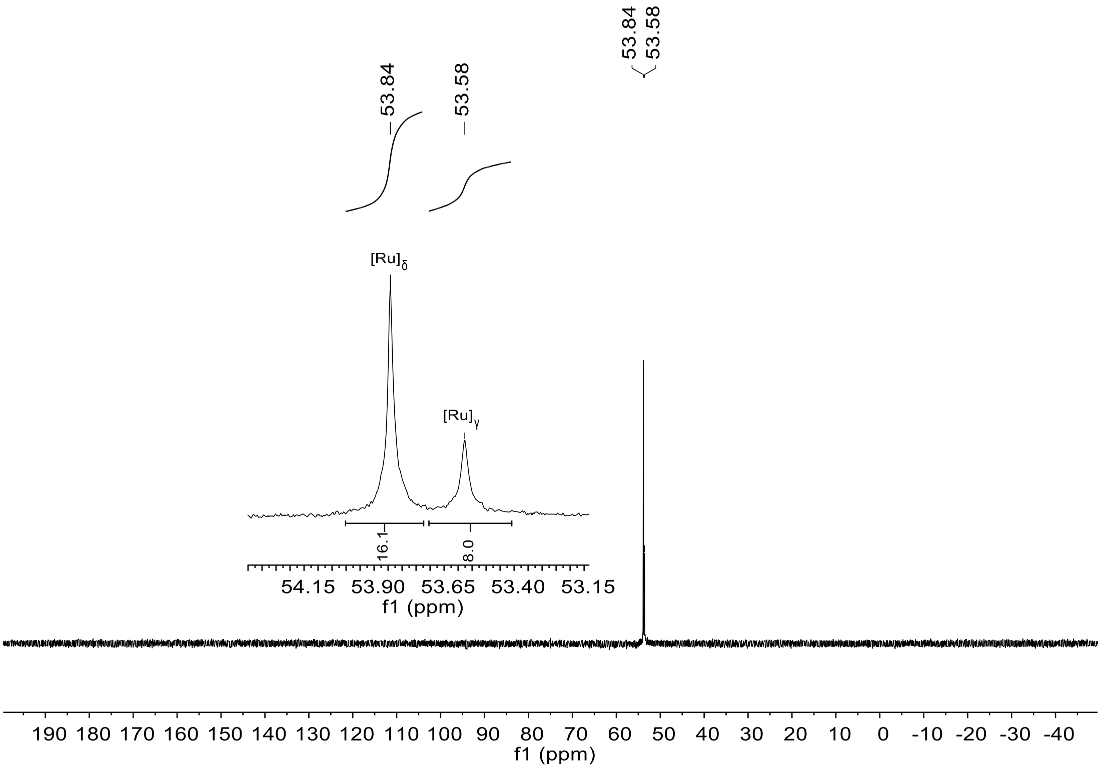

Figure S33. ^{31}P NMR spectrum of **24**.

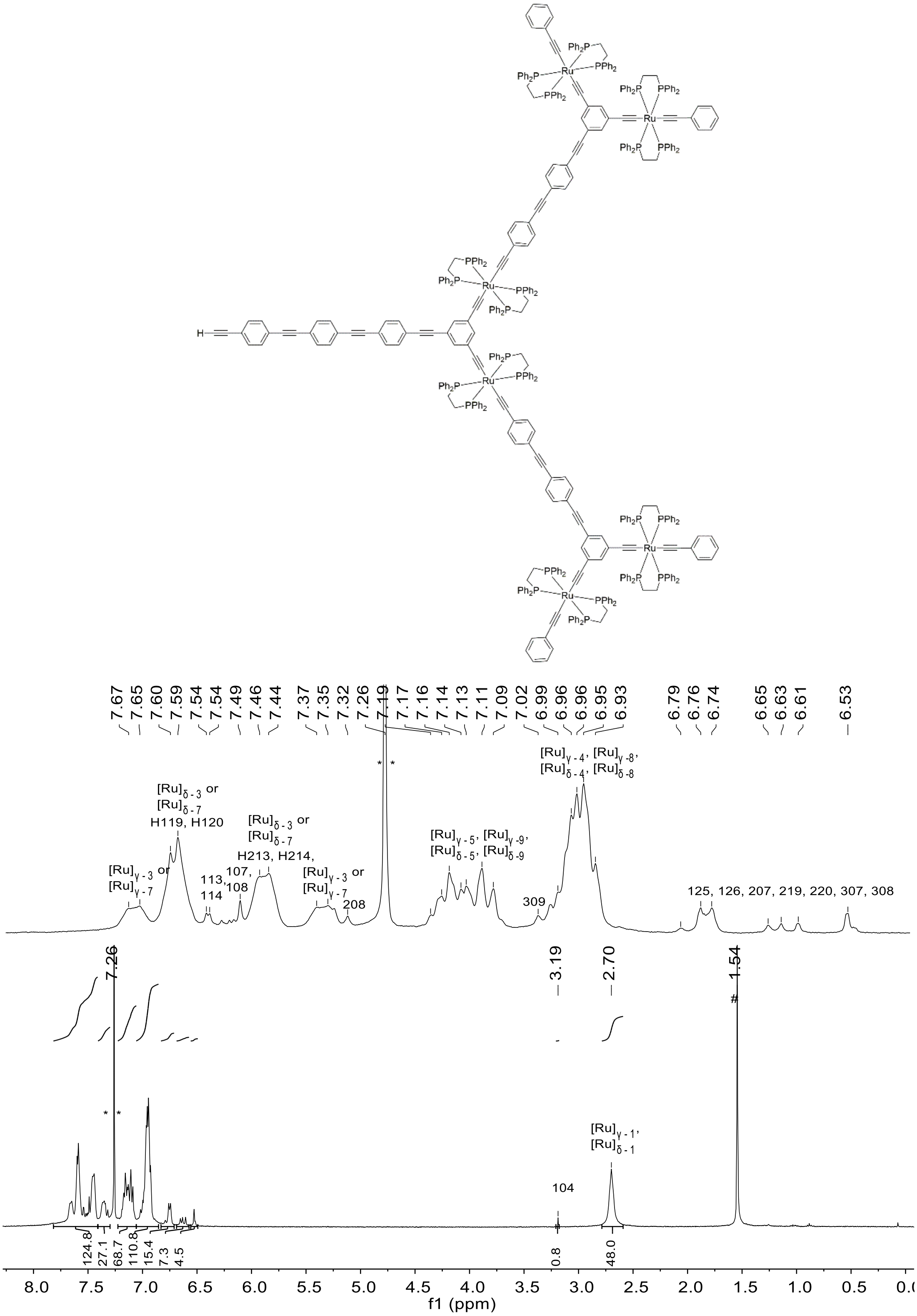

Figure S34. ^{1}H NMR spectrum of **25**. The peak marked as * * corresponds to the residual $CHCl_3$ signal. The peak marked as # corresponds to the residual water signal.

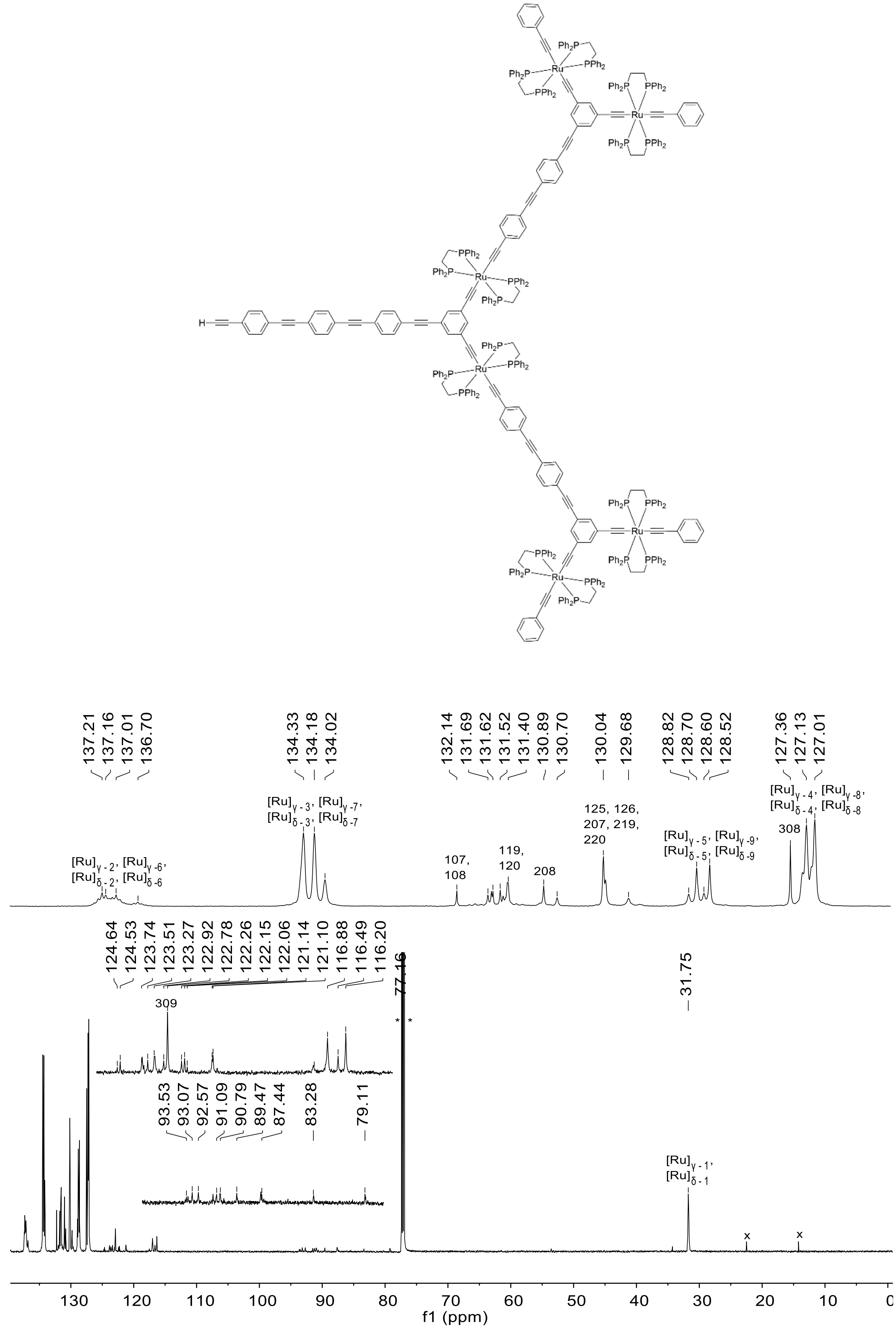

Figure S35. ^{13}C NMR spectrum of **25**. The peak marked as * * corresponds to $CDCl_3$. The peak marked as x corresponds to *n*-pentane.

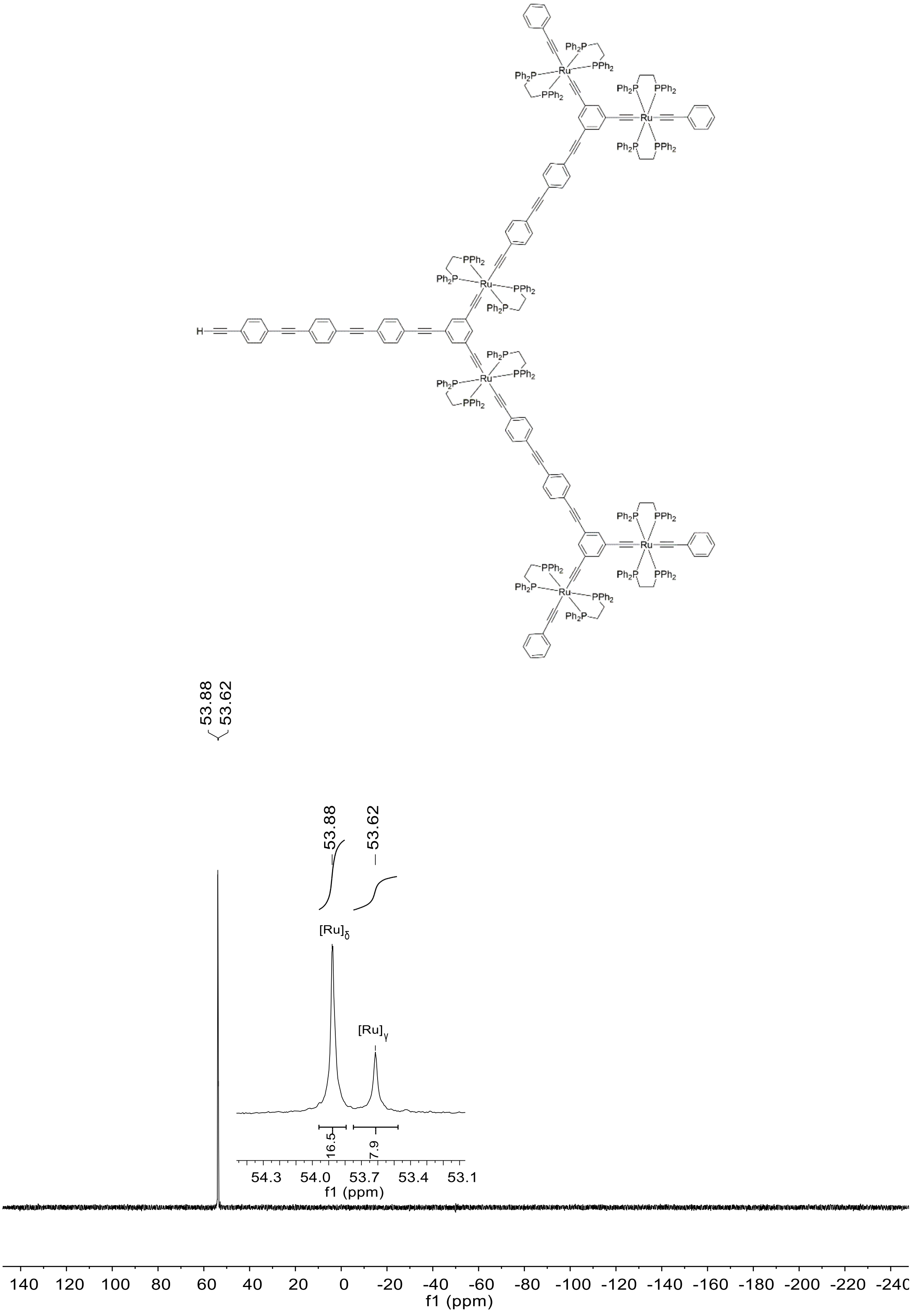

Figure S36. ^{31}P NMR spectrum of **25**.

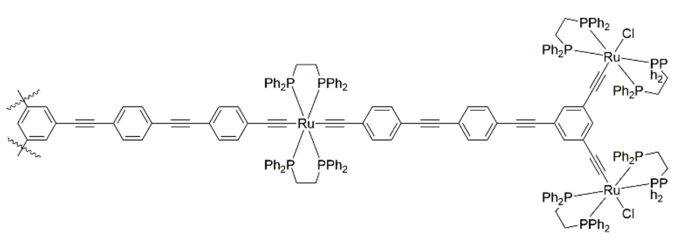

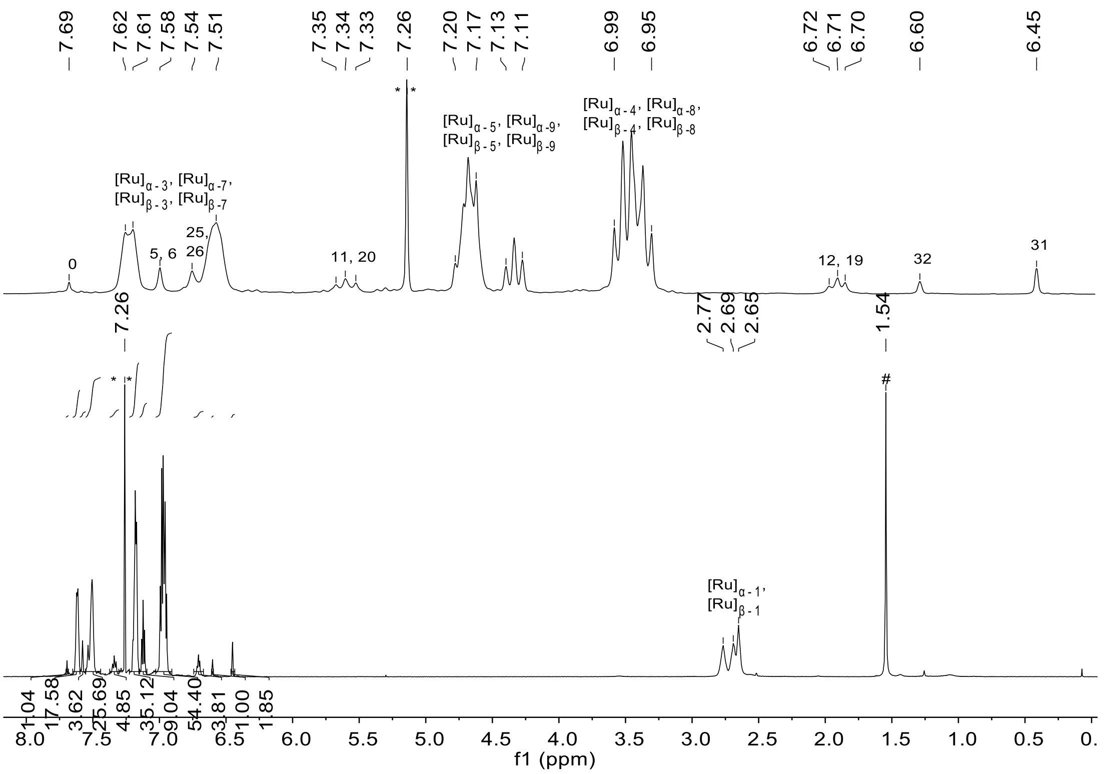

Figure S37. ^{1}H NMR spectrum of **32**. The peak marked as * * corresponds to the residual $CHCl_3$ signal. The peak marked as # corresponds to the residual water signal.

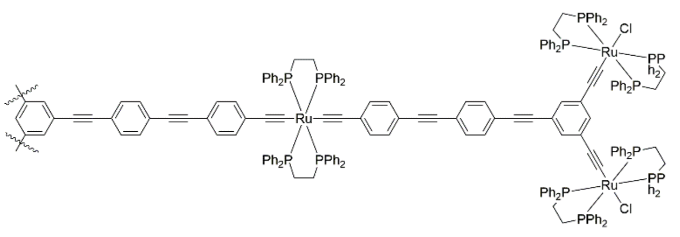

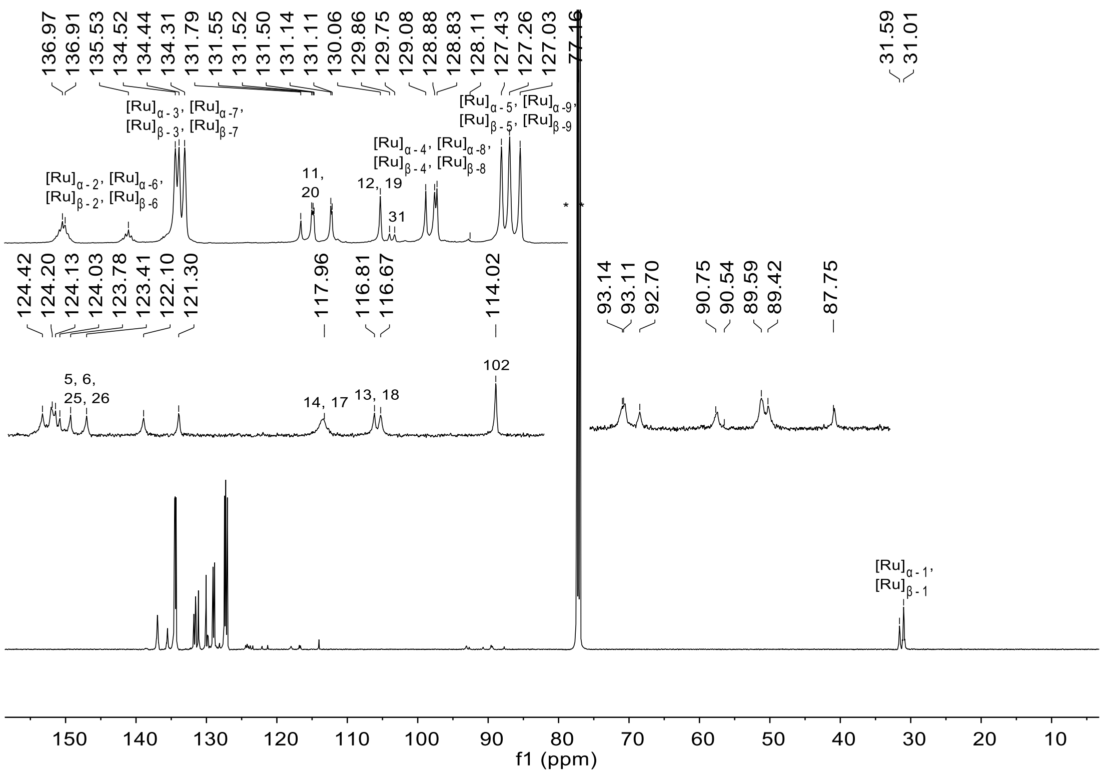

Figure S38. ^{13}C NMR spectrum of **32**. The peak marked as * * corresponds to $CDCl_3$.

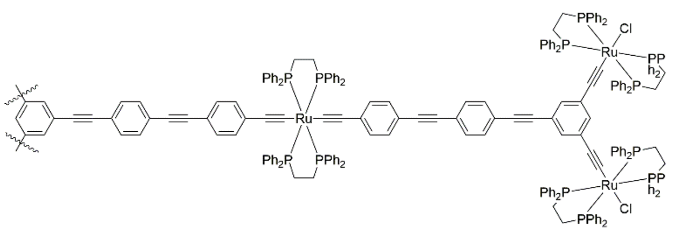

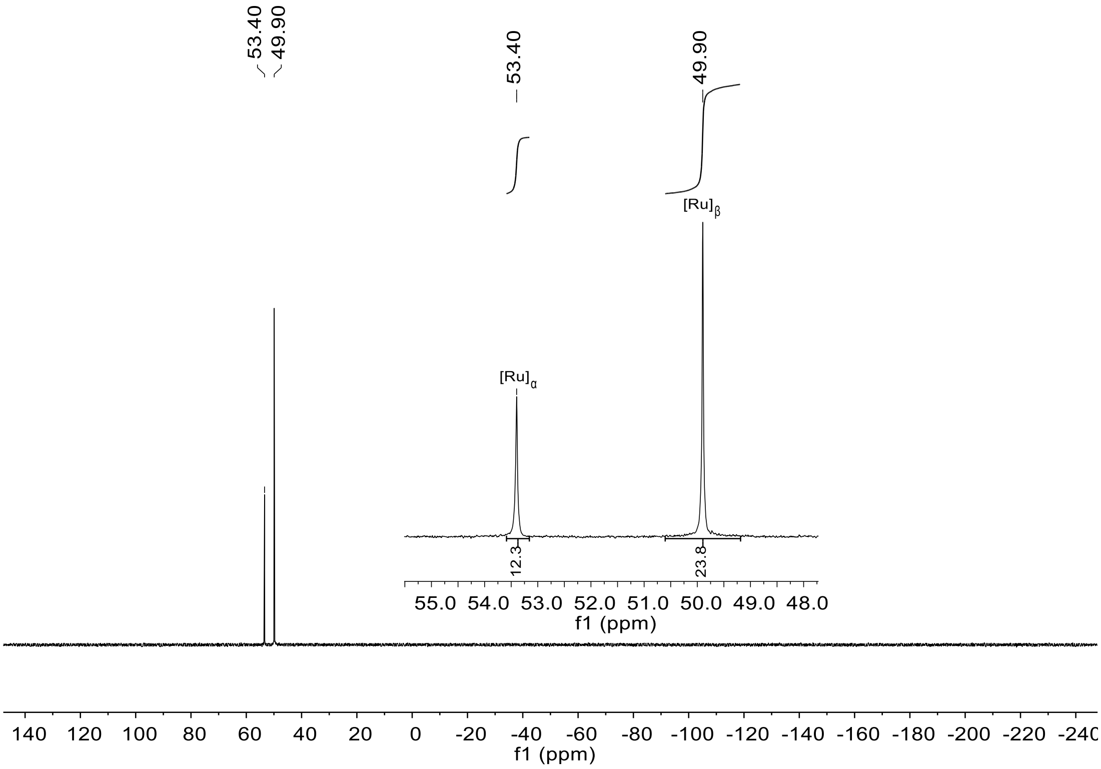

Figure S39. ^{31}P NMR spectrum of **32**.

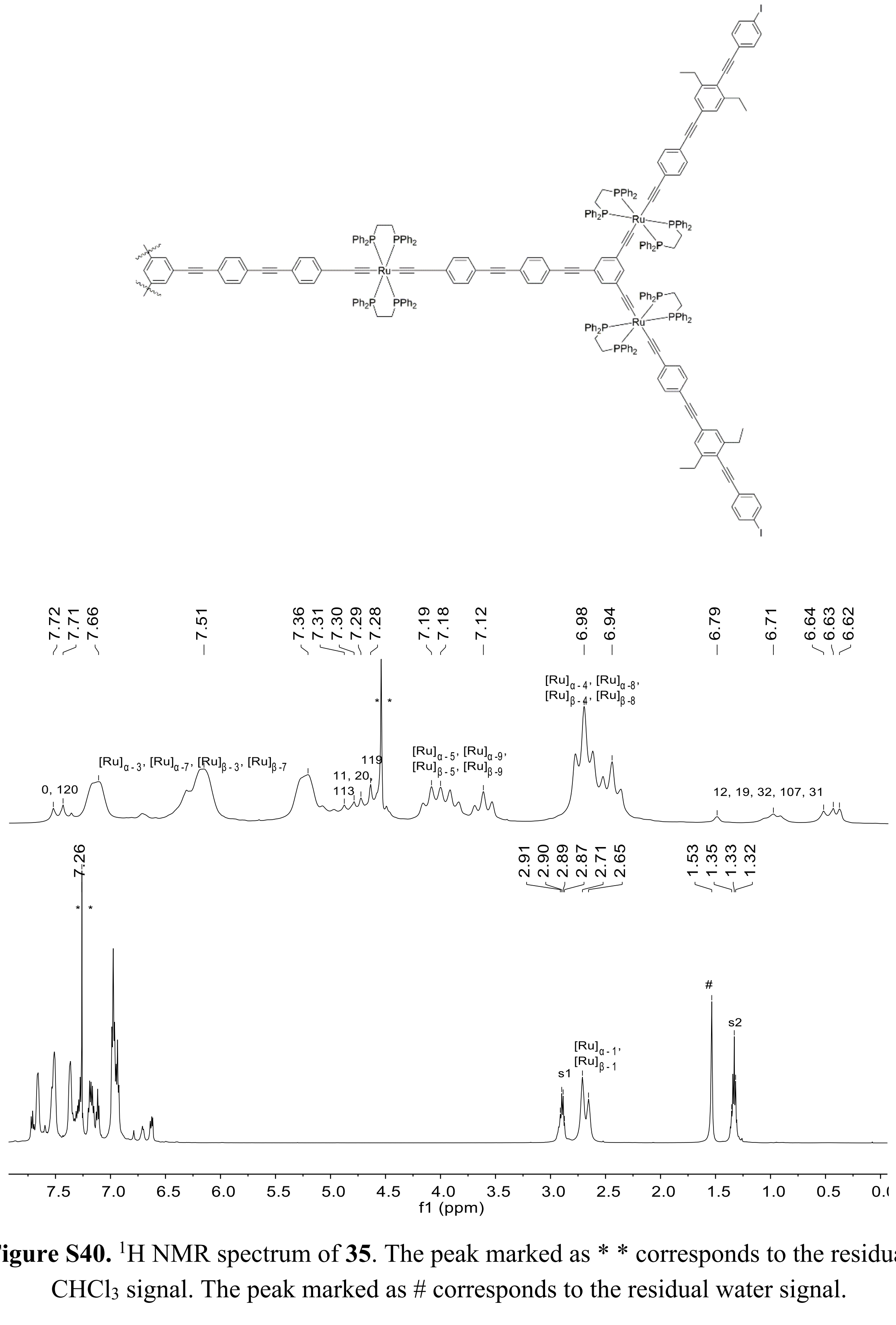

Figure S40. ^{1}H NMR spectrum of **35**. The peak marked as * * corresponds to the residual $CHCl_3$ signal. The peak marked as # corresponds to the residual water signal.

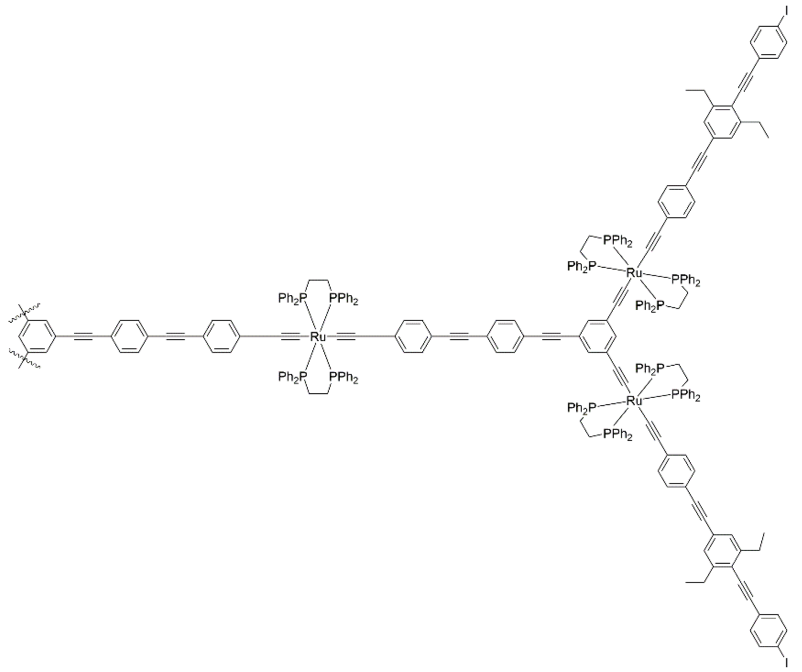

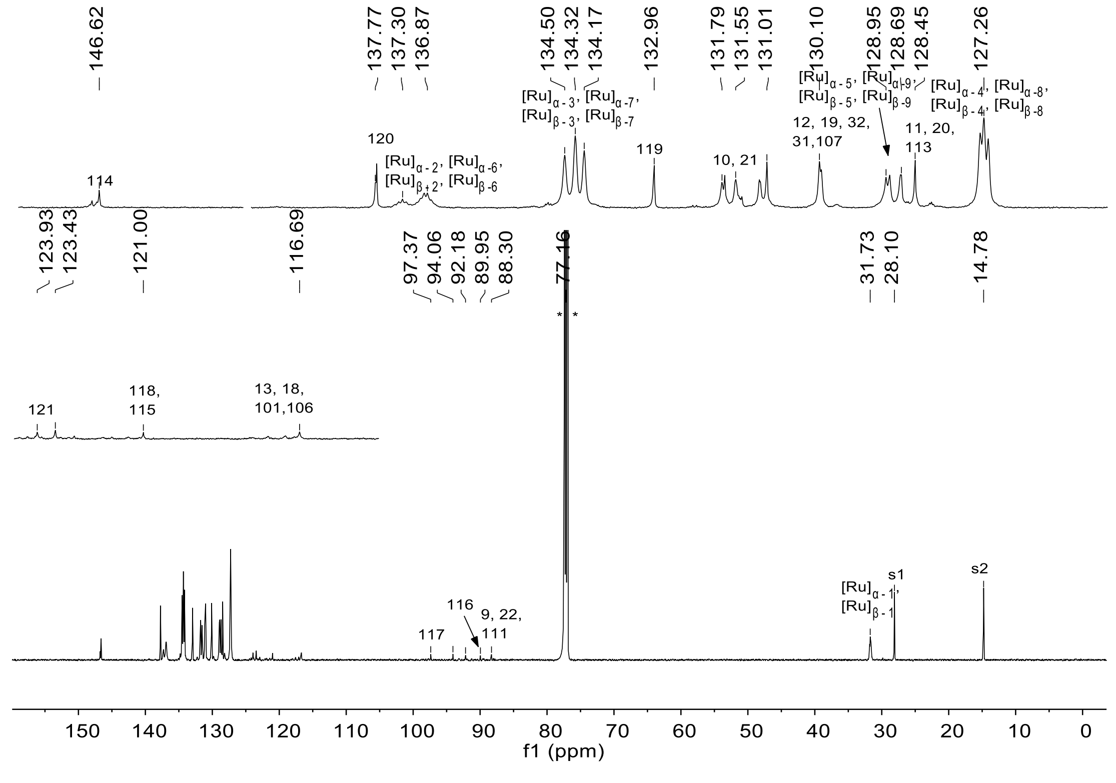

Figure S41. ^{13}C NMR spectrum of **35**. The peak marked as * * corresponds to $CDCl_3$.

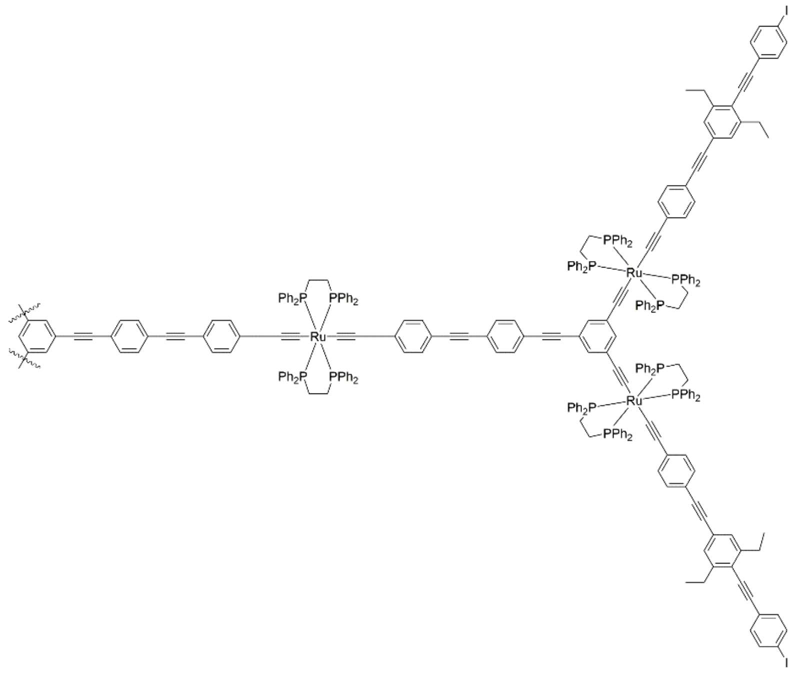

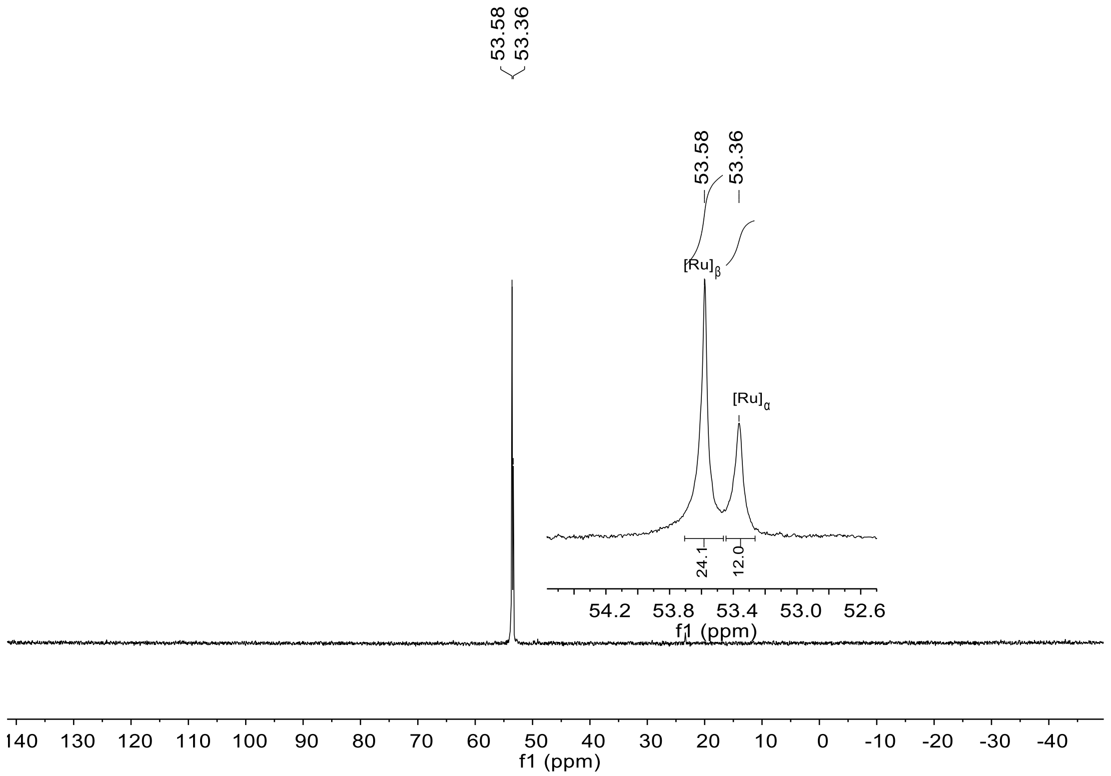

Figure S42. ^{31}P NMR spectrum of **35**.

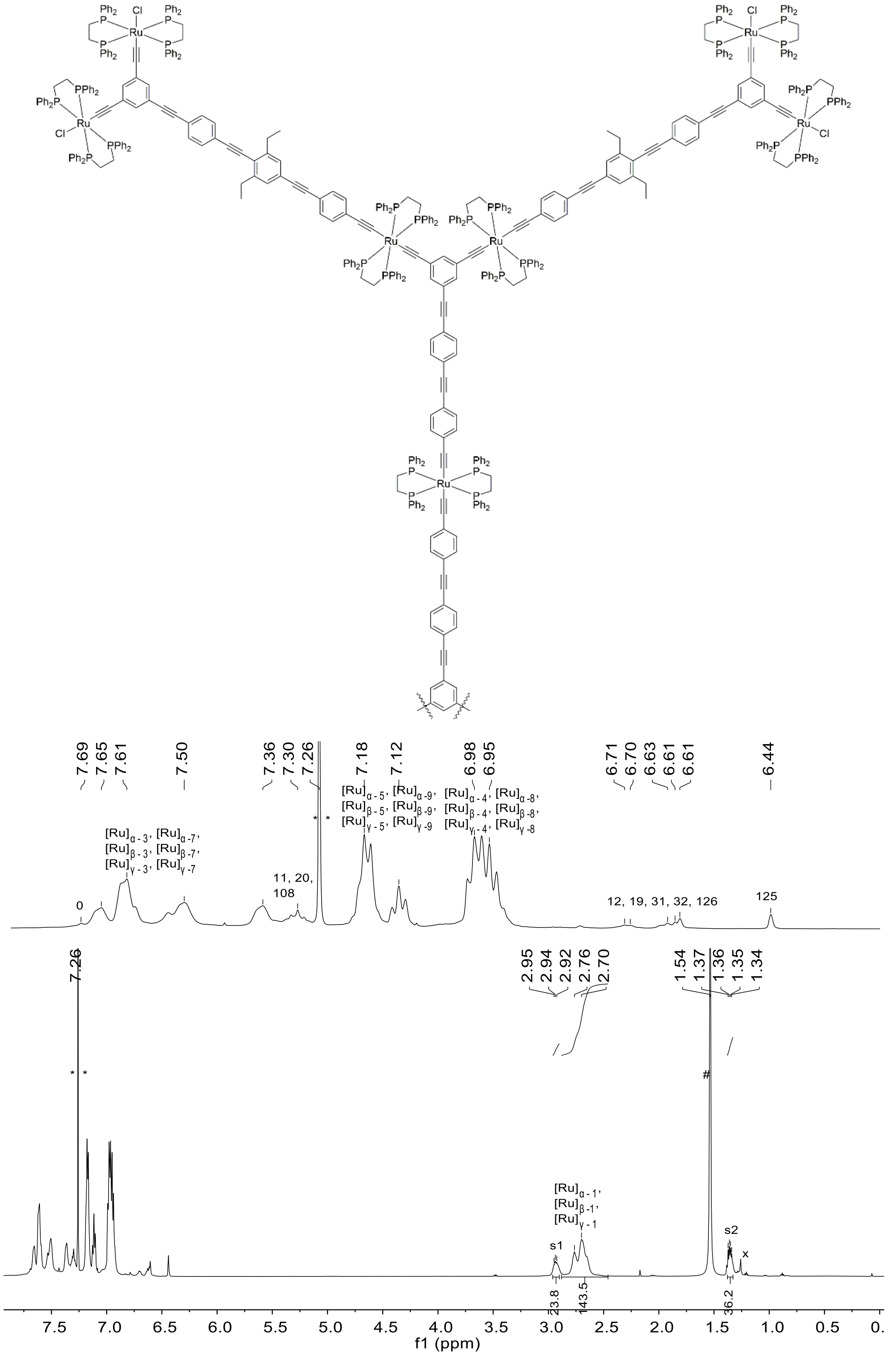

Figure S43. ^{1}H NMR spectrum of **37**. The peak marked as * * corresponds to the residual $CHCl_3$ signal. The peak marked as # corresponds to the residual water signal.

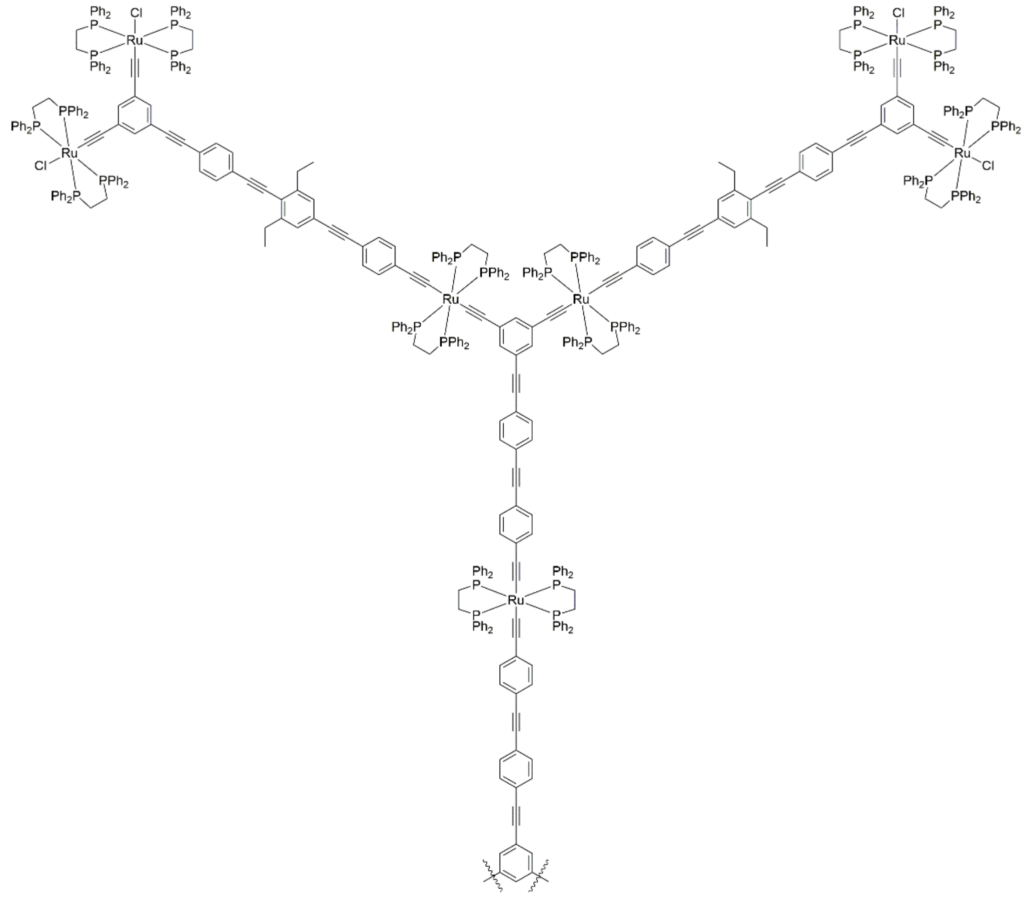

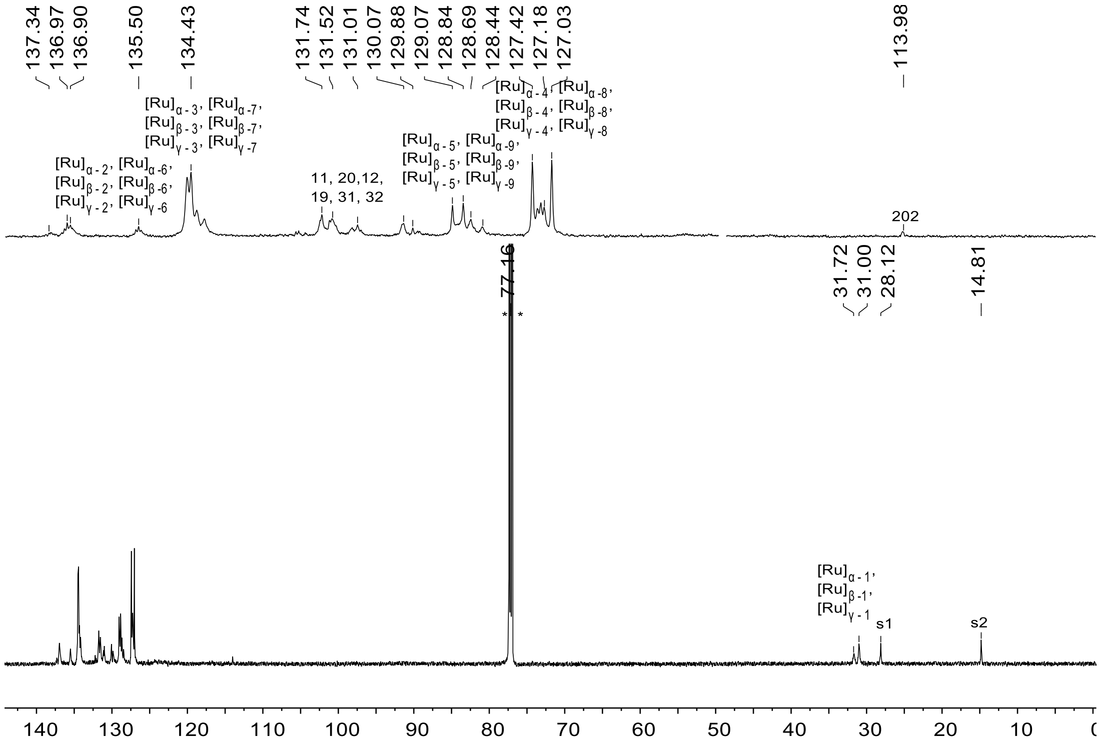

Figure S44. ^{13}C NMR spectrum of **37**. The peak marked as * * corresponds to $CDCl_3$.

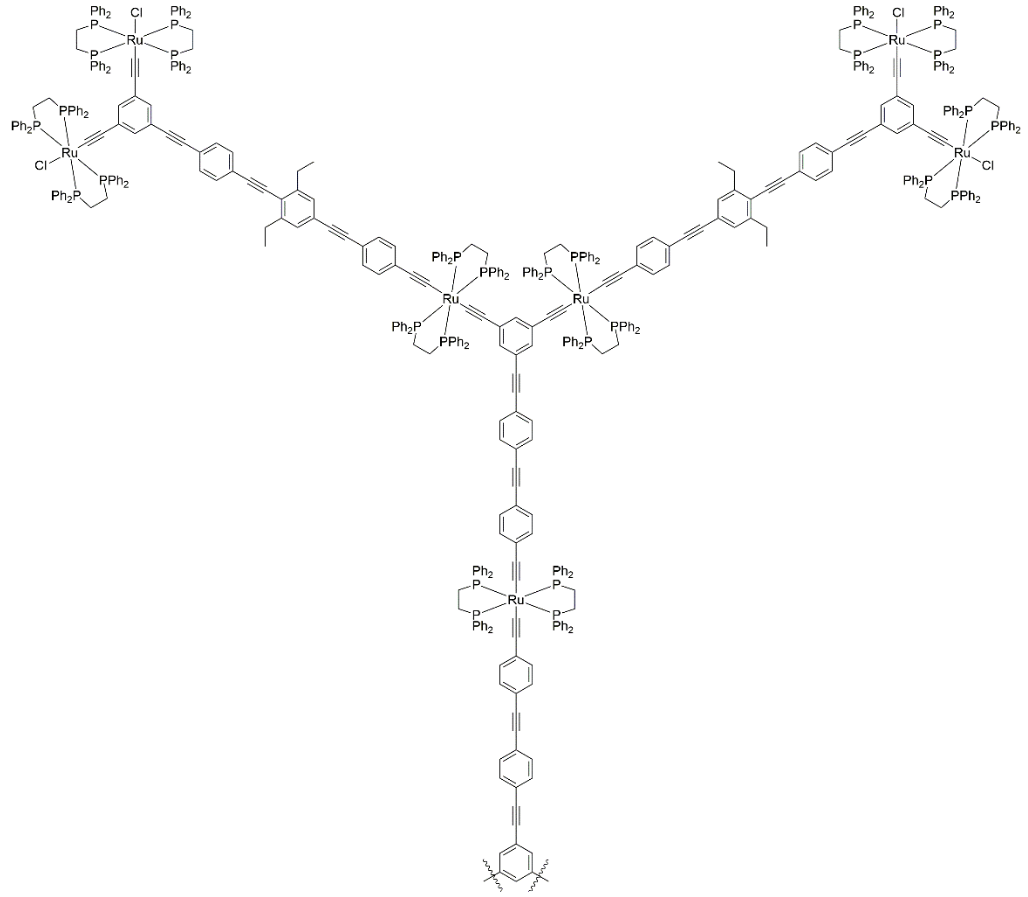

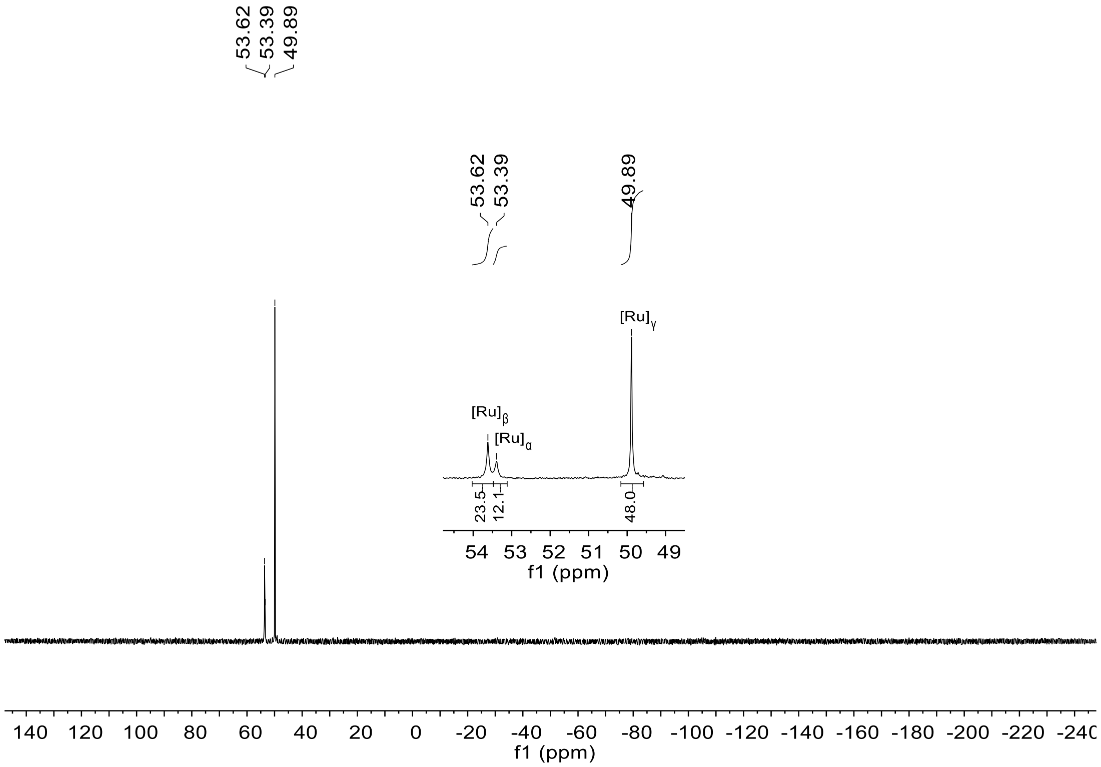

Figure S45. ^{31}P NMR spectrum of **37**.

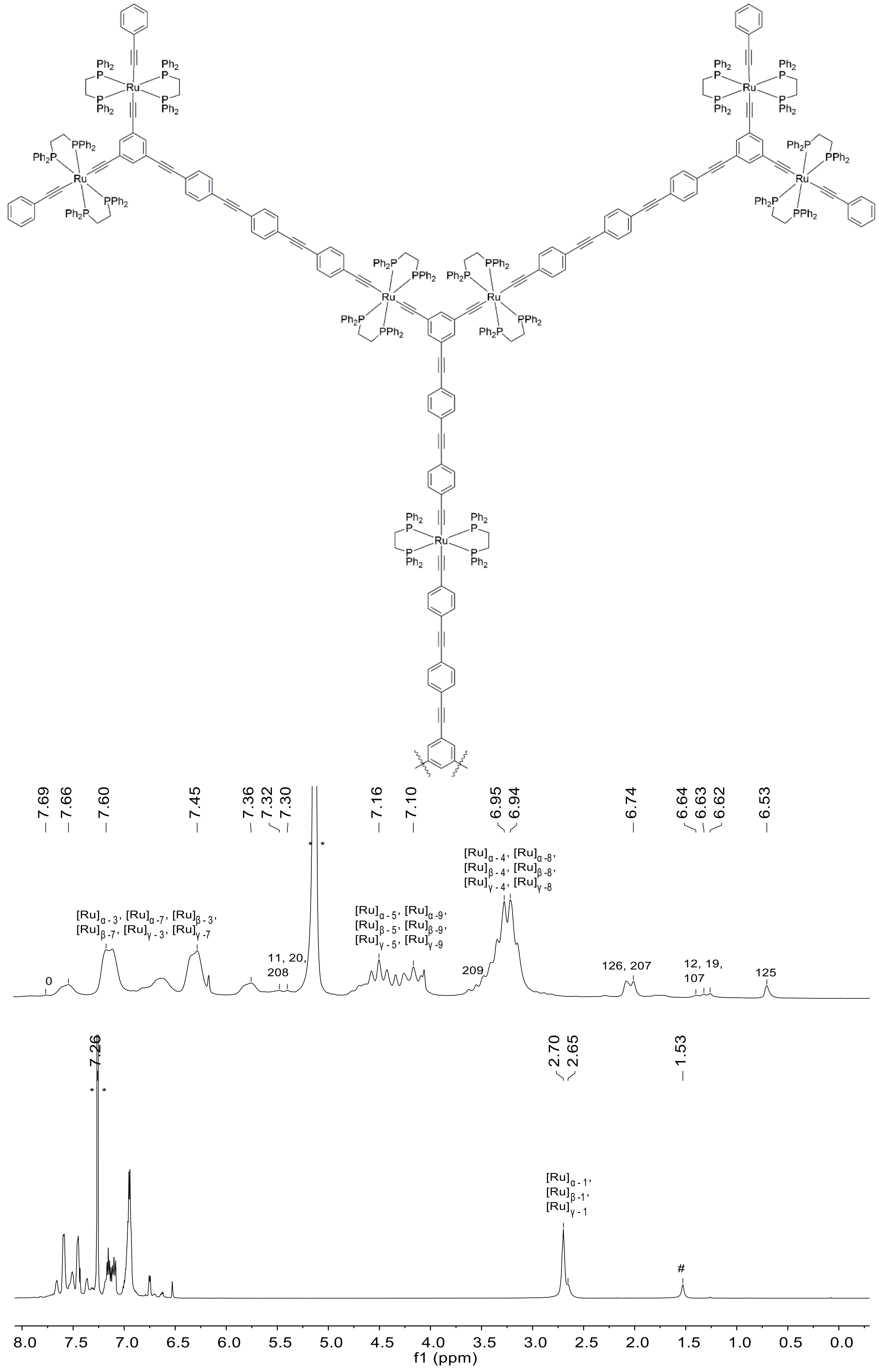

Figure S46. ^{1}H NMR spectrum of **2G$_{22,03,01}$**. The peak marked as * * corresponds to the residual $CHCl_3$ signal. The peak marked as # corresponds to the residual water signal.

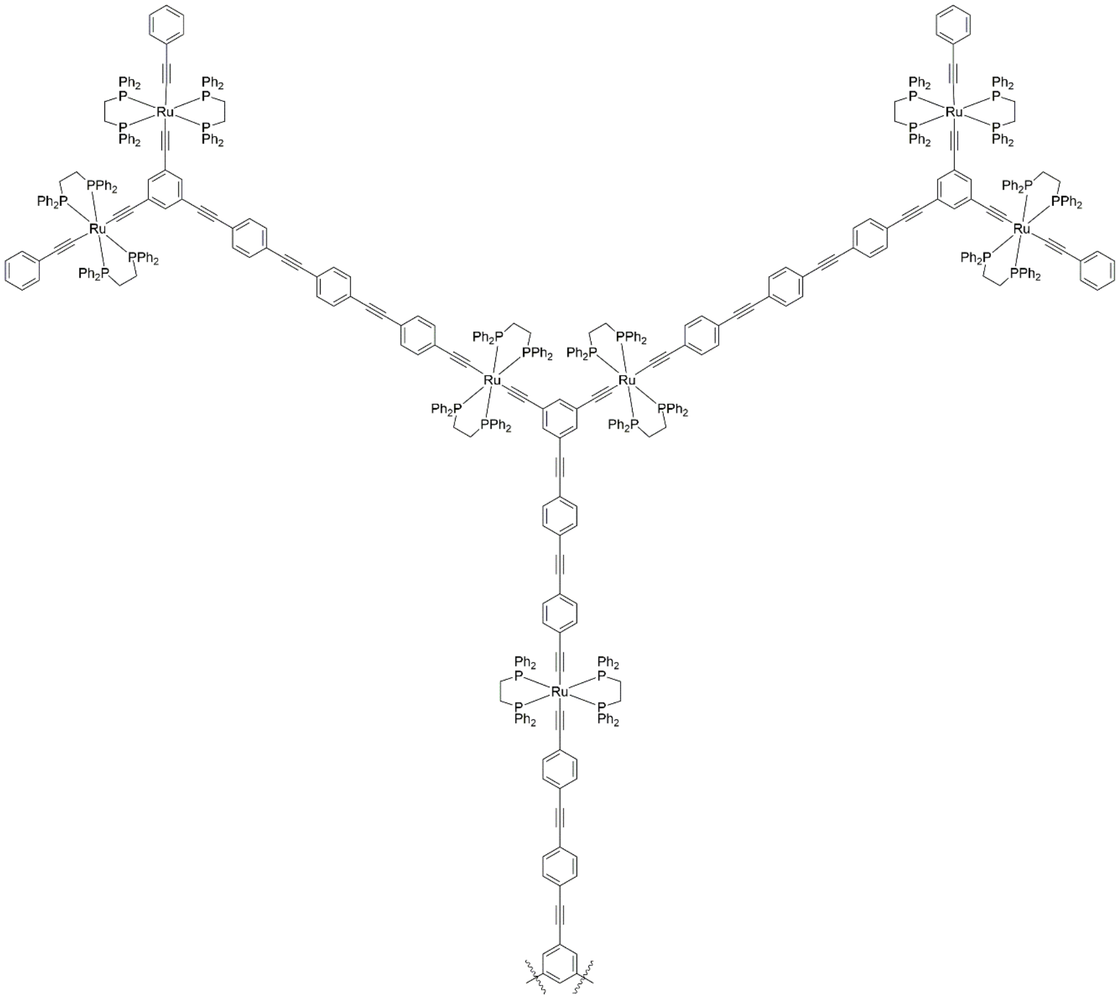

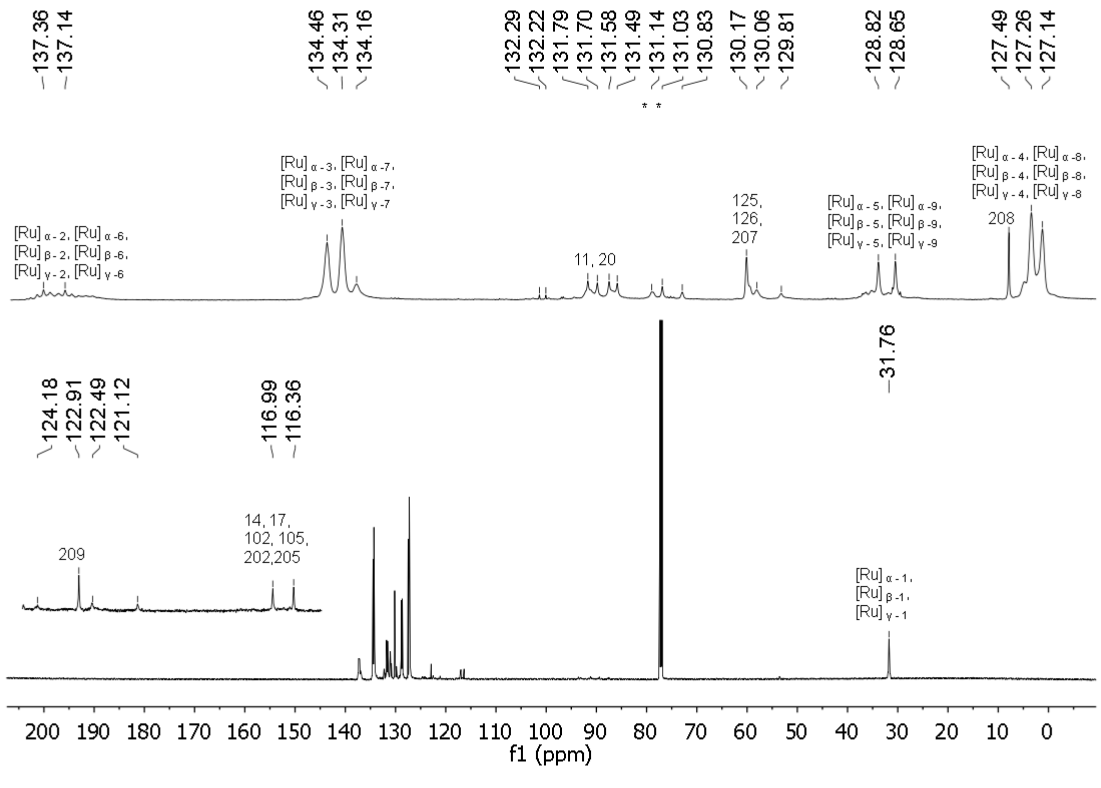

Figure S47. ^{13}C NMR spectrum of **$2G_{22,03,01}$** The peak marked as * * corresponds to $CDCl_3$.

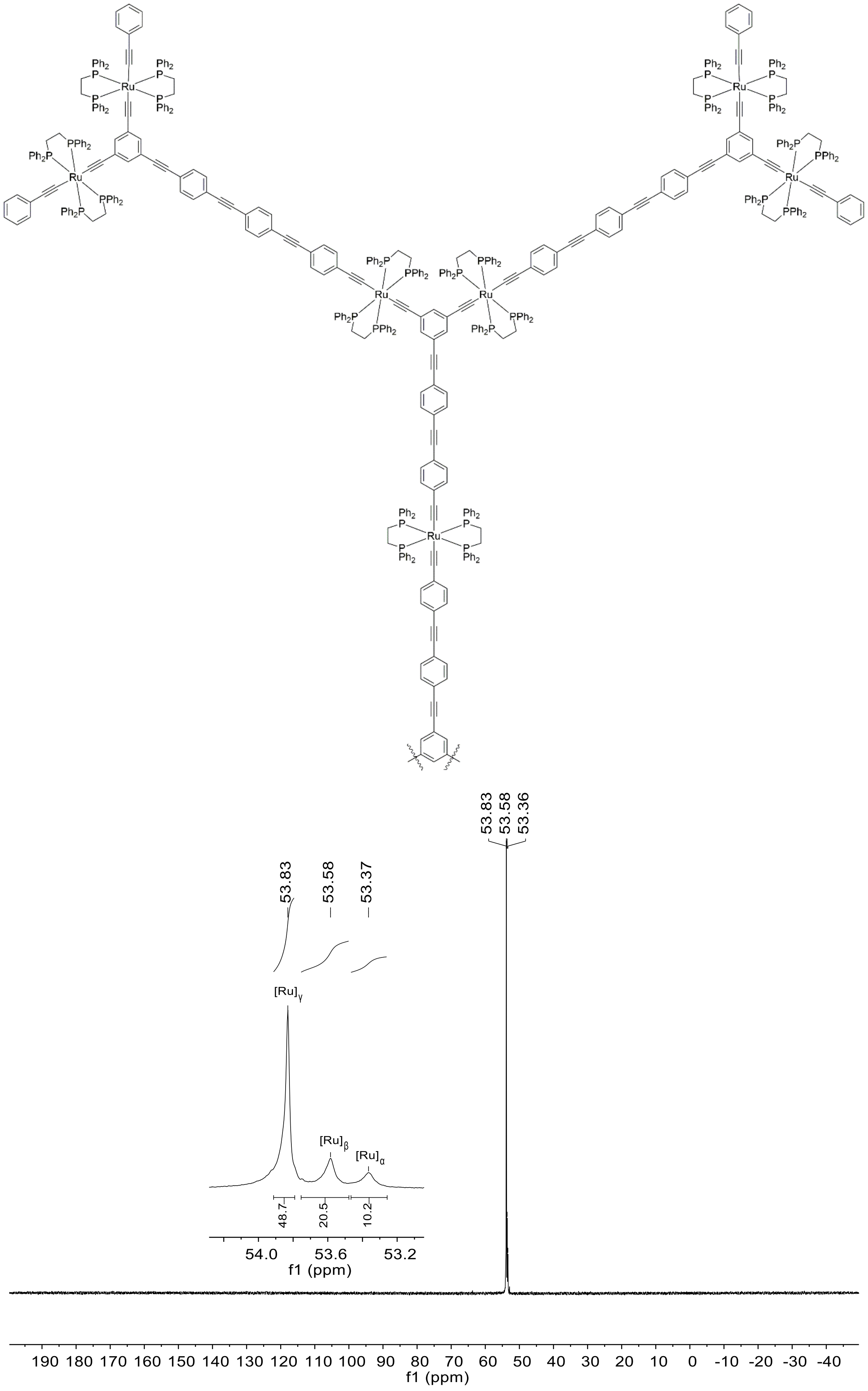

Figure S48. ^{31}P NMR spectrum of **$2G_{22,03,01}$**.

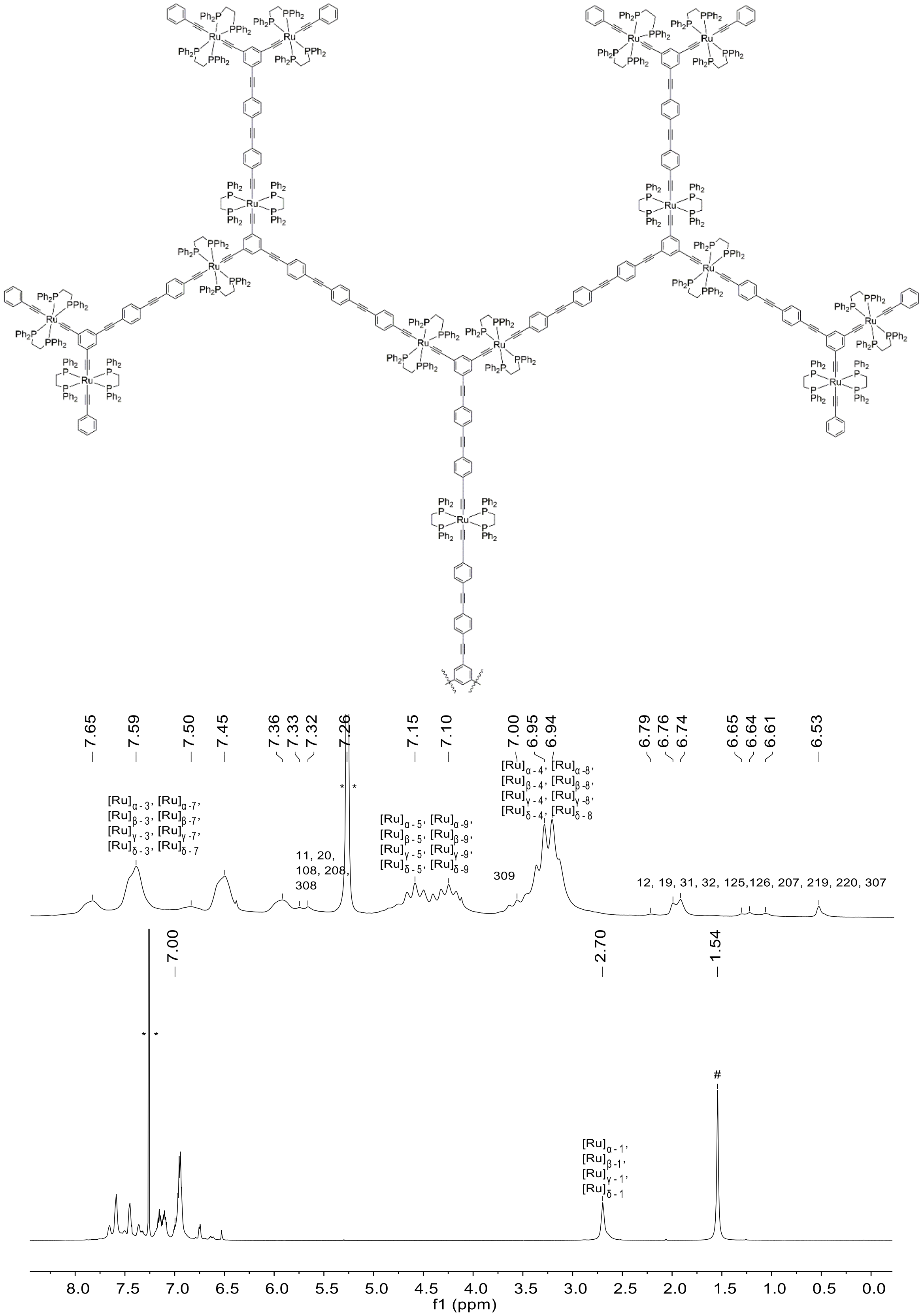

Figure S49. ^{1}H NMR spectrum of **3G$_{22,03,02,01}$**. The peak marked as * * corresponds to the residual $CHCl_3$ signal. The peak marked as # corresponds to the residual water signal.

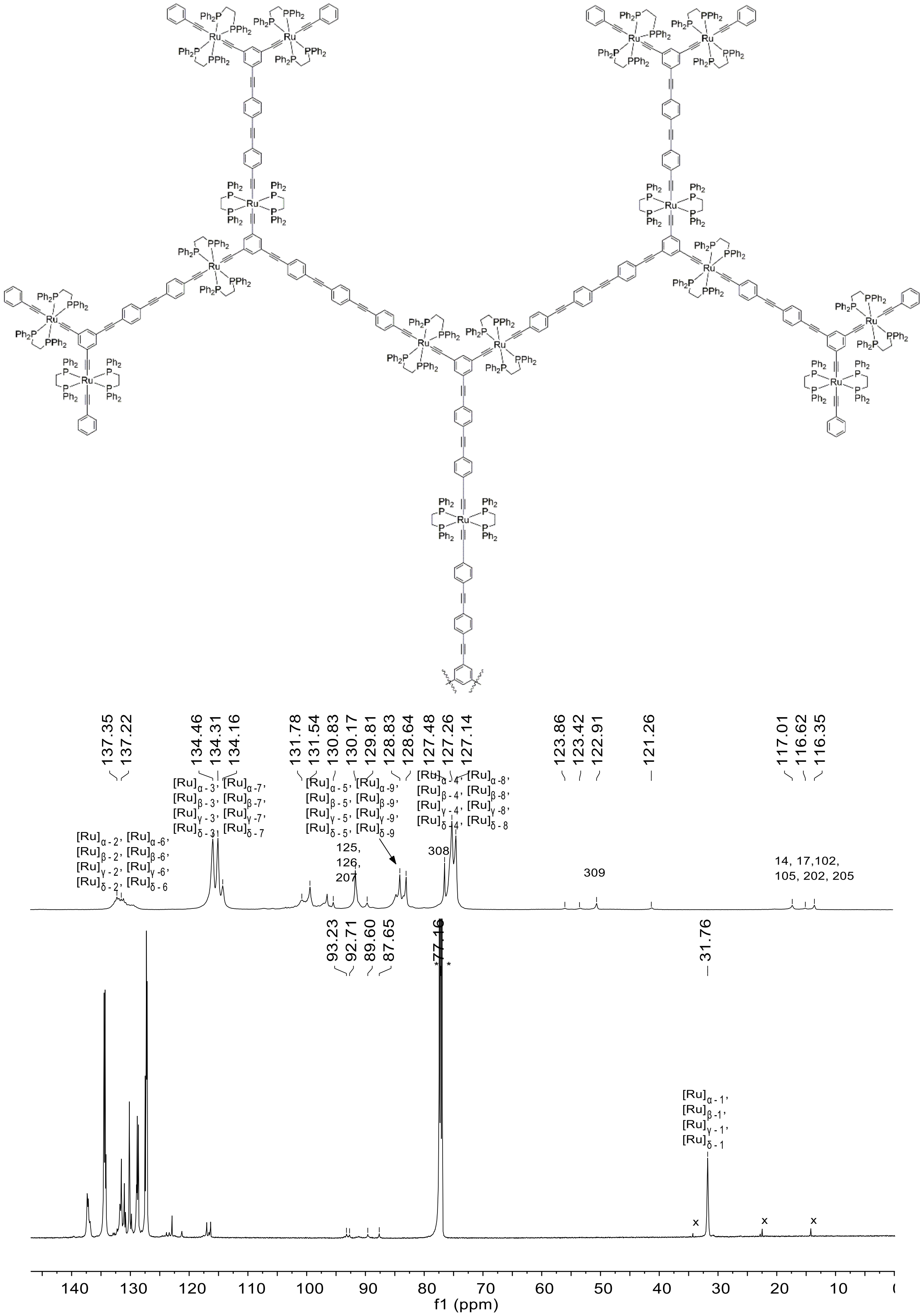

Figure S50. ^{13}C NMR spectrum of **3G$_{22,03,02,01}$**. The peak marked as * * corresponds to $CDCl_3$. The peaks marked as x corresponds to *n*-pentane.

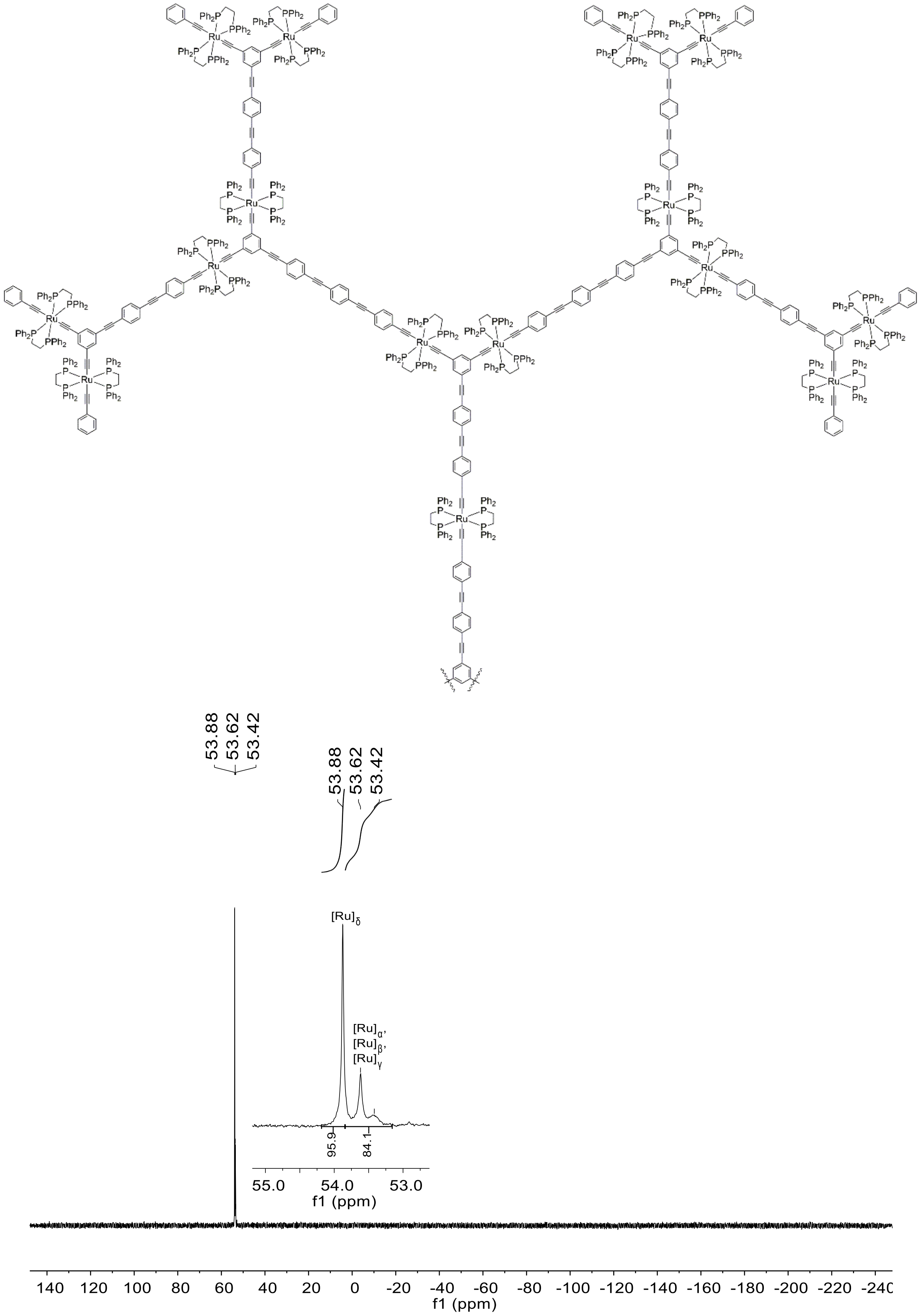

Figure S51. ^{31}P NMR spectrum of $\mathbf{3G_{22,03,02,01}}$.

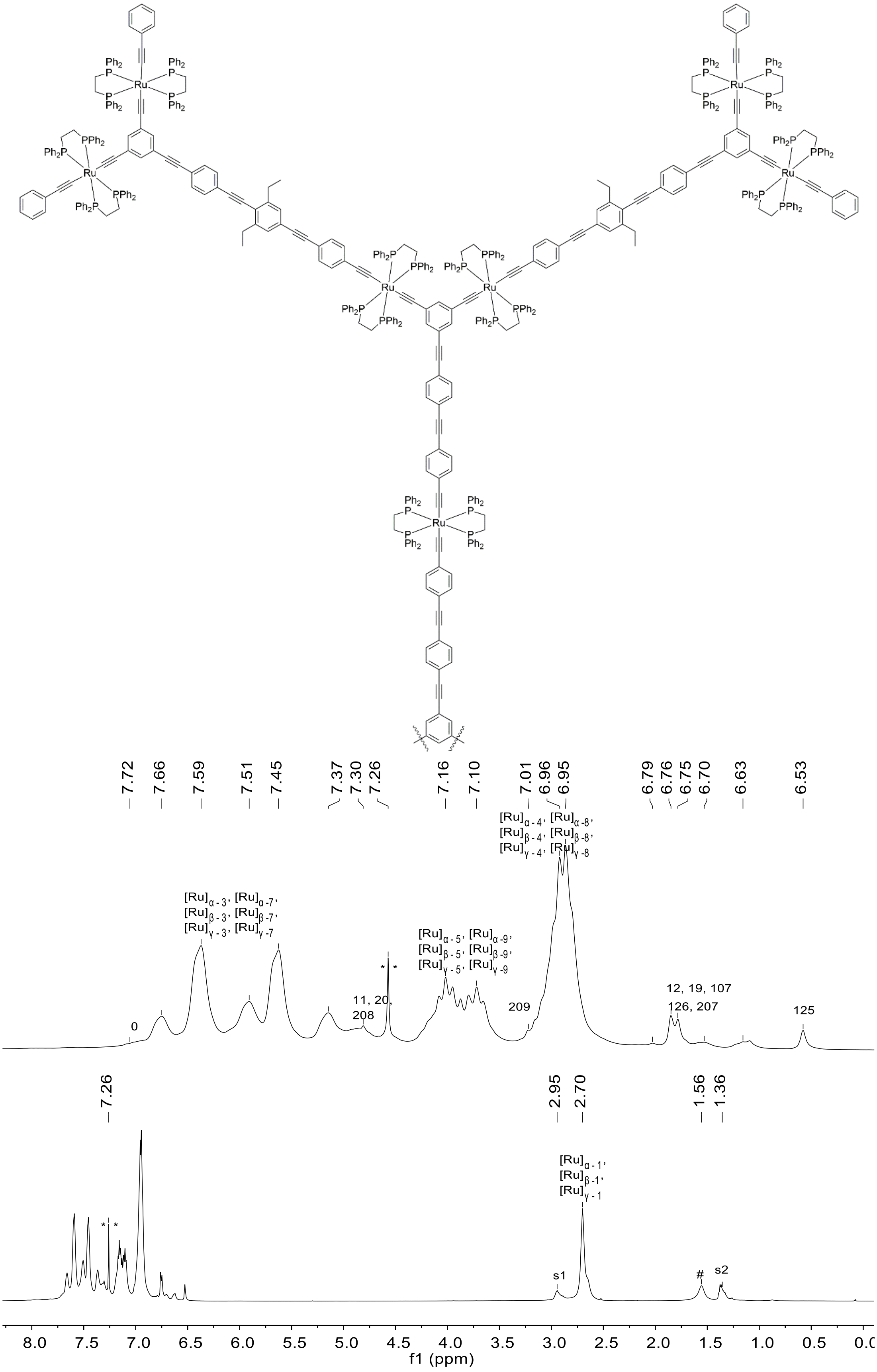

Figure S52. ^{1}H NMR spectrum of **2G$_{22,03,01}$-s**. The peak marked as * * corresponds to the residual $CHCl_3$ signal. The peak marked as # corresponds to the residual water signal.

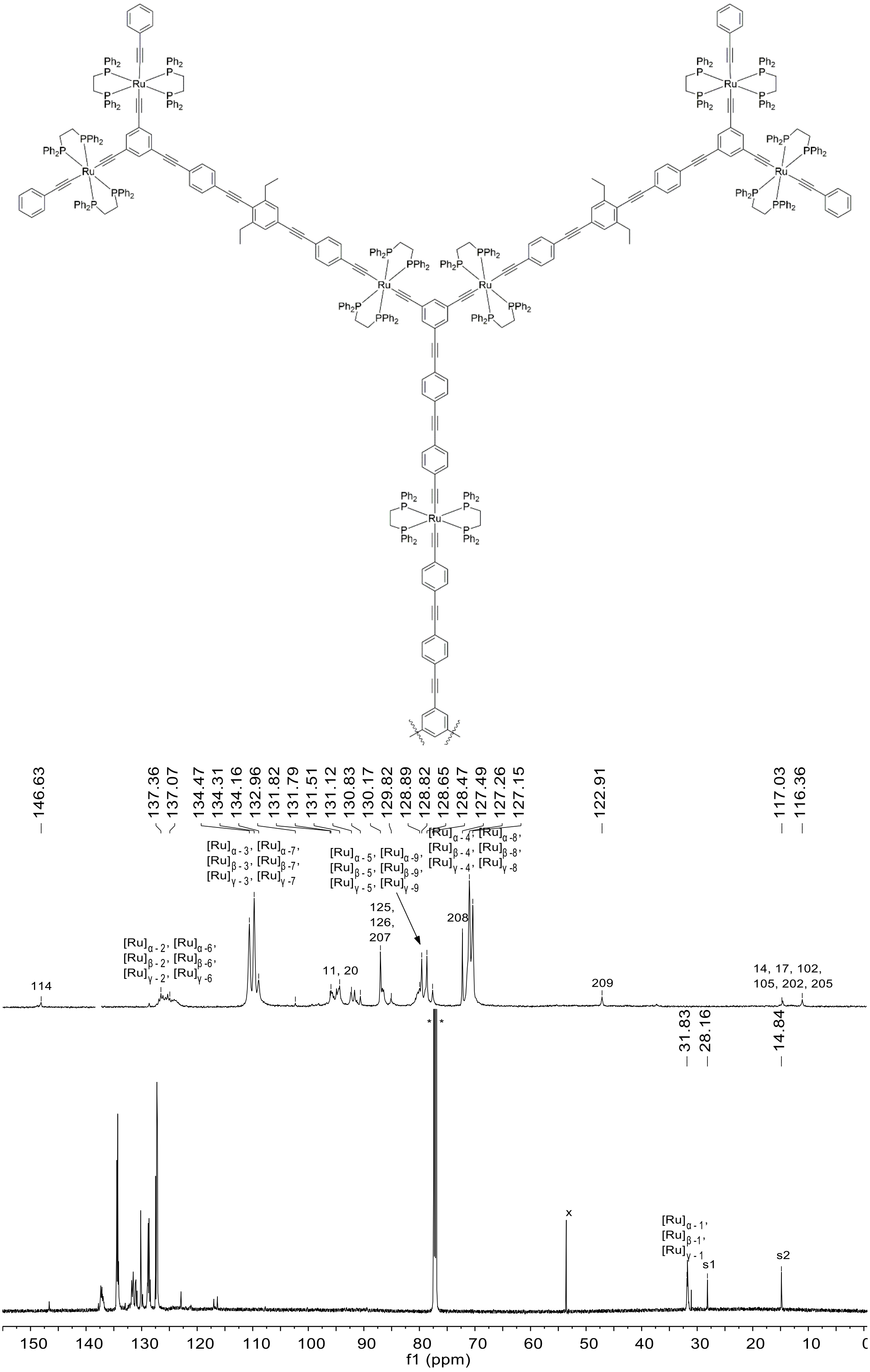

Figure S3. ^{13}C NMR spectrum of **$2G_{22,03,01}$-s**. The peak marked as * * corresponds to $CDCl_3$. The peak marked as x corresponds to the residual CH_2Cl_2 signal.

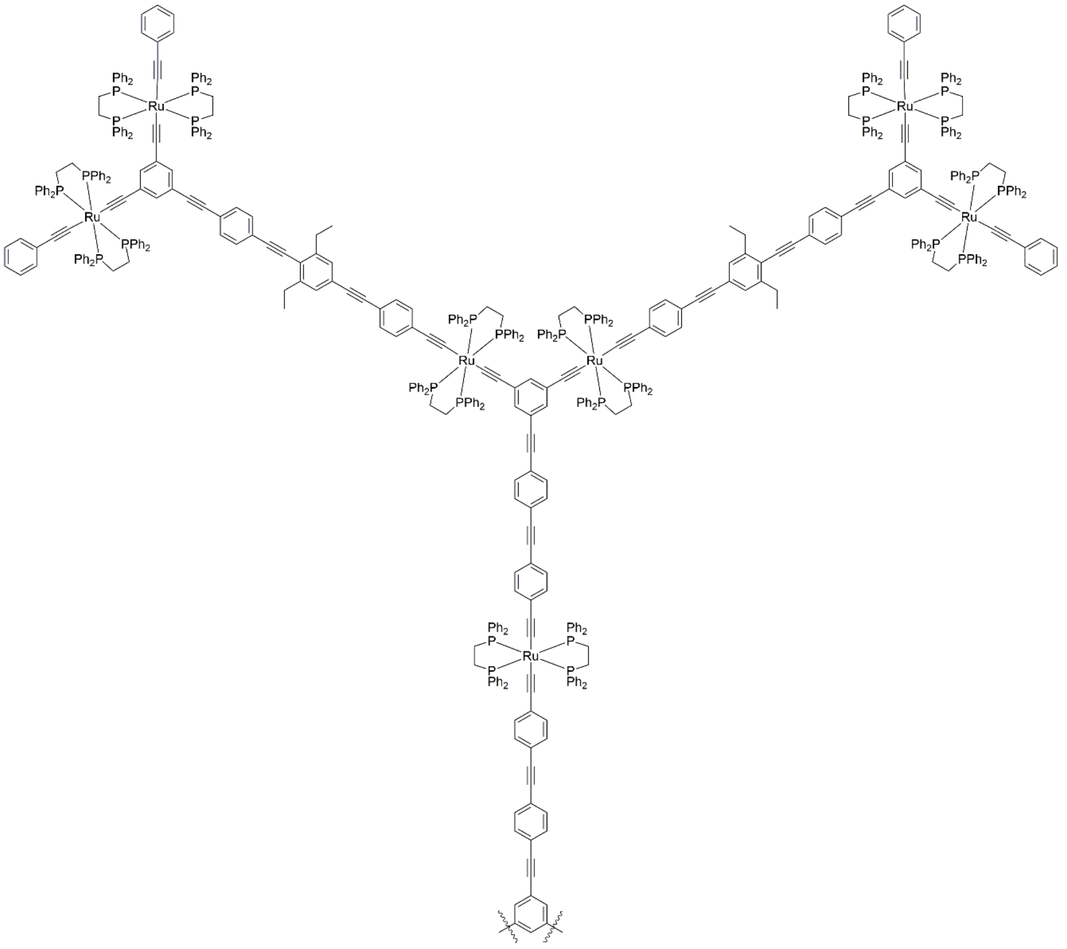

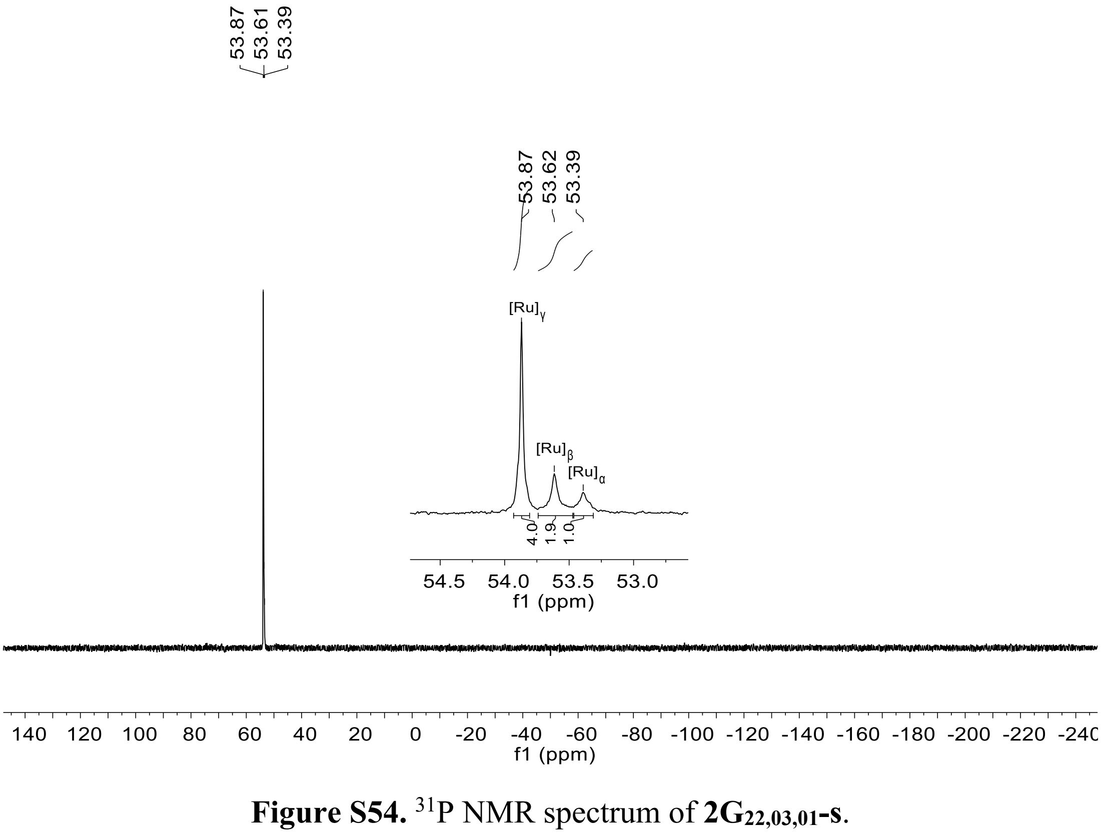

Figure S54. ^{31}P NMR spectrum of $\mathbf{2G_{22,03,01}}$**-s**.

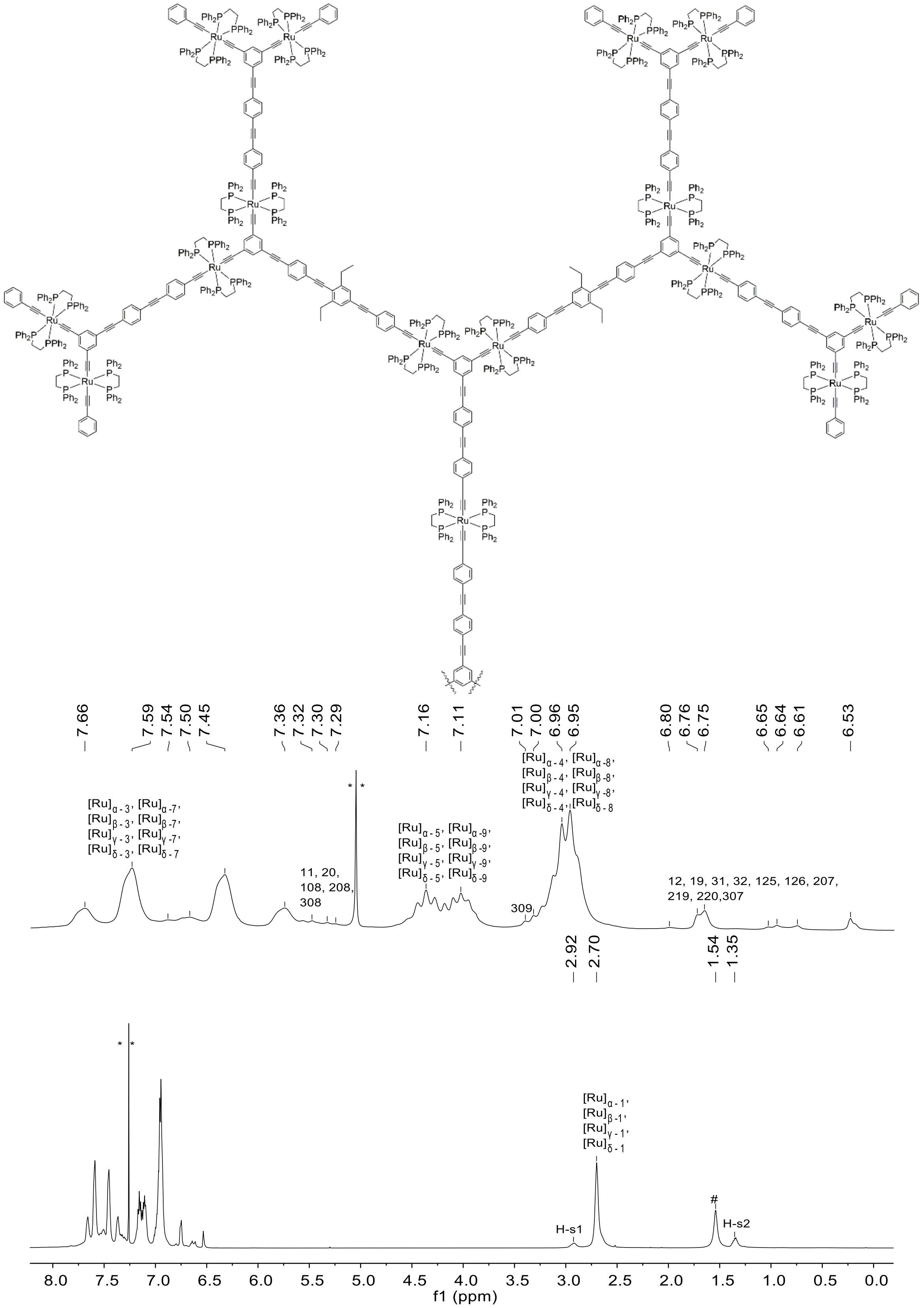

Figure S55. ^{1}H NMR spectrum of **3G$_{22,03,02,01}$-s**. The peak marked as * * corresponds to the residual $CHCl_3$ signal. The peak marked as # corresponds to the residual water signal.

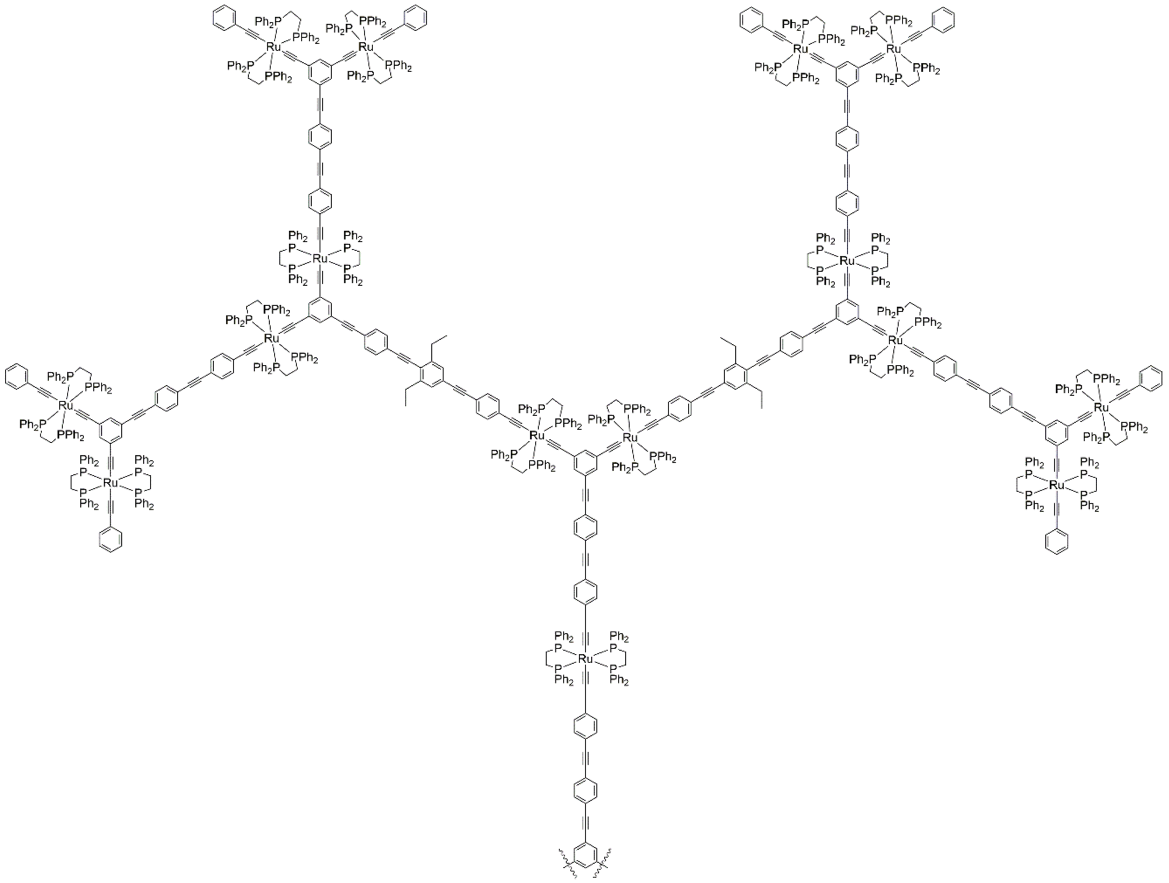

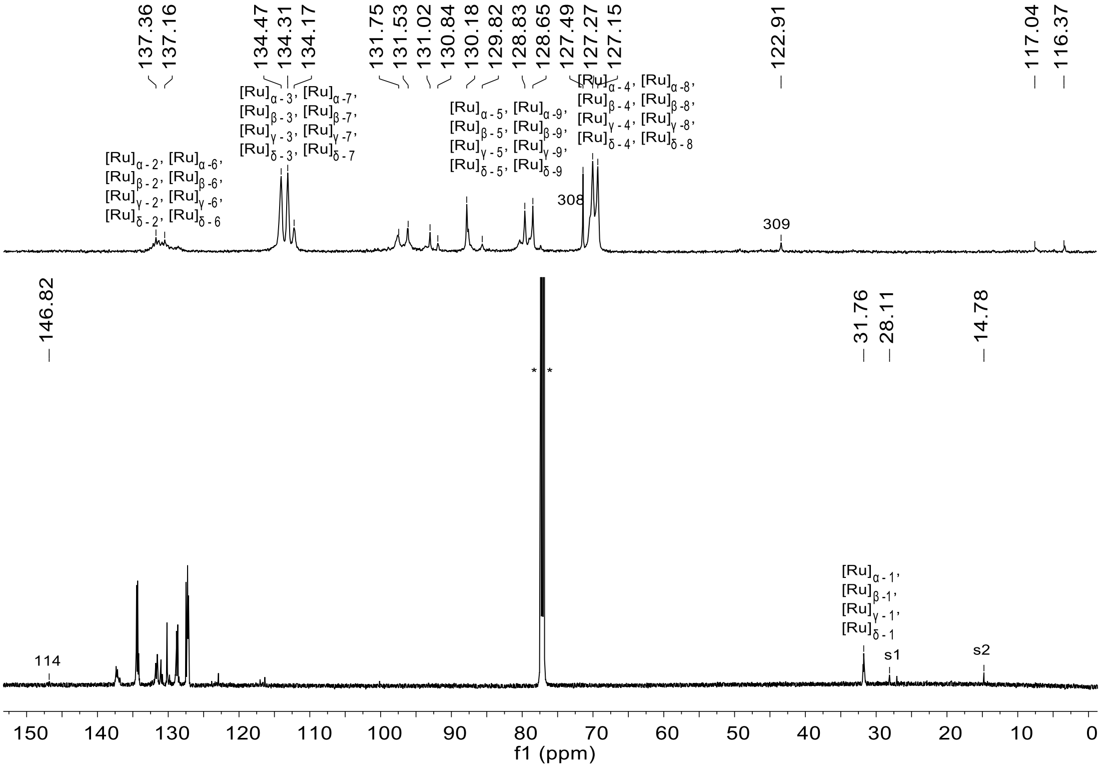

Figure S56. ^{13}C NMR spectrum of **3G$_{22,03,02,01}$-s**. The peak marked as * * corresponds to $CDCl_3$.

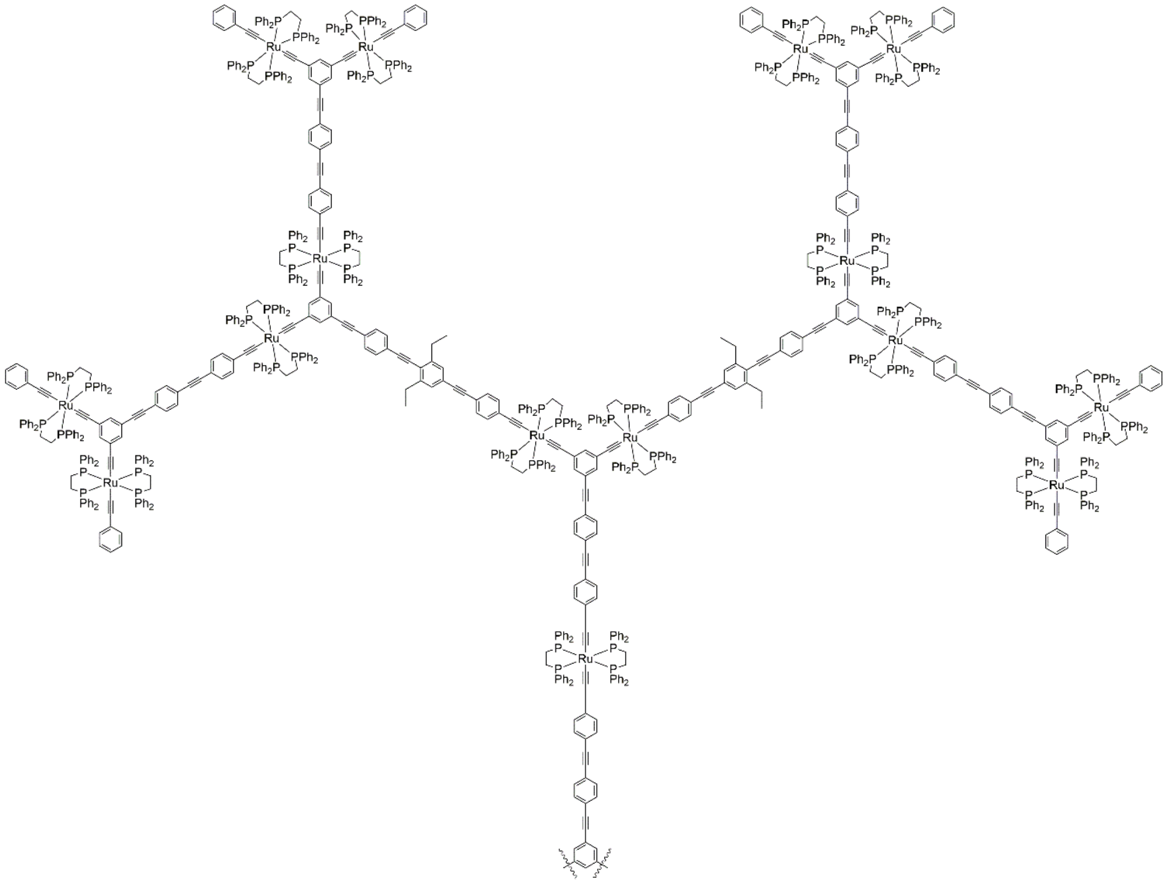

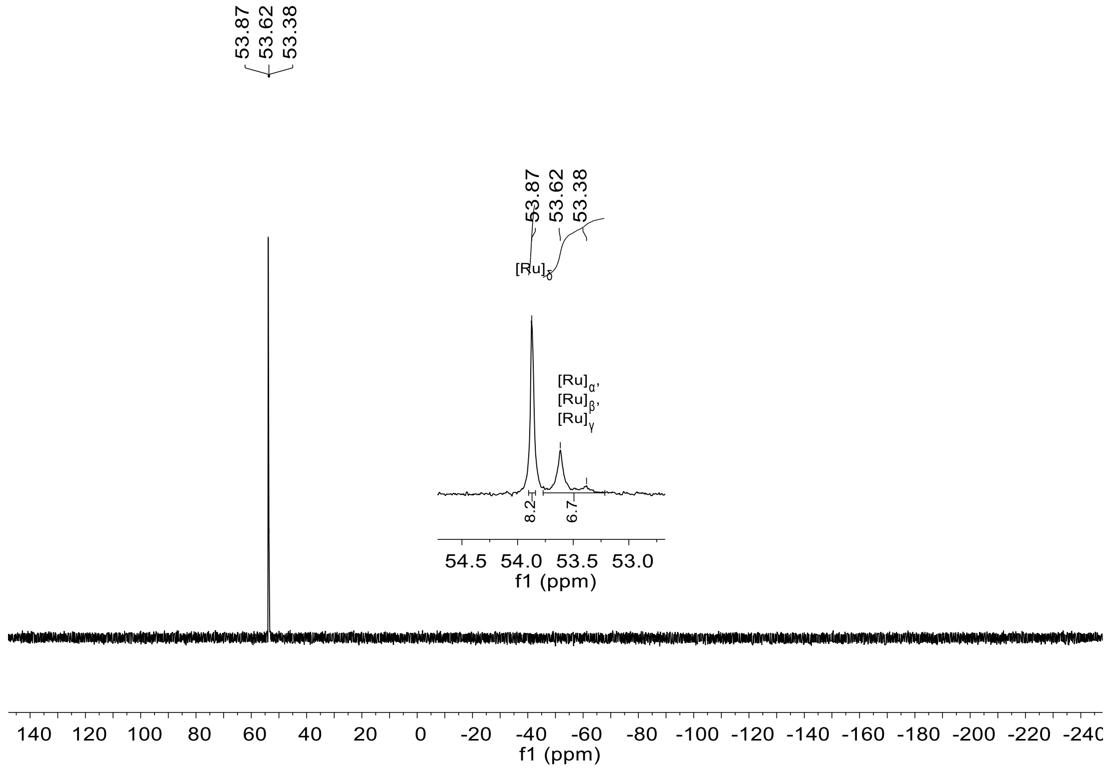

Figure S57. ^{31}P NMR spectrum of **3G$_{22,03,02,01}$-s**.

DOSY 2D NMR Spectra of Ruthenium Dendrimers.

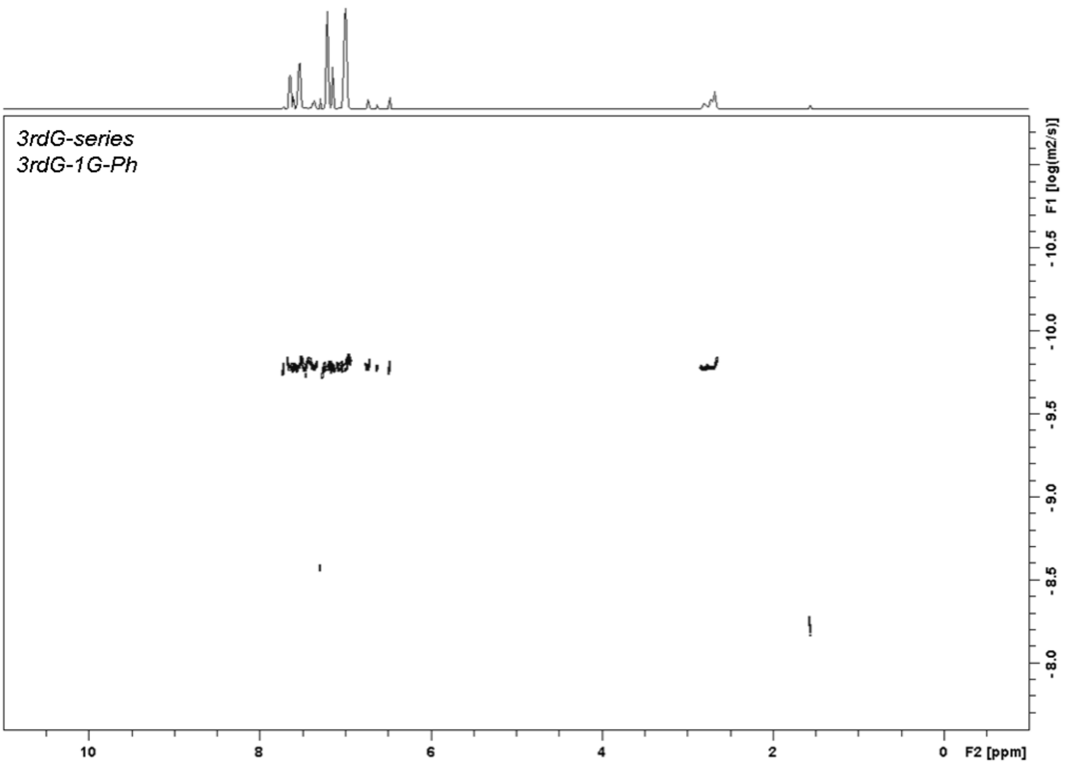

Figure S58. DOSY spectrum of $\mathbf{1G_{22,01}}$.

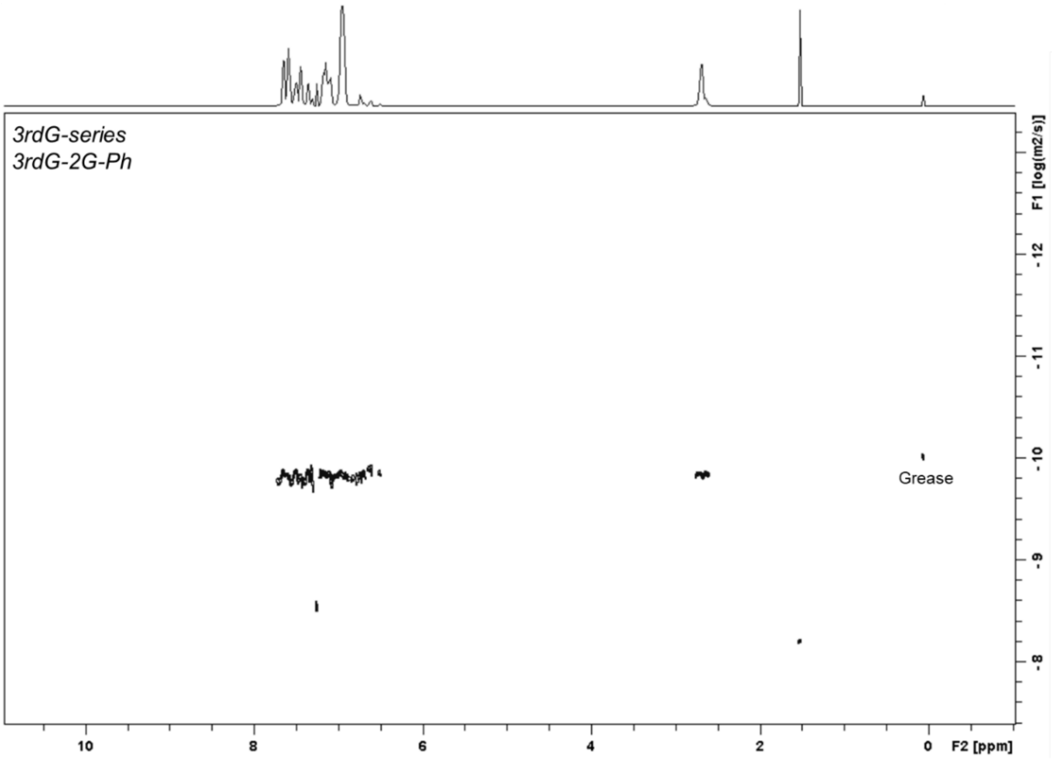

Figure S59. DOSY spectrum of $\mathbf{2G_{22,03,01}}$.

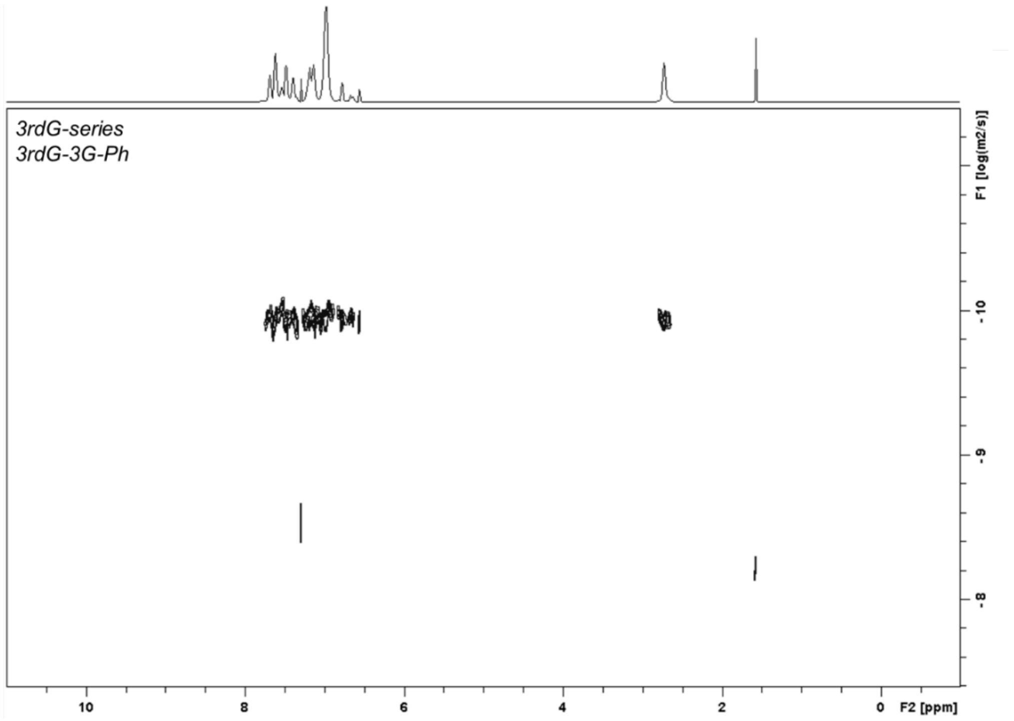

Figure S60. DOSY spectrum of **3G**$_{22,03,02,01}$.

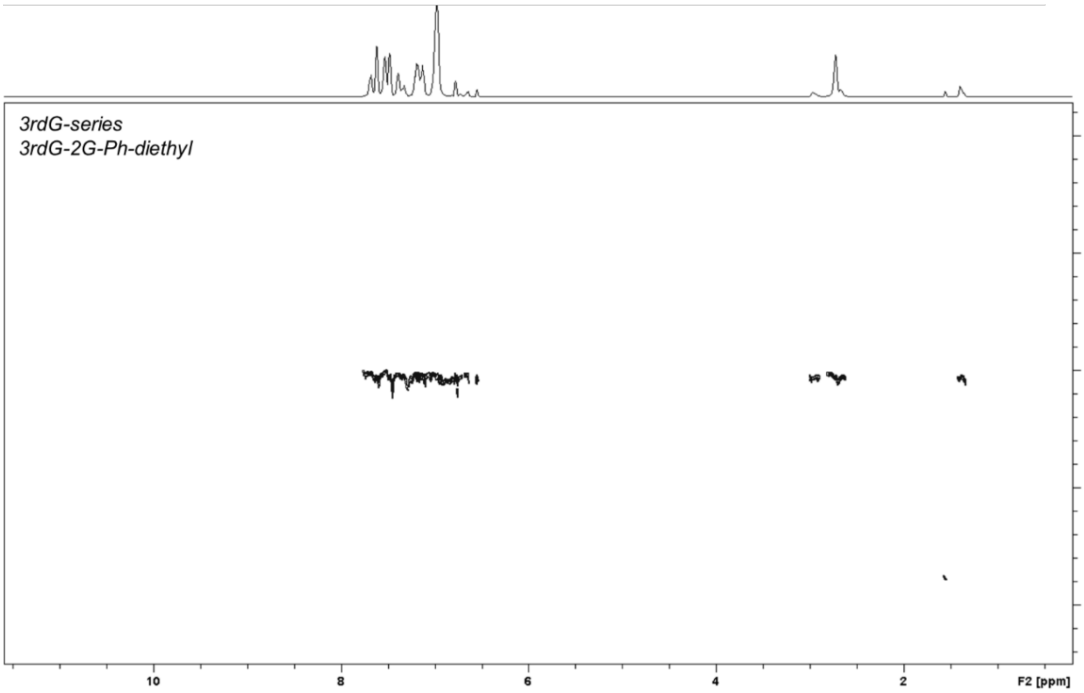

Figure S61. DOSY spectrum of **2G**$_{22,03,01}$**-s**.

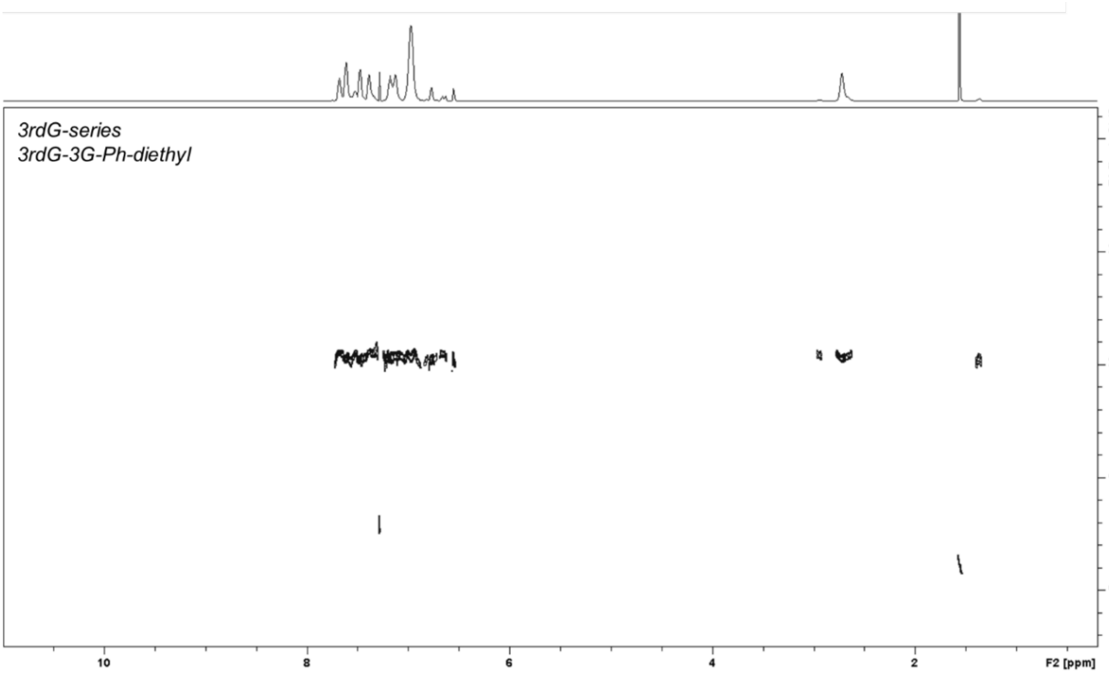

Figure S62. DOSY spectrum of **$3G_{22,03,02,01}$-s**.

MS, TEM and Size-exclusion Studies of Ruthenium Dendrimers

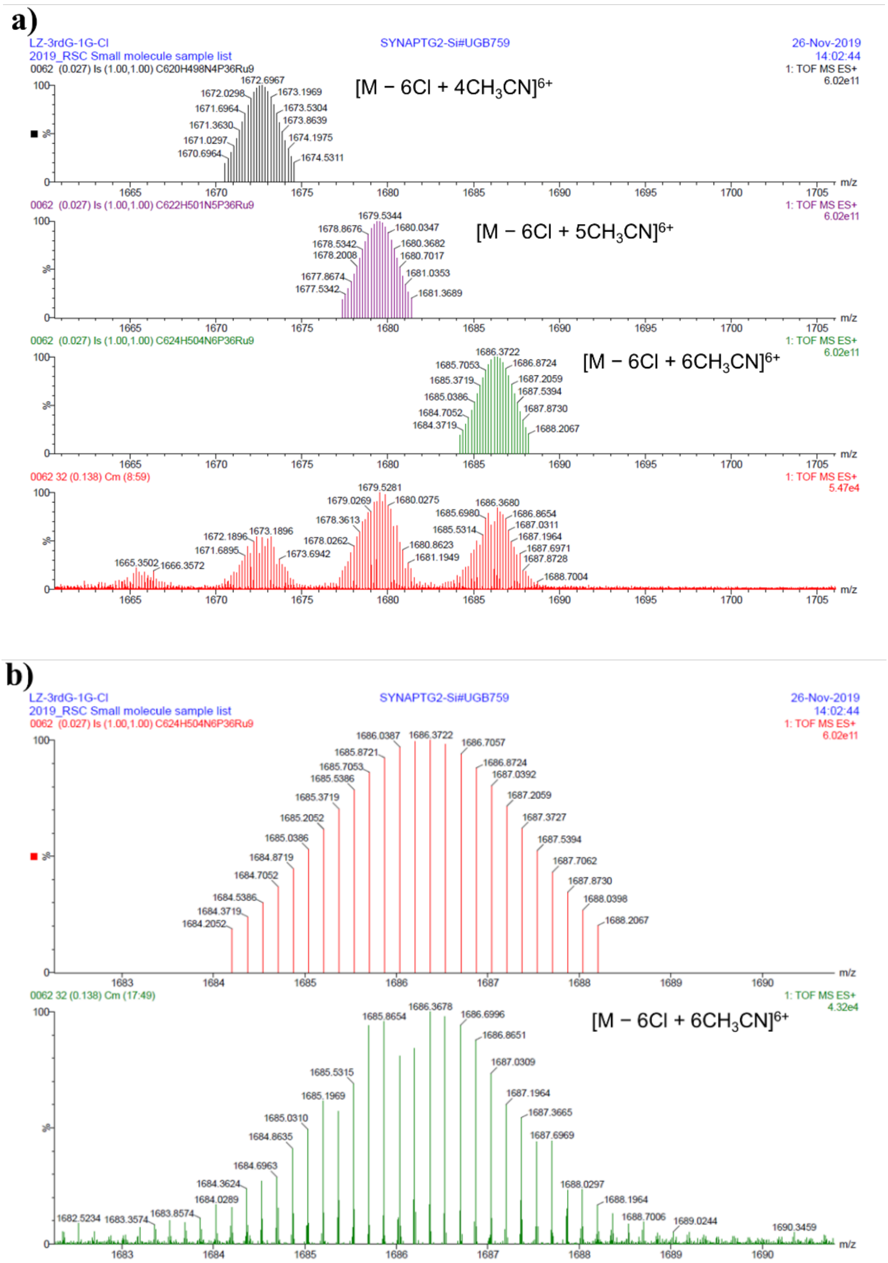

Figure S63. a) HRMS spectrum of the first-generation dendrimer **32**; b) Theoretical (top) and experimental (bottom) ESI-TOF-MS spectra of dendrimer **32**.

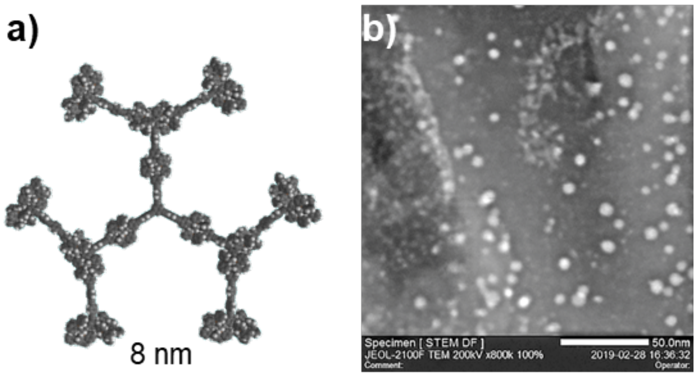

Figure S64. a) Molecular model of the second-generation ruthenium alkynyl dendrimer $\mathbf{2G_{22,02,01}}$; b) TEM image of dendrimer $\mathbf{2G_{22,02,01}}$. The diameter distribution of the sample ranges between 5-8 nm.

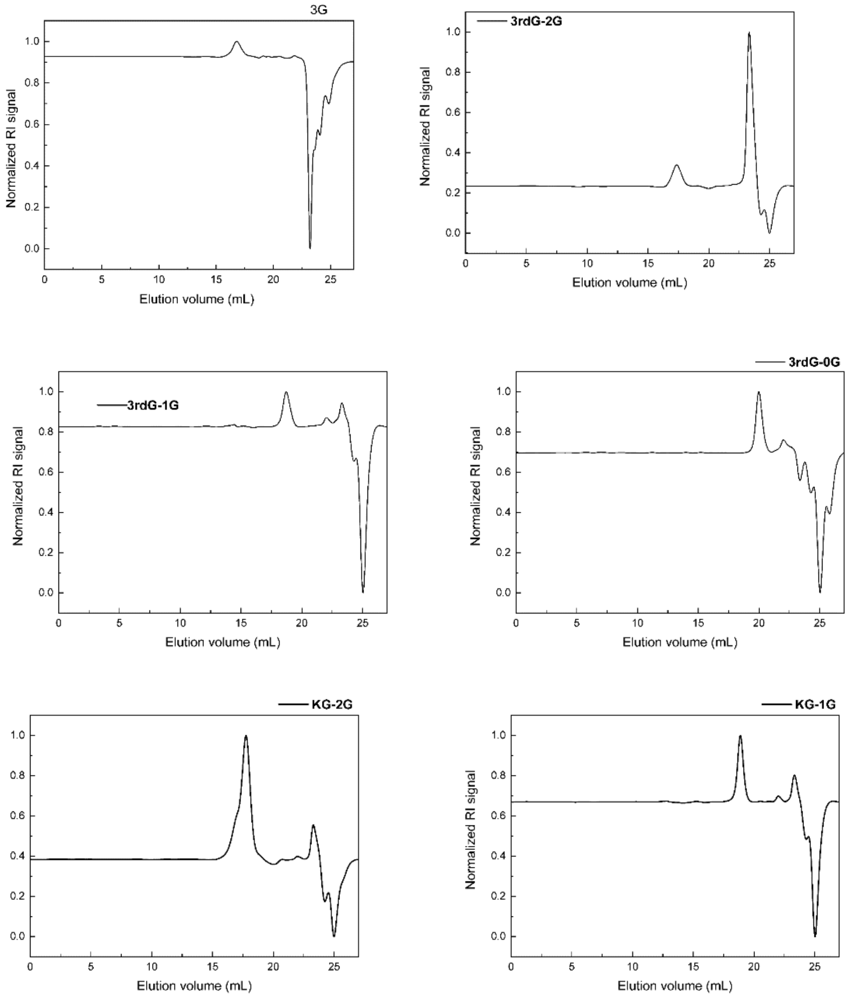

Figure S65. SEC traces of ruthenium dendrimers of various generations. (All dendrimers demonstrated a single dominant peak before solvent elution. In addition, with the increment

of generation, 0G-3G displayed a decrease of elution volume from 20.0 mL (**0G$_{21}$**) to 18.7 mL (**1G$_{22,01}$**), to 17.4 mL (**2G$_{22,03,01}$**), and to 16.8 mL (**3G$_{22,03,02,01}$**), thus revealing the size increase of the obtained dendrimers with the growth of generation. Also, to compare the dendrimers of the same generation, the elution volume of **2G$_{12,02,01}$** (17.8 mL) is larger than **2G$_{22,03,01}$** (17.4 mL), and **1G$_{12,01}$** (18.9 mL) is larger than **1G$_{22,01}$** (18.7), indicating the former has a relatively smaller structure compared to the latter of the same generation.

UV-Vis and Z-scan Studies of Ruthenium Dendrimers

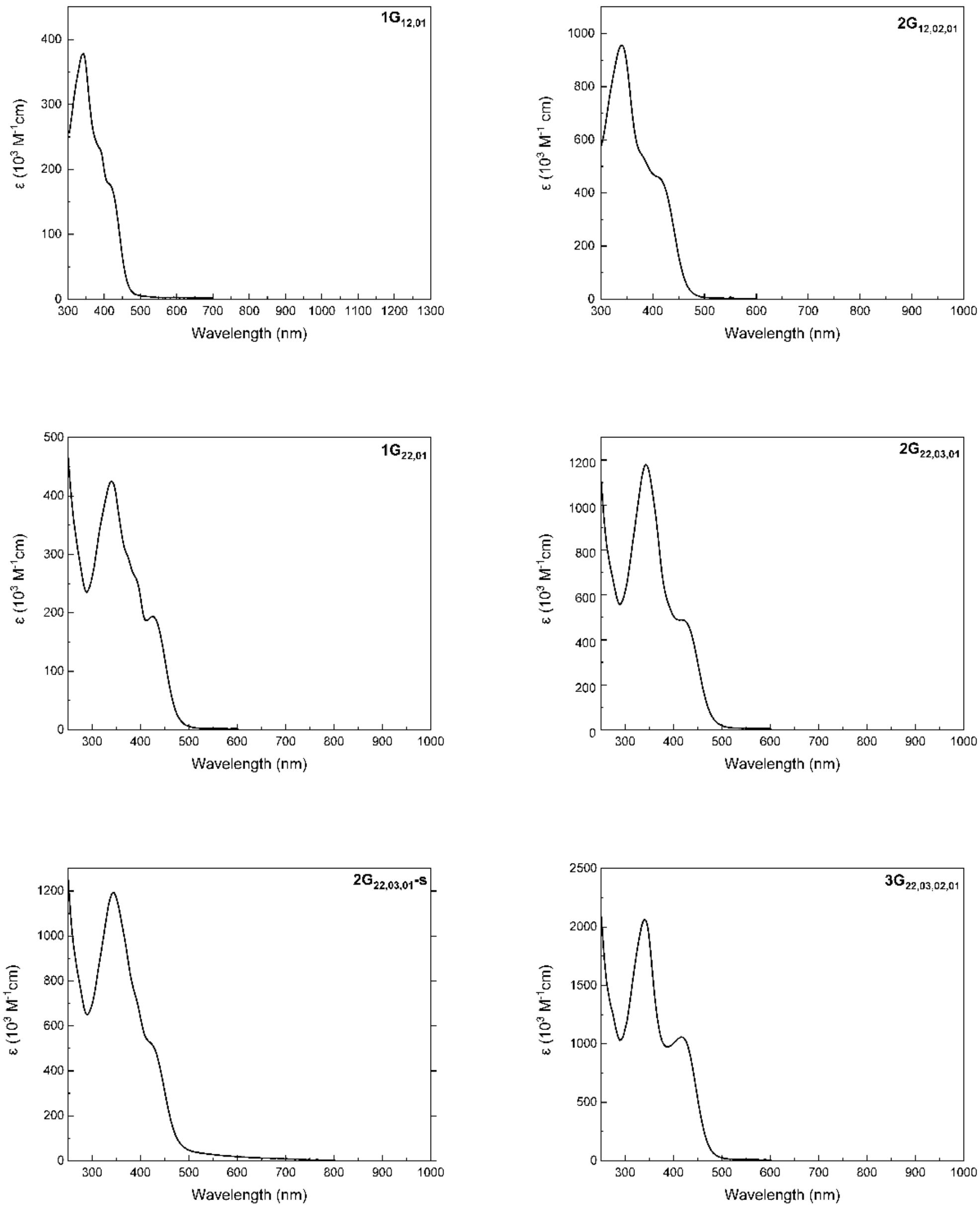

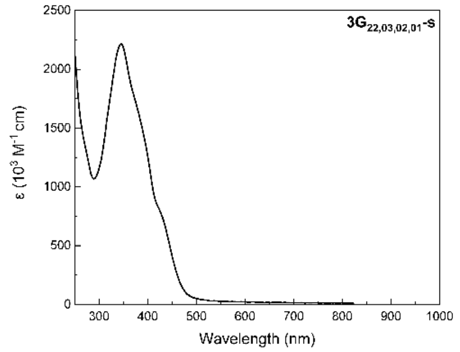

Figure S66. UV-vis spectra of ruthenium dendrimers of various generations.

Table S1. Linear optical absorption and nonlinear optical absorption cross-section maxima.[a]

Complex	$\lambda_{1,\,max}$[b] [ε][c]	$\lambda_{2,\,max}$[b] [ε][c]	σ_2[d] (λ_{max})[b]	σ_3[e] (λ_{max})[b]	σ_4[f] (λ_{max})[b]	σ_5[g] (λ_{max})[b]
**3G$_{22,03,02,01}$-s**	346 [222]	424 [81]	111,500 (700)	23,200 (1250)	7300 (1650)	500 (2050)
			50,600 (900)			
**3G$_{22,03,02,01}$**	340 [206]	418 [106]	113,200 (725)	22,200 (1200)	5850 (1650)	350 (2100)
			45 900 (900)			
**2G$_{22,03,01}$-s**	343 [119]	421 [52]	42,450 (750)	15,000 (1250)	3700 (1650)	170 (2100)
			16,000 (875)			
**2G$_{22,03,01}$**	343 [118]	415 [49]	43,600 (700)	13,750 (1250)	2950 (1650)	100 (2160)
			20,000 (900)			
**1G$_{22,01}$**	340 [43]	427 [19]	13,700 (725)	4800 (1200)	1200 (1650)	0
			8800 (900)			
**0G$_{21}$**[h]	[i]	422 [12.6]	3200 (900)	2300 (1200)	0	0
**2G$_{12,02,01}$**	340 [96]	409 [46]	34,700 (750)	7950 (1250)	2700 (1650)	0
			18,000 (900)			
**1G$_{12,01}$**	343 [38]	412 [18]	14,000 (725)	2500 (1250)	900 (1650)	0
			6300 (875)			
**0G$_{11}$**[j]	[i]	412 [11.6]	1500 (650), 370 (810)	100 (1240)	0	0

[a] CH_2Cl_2. [b] nm. [c] 10^4 L mol^{-1} cm^{-1}. [d] GM = 10^{-50} cm^4 s photon^{-1}. [e] 10^{-80} cm^6 s^2 photon^{-2}. [f] 10^{-110} cm^8 s^3 photon^{-3}. [g] 10^{-140} cm^{10} s^4 photon^{-4}. [h] Ref. 21. [i] not reported. [j] Ref. 80.

Table S2. Comparison of the molecular weight- and effective-number-of-π-electrons-scaled NLO parameters at local maxima for dendrimers.[a]

Complex	σ_2/M [c], σ_2/N_{eff}^2 [d] (λ_{max})[b]	σ_3/M [h], σ_3/N_{eff}^3 [e] (λ_{max})[b]	σ_4/M [i], σ_4/N_{eff}^4 [f] (λ_{max})[b]	σ_5/M [j], σ_5/N_{eff}^5 [g] (λ_{max})[b]
**3G22,03,02,01-s**	2.15, 7.97 (700)<br>0.98, 3.62 (900)	0.45, 0.014 (1250)	0.14, 3.73 (1650)	0.0097, 2.16 (2050)
**3G22,03,02,01**	2.20, 8.09 (725)<br>0.89, 3.28 (900)	0.43, 0.013 (1200)	0.11, 2.99 (1650)	0.0068, 1.51 (2100)
**2G22,03,01-s**	1.71, 5.21 (750)<br>0.64, 1.96 (875)	0.60, 0.020 (1250)	0.15, 5.56 (1650)	0.0068, 2.83 (2100)
**2G22,03,01**	1.78, 5.35 (700)<br>0.82, 2.45 (900)	0.56, 0.019 (1250)	0.12, 4.44 (1650)	0.0041, 1.67 (2100)
**1G22,01**	1.31, 4.77 (725)<br>0.85, 3.06 (900)	0.46, 0.031 (1200)	0.11, 1.45 (1650)	0, 0
**0G21**	0.85, 2.30 (900)	0.61, 0.044 (1200)	0, 0	0, 0
**2G12,02,01**	1.47, 6.83 (750)<br>0.76, 3.54 (900)	0.34, 0.022 (1250)	0.11, 1.04 (1650)	0, 0
**1G12,01**	1.38, 6.64 (725)<br>0.62, 2.98 (875)	0.25, 0.026 (1250)	0.09, 2.03 (1650)	0, 0
**0G11**	0.43, 2.40 (650)<br>0.11, 0.59 (810)	0.029, 0.0064 (1240)	0, 0	0, 0

[a] CH_2Cl_2 solvent, N_{eff} = 118.3 (**3G22,03,02,01-s**, **3G22,03,02,01**), 90.3 (**2G22,03,01-s**, **2G22,03,01**), 53.6 (**1G22,01**), 71.3 (**2G12,02,01**), 46.0 (**1G12,01**), 37.3 (**0G21**), 25.0 (**0G11**). [b] nm. [c] GM mol g^{-1}. [d] GM = 10^{-50} cm^4 s $photon^{-1}$. [e] 10^{-80} cm^6 s^2 $photon^{-2}$. [f] 10^{-110} cm^8 s^3 $photon^{-3}$. [g] 10^{-140} cm^{10} s^4 $photon^{-4}$. [h] 10^{-80} cm^6 s^2 $photon^{-2}$ mol g^{-1}. [i] 10^{-110} cm^8 s^3 $photon^{-3}$ mol g^{-1}. [j] 10^{-140} cm^{10} s^4 $photon^{-4}$ mol g^{-1}.

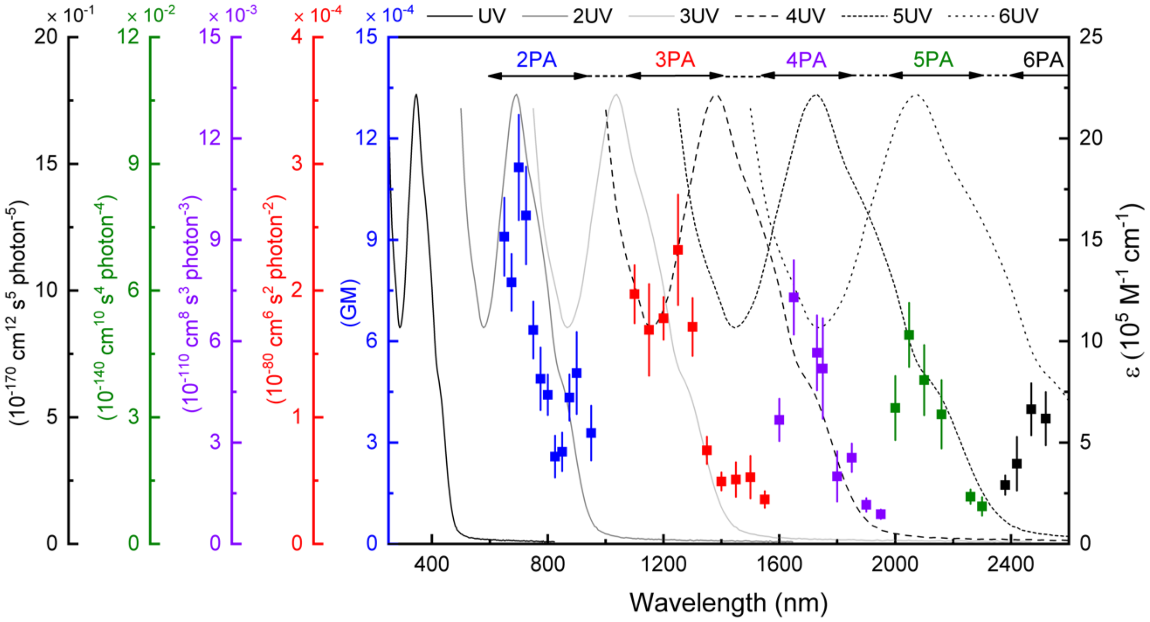

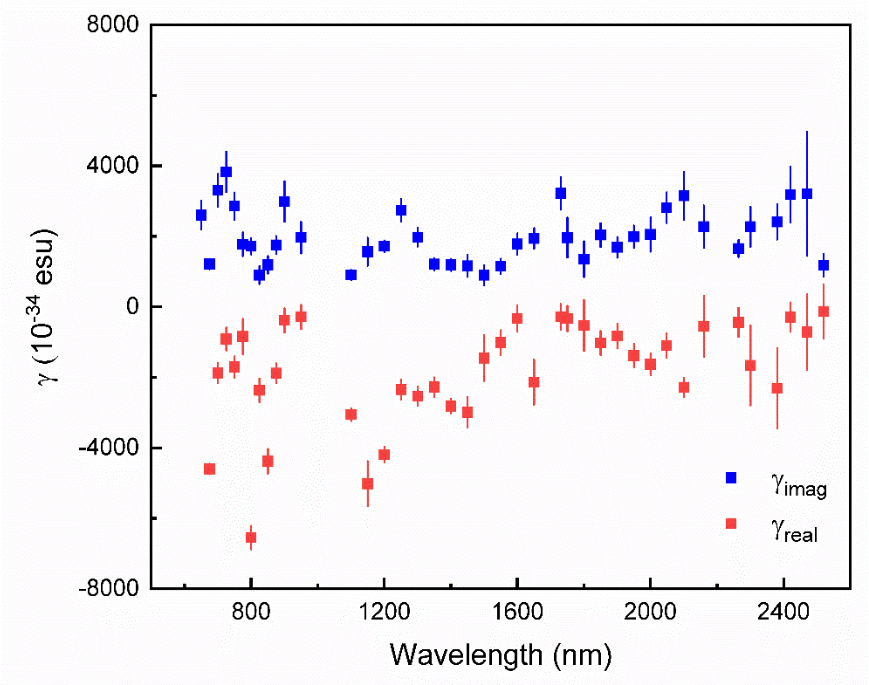

Figure S67. Wavelength dependence of the nonlinear absorption (top) and cubic NLO coefficients (bottom) of **3G$_{22,03,02,01}$-s**.

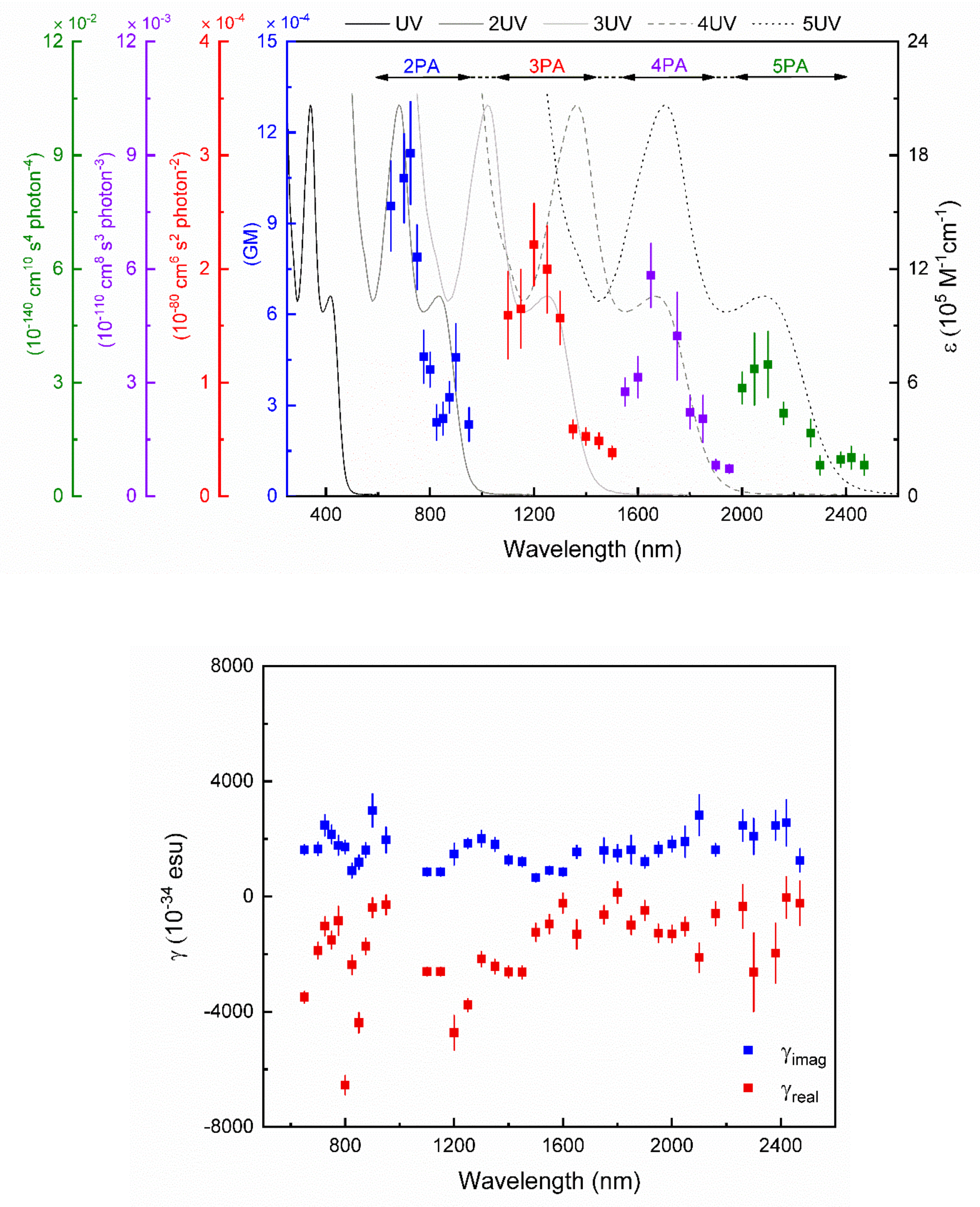

Figure S68. Wavelength dependence of the nonlinear absorption (top) and cubic NLO coefficients (bottom) of $\mathbf{3G_{22,03,02,01}}$.

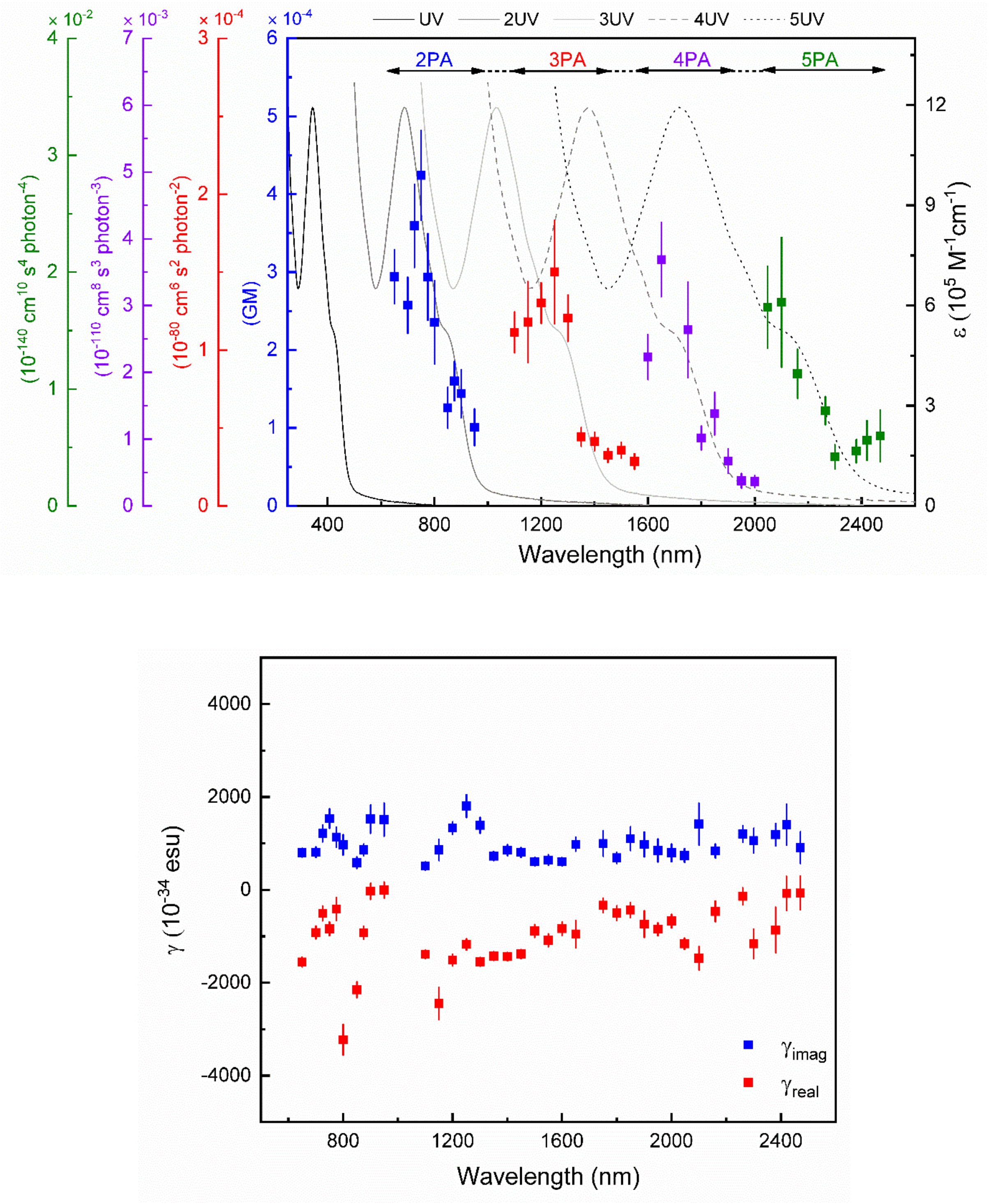

Figure S69. Wavelength dependence of the nonlinear absorption (top) and cubic NLO coefficients (bottom) of **2G22,03,01-s**.

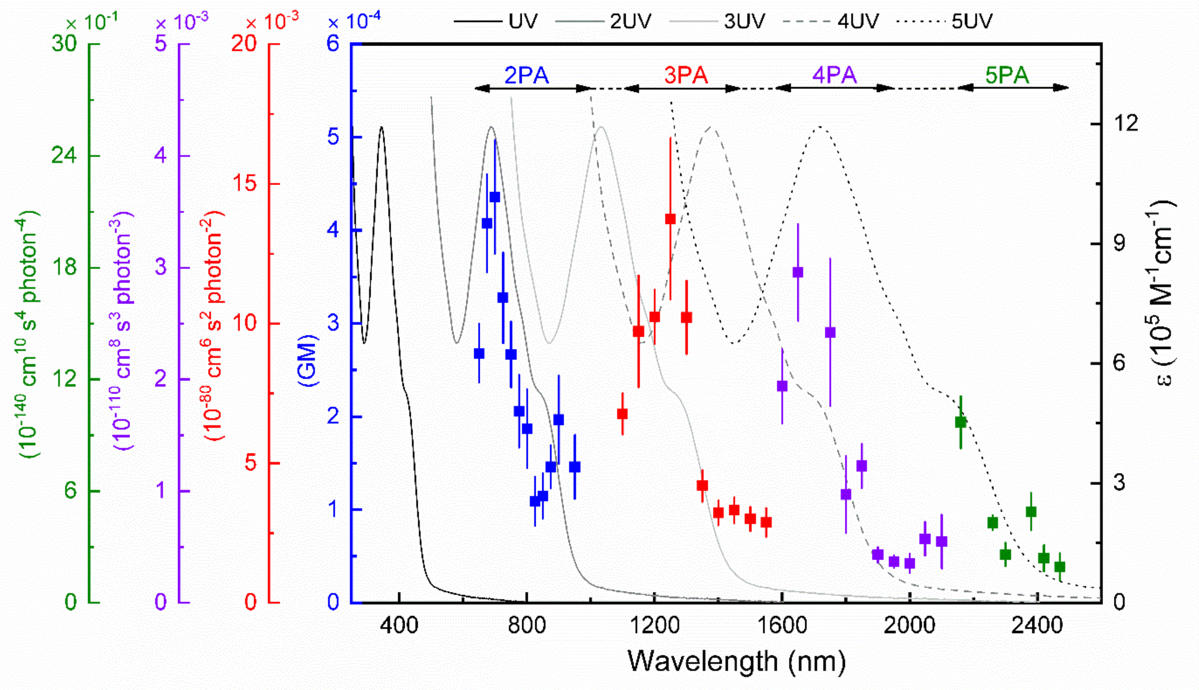

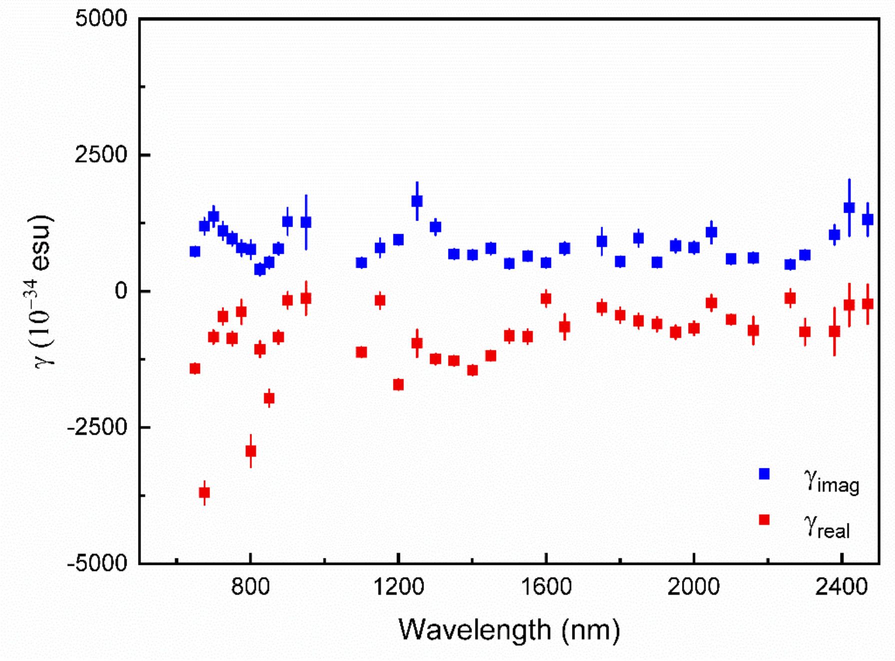

Figure S70. Wavelength dependence of the nonlinear absorption (top) and cubic NLO coefficients (bottom) of **2G$_{22,03,01}$**.

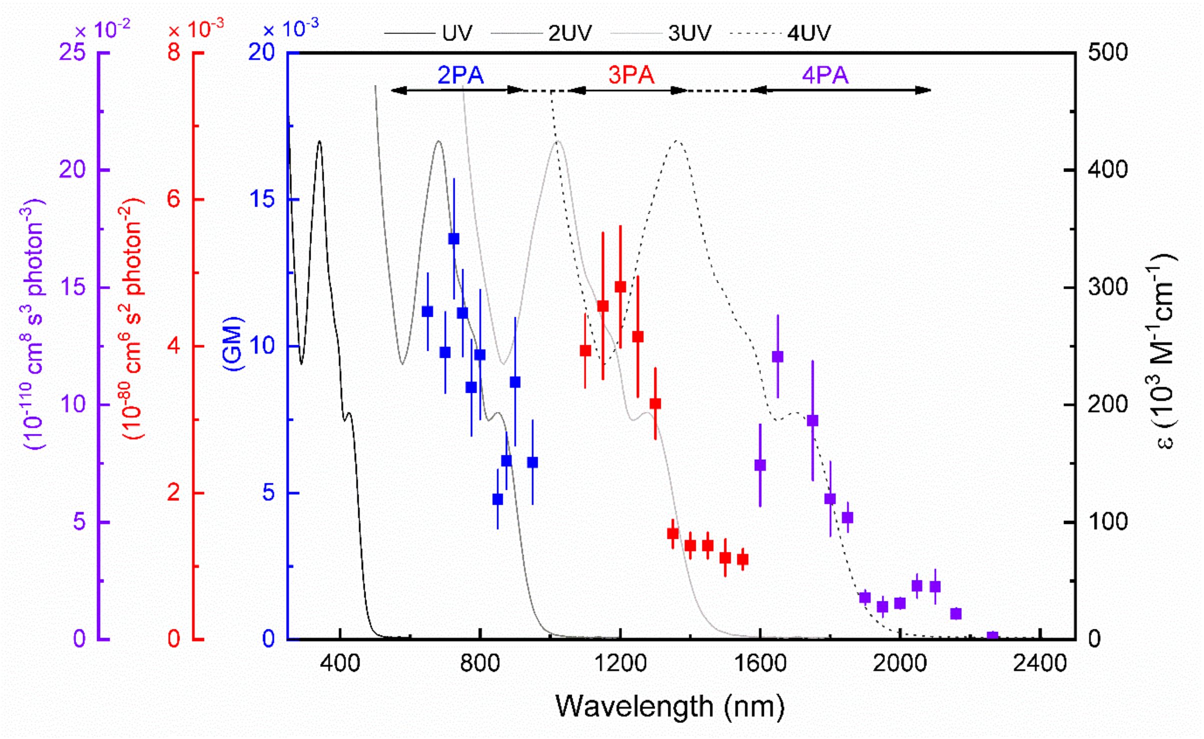

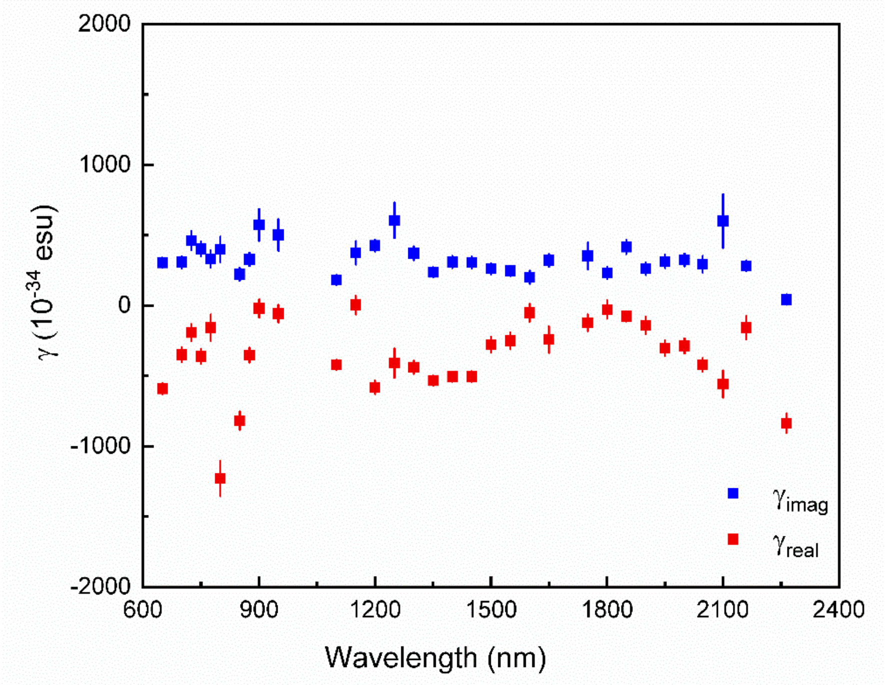

Figure S71. Wavelength dependence of the nonlinear absorption (top) and cubic NLO coefficients (bottom) of $\mathbf{1G_{22,01}}$.

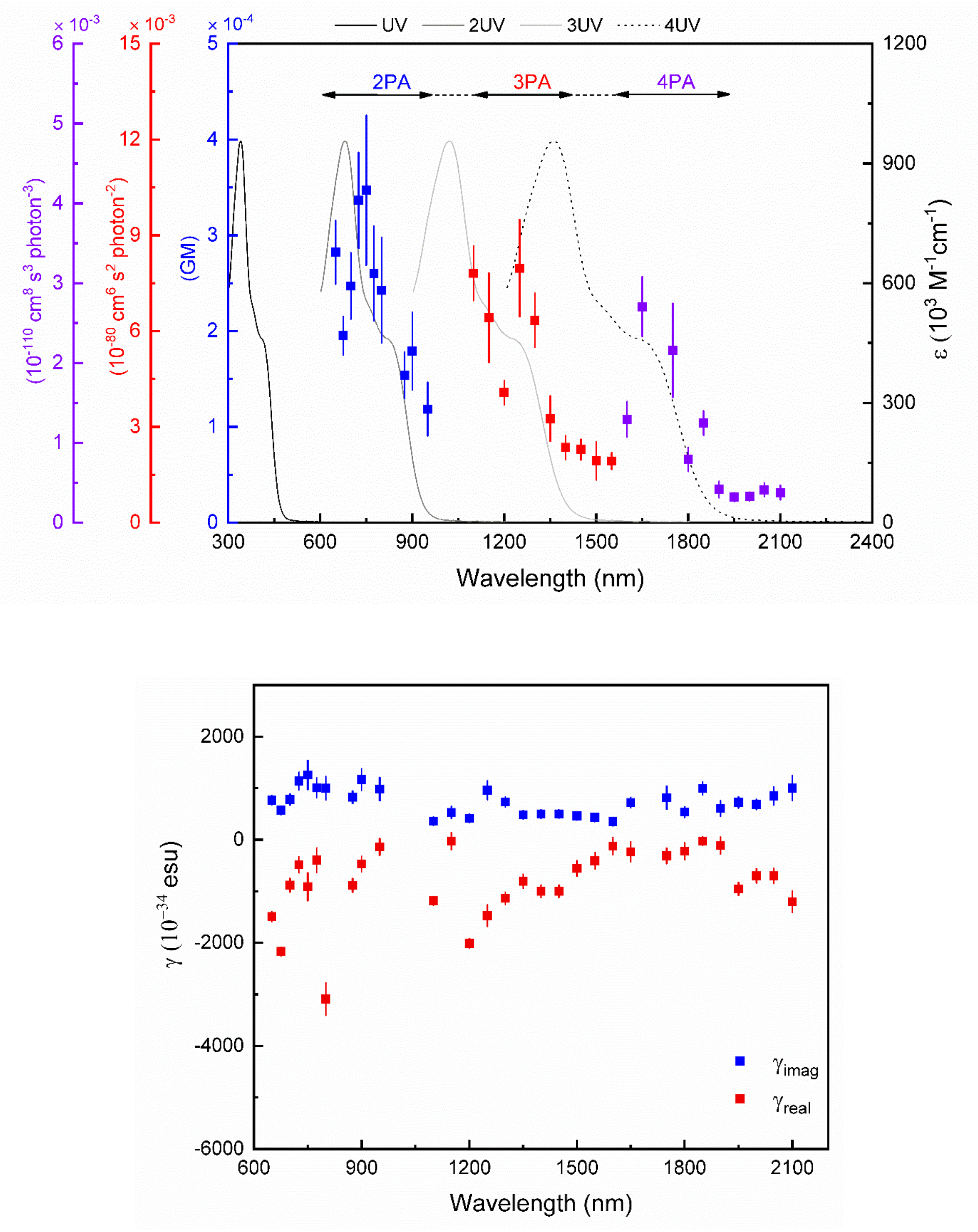

Figure S72. Wavelength dependence of the nonlinear absorption (top) and cubic NLO coefficients (bottom) of $\mathbf{2G_{12,02,01}}$.

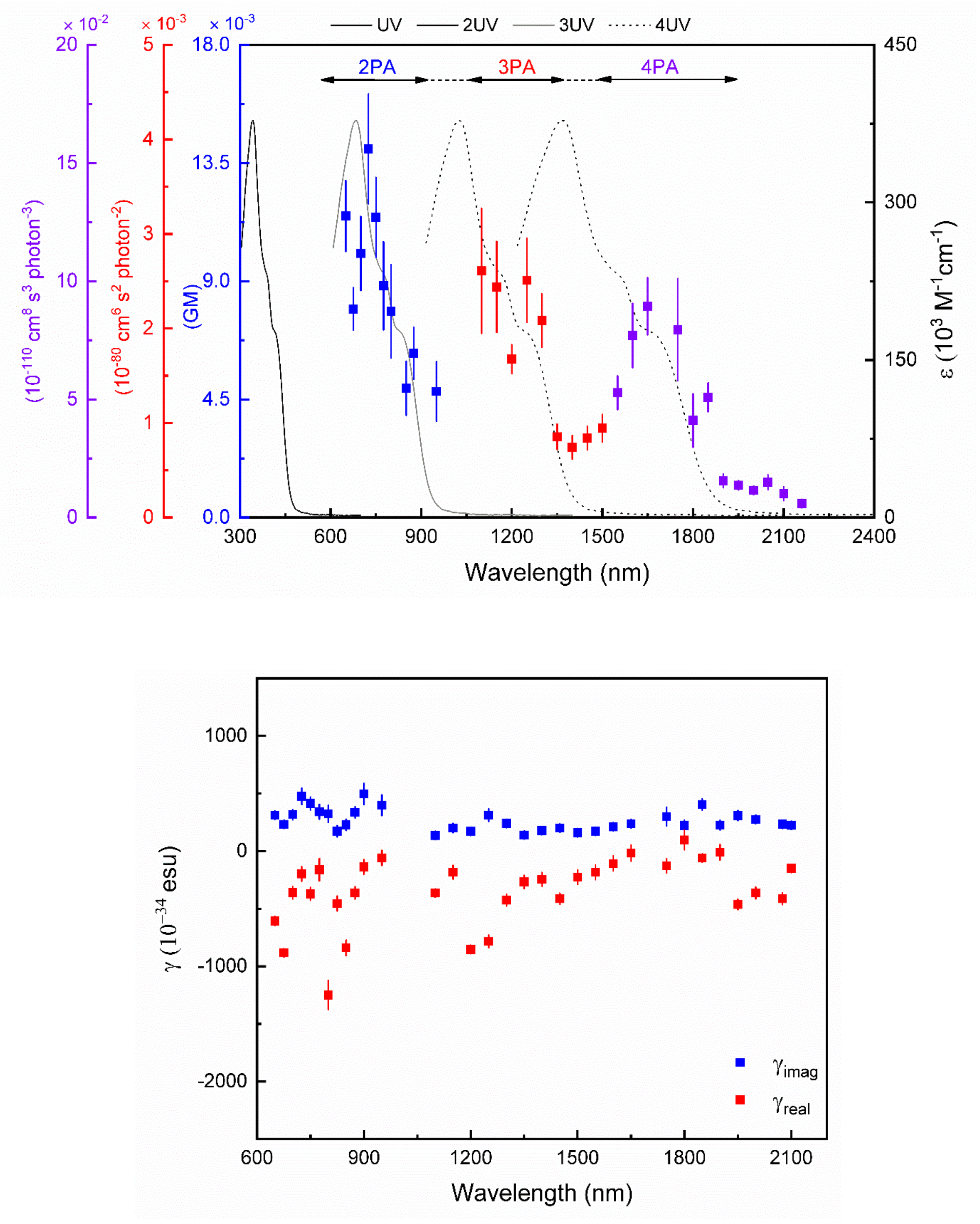

Figure S73. Wavelength dependence of the nonlinear absorption (top) and cubic NLO coefficients (bottom) of $\mathbf{1G_{12,01}}$.

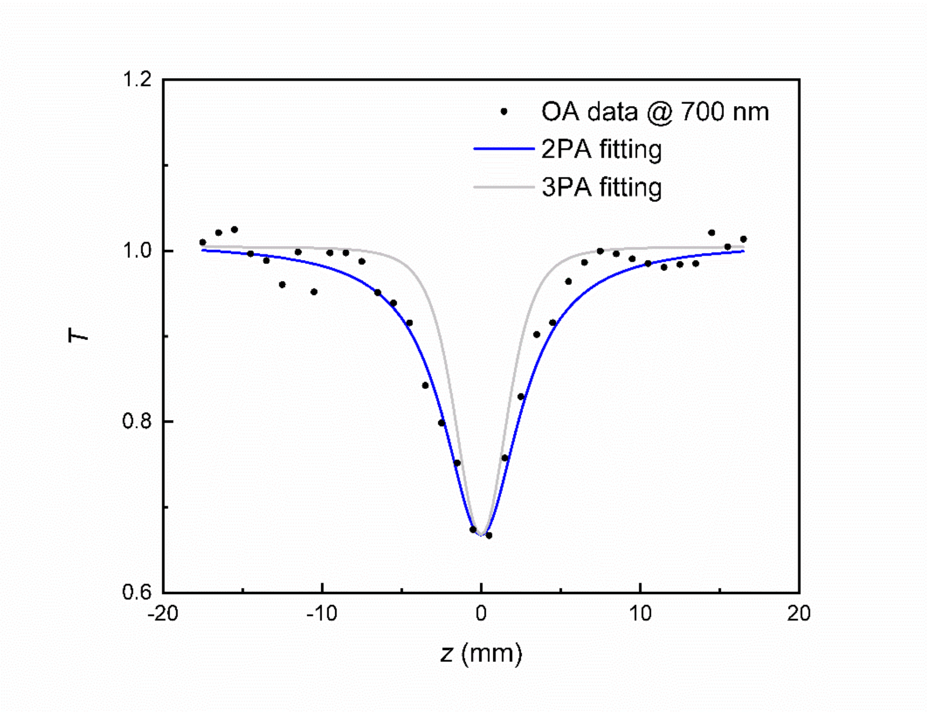

Figure S74. Open-aperture Z-scan data at 700 nm for a solution of **$3G_{22,03,02,01}$-s** in CH_2Cl_2 (black circles) and theoretical curves calculated for w_0 = 18 μm (determined from closed-aperture scans for pure solvent) assuming 2PA (blue line) or 3PA (grey line).

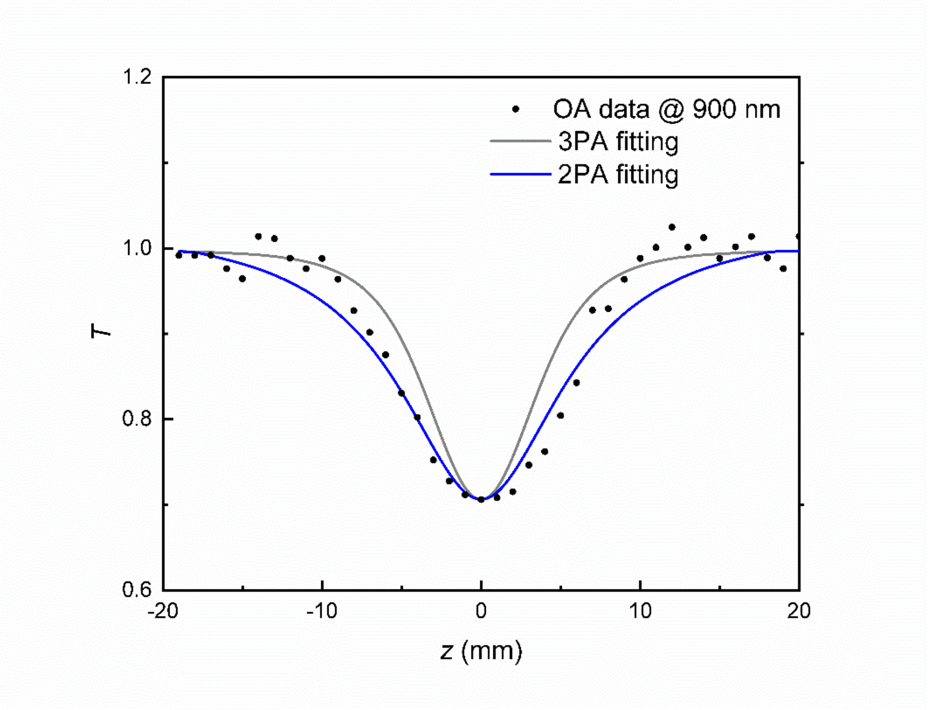

Figure S75. Open-aperture Z-scan data at 900 nm for a solution of **$3G_{22,03,02,01}$-s** in CH_2Cl_2 (black circles) and theoretical curves calculated for w_0 = 37 μm (determined from closed-aperture scans for pure solvent) assuming 2PA (blue line) or 3PA (grey line).

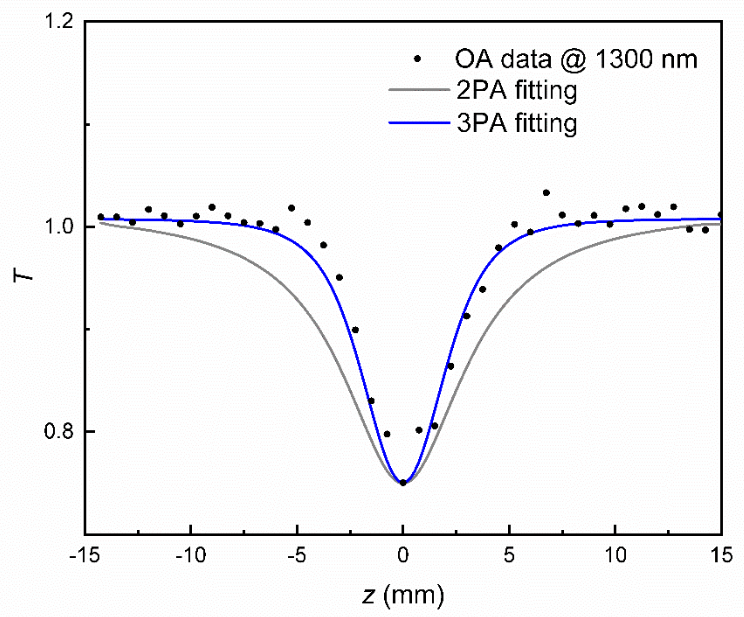

Figure S76. Open-aperture Z-scan data at 1300 nm for a solution of **3G22,03,02,01-s** in CH_2Cl_2 (black circles) and theoretical curves calculated for w_0 = 35 µm (determined from closed-aperture scans for pure solvent) assuming 2PA (grey line) or 3PA (blue line).

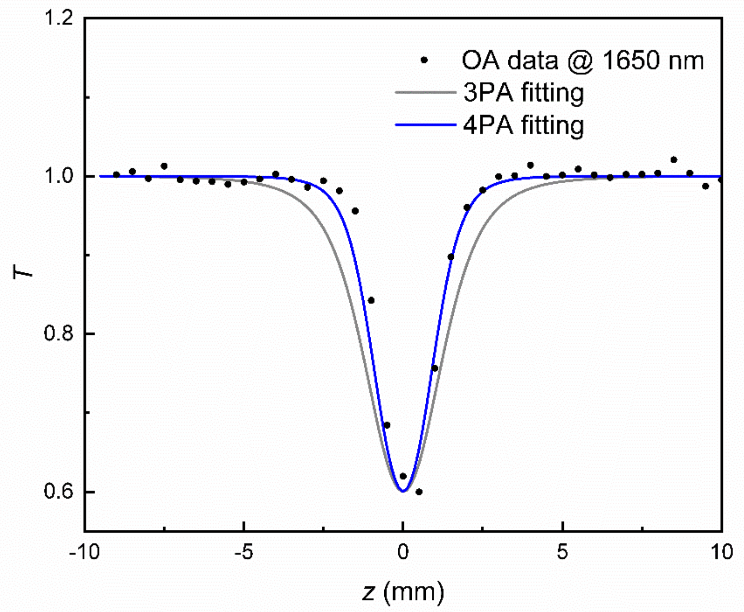

Figure S77. Open-aperture Z-scan data at 1650 nm for a solution of **3G22,03,02,01-s** in CH_2Cl_2 (black circles) and theoretical curves calculated for w_0 = 38 µm (determined from closed-aperture scans for pure solvent) assuming 3PA (grey line) or 4PA (blue line).

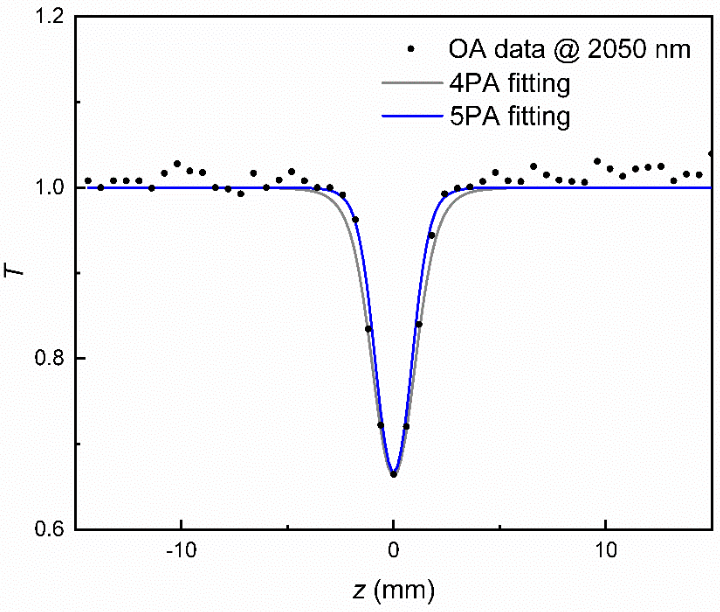

Figure S78. Open-aperture Z-scan data at 2050 nm for a solution of **3G$_{22,03,02,01}$-s** in CH_2Cl_2 (black circles) and theoretical curves calculated for w_0 = 32 μm (determined from closed-aperture scans for pure solvent) assuming 4PA (grey line) or 5PA (blue line).

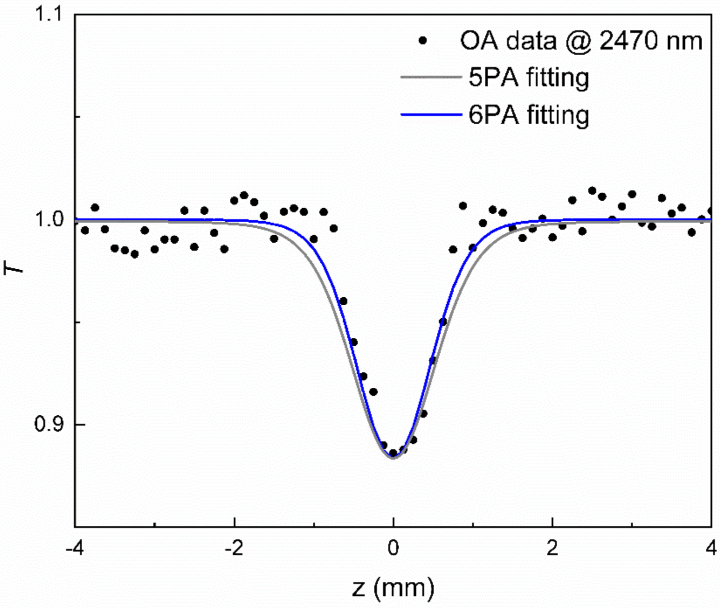

Figure S79. Open-aperture Z-scan data at 2470 nm for a solution of **3G$_{22,03,02,01}$-s** in CH_2Cl_2 (black circles) and theoretical curves calculated for w_0 = 32 μm (determined from closed-aperture scans for pure solvent) assuming 5PA (grey line) or 6PA (blue line).

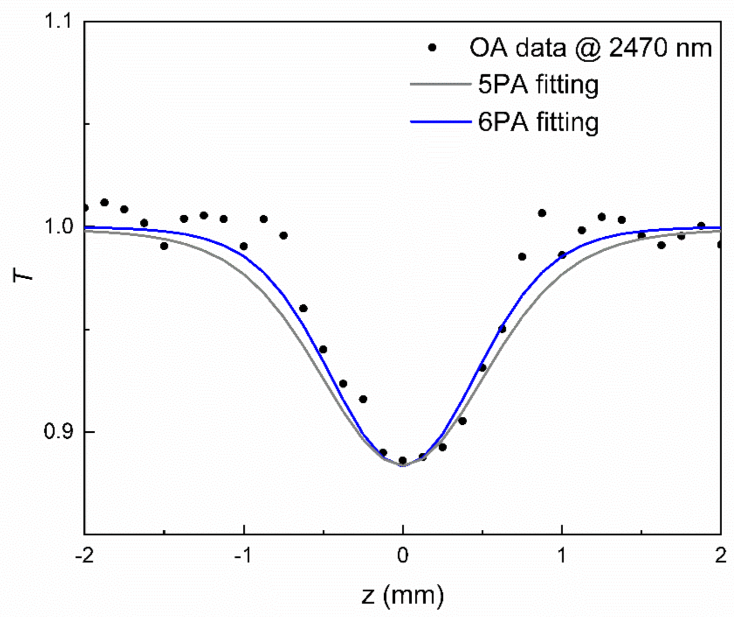

Figure S80. Partial scan (+/- 2 mm from the focal point) open-aperture Z-scan data at 2470 nm for a solution of **3G22,03,02,01-s** in CH_2Cl_2 (black circles) and theoretical curves calculated for w_0 = 32 μm (determined from closed-aperture scans for pure solvent) assuming 5PA (grey line) or 6PA (blue line).

Table S3. Three-photon, four-photon, five-photon, and six-photon absorption data for coordination complexes, organic molecules, polymers, MOFs, perovskites, and carbon-based materials (for data pre-2016, see ref [13]).

**Three-photon absorption materials**	**3PA quantities (λ_{ex} /nm)**	**Conditions**	**Ref.**
**Coordination complexes**			
({5,15-Bis(3,5-dibutoxycarbonylphenyl)}tetrabenzoporphyrin)platinum(II)	0.79 × 10^{-80} cm^6 s^2 $photon^{-2}$ (1800）	100 fs, 3PEP	[18]
(Tetrabenzoporphyrin)platinum(II)	0.97 × 10^{-80} cm^6 s^2 $photon^{-2}$ (1800）	100 fs, 3PEP	[18]
({2,6-Di[(3'-(ethoxycarbonyl)propyloxy)phenyl]}phthalimidoporphyrin)platinum(II)	1.69 × 10^{-80} cm^6 s^2 $photon^{-2}$ (1700）	100 fs, 3PEP	[18]
Bis[2-(2-pyridinyl-*N*)phenyl-*C*](1-(4-(*tert*-butyl)phenyl)-2-phenyl-imidazo[4,5-*f*][1,10]phenanthroline)(chlorido)iridium(III)	188 × 10^{-80} cm^6 s^2 $photon^{-2}$ (750)	fs, 3PEF	[19]
Bis[2-(2-pyridinyl-*N*)phenyl-*C*](1-(4-phenoxyphenyl)-2-phenyl-1*H*-imidazo[4,5-*f*][1,10]phenanthroline)(chlorido)iridium(III)	263 × 10^{-80} cm^6 s^2 $photon^{-2}$ (980)	fs, 3PEF	[19]
(2(3),9(10),16(17),23(24)-Tetrakis((4-(1,4,5-triphenyl-1*H*-imidazol-2-yl)phenyl)ethynyl)phthalocyanine)zinc(II)	299 × 10^{-80} cm^6 s^2 $photon^{-2}$ (1200)	70 fs, Z-scan	[20]
1,3,5-C_6H_3{C≡C-1,4-C_6H_4C≡C-*trans*-[Ru(dppe)$_2$]C≡C-1,4-C_6H_4C≡C-1,4-C_6H_4C≡C-1-C_6H_3-3,5-[C≡C-1,4-C_6H_4C≡C-1-C_6H_2-3,5-(*t*-Bu)$_2$-4-OMe]$_2$}$_3$	3500 × 10^{-80} cm^6 s^2 $photon^{-2}$ (1100)	130 fs, Z-scan	[13]
1,3,5-C_6H_3{C≡C-1,4-C_6H_4C≡C-*trans*-[Ru(dppe)$_2$]C≡C-1,4-C_6H_4C≡C-1,4-C_6H_4C≡C-1-C_6H_3-3,5-[C≡C-*trans*-[Ru(dppe)$_2$]C≡C-1,4-C_6H_4C≡C-1-C_6H_2-3,5-(*t*-Bu)$_2$-4-OMe]$_2$}$_3$	10,000 × 10^{-80} cm^6 s^2 $photon^{-2}$ (1200)	130 fs, Z-scan	[13]
1,3,5-{*trans*-[(dppe)$_2$(Cl)Ru(C≡C-1,4-C_6H_4C≡C)]}$_3$$C_6H_3$	(190 ± 50) × 10^{-80} cm^6 s^2 $photon^{-2}$ (1290)	130 fs, Z-scan	[21]

1,3,5-{*trans*-[(dppe)$_2$(Cl)Ru(C≡C-1,4-C_6H_4C≡C-1,4-C_6H_4C≡C)]}$_3$$C_6H_3$	(1800 ± 300) × 10^{-80} cm^6 s^2 $photon^{-2}$ (1200)	130 fs, Z-scan	[21]
1,3,5-{*trans*-[(dppe)$_2$(Cl)Ru(C≡C-1,4-C_6H_4C≡C-1-C_6H_2-2,5-{Et-hex}$_2$-4-C≡C-1,4-C_6H_4C≡C)]}$_3$$C_6H_3$	(1800 ± 300) × 10^{-80} cm^6 s^2 $photon^{-2}$ (1200)	130 fs, Z-scan	[21]
1,3,5-{*trans*-[(dppe)$_2$(Cl)Ru(C≡C-1,4-C_6H_4C≡C-1-C_6H_2-2,5-{Et-hex}$_2$-4-C≡C-1,4-C_6H_4C≡C-1,4-C_6H_4C≡C)]}$_3$$C_6H_3$	(3200 ± 500) × 10^{-80} cm^6 s^2 $photon^{-2}$ (1200)	130 fs, Z-scan	[21]
1,3,5-{*trans*-[(dppe)$_2$(Cl)Ru(C≡C-1,4-C_6H_4C≡C-1-C_6H_2-2,5-{Et-hex}$_2$-4-C≡C-1,4-C_6H_4C≡C-1,4-C_6H_4C≡C-1,4-C_6H_4C≡C-1,4-C_6H_4C≡C-1,4-C_6H_4C≡C)]}$_3$$C_6H_3$	(3300 ± 400) × 10^{-80} cm^6 s^2 $photon^{-2}$ (1200)	130 fs, Z-scan	[21]
1,3,5-{*trans*-[(dppe)$_2$(PhC≡C)Ru(C≡C-1,4-C_6H_4C≡C)]}$_3$$C_6H_3$	(100 ± 20) × 10^{-80} cm^6 s^2 $photon^{-2}$ (1240)	130 fs, Z-scan	[21]
1,3,5-{*trans*-[(dppe)$_2$(1,4-$O_2NC_6H_4$C≡C)Ru(C≡C-1,4-C_6H_4C≡C)]}$_3$$C_6H_3$	(740 ± 130) × 10^{-80} cm^6 s^2 $photon^{-2}$ (1290)	130 fs, Z-scan	[21]
1,3,5-{*trans*-[(dppe)$_2$(PhC≡C)Ru(C≡C-1,4-C_6H_4C≡C-1,4-C_6H_4C≡C)]}$_3$$C_6H_3$	(2300 ± 600) × 10^{-80} cm^6 s^2 $photon^{-2}$ (1200)	130 fs, Z-scan	[21]
1,3,5-{*trans*-[(dppe)$_2$(1,4-$O_2NC_6H_4$C≡C)Ru(C≡C-1,4-C_6H_4C≡C-1,4-C_6H_4C≡C)]}$_3$$C_6H_3$	(5000 ± 800) ×10^{-80} cm^6 s^2 $photon^{-2}$ (1200)	130 fs, Z-scan	[21]
{2-hydroxy-9(10),16(17),23(24)-tri(*tert*-butyl)phthalocyanine}zinc(II) dimer	8.8 cm^3 GW^{-2}	280 fs, Z-scan	[22]
{5,10,15,20-(Tetraphenyl)porphyrinato}zinc(II)	7.2 × 10^{-70} cm^6 s^2 $photon^{-2}$ (950), 1000 cm^3 GW^{-2} (950) – cumulative thermal effects suggested	150 fs, Z-scan	[23]
{2(3),9(10),16(17),23(24)-Tetrakis(4-diphenylaminophenyl)phthalocyanine}zinc(II)	87 × 10^{-70} cm^6 s^2 $photon^{-2}$ (950), 12,000 cm^3 GW^{-2} (950) – cumulative thermal effects suggested	150 fs, Z-scan	[23]
trans,trans-[(dppe)$_2$(Cl)Ru(μ-C≡C-1,4-C_6H_4-(*E*)-CH=CH-1-C_6H_2-2,5-{hexyloxy}$_2$-4-(*E*)-CH=CH-1,4-C_6H_4C≡C)Ru(Cl)(dppe)$_2$]	3500 × 10^{-80} cm^6 s^2 $photon^{-2}$ (1100)	130 fs, Z-scan	[24]

trans,trans-[(dppe)$_2$(Cl)Ru(μ-C≡C-1,4-C$_6$H$_4$-(*E*)-CH=CH-1-C$_6$H$_2$-2,5-{hexyloxy}$_2$-4-(*E*)-CH=CH-1-C$_6$H$_2$-2,5-{hexyloxy}$_2$-4-(*E*)-CH=CH-1-C$_6$H$_2$-2,5-{hexyloxy}$_2$-4-(*E*)-CH=CH-1,4-C$_6$H$_4$C≡C)Ru(Cl)(dppe)$_2$]	11,000 × 10^{-80} cm^6 s^2 photon^{-2} (1100)	130 fs, Z-scan	[24]
trans,trans-[(dppe)$_2$(Cl)Ru(μ-C≡C-1,4-C$_6$H$_4$-(*E*)-CH=CH-1,4-C$_6$H$_4$-(*E*)-CH=CH-1-C$_6$H$_2$-2,5-{hexyloxy}$_2$-4-(*E*)-CH=CH-1-C$_6$H$_2$-2,5-{hexyloxy}$_2$-4-(*E*)-CH=CH-1-C$_6$H$_2$-2,5-{hexyloxy}$_2$-4-(*E*)-CH=CH-1-C$_6$H$_2$-2,5-{hexyloxy}$_2$-4-(*E*)-CH=CH-1-C$_6$H$_2$-2,5-{hexyloxy}$_2$-4-(*E*)-CH=CH-1,4-C$_6$H$_4$-(*E*)-CH=CH-1,4-C$_6$H$_4$C≡C)Ru(Cl)(dppe)$_2$]	15,500 × 10^{-80} cm^6 s^2 photon^{-2} (1100)	130 fs, Z-scan	[24]
Bis(5-*N,N*-diethylthiophene-2-terpyridine)zinc(II)	0.117 × 10^{-80} cm^6 s^2 photon^{-2} (1700)	140 fs, 3PEF	[25]
[Ir{*N,N'*-2,2',6',2"-terpyridine-4'-[1,4-C$_6$H$_4$C(O)OSn(*n*-Butyl)$_3$]}(2-phenylpyridine-*N,C*)$_2$]PF$_6$	0.250 × 10^{-80} cm^6 s^2 photon^{-2} (1400)	200 fs, 3PEF	[26]
(5-(*N*(R)-Pyrrol-2-yl-(*E*)-vinyl)-5′-methyl-2,2′-bipyridyl)zinc(II) perchlorate, R = CH$_2$CH$_2$OCH$_2$CH$_2$OCH$_2$CH$_2$OMe	1.85 × 10^{-80} cm^6 s^2 photon^{-2} (1150)	110 fs, 3PEF	[27]
**Organic molecules**			
2,3-Bis(4-(phenyl(4-(1,2,2-triphenylvinyl)phenyl)amino)phenyl)fumaronitrile	382 × 10^{-80} cm^6 s^2 photon^{-2} (1560)	160 fs, nonlinear transmission	[28]
{5-(Diphenylamino)thiophene-2-vinyl}pyridinium hexafluorophosphate	115 × 10^{-90} cm^6 s^2 photon^{-2} (1200)	120 fs, 3PEF	[29]
5-(*N*(R)-Pyrrol-2-yl-(*E*)-vinyl)-5′-methyl-2,2′-bipyridyl, R = CH$_2$CH$_2$OCH$_2$CH$_2$OCH$_2$CH$_2$OMe	1.78 × 10^{-80} cm^6 s^2 photon^{-2} (1150)	110 fs, 3PEF	[27]
Indenoquinoxaline dendron D1, below	114 × 10^{-80} cm^6 s^2 photon^{-2} (1050)	130 fs, Z-scan	[30]

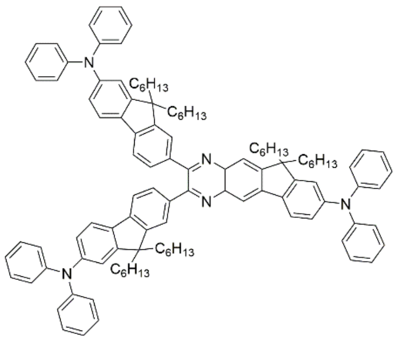

Indenoquinoxaline dendron D2, below	165×10^{-80} cm^6 s^2 photon^{-2} (1050)	130 fs, Z-scan	[30]

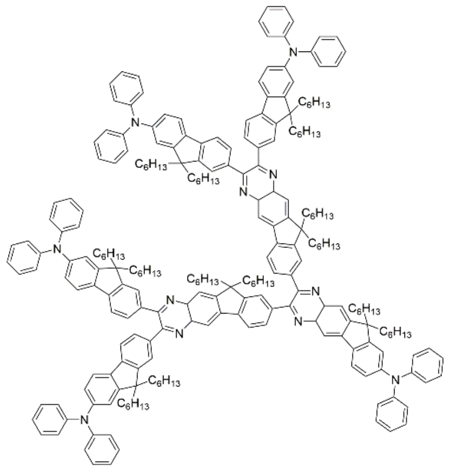

Indenoquinoxaline dendron D3, below	203×10^{-80} cm^6 s^2 photon^{-2} (1050)	130 fs, Z-scan	[30]

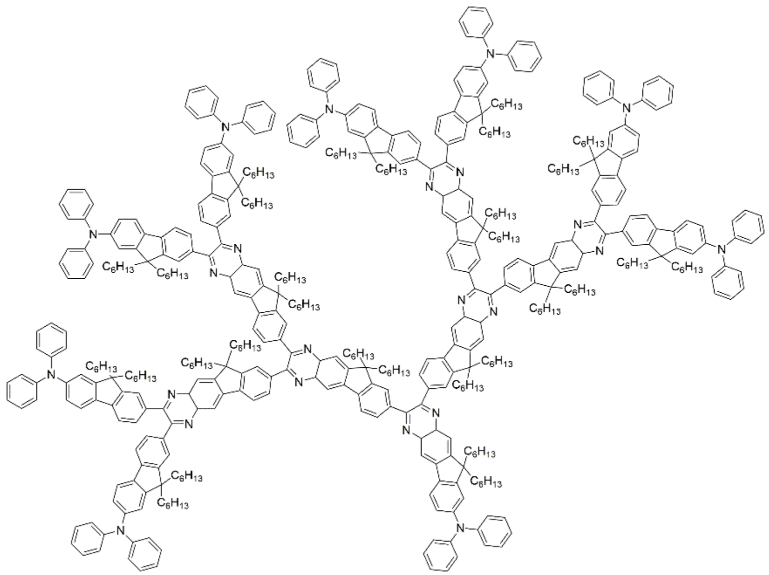

Indenoquinoxaline dendron D4, below	603 × 10^{-80} cm^6 s^2 photon^{-2} (1050)	130 fs, Z-scan	[30]

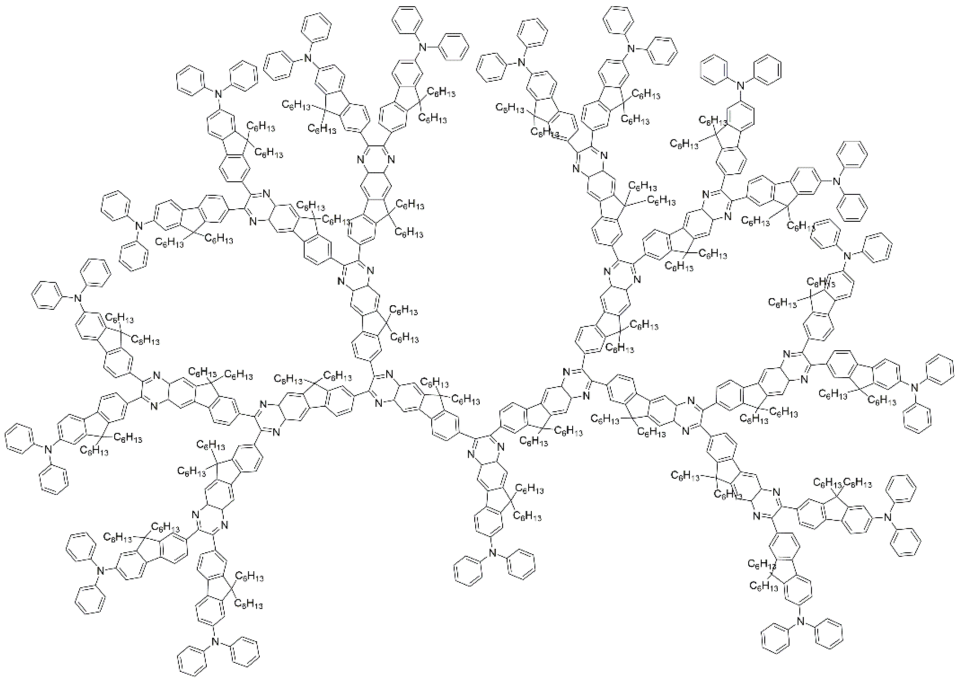

5,6-Bis{4′-(diphenylamino)-1,1′-biphenyl-4-yl}pyrazine-2,3-dicarbonitrile	29.5 × 10^{-80} cm^6 s^2 photon^{-2} (1550)	160 fs, nonlinear transmissivity	[31]
Cyclophosphazene-cored molecule 1, below	11,300 × 10^{-80} cm^6 s^2 photon^{-2} (800)	fs, DFWM	[32]

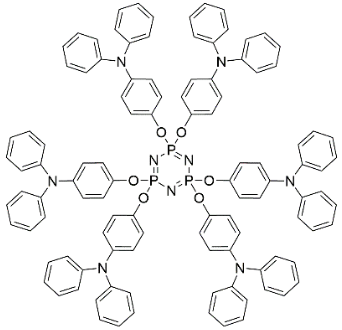

Cyclophosphazene-cored molecule 2, below	18,900 × 10^{-80} cm^6 s^2 $photon^{-2}$ (800)	fs, DFWM	[32]

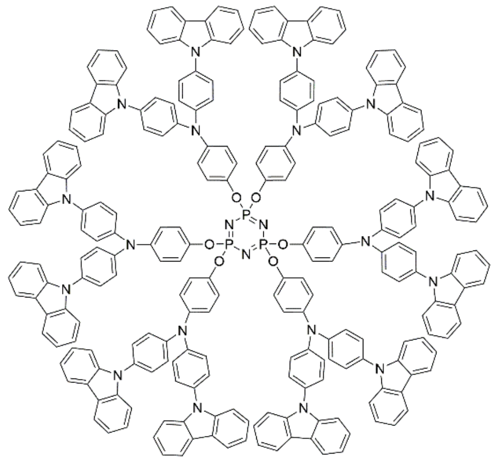

3-Phenyl-1-(thiophen-2-yl)prop-2*E*-en-1-one	8.5 × 10^{-4} cm^3 GW^{-2} (800)	100 fs, Z-scan	[33]
3-(4-Chlorophenyl)-1-(thiophen-2-yl)prop-2*E*-en-1-one	10.0 × 10^{-4} cm^3 GW^{-2} (800)	100 fs, Z-scan	[33]
3-{4-(1-Methylethyl)phenyl-1-(thiophen-2-yl)prop-2*E*-en-1-one	14.5 × 10^{-4} cm^3 GW^{-2} (800)	100 fs, Z-scan	[33]
5-Phenyl-1-(thiophen-2-yl)penta-2*E*,4*E*-dien-1-one	25.0 × 10^{-4} cm^3 GW^{-2} (800)	100 fs, Z-scan	[33]
1,3-Di(thiophen-2-yl)prop-2*E*-en-1-one	30.6 × 10^{-4} cm^3 GW^{-2} (800)	100 fs, Z-scan	[33]
3-(4-Hydroxyphenyl)-1-(thiophen-2-yl)prop-2*E*-en-1-one	40.0 × 10^{-4} cm^3 GW^{-2} (800)	100 fs, Z-scan	[33]
3-(4-Methoxyphenyl)-1-(thiophen-2-yl)prop-2*E*-en-1-one	49.9 × 10^{-4} cm^3 GW^{-2} (800)	100 fs, Z-scan	[33]
3-{4-(Methylthio)phenyl}-1-(thiophen-2-yl)prop-2*E*-en-1-one	68.0 × 10^{-4} cm^3 GW^{-2} (800)	100 fs, Z-scan	[33]

3-{4-(Dimethylamino)phenyl}-1-(thiophen-2-yl)prop-2*E*-en-1-one	105×10^{-4} cm^3 GW^{-2} (800)	100 fs, Z-scan	[33]
Boron-dipyrromethene dye, below	0.93×10^{-80} cm^6 s^2 $photon^{-2}$ (1600)	100 fs, 3PEF	[34]
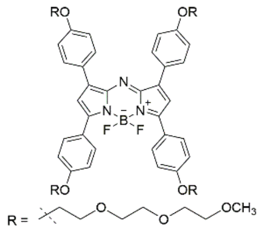			
(*E*)-1-Butyl-4-(2-(3a,7a-dihydro-1*H*-indol-3-yl)vinyl)quinolin-1-ium nitrate	0.36×10^{-80} cm^6 s^2 $photon^{-2}$ (1600)	fs, 3PEF	[35]
5′,5‴-(9,9-Dioctyl-9*H*-fluorene-2,7-diyl)-bis(3'-(1-(4-butylphenyl)-1*H*-phenanthro[9,10-*d*]imidazol-2-yl)-*N,N*-diphenyl-[1,1'-biphenyl]-4-amine)	$(209 \pm 12) \times 10^{-80}$ cm^6 s^2 $photon^{-2}$ (1010)	120 fs, 3PEF	[36]
5′,5‴-(9,9'-Spirobi[fluorene]-2,7-diyl)-bis(3'-(1-(4-butylphenyl)-1*H*-phenanthro[9,10-*d*]imidazol-2-yl)-*N,N*-diphenyl-[1,1'-biphenyl]-4-amine)	$(356 \pm 15) \times 10^{-80}$ cm^6 s^2 $photon^{-2}$ (1010)	120 fs, 3PEF	[36]
Star-shaped triazine-cored ladder-type ter(*p*-phenylene) [TA(TL)-Ph(3)-NPh], below	$16,000 \times 10^{-80}$ cm^6 s^2 $photon^{-2}$ (1260)	130 fs, 3PEF	[37]
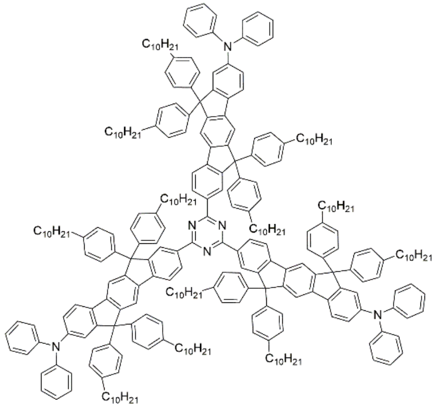			

Star-shaped triazine-cored ladder-type ter(*p*-phenylene) [TA(TL)-Ph(3)-ODZ], below	20,000 × 10^{-80} cm^6 s^2 $photon^{-2}$ (1200)	130 fs, 3PEF	[37]

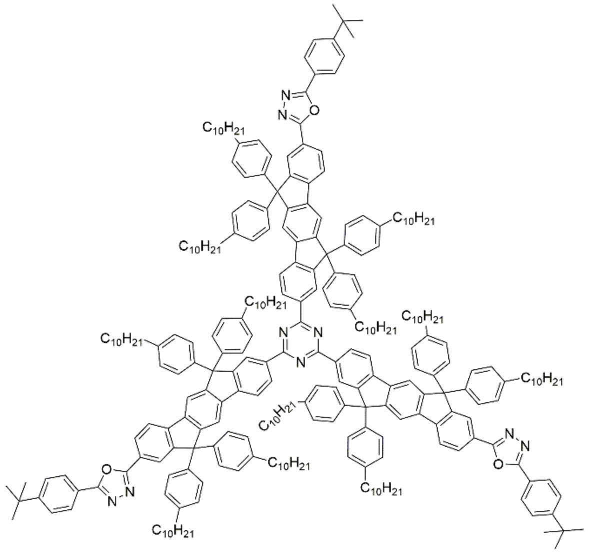

Star-shaped triazine-cored ladder-type ter(*p*-phenylene) TA(TL)-Ph(3)-CBZ], below	33,000 × 10^{-80} cm^6 s^2 $photon^{-2}$ (1200)	130 fs, 3PEF	[37]

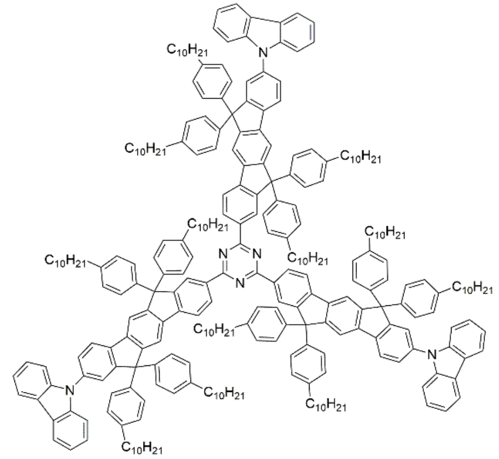

Spiro-fused ladder-type oligo(*p*-phenylene) (SpL(2)-1), below	156,000 × 10^{-80} cm^6 s^2 $photon^{-2}$ (940)	120 fs, Z-scan and 3PEF	[38]
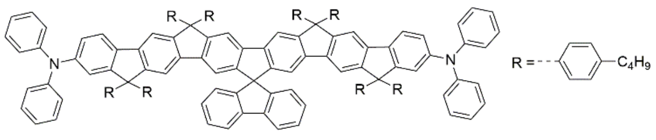			
Spiro-fused ladder-type oligo(*p*-phenylene) (SpL(2)-2), below	456,000 × 10^{-80} cm^6 s^2 $photon^{-2}$ (930)	120 fs, Z-scan and 3PEF	[38]
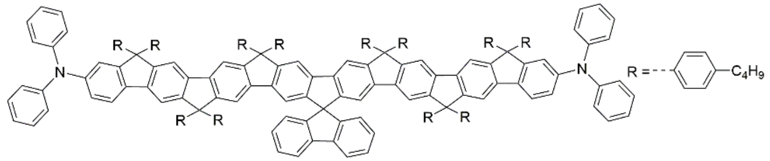			
Spiro-fused ladder-type oligo(*p*-phenylene) (SpL(2)-3), below	673,000 × 10^{-80} cm^6 s^2 $photon^{-2}$ (940)	120 fs, Z-scan and 3PEF	[38]
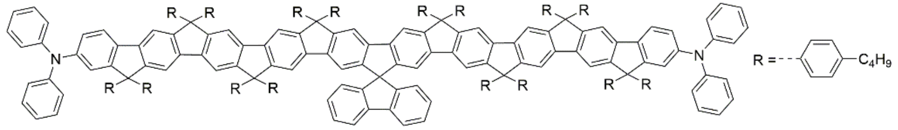			
Spiro-fused ladder-type oligo(*p*-phenylene) (SpL-1), below	308,000 × 10^{-80} cm^6 s^2 $photon^{-2}$ (950)	120 fs, Z-scan and 3PEF	[38]
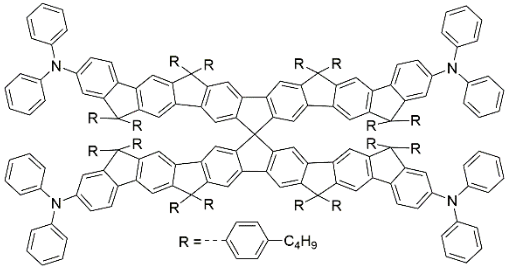			
Spiro-fused ladder-type oligo(*p*-phenylene) (SpL-2), below	972,000 × 10^{-80} cm^6 s^2 $photon^{-2}$ (940)	120 fs, Z-scan and 3PEF	[38]

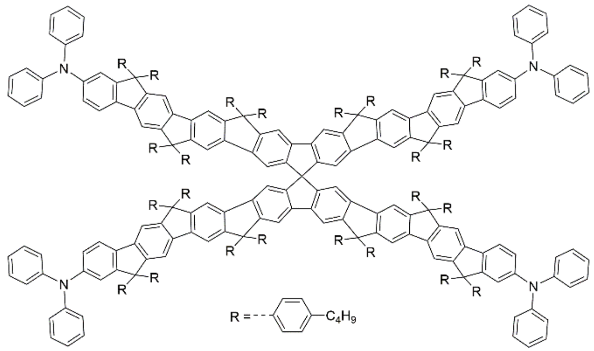			
Spiro-fused ladder-type oligo(*p*-phenylene) (SpL-3), below	2,390,000 × 10^{-80} cm^6 s^2 $photon^{-2}$ (940)	120 fs, Z-scan and 3PEF	[38]
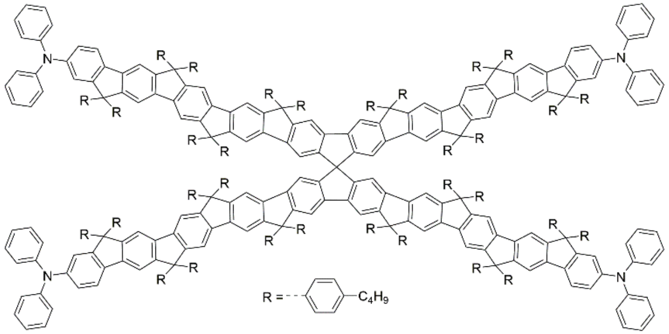			
**Polymers and polymeric composites**			
Graphene oxide-polyvinyl alcohol films (2 mg GO loading)	8790 cm^3 GW^{-2} (532)	5 ns, Z-scan	[39]
Graphene oxide-polyvinyl alcohol films (4 mg GO loading)	13,990 cm^3 GW^{-2} (532)	5 ns, Z-scan	[39]
Graphene oxide-polyvinyl alcohol films (6 mg GO loading)	37,990 cm^3 GW^{-2} (532)	5 ns, Z-scan	[39]
TPE-Br-to-TBDTT encapsulated with BSA nanoparticles (TPE-Br:TBDTT = 25:1)	0.192 × 10^{-80} cm^6 s^2 $photon^{-2}$ (1600)	fs, 3PEF	[40]
Montmorillonite/chitosan/poly(phenylenediamine) composites	0.0127 × 10^{-80} cm^6 s^2 $photon^{-2}$ (800)	110 fs, Z-scan	[41]

CdSe/ZnS quantum dots in PMMA (0.07 mg mL^{-1})	1.02 $\times 10^{-2}$ cm^3 GW^{-2} (1000-1340)	4 ns, Z-scan	[42]
CdSe/ZnS quantum dots in PMMA (0.1 mg mL^{-1})	0.6 $\times$ 10^{-2} cm^3 GW^{-2} (1000-1340)	4 ns, Z-scan	[42]
2,3-Bis(4′-(diphenylamino)-[1,1′-biphenyl]-4-yl)fumaronitrile encapsulated within Pluronic™ F-127 nanoparticle	57.7 $\times$ 10^{-80} cm^6 s^2 $photon^{-2}$ (1550)	160 fs, nonlinear transmissivity	[43]
**MOFs and coordination polymers**			
$Fe_4[Fe(CN)_6]_3 \cdot 15H_2O$ (Prussian blue)	450 $\times$ 10^{-80} cm^6 s^2 $photon^{-2}$ (1400)	~130 fs, Z-scan	[44]
$Zr_6O_4(OH)_4(TCPE)_3$	131 $\times$ 10^{-80} cm^6 s^2 $photon^{-2}$ (1100)	100 fs, Z-scan	[45]
$Hf_6O_4(OH)_4(TCPE)_3$	60 $\times$ 10^{-80} cm^6 s^2 $photon^{-2}$ (900-1200)	100 fs, Z-scan	[45]
$Hf_6O_4(OH)_6(H_2O)_2(CO_2CF_3)_2(TCPE)_2$	443 $\times$ 10^{-80} cm^6 s^2 $photon^{-2}$ (1020)	100 fs, Z-scan	[45]
$Hf_6O_4(OH)_6(H_2O)_2(OH)_2(TCPE)_2$	168 $\times$ 10^{-80} cm^6 s^2 $photon^{-2}$ (1050)	100 fs, Z-scan	[45]
$Zr_6O_4(OH)_6(H_2O)_2(CO_2CF_3)_2(TCPE)_2$	1495 $\times$ 10^{-80} cm^6 s^2 $photon^{-2}$ (980)	100 fs, Z-scan	[45]
$Zr_6O_4(OH)_6(H_2O)_2(OH)_2(TCPE)_2$	931 $\times 10^{-80}$ cm^6 s^2 $photon^{-2}$ (1020)	100 fs, Z-scan	[45]
Zn_2(benzoate)$_4$(*trans*,*trans*-9,10-bis(4-pyridylethenyl) anthracene)$_2$	310 $\times$ 10^{-80} cm^6 s^2 $photon^{-2}$ (1200)	fs, three-photon-induced PL	[46]
Ni_3(2,3,6,7,10,11-hexahydroxytriphenylene)$_2$	(1.80 ± 0.31) $\times$ 10^{-70} cm^6 s^2 $photon^{-2}$ (532)	15 ns, Z-scan	[47]
Co_3(2,3,6,7,10,11-hexahydroxytriphenylene)$_2$	(7.80 ± 0.31) $\times$ 10^{-70} cm^6 s^2 $photon^{-2}$ (532)	15 ns, Z-scan	[47]
**Perovskites**			
**Inorganic perovskites**			
$CsPbCl_3$ quantum dots	12,000 $\times$ 10^{-80} cm^6 s^2 $photon^{-2}$ (1064)	100 fs, nonlinear transmittance	[48]

$CsPbCl_3$ microcrystal	0.089 cm^3 GW^{-2} (1200)	50 fs, Z-scan	[49]
$CsPbBr_3$ nanocrystals	(380,000 ± 60,000 – 8,000,000 ± 1,000,000) × 10^{-80} cm^6 s^2 $photon^{-2}$ (1050-1500)	50 fs, Z-scan	[50]
$CsPbBr_3$ cubic nanocrystals	72 × 10^{-80} cm^6 s^2 $photon^{-2}$ nm^{-3} (1200)	100 fs, Z-scan	[51]
$CsPbBr_3$ 2D nanoplates	720 × 10^{-80} cm^6 s^2 $photon^{-2}$ nm^{-3} (1200)	100 fs, Z-scan	[51]
$CsPbBr_3$ nanorods	270,000 × 10^{-80} cm^6 s^2 $photon^{-2}$ (1300-1600)	100 fs, Z-scan	[52]
$CsPbBr_3$ quantum dots	310,000 × 10^{-80} cm^6 s^2 $photon^{-2}$ (1064)	100 fs, nonlinear transmittance	[48]
$CsPbI_3$ quantum dots	11,000,000 × 10^{-80} cm^6 s^2 $photon^{-2}$ (1064)	100 fs, nonlinear transmittance	[48]
$CsPbBr_{0.5}I_{2.5}$ quantum dots	6,200,000 × 10^{-80} cm^6 s^2 $photon^{-2}$ (1420)	100 fs, nonlinear transmittance	[48]
$CsPbBr_{1.5}I_{1.5}$ quantum dots	910,000 × 10^{-80} cm^6 s^2 $photon^{-2}$ (1064)	100 fs, nonlinear transmittance	[48]
$CsPbCl_{1.5}Br_{1.5}$ quantum dots	69,000 × 10^{-80} cm^6 s^2 $photon^{-2}$ (1064)	100 fs, nonlinear transmittance	[48]
$CsPbBr_{2.7}I_{0.3}$ nanoplates	(200,000 – 2,300,000) × 10^{-80} cm^6 s^2 $photon^{-2}$ (1300)	100 fs, nonlinear transmittance	[48]
$CsPb(Br_{0.85}I_{0.15})_3$ nanorods	360,000 × 10^{-80} cm^6 s^2 $photon^{-2}$ (1300-1600)	100 fs, Z-scan	[52]
$CsPb(Cl_{0.8}Br_{0.2})_3$ nanorods	220,000 × 10^{-80} cm^6 s^2 $photon^{-2}$ (1300-1600)	100 fs, Z-scan	[52]
Zero-dimensional Cs_4PbBr_6 non-fluorescent nanocrystal film	148,000,000 × 10^{-80} cm^6 s^2 $photon^{-2}$ (900)	70 fs, Z-scan and DFWM	[53]
Zero-dimensional Cs_4PbBr_6 fluorescent nanocrystal film	107,000,000 × 10^{-80} cm^6 s^2 $photon^{-2}$ (900)	70 fs, Z-scan and DFWM	[53]

$CsPbCl_3$: Mn nanocrystals	14,900 × 10^{-80} cm^6 s^2 $photon^{-2}$ (1300)	fs, Z-scan	[54]
$CsPbCl_3$: Mn nanoplates	65,400 × 10^{-80} cm^6 s^2 $photon^{-2}$ (1300)	fs, Z-scan	[54]
$(Cs_{0.06}FA_{0.79}MA_{0.15})Pb(I_{0.85}Br_{0.15})_3$ film	691 - 900 cm^3 GW^{-2} (790)	50 fs, Z-scan	[55]
$CsPb_{0.8}Zn_{0.2}I_3$ nanocrystals	120,000 × 10^{-80} cm^6 s^2 $photon^{-2}$ (1820)	fs, Z-scan	[56]
**Organic-inorganic hybrid perovskites**			
$CH_3NH_3PbBr_3$ nanocrystals	(330,000 ± 50,000 – 2,700,000 ± 400,000) × 10^{-80} cm^6 s^2 $photon^{-2}$ (1050-1500)	50 fs, Z-scan	[50]
$CH_3NH_3PbBr_3/(OA)_2PbBr_4$ core-shell nanocrystals	(2,500,000 ± 400,000 – 22,000,000 ± 3,000,000) × 10^{-80} cm^6 s^2 $photon^{-2}$ (1050-1500)	50 fs, Z-scan	[50]
$CH_3NH_3PbCl_3$ single crystal	(0.05 ± 0.01) cm^3 GW^{-2} (1064)	30 ps, Z-scan	[57]
$(C_4H_9NH_3)_2PbI_4$ flakes	(20,000 – 300,000) × 10^{-80} cm^6 s^2 $photon^{-2}$ (1100-1500)	200 fs, 3PEF	[58]
$(C_6H_{11}NH_3)_2PbI_4$ flakes	(560,000 – 2,370,000) × 10^{-80} cm^6 s^2 $photon^{-2}$ (1100-1500)	200 fs, 3PEF	[58]
$(C_6H_5(CH_2)_2NH_3)_2PbI_4$ flakes	(780,000 – 3,840,000) × 10^{-80} cm^6 s^2 $photon^{-2}$ (1100-1500)	200 fs, 3PEF	[58]
**Other inorganic nanoparticles**			
$CuNb_2O_6$	21.4 cm^3 GW^{-2} (800)	150 fs, Z-scan	[59]
ZnO nanocrystals embedded in Al_2O_3 matrix	1.1 × 10^{-1} cm^3 GW^{-2} (800)	fs, Z-scan and pump-probe techniques	[60]
Tellurite glass	7.1 cm^3 GW^{-2} (532)	5 ns, Z-scan	[61]
Er^{3+} ion-doped tellurite glass (0.185 × 10^{20} ions/cc)	19.4 cm^3 GW^{-2} (532)	5 ns, Z-scan	[61]

Er^{3+} ion-doped tellurite glass (0.371 × 10^{20} ions/cc)	21 cm^3 GW^{-2} (532)	5 ns, Z-scan	[61]
Er^{3+} ion-doped tellurite glass (1.111 × 10^{20} ions/cc)	46 cm^3 GW^{-2} (532)	5 ns, Z-scan	[61]
Er^{3+} ion-doped tellurite glass (1.848 × 10^{20} ions/cc)	76 cm^3 GW^{-2} (532)	5 ns, Z-scan	[61]
Er^{3+} ion-doped tellurite glass (3.680 × 10^{20} ions/cc)	92 cm^3 GW^{-2} (532)	5 ns, Z-scan	[61]
Barium bismuth borate glasses (10BaO - $35Bi_2O_3$ - $55B_2O_3$)	1.8 × 10^{-19} cm^3 GW^{-2} (532)	30 ps, Z-scan	[62]
	8.2 × 10^{-23} cm^3 GW^{-2} (800)	100 fs, Z-scan	[62]
Barium bismuth borate glasses (10BaO - $20Bi_2O_3$ - $50B_2O_3$)	8.25× 10^{-17} cm^3 GW^{-2} (532)	30 ps, Z-scan	[63]
	4.04 × 10^{-24} cm^3 GW-2 (800)	100 fs, Z-scan	[63]
Barium bismuth borate glasses (10BaO - $25Bi_2O_3$ - $45B_2O_3$)	12.38 × 10^{-17} cm^3 GW^{-2} (532)	30 ps, Z-scan	[63]
	4.585 × 10^{-24} cm^3 GW^{-2} (800)	100 fs, Z-scan	[63]
Barium bismuth borate glasses (10BaO - $30Bi_2O_3$ - $40B_2O_3$)	38.16 × 10^{-17} cm^3 GW^{-2} (532)	30 ps, Z-scan	[63]
	5.20 × 10^{-24} cm^3 GW^{-2} (800)	100 fs, Z-scan	[63]
In-doped ZnO nanowires	3.48 × 10^{-2} cm^3 GW^{-2} (1064)	50 ps, Z-scan	[64]
Sb_2Se_3 nanoparticles	1.8 × 10^{-7} cm^3 GW^{-2} (800)	100 fs, Z-scan	[65]
Orientation-patterned GaAs crystal	0.35 ± 0.08 cm^3 GW^{-2} (2300)	fs, Z-scan	[66]
α-FeOOH nanorod (average aspect ratio 33.1, length ca. 400 nm)	(290 – 310) × 10^{-80} cm^6 s^2 $photon^{-2}$ (1250)	80 fs, Z-scan and nonlinear transmission	[67]
α-FeOOH nanorod (average aspect ratio 28.2, length ca. 400 nm)	(530 - 560) × 10^{-80} cm^6 s^2 $photon^{-2}$ (1250)	80 fs, Z-scan and nonlinear transmission	[67]
α-FeOOH nanorod (average aspect ratio 24.4, length ca. 400 nm)	(710 – 720) × 10^{-80} cm^6 s^2 $photon^{-2}$ (1250)	80 fs, Z-scan and nonlinear transmission	[67]
α-FeOOH nanorod (average aspect ratio 19.9, length ca. 400 nm)	(830 - 850) × 10^{-80} cm^6 s^2 $photon^{-2}$ (1250)	80 fs, Z-scan and nonlinear transmission	[67]

α-FeOOH nanorod (average aspect ratio 15.9, length ca. 400 nm)	(1030 - 1080) × 10^{-80} cm^6 s^2 $photon^{-2}$ (1250)	80 fs, Z-scan and nonlinear transmission	[67]
CdSe nanocrystal (2.5 nm diameter)	410 × 10^{-80} cm^6 s^2 $photon^{-2}$ (1250)	80 fs, Z-scan and nonlinear transmission	[68]
CdSe nanocrystal (4.4 nm diameter)	2420 × 10^{-80} cm^6 s^2 $photon^{-2}$ (1250)	80 fs, Z-scan and nonlinear transmission	[68]
CdSe/CdS core/shell nanocrystal	379,000 × 10^{-80} cm^6 s^2 $photon^{-2}$ (1250)	80 fs, Z-scan and nonlinear transmission	[68]
$(Ag/SiO_2)_4$ metal–dielectric multilayer	117.55 cm^3 GW^{-2} (705)	120 fs, Z-scan	[69]
ZnS/Ag_2S composite nanoparticle (ZnS : Ag_2S = 1:0.11)	19 cm^3 GW^{-2} (532)	5 ns, Z-scan	[70]
**Carbon-based materials**			
Graphene-ZnO nanocomposites	8.54 × 10^{-3} cm^3 GW^{-2} (1030)	340 fs, Z-scan	[71]
F,N doped carbon dots (N-CDs-F)	9.55 × 10^{-80} cm^6 s^2 $photon^{-2}$ (1600)	fs, 3PEF	[72]
"Organosilane-chained" carbon dots	5.2 × 10^{-3} cm^3 GW^{-2} (1400)	50 fs, Z-scan	[73]
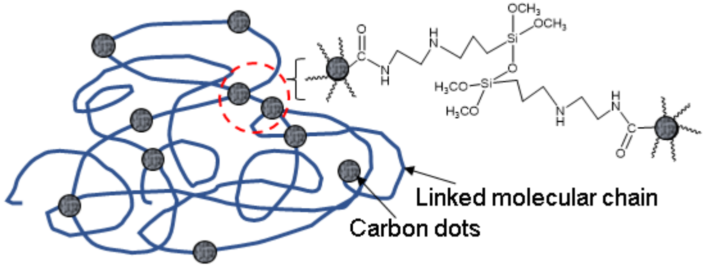			
Graphene oxide-encapsulated 2,3-bis(4-(phenyl(4-(1,2,2-triphenylvinyl)phenyl)amino)phenyl)fumaronitrile nanoparticles	145 × 10^{-70} cm^6 s^2 (1560)	160 fs, nonlinear transmission	[28]

**Four-photon absorption materials**	**4PA quantities (λ_{ex} /nm)**	**Conditions**	**Ref.**
**Coordination complexes**			
Pt(II) [4-(4-fluorophenyl)-2,6-diphenylpyridine](DMSO)	7.0×10^{-82} cm^8 s^3 photon^{-3} (1600)	120 fs, 4PEF	[74]
Pt(II) [4-(3,4-difluorophenyl)-2,6-diphenylpyridine](DMSO)	15.2×10^{-82} cm^8 s^3 photon^{-3} (1600)	120 fs, 4PEF	[74]
Pt(II) [4-(3,5-difluorophenyl)-2,6-diphenylpyridine](DMSO)	6.4×10^{-82} cm^8 s^3 photon^{-3} (1600)	120 fs, 4PEF	[74]
1,3,5-(3,5-{4-MeO-3,5-(*t*-Bu)$_2$-C$_6$H$_2$-1-C≡C-1,4-C$_6$H$_4$C≡C-*trans*-[Ru(dppe)$_2$]C≡C}$_2$C$_6$H$_3$-1-C≡C-1,4-C$_6$H$_4$C≡C-1,4-C$_6$H$_4$C≡C-*trans*-[Ru(dppe)$_2$]C≡C-1,4-C$_6$H$_4$C≡C)$_3$C$_6$H$_3$	2100×10^{-110} cm^8 s^3 photon^{-3} (1600)	130 fs, Z-scan	[13]
1,3,5-{*trans*-[(dppe)$_2$(4-O$_2$NC$_6$H$_4$C≡C)Ru(C≡C-1,4-C$_6$H$_4$C≡C-1,4-C$_6$H$_4$C≡C)]}$_3$C$_6$H$_3$	$(180 \pm 50) \times 10^{-110}$ cm^8 s^3 photon^{-3} (1750)	130 fs, Z-scan	[21]
2(3), 9(10), 16(17), 23(24) Tetrakis-4-((4-(1,4,5-triphenyl-1*H*-imidazol-2-yl)phenyl)ethynyl)phthalocyanine zinc(II)	6270×10^{-110} cm^8 s^3 photon^{-3} (1300)	70 fs, Z-scan	[20]
**Organic molecules**			
Spiro-fused ladder-type oligo(*p*-phenylene) (SpL-3), below	5100×10^{-110} cm^8 s^3 photon^{-3} (1440)	120 fs, Z-scan and 4PEF	[38]

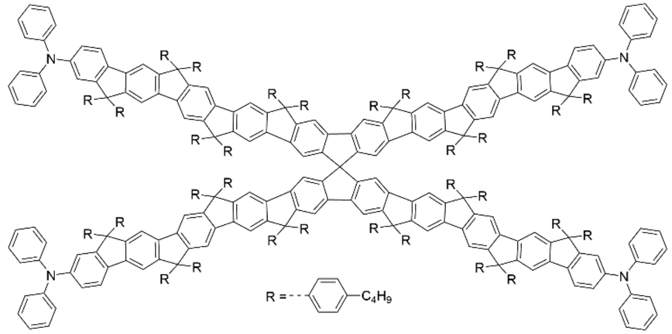

**Polymer and polymeric composites**			
CdSe/ZnS quantum dots in PMMA (0.07 mg mL^{-1})	1.18×10^{-4} cm^5 GW^{-3} (1000-1340)	4 ns, Z-scan	[42]
CdSe/ZnS quantum dots in PMMA (0.1 mg mL^{-1})	0.57×10^{-4} cm^5 GW^{-3} (1000-1340)	4 ns, Z-scan	[42]
**MOFs and coordination polymers**			
$Zr_6O_4(OH)_4(TCPE)_3$	109×10^{-110} cm^8 s^3 $photon^{-3}$ (1450)	100 fs, Z-scan	[45]
$Hf_6O_4(OH)_4(TCPE)_3$	70×10^{-110} cm^8 s^3 $photon^{-3}$ (1200-1600)	100 fs, Z-scan	[45]
$Zr_6O_4(OH)_6(H_2O)_2(OH)_2(TCPE)_2$	235×10^{-110} cm^8 s^3 $photon^{-3}$ (1470)	100 fs, Z-scan	[45]
$Hf_6O_4(OH)_6(H_2O)_2(CO_2CF_3)_2(TCPE)_2$	120×10^{-110} cm^8 s^3 $photon^{-3}$ (1530)	100 fs, Z-scan	[45]
$Hf_6O_4(OH)_6(H_2O)_2(OH)_2(TCPE)_2$	38×10^{-110} cm^8 s^3 $photon^{-3}$ (1500)	100 fs, Z-scan	[45]
$Zr_6O_4(OH)_6(H_2O)2(CO_2CF_3)_2(TCPE)_2$	400×10^{-110} cm^8 s^3 $photon^{-3}$ (1450)	100 fs, Z-scan	[45]
**Perovskites**			
$CsPbCl_3$ microcrystal	1.1×10^{-4} cm^5 GW^{-3} (1600)	50 fs, Z-scan	[49]
$CsPbBr_3$ nanocrystal	$(0.07 \pm 0.01 - 7.0 \pm 1.0) \times 10^{-104}$ cm^8 s^3 $photon^{-3}$ (1550-2000)	50 fs, Z-scan	[50]
$CH_3NH_3PbBr_3$ nanocrystal	$(0.036 \pm 0.005 - 3.0 \pm 0.5) \times 10^{-104}$ cm^8 s^3 $photon^{-3}$ (1550-2000)	50 fs, Z-scan	[50]
$CH_3NH_3PbBr_3/(OA)_2PbBr_4$ core-shell nanocrystals	$(210{,}000 \pm 30{,}000 – 24{,}000{,}000 \pm 4{,}000{,}000) \times 10^{-110}$ cm^8 s^3 $photon^{-3}$ (1550-2000)	50 fs, Z-scan	[50]
$CsPbBr_{2.7}I_{0.3}$ nanoplates	$(130{,}000 – 2{,}060{,}000) \times 10^{-110}$ cm^8 s^3 $photon^{-3}$ (1600)	100 fs, nonlinear transmittance	[75]
Zero-dimensional Cs_4PbBr_6 non-fluorescent nanofilm	3.97×10^{-100} cm^8 s^3 $photon^{-3}$ (1400)	70 fs, Z-scan and DFWM	[53]

Zero-dimensional Cs_4PbBr_6 fluorescent nanofilm	24.7×10^{-100} cm^8 s^3 $photon^{-3}$ (1400)	70 fs, Z-scan and DFWM	[53]
**Other inorganic nanoparticles**			
$(Ag/SiO_2)_4$ metal–dielectric multilayer	2.82 cm^5 GW^{-3} (715)	120 fs, Z-scan	[69]
$CdSiP_2$	(5.3 - 7.80) $\times 10^{-6}$ cm^5 GW^{-3} (1500)	35 fs, Z-scan	[76]
GaP crystals	(2.6-65) $\times$ 10^{-4} cm^5 GW^{-3}	135 fs, Z-scan	[77]
ZnTe crystals	(3.5-9.1) $\times$ 10^{-4} cm^5 GW^{-3}	135 fs, Z-scan	[77]
ZnSe:Mn quantum dots	20.3×10^{-80} cm^8 s^3 $photon^{-3}$ (1350)	50 fs, Z-scan	[78]
**Carbon-based materials**			
F,N doped carbon dots (N-CDs-F)	6.32×10^{-80} cm^8 s^3 $photon^{-3}$ (2250)	fs, 4PEF	[72]
"Organosilane-chained" carbon dots	1.16×10^{-6} cm^5 GW^{-3} (1900)	50 fs, Z-scan	[73]

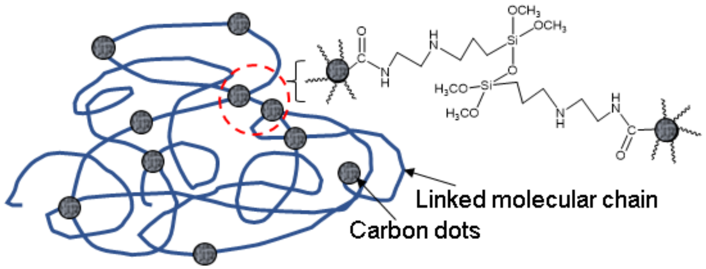

**Five-photon absorption materials**	**5PA quantities (λ_{ex} /nm)**	**Conditions**	**Ref.**
**Organic molecules**			
Spiro-fused ladder-type oligo(*p*-phenylene) (SpL-3), below	9320×10^{-140} cm^{10} s^4 $photon^{-4}$ (1540)	120 fs, Z-scan and 5PEF	[38]

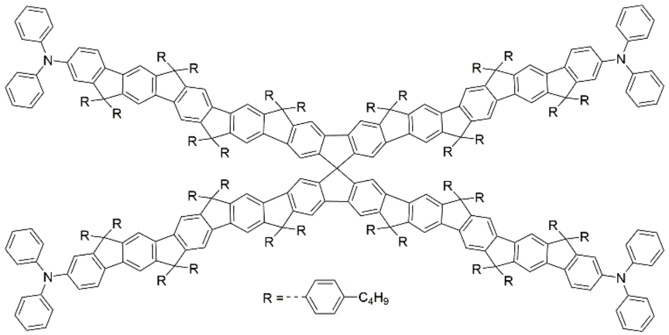

**Perovskites**			
$CsPbCl_3$ microcrystal	2.3×10^{-7} cm^7 GW^{-4} (1800)	50 fs, Z-scan	[49]
$CH_3NH_3PbBr_3/(OA)_2PbBr_4$ core-shell nanocrystals	$(2900 \pm 400 – 200{,}000 \pm 30{,}000) \times 10^{-140}$ cm^{10} s^4 photon^{-4} (2050-2300)	50 fs, Z-scan	[50]
$CH_3NH_3PbBr_3$ nanocrystals	$(390 \pm 60$ - $24{,}000 \pm 4000) \times 10^{-140}$ cm^{10} s^4 photon^{-4} (2050-2300)	50 fs, Z-scan	[50]
$CsPbBr_3$ nanocrystals	$(900 \pm 100$ - $65{,}000 \pm 10{,}000) \times 10^{-140}$ cm^{10} s^4 photon^{-4} (2050-2300)	50 fs, Z-scan	[50]
$CsPbBr_{2.7}I_{0.3}$ nanoplates	$15{,}000 \times 10^{-140}$ cm^{10} s^4 photon^{-4} (2200)	100 fs, nonlinear transmittance	[75]
**Carbon-based materials**			
Graphene-ZnO nanocomposites	7.87×10^{-5} cm^7 GW^{-4} (1030)	340 fs, Z-scan	[71]
**Six-photon absorption materials**	**6PA quantities (λ_{ex} /nm)**	**Conditions**	**Ref.**
**Organic molecules**			
Spiro-fused ladder-type oligo(*p*-phenylene) (SpL-3), below	86.7×10^{-170} cm^{12} s^5 photon^{-5} (1820)	120 fs, Z-scan and 6PEF	[38]

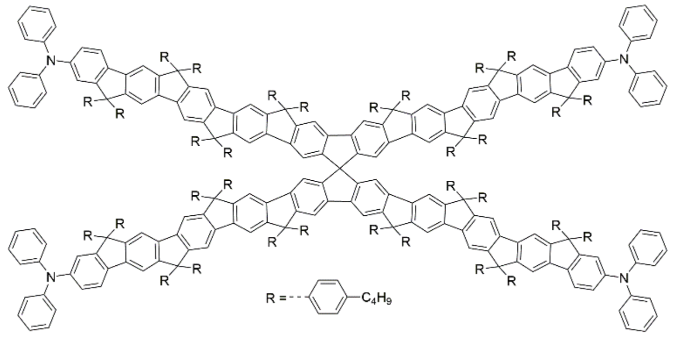

**Perovskites**			
$CsPbCl_3$ microcrystal	1.1×10^{-10} cm^9 GW^{-5} (2200)	50 fs, Z-scan	[49]
$CH_3NH_3PbCl_3$ microcrystal	9×10^{-10} cm^9 GW^{-5} (2400)	50 fs, Z-scan	[79]

3PEP = three-photon excited phosphorescence. 3PEF = three-photon excited fluorescence. Et-hex = 2-ethylhexyl. DFWM = degenerate four-wave mixing. GO = graphene oxide. TPE-Br = 4,4′,4″,4‴-(ethene-1,1,2,2-tetrayltetrakis(benzene-4,1-diyl))tetrakis (1-(4-bromobenzyl)pyridin-1-ium) bromide. TBDTT = 2-({4-[7-(4-{phenyl[4-(triphenylethenyl)phenyl]amino}phenyl)-2,1,3-benzothiadiazol-4-yl]phenyl}(thiophen-2-yl)methylidene)propanedinitrile. BSA = bovine serum albumin. PMMA = poly(methyl methacrylate). H_4TCPE = tetrakis[4-(4-carboxyphenyl)phenyl]ethylene. FA = formamidinium. MA = methylammonium. OA = octylammonium.

References

1. D. Touchard, P. Haquette, S. Guesmi, L. Le Pichon, A. Daridor, L. Toupet, P. H. Dixneuf, *Organometallics* **1997**, *16*, 3640-3648.
2. *Purification of Laboratory Chemicals (Sixth Edition)*, W. L. F. Armarego; C. L. L. Chai, Eds. Butterworth-Heinemann: Oxford, **2009**; Ch. 5. pp 445-576.
3. R. P. Hsung, C. E. D. Chidsey, L. R. Sita, *Organometallics* **1995**, *14*, 4808-4815.
4. K. A. Green, P. V. Simpson, T. C. Corkery, M. P. Cifuentes, M. Samoc, M. G. Humphrey, *Macromol. Rapid Commun.* **2012**, *33*, 573-578.
5. H. Zhao, P. V. Simpson, A. Barlow, G. J. Moxey, M. Morshedi, N. Roy, R. Philip, C. Zhang, M. P. Cifuentes, M. G. Humphrey, Chem. *Eur. J.* **2015**, *21*, 11843-11854.
6. G. T. Dalton, M. P. Cifuentes, L. A. Watson, S. Petrie, R. Stranger, M. Samoc, M. G. Humphrey, *Inorg. Chem.* **2009**, *48*, 6534-6547.
7. S. K. Hurst, M. P. Cifuentes, M. G. Humphrey, *Organometallics* **2002**, *21*, 2353-2355.
8. T. Schwich. Towards Octupolar Ruthenium Acetylide Complexes for Nonlinear Optical Materials: Synthesis, Characterization and Linear and Nonlinear Optical Properties. PhD Thesis, Australian National University, Canberra, **2012**.
9. R. Evans, Z. Deng, A. K. Rogerson, A. S. McLachlan, J. J. Richards, M. Nilsson, G. A. Morris, *Angew. Chem. Int. Ed.* **2013**, *52*, 3199-3202.
10. M. Holz, H. Weingartner, *J. Magn. Reson.* **1991**, *92*, 115-125.
11. M. Sheik-Bahae, A. A. Said, T. Wei, D. J. Hagan, E. W. V. Stryland, *IEEE J. Quantum Electron*. **1990**, *26*, 760-769.
12. D. S. Corrêa, L. De Boni, L. Misoguti, I. Cohanoschi, F. E. Hernandez, C. R. Mendonça, *Opt. Commun.* **2007**, *277*, 440-445.
13. P. V. Simpson, L. A. Watson, A. Barlow, G. Wang, M. P. Cifuentes, M. G. Humphrey, *Angew. Chem. Int. Ed.* **2016**, *55*, 2387-2391.
14. M. G. Kuzyk, *Phys. Rev. A* **2005**, *72*, 053819.
15. J. Pérez-Moreno, M. G. Kuzyk, *Adv. Mater.* **2011**, *23*, 1428-1432.
16. T. Schwich, M. P. Cifuentes, P. A. Gugger, M. Samoc, M. G. Humphrey, *Adv. Mater.* **2011**, *23*, 1433-1435.
17. M. G. Kuzyk, *J. Chem. Phys*. **2003**, *119*, 8327-8334.
18. L. Ravotto, S. L. Meloni, T. V. Esipova, A. E. Masunov, J. M. Anna, S. A. Vinogradov, *J. Phys. Chem. A* **2020**, *124*, 11038-11050.
19. C. Jin, F. Liang, J. Wang, L. Wang, J. Liu, X. Liao, T. W. Rees, B. Yuan, H. Wang, Y. Shen, *Angew. Chem. Int. Ed.* **2020**, *59*, 15987-15991.
20. S. Bhattacharya, C. Biswas, S. S. K. Raavi, J. V. S. Krishna, D. Koteshwar, L. Giribabu, S. V. Rao, *RSC Adv.* **2019**, *9*, 36726-36741.
21. T. Schwich, A. Barlow, M. P. Cifuentes, J. Szeremeta, M. Samoc, M. G. Humphrey, *Chem. Eur. J.* **2017**, *23*, 8395-8399.
22. A. V. Kazak, M. A. Marchenkova, K. S. Khorkov, D. A. Kochuev, A. V. Rogachev, I. V. Kholodkov, N. V. Usol'tseva, M. S. Savelyev, A. Y. Tolbin, *Appl. Surf. Sci.* **2021**, *545*, 148993.

23. M. Bharati, S. Bhattacharya, J. S. Krishna, L. Giribabu, S. V. Rao, *Opt. Laser Technol.* **2018**, *108*, 418-425.
24. B. Gao, L. M. Mazur, M. Morshedi, A. Barlow, H. Wang, C. Quintana, C. Zhang, M. Samoc, M. P. Cifuentes, M. G. Humphrey, *Chem. Commun.* **2016**, *52*, 8301-8304.
25. Z. Feng, D. Li, M. Zhang, T. Shao, Y. Shen, X. Tian, Q. Zhang, S. Li, J. Wu, Y. Tian, *Chem. Sci.* **2019**, *10*, 7228-7232.
26. Q. Zhang, X. Lu, H. Cao, H. Wang, T. Zhu, X. Tian, D. Li, H. Zhou, J. Wu, Y. Tian, *ACS Appl. Bio Mater.* **2020**, *3*, 8105-8112.
27. D. S. Philips, S. Sreejith, T. He, N. V. Menon, P. Anees, J. Mathew, S. Sajikumar, Y. Kang, M. C. Stuparu, H. Sun, *Chem. Asian J.* **2016**, *11*, 1523-1527.
28. Z. Zhu, J. Qian, X. Zhao, W. Qin, R. Hu, H. Zhang, D. Li, Z. Xu, B. Z. Tang, S. He, *ACS Nano* **2016**, *10*, 588-597.
29. H. Chen, F. Wang, T. Wang, *Tetrahedron Lett.* **2021**, *67*, 152892.
30. T.-C. Lin, W. Chien, L. M. Mazur, Y.-Y. Liu, K. Jakubowski, K. Matczyszyn, M. Samoc, R. W. Amini, *J. Mater. Chem. C* **2017**, *5*, 8219-8232.
31. Y. Wang, M. Chen, N. Alifu, S. Li, W. Qin, A. Qin, B. Z. Tang, J. Qian, *ACS Nano* **2017**, *11*, 10452-10461.
32. K. P. K. Naik, V. Sreeramulu, E. Ramya, K. Muralidharan, D. N. Rao, *Mater. Chem. Phys.* **2016**, *180*, 38-45.
33. P. Tejkiran, M. B. Teja, P. S. S. Kumar, P. Sankar, R. Philip, S. Naveen, N. Lokanath, G. N. Rao, *J. Photochem. Photobiol. A: Chem.* **2016**, *324*, 33-39.
34. C. Ren, X. Deng, W. Hu, J. Li, X. Miao, S. Xiao, H. Liu, Q. Fan, K. Wang, T. He, *Chem. Commun.* **2019**, *55*, 5111-5114.
35. I. E. H. Elhussin, S. Zhang, J. Liu, D. Li, Q. Zhang, S. Li, X. Tian, J. Wu, Y. Tian, *Chem. Commun.* **2020**, *56*, 1859-1862.
36. X. Zhang, S. Cao, L. Huang, L. Chen, X. Ouyang, *Dyes Pigm.* **2017**, *145*, 110-115.
37. L. Guo, X. Liu, T. Zhang, H.-B. Luo, H. H. Fan, M. S. Wong, *J. Mater. Chem. C* **2020**, *8*, 1768-1772.
38. Y. Jiang, K. F. Li, K. Gao, H. Lin, H. L. Tam, Y. Y. Liu, Y. Shu, K. L. Wong, W. Y. Lai, K. W. Cheah, *Angew. Chem. Int. Ed.* **2021**, *60*, 10007-10015.
39. B. Karthikeyan, R. Udayabhaskar, S. Hariharan, *Appl. Phys. Lett.* **2016**, *109*, 021904.
40. M. Liu, B. Gu, W. Wu, Y. Duan, H. Liu, X. Deng, M. Fan, X. Wang, X. Wei, K.-T. Yong, *Chem. Mater.* **2020**, *32*, 6437-6443.
41. E. Ramya, C. Rajashree, P. Nayak, D. N. Rao, *Appl. Clay Sci.* **2017**, *150*, 323-332.
42. H. Pan, H. Chu, Y. Li, N. Qi, S. Zhao, G. Li, D. Li, *Nanotechnology* **2020**, *31*, 195703.
43. Y. Wang, X. Han, W. Xi, J. Li, A. W. Roe, P. Lu, J. Qian, *Adv. Healthc. Mater.* **2017**, *6*, 1700685.
44. J. K. Zareba, J. Szeremeta, M. Waszkielewicz, M. Nyk, M. Samoć, *Inorg. Chem.* **2016**, *55*, 9501-9504.
45. R. Medishetty, L. Nemec, V. Nalla, S. Henke, M. Samoć, K. Reuter, R. A. Fischer, *Angew. Chem. Int. Ed.* **2017**, *56*, 14743-14748.
46. M. Liu, H. S. Quah, S. Wen, Y. Li, J. J. Vittal, W. Ji, *J. Phys. Chem. C* **2018**, *122*, 777-781.
47. Y. Sun, H. Li, X. Gao, Z. Yu, Z. Huang, C. Zhang, *Adv. Opt. Mater.* **2021**, 2100622.

48. A. Pramanik, K. Gates, Y. Gao, S. Begum, P. Chandra Ray, *J. Phys. Chem. C* **2019**, *123*, 5150-5156.
49. D. Yang, S. Chu, Y. Wang, C. K. Siu, S. Pan, S. F. Yu, *Opt. Lett.* **2018**, *43*, 2066-2069.
50. W. Chen, S. Bhaumik, S. A. Veldhuis, G. Xing, Q. Xu, M. Grätzel, S. Mhaisalkar, N. Mathews, T. C. Sum, *Nat. Commun.* **2017**, *8*, 1-9.
51. T. He, J. Li, X. Qiu, S. Xiao, C. Yin, X. Lin, *Adv. Opt. Mater.* **2018**, *6*, 1800843.
52. J. Li, Q. Jing, S. Xiao, Y. Gao, Y. Wang, W. Zhang, X. W. Sun, K. Wang, T. He, *J. Phys. Chem. Lett.* **2020**, *11*, 4817-4825.
53. K. Krishnakanth, S. Seth, A. Samanta, S. V. Rao, *Nanoscale* **2019**, *11*, 945-954.
54. T. He, J. Li, X. Qiu, S. Xiao, X. Lin, *Photonics Res.* **2018**, *6*, 1021-1027.
55. H. Syed, W. Kong, V. Mottamchetty, K. J. Lee, W. Yu, V. R. Soma, J. Yang, C. Guo, *Adv. Opt. Mater.* **2020**, *8*, 1901766.
56. F. Zhao, J. Li, J. Yu, Z. Guo, S. Xiao, Y. Gao, R. Pan, T. He, R. Chen, *J. Phys. Chem. C* **2020**, *124*, 27169-27175.
57. F. O. Saouma, D. Y. Park, S. H. Kim, M. S. Jeong, J. I. Jang, *Chem. Mater.* **2017**, *29*, 6876-6882.
58. Z. Chen, Q. Zhang, M. Zhu, H. Chen, X. Wang, S. Xiao, K. P. Loh, G. Eda, J. Meng, J. He, *J. Phys. Chem. Lett.* **2021**, *12*, 7010-7018.
59. N. Priyadarshani, T. S. Girisun, S. V. Rao, *Opt. Mater.* **2017**, *66*, 534-541.
60. G. Wang, S. Xiao, Y. Peng, Y. Wang, C. Yuan, J. He, *Opt. Lett.* **2019**, *44*, 179-182.
61. M. Sajna, S. Perumbilavil, V. Prakashan, M. Sanu, C. Joseph, P. Biju, N. Unnikrishnan, *Mater. Res. Bull.* **2018**, *104*, 227-235.
62. M. V. Rao, V. R. K. Kumar, N. Shihab, D. N. Rao, *Opt. Laser Technol.* **2018**, *107*, 110-115.
63. M. V. Rao, V. R. K. Kumar, N. Shihab, D. N. Rao, *Opt. Mater.* **2018**, *84*, 178-183.
64. X.-Y. Yan, C.-B. Yao, J. Li, J.-Y. Hu, Q.-H. Li, S.-B. Yang, *Opt. Mater.* **2016**, *55*, 73-77.
65. M. Molli, P. Pradhan, D. Dutta, A. Jayaraman, A. B. Kademane, V. S. Muthukumar, V. Kamisetti, R. Philip, *Appl. Phys. A* **2016**, *122*, 549.
66. O. H. Heckl, B. J. Bjork, G. Winkler, P. B. Changala, B. Spaun, G. Porat, T. Q. Bui, K. F. Lee, J. Jiang, M. E. Fermann, *Opt. Lett.* **2016**, *41*, 5405-5408.
67. B. Zhu, F. Wang, C. Wang, Y. Cao, L. Guo, J. Zhang, Y. Gu, *J. Condens. Matter Phys.* **2016**, *28*, 285801.
68. B. Zhu, F. Wang, K. Zhang, J. Zhang, Y. Gu, *Appl. Phys. Express* **2016**, *9*, 082602.
69. J. N. Acharyya, D. N. Rao, M. Adnan, C. Raghavendar, R. Gangineni, G. V. Prakash, *Adv. Mater. Interfaces* **2020**, *7*, 2000035.
70. N. S. Babu, *Mater. Today: Proc.* **2021**, *45*, 3976-3981.
71. Q. Tong, Y.-H. Wang, X.-X. Yu, B. Wang, Z. Liang, M. Tang, A.-S. Wu, H.-J. Zhang, F. Liang, Y.-F. Xie, J. Wang, *Nanotechnology* **2018**, *29*, 165706.
72. L. Jiang, H. Ding, M. Xu, X. Hu, S. Li, M. Zhang, Q. Zhang, Q. Wang, S. Lu, Y. Tian, *Small* **2020**, *16*, 2000680.
73. W. Zhang, Y. Ni, X. Xu, W. Lu, P. Ren, P. Yan, C. K. Siu, S. Ruan, S. F. Yu, *Nanoscale* **2017**, *9*, 5957-5963.
74. Q. Zhang, S. Wang, Y. Zhu, C. Zhang, H. Cao, W. Ma, X. Tian, J. Wu, H. Zhou, Y. Tian, *Inorg. Chem.* **2021**, *60*, 2362-2371.

75. J. Li, F. Zhao, S. Xiao, J. Cheng, X. Qiu, X. Lin, R. Chen, T. He, *Opt. Lett.* **2019**, *44*, 3873-3876.
76. M. R. Ferdinandus, J. J. Gengler, K. L. Averett, K. T. Zawilski, P. G. Schunemann, C. M. Liebig, *Opt. Mater. Express* **2020**, *10*, 2066-2074.
77. B. Monoszlai, P. Nugraha, G. Tóth, G. Polónyi, L. Pálfalvi, L. Nasi, Z. Ollmann, E. J. Rohwer, G. Gäumann, J. Hebling, *Opt. Express* **2020**, *28*, 12352-12362.
78. S. Ren, Y. Ren, S. Hu, Y. Zhao, B. Shen, L. Hong, R. Hu, F. Zhou, J. Qu, L. Liu, *IEEE Photonics J.* **2019**, *11*, 1-9.
79. D. Yang, C. Xie, X. Xu, P. You, F. Yan, S. F. Yu, *Adv. Opt. Mater.* **2018**, *6*, 1700992.
80. M. Samoc, T. C. Corkery, A. M. McDonagh, M. P. Cifuentes, M. G. Humphrey, *Aust. J. Chem.* **2011**, *64*, 1269-1273.
